# Supplementary material for: Implementation factors influencing the sustained provision of tele-audiology services: insights from a combined methodology of scoping review and qualitative semistructured interviews
Source: BMJ Open. 2023 Oct 20;13(10):e075430. doi: 10.1136/bmjopen-2023-075430 (PMC10603431; doi:10.1136/bmjopen-2023-075430)
Supplement: Supplementary data [file bmjopen-2023-075430supp002.pdf]

Supplemental file 2
Search strategies used for the article selection

- ((((((((((((((telehealth[Title]) OR (telepractice[Title])) OR (teleaudiology[Title])) OR (telemedicine[Title])) OR (telerehabilitation[Title])) OR (teletherapy[Title])) OR (mhealth[Title])) OR (ehealth[Title])) OR (internet-based[Title])) OR (computer-based[Title])) OR (web-based[Title])) OR (tablet-based[Title])) OR (mobile-based[Title])) OR (application[Title])) AND (Audiology[Title])) OR (Aural rehabilitation[Title])) OR (teleaudiology[Title]) Filters: English, from 2010 - 2023
- ((((((((((((((((((((((telehealth[Title]) OR (telepractice[Title])) OR (teleaudiology[Title])) OR (telemedicine[Title])) OR (telerehabilitation[Title])) OR (teletherapy[Title])) OR (mhealth[Title])) OR (ehealth[Title])) OR (internet-based[Title])) OR (computer-based[Title])) OR (web-based[Title])) OR (tablet-based[Title])) OR (mobile-based[Title])) OR (application[Title])) AND (Audiology[Title])) OR (Aural rehabilitation[Title])) OR (teleaudiology[Title])) OR (Auditory verbal therapy)) OR (hearing loss)) OR (hearing screening)) OR (ear care)) AND (synchronous)) OR (asynchronous)) AND (Adoption)) OR (implementation) Filters: English, from 2010 - 2024
- (((((((((((((((((((Teleaudiology[Title]) OR (Telepractice[Title])) OR (Telehealth[Title])) OR (Telemedicine[Title])) OR (Telerehabilitation[Title])) OR (Teletherapy[Title])) OR (e-health[Title])) OR (m-health[Title])) OR (Internet-based[Title])) OR (Computer-based[Title])) OR (Web-based[Title])) OR (Tablet-based[Title])) OR (Mobile-based[Title])) AND (tele-audiology[MeSH Terms])) AND (Implementation[Title/Abstract])) OR (Adoption[Title/Abstract])) OR (sustainability[Title/Abstract])) OR (acceptance[Title/Abstract])) OR (perspectives[Title/Abstract])) OR (perceptions[Title/Abstract])) OR (readiness[Title/Abstract])) AND (teleaudiology[Title]) Filters: English
- ((((((Synchronous[Title]) OR (Asynchronous[Title])) OR (Hybrid[Title])) OR ("Store and forward"[Title])) OR (Videoconference[Title])) OR (Videoconferencing[Title])) OR (Real-time[Title])) AND (Teleaudiology[Title]) Filters: English
- ((((((((((((((((Telepractice[Title]) OR (Teleaudiology[Title])) OR (telehealth[Title])) OR (telemedicine[Title])) OR (teletherapy[Title])) OR (telerehabilitation[Title])) OR (e-health[Title])) OR (m-health[Title])) OR (Internet-based[Title])) OR (Computer-based[Title])) OR (Web-based[Title])) OR (Tablet-based[Title])) OR (Mobile-based[Title])) AND ("Auditory Brainstem Response"[Title])) OR (Audiometry[Title])) OR (Tympanometry[Title])) OR ("Auditory steady state response"[Title])) OR (Videotoscopy[Title]) Filters: English, from 2010 - 2023
- (((((((((((((((((((((Telepractice[Title]) OR (Teleaudiology[Title])) OR (telehealth[Title])) OR (telemedicine[Title])) OR (teletherapy[Title])) OR (telerehabilitation[Title])) OR (e-health[Title])) OR (m-health[Title])) OR (Internet-based[Title])) OR (Computer-based[Title])) OR (Web-based[Title])) OR (Tablet-based[Title])) OR (Mobile-based[Title])) AND (Cochlear Implant[Title])) OR (Hearing aids[Title])) OR (hearing aid trial[Title])) OR (hearing aid programming[Title])) OR (hearing aid trial[Title])) OR (Tinnitus[Title])) OR (Vestibular[Title])) OR (Middle ear diseases[Title])) OR (Cochlear implant mapping[Title]) Filters: English, from 2010 - 2023
- (((((((((((((((((Teleaudiology[Title/Abstract]) OR (Telehealth[Title/Abstract])) OR (Telepractice[Title/Abstract])) OR (Telemedicine[Title/Abstract])) OR (Telerehabilitation[Title/Abstract])) OR (Teletherapy[Title/Abstract])) OR (e-health[Title/Abstract])) OR (m-health[Title/Abstract])) OR (Internet-based[Title/Abstract])) OR (Computer-based[Title/Abstract])) OR (Web-based[Title/Abstract])) OR (Tablet-based[Title/Abstract])) OR (Mobile-

based[Title/Abstract])) AND (Audiology[Title/Abstract])) OR (Aural rehabilitation[Title])) OR (Auditory verbal therapy[Title])) OR (Ear care[Title])) OR (Hearing care[Title])) OR (Hearing health[Title])) OR (Hearing loss[Title]) Filters: English, from 2010 - 2023

- ((((((((((((((((((Teleaudiology[Title]) OR (Telepractice[Title])) OR (Telehealth[Title])) OR (Telemedicine[Title])) OR (Telerehabilitation[Title])) OR (Teletherapy[Title])) OR (e-health[Title])) OR (m-health[Title])) OR (Internet-based[Title])) OR (Computer-based[Title])) OR (Web-based[Title])) OR (Tablet-based[Title])) OR (Mobile-based[Title])) AND (Evaluation[Title])) OR (Diagnostics[Title])) OR (Assessment[Title])) OR (Treatment[Title])) OR (Management[Title])) OR (Rehabilitation[Title])) OR (Therapy[Title]) Filters: English, from 2008 - 2024
- ((((((((((((((((((((((("Teleaudiology"[Title]) OR (Telepractice[Title])) OR (Telehealth[Title])) OR (Telemedicine[Title])) OR (Telerehabilitation[Title])) OR (Teletherapy[Title])) OR (e-health[Title])) OR (m-health[Title])) OR (Internet-based[Title])) OR (Computer-based[Title])) OR (Web-based[Title])) OR (Tablet-based[Title])) OR (Mobile-based[Title]))) AND (Synchronous[Title])) OR (Asynchronous[Title])) OR (Hybrid[Title])) OR ("Store and forward"[Title])) OR (Videoconference[Title])) OR (Videoconferencing[Title])) OR ("Real time"[Title]) Filters: English, from 2010 - 2024
- ((((((("Teleaudiology"[Title]) AND (Implementation[Title])) OR (Adoption[Title])) OR (Sustainability[Title])) OR (Acceptance[Title])) OR (Readiness[Title])) OR (Perspectives[Title])) OR (Perceptions[Title]) Filters: English, from 2010 – 2024
- ((Audiology) AND (implementation)) AND (teleaudiology)
- telehealth OR tele audiology OR telepractice AND audiology OR "aural rehabilitation" Filters: from 2010 - 2023
- telehealth OR (teleaudiology) OR telepractice AND audiology OR "aural rehabilitation" AND (implementation OR adoption) Filters: English, from 2010 - 2023
- telehealth OR teleaudiology OR telepractice OR Telemedicine AND audiology OR "aural rehabilitation" AND implementation OR adoption Filters: English, from 2008 – 2024
- (TITLE-ABS-KEY (teleaudiology) AND TITLE-ABS-KEY (implementation) OR TITLE-ABS-KEY (adoption))
- (TITLE-ABS-KEY (teleaudiology) OR TITLE-ABS-KEY (telepractice) OR TITLE-ABS-KEY (telehealth) AND TITLE-ABS-KEY (evaluation) AND TITLE-ABS-KEY (aural AND rehabilitation) AND TITLE-ABS-KEY (implementation) OR TITLE-ABS-KEY (adoption)) AND PUBYEAR > 2010 AND PUBYEAR < 2024
- (TITLE-ABS-KEY (teleaudiology) OR TITLE-ABS-KEY (telepractice) OR TITLE-ABS-KEY (telehealth) AND TITLE-ABS-KEY (aural AND rehabilitation) AND TITLE-ABS-KEY (implementation)) AND PUBYEAR > 2013 AND PUBYEAR < 2024
- (TITLE-ABS-KEY (teleaudiology) AND TITLE-ABS-KEY (implementation) OR ALL (sustainability) AND ALL (synchronous) OR ALL (asynchronous)) AND PUBYEAR > 2009
- (TITLE-ABS-KEY (teleaudiology) AND TITLE-ABS-KEY (implementation) OR ALL (acceptance) AND ALL (evaluation)) AND PUBYEAR > 2009
- (TITLE-ABS-KEY (teleaudiology) AND TITLE-ABS-KEY (implementation) OR ALL (readiness) AND ALL (evaluation) AND ALL (audiology)) AND PUBYEAR > 2009

- (TITLE-ABS-KEY (teleaudiology) AND TITLE-ABS-KEY (implementation) OR ALL (perspectives) OR ALL (perceptions)) AND PUBYEAR > 2010 AND PUBYEAR < 2024
- (TITLE-ABS-KEY (teleaudiology) AND ALL (audiology) AND ALL (pediatric) OR ALL (children)) AND PUBYEAR > 2009 AND PUBYEAR < 2024
- (TITLE-ABS-KEY (telemedicine) OR TITLE-ABS-KEY (telehealth) OR ALL (telepractice) AND ALL (audiology) OR ALL (aural)) AND PUBYEAR > 2009
- telepractice AND audiology AND evaluation OR newborn
- telepractice AND audiology AND evaluation OR newborn
- (TITLE-ABS-KEY (telerehabilitation) AND TITLE-ABS-KEY (audiology) OR TITLE-ABS-KEY (aural AND rehabilitation) OR TITLE-ABS-KEY (auditory AND verbal) AND TITLE-ABS-KEY (pediatric) OR TITLE-ABS-KEY (paediatric)) AND PUBYEAR > 2009
- (TITLE-ABS-KEY (telerehabilitation) OR TITLE-ABS-KEY (teletherapy) AND TITLE-ABS-KEY (audiology) AND TITLE-ABS-KEY (pediatric) OR TITLE-ABS-KEY (paediatric) OR TITLE-ABS-KEY (child) OR TITLE-ABS-KEY (children)) AND PUBYEAR > 2009
- (TITLE-ABS-KEY (telerehabilitation) OR TITLE-ABS-KEY (teletherapy) OR TITLE-ABS-KEY (mhealth) OR TITLE-ABS-KEY (ehealth) AND TITLE-ABS-KEY (teenage) OR TITLE-ABS-KEY (adolescent) AND TITLE-ABS-KEY (hearing AND loss) OR TITLE-ABS-KEY (hearing AND care) OR TITLE-ABS-KEY (hearing AND health)) AND PUBYEAR > 2009
- (TITLE-ABS-KEY (internet) OR TITLE-ABS-KEY (computer) OR TITLE-ABS-KEY (web) OR TITLE-ABS-KEY (tablet AND based) OR TITLE-ABS-KEY (mobile AND based) AND TITLE-ABS-KEY (adults) OR TITLE-ABS-KEY (geriatric) OR TITLE-ABS-KEY (old AND age) AND TITLE-ABS-KEY (ear AND care)) AND PUBYEAR > 2009
- (TITLE-ABS-KEY (teleaudiology) AND TITLE-ABS-KEY (internet AND based) OR TITLE-ABS-KEY (computer AND based) OR TITLE-ABS-KEY (web AND based) OR TITLE-ABS-KEY (tablet AND based) OR TITLE-ABS-KEY (mobile AND based)) AND PUBYEAR > 2009
- (TITLE-ABS-KEY (teleaudiology) AND TITLE-ABS-KEY (internet AND based) OR TITLE-ABS-KEY (computer AND based) OR TITLE-ABS-KEY (web AND based) OR TITLE-ABS-KEY (tablet AND based) OR TITLE-ABS-KEY (mobile AND based) AND TITLE-ABS-KEY (diagnostics) OR TITLE-ABS-KEY (assessments)) AND PUBYEAR > 2009
- (TITLE-ABS-KEY (teleaudiology) AND TITLE-ABS-KEY (internet AND based) OR TITLE-ABS-KEY (computer AND based) OR TITLE-ABS-KEY (web AND based) OR TITLE-ABS-KEY (tablet AND based) OR TITLE-ABS-KEY (mobile AND based) AND TITLE-ABS-KEY (treatment) OR TITLE-ABS-KEY (management) OR TITLE-ABS-KEY (rehabilitation) OR TITLE-ABS-KEY (therapy)) AND PUBYEAR > 2009
- (TITLE-ABS-KEY (teleaudiology) AND TITLE-ABS-KEY (hybrid) OR TITLE-ABS-KEY (store) OR TITLE-ABS-KEY (forward) OR TITLE-ABS-KEY (videoconference) OR TITLE-ABS-KEY (videoconferencing) OR TITLE-ABS-KEY (real AND time)) AND PUBYEAR > 2009
- (TITLE-ABS-KEY (teleaudiology) AND TITLE-ABS-KEY (auditory AND brainstem AND response) OR TITLE-ABS-KEY (audiometry) OR TITLE-ABS-KEY (tympanometry) OR TITLE-ABS-KEY (auditory AND steady AND state AND response)) AND PUBYEAR > 2009

- (TITLE-ABS-KEY (teleaudiology) AND TITLE-ABS-KEY (video-otoscopy) OR TITLE-ABS-KEY (cochlear AND implant) OR TITLE-ABS-KEY (hearing AND aids) OR TITLE-ABS-KEY (hearing AND aid AND trial) AND TITLE-ABS-KEY (hearing AND aid AND fitting)) AND PUBYEAR > 2009
- (TITLE-ABS-KEY (teleaudiology) AND TITLE-ABS-KEY (cochlear AND implant AND mapping) OR TITLE-ABS-KEY (tinnitus) OR TITLE-ABS-KEY (vestibular) OR TITLE-ABS-KEY (middle AND ear AND diseases)) AND PUBYEAR > 2009
- (TITLE-ABS-KEY (teleaudiology) OR TITLE-ABS-KEY (telepractice) OR TITLE-ABS-KEY (telehealth) AND TITLE-ABS-KEY (rural) OR TITLE-ABS-KEY (suburban) OR TITLE-ABS-KEY (urban) OR TITLE-ABS-KEY (village) OR TITLE-ABS-KEY (hamlet) OR TITLE-ABS-KEY (country) AND TITLE-ABS-KEY (audiology)) AND PUBYEAR > 2009
- (TITLE-ABS-KEY (teleaudiology) AND TITLE-ABS-KEY (community) OR TITLE-ABS-KEY (school) OR TITLE-ABS-KEY (district) OR TITLE-ABS-KEY (remote) OR TITLE-ABS-KEY (home)) AND PUBYEAR > 2009
- (TITLE-ABS-KEY (teleaudiology) AND TITLE-ABS-KEY (universities) OR TITLE-ABS-KEY (clinic) OR TITLE-ABS-KEY (pre-school) OR TITLE-ABS-KEY (day AND care) OR TITLE-ABS-KEY (primary)) AND PUBYEAR > 2009
- teleaudiology OR telepractice OR telehealth AND audiology AND implementation OR adoption OR sustainability
- teleaudiology AND audiology AND implementation OR adoption OR sustainability
- teleaudiology OR telepractice OR telehealth OR telemedicine AND audiology AND implementation OR adoption OR sustainability
- teleaudiology OR telepractice OR telehealth OR telemedicine OR "m-health" OR "e-health" AND audiology AND implementation OR adoption OR sustainability
- teleaudiology OR telepractice OR telehealth OR telemedicine OR "m-health" OR "e-health" OR "computer-based" OR "Web-based" AND audiology OR aural rehabilitation AND implementation OR adoption OR sustainability
- teleaudiology OR telepractice OR telehealth OR telemedicine OR "mobile-based" OR internet-based OR "tablet-based" OR "m-health" OR "e-health" OR "computer-based" OR "Web-based" AND audiology OR aural rehabilitation AND implementation OR adoption OR sustain
- "teleaudiology" OR telepractice OR telehealth OR telemedicine OR "mobile-based" OR internet-based OR "tablet-based" OR "m-health" OR "e-health" OR "computer-based" OR "Web-based" AND audiology OR aural rehabilitation OR "hearing loss" OR "auditory verbal t
- "teleaudiology" OR telepractice OR telehealth OR telemedicine OR "mobile-based" OR internet-based OR "tablet-based" OR "m-health" OR "e-health" OR "computer-based" OR "Web-based" AND audiology OR aural rehabilitation OR "hearing loss" OR "ear care"
- "teleaudiology" OR telepractice OR telehealth OR telemedicine OR "mobile-based" OR internet-based OR "tablet-based" OR "m-health" OR "e-health" OR "computer-based" OR "Web-based" AND audiology OR aural rehabilitation OR "hearing loss" OR "hearing health"
- "teleaudiology" OR telepractice OR telehealth OR telemedicine OR "mobile-based" OR internet-based OR "tablet-based" OR "m-health" OR "e-health" OR "computer-based" OR "Web-based" AND audiology OR aural rehabilitation OR "hearing loss" OR "hearing care"

- "teleaudiology" OR telepractice OR telehealth OR telemedicine OR "mobile-based" OR internet-based OR "tablet-based" OR "m-health" OR "e-health" OR "computer-based" OR "Web-based" AND audiology OR aural rehabilitation AND "Evaluation" OR "Diagnostics" OR "A
- "teleaudiology" OR telepractice OR telehealth OR telemedicine OR "mobile-based" OR internet-based OR "tablet-based" OR "m-health" OR "e-health" OR "computer-based" OR "Web-based" AND audiology OR aural rehabilitation AND "Treatment" OR "Management"
- "teleaudiology" OR telepractice OR telehealth OR telemedicine OR "mobile-based" OR internet-based OR "tablet-based" OR "m-health" OR "e-health" OR "computer-based" OR "Web-based" AND audiology OR aural rehabilitation AND therapy OR rehabilitation
- "teleaudiology" OR telepractice OR telehealth OR telemedicine OR "mobile-based" OR internet-based OR "tablet-based" OR "m-health" OR "e-health" OR "computer-based" OR "Web-based" AND audiology OR aural rehabilitation AND implementation
- "teleaudiology" OR telepractice OR telehealth OR telemedicine OR "mobile-based" OR internet-based OR "tablet-based" OR "m-health" OR "e-health" OR "computer-based" OR "Web-based" AND audiology OR aural rehabilitation AND implementation OR adoption
- Audiology OR aural rehabilitation AND sustainability OR acceptance AND "teleaudiology" OR telepractice OR telehealth OR telemedicine OR "mobile-based" OR internet-based OR "tablet-based" OR "m-health" OR "e-health" OR "computer-based" OR "Web-based"
- Audiology OR aural rehabilitation AND Readiness OR Perspectives OR Perceptions AND "teleaudiology" OR telepractice OR telehealth OR telemedicine OR "mobile-based" OR internet-based OR "tablet-based" OR "m-health" OR "e-health" OR "computer-based" OR "Web-b
- Pediatric OR Paediatric AND "teleaudiology" OR telepractice OR telehealth OR telemedicine OR "mobile-based" OR internet-based OR "tablet-based" OR "m-health" OR "e-health" OR "computer-based" OR "Web-b
- Infant OR children AND audiology AND "teleaudiology" OR telepractice OR telehealth OR telemedicine OR "mobile-based" OR internet-based OR "tablet-based" OR "m-health" OR "e-health" OR "computer-based" OR "Web-b
- Teenage OR Adolescent AND audiology AND "teleaudiology" OR telepractice OR telehealth OR telemedicine OR "mobile-based" OR internet-based OR "tablet-based" OR "m-health" OR "e-health" OR "computer-based" OR "Web-b
- Adults OR Geriatric OR Old age AND audiology AND "teleaudiology" OR telepractice OR telehealth OR telemedicine OR "mobile-based" OR internet-based OR "tablet-based" OR "m-health" OR "e-health" OR "computer-based" OR "Web-b
- Synchronous OR Asynchronous OR Hybrid OR "Store and forward" OR Videoconference OR Videoconferencing OR "Real-time" AND audiology AND "teleaudiology" OR telepractice OR telehealth OR telemedicine OR "mobile-based" OR internet-based OR "tablet-based" OR "m-

- "Auditory Brainstem Response" OR Audiometry AND audiology AND "teleaudiology" OR telepractice OR telehealth OR telemedicine OR "mobile-based" OR internet-based OR "tablet-based" OR "m-
- "Tympanometry" OR "Auditory steady state response" AND audiology AND "teleaudiology" OR telepractice OR telehealth OR telemedicine OR "mobile-based" OR internet-based OR "tablet-based"
- "Video-otoscopy" OR "Cochlear Implant" AND audiology AND "teleaudiology" OR telepractice OR telehealth OR telemedicine OR "mobile-based" OR internet-based OR "tablet-based"
- "Hearing aids" OR "Hearing aid trial" OR " Hearing aid fitting" AND audiology AND "teleaudiology" OR telepractice OR telehealth OR telemedicine OR "mobile-based" OR internet-based OR "tablet-based"
- "Cochlear implant mapping" OR "Tinnitus" OR "Vestibular" OR "Middle ear diseases" AND audiology AND "teleaudiology" OR telepractice OR telehealth OR telemedicine OR "mobile-based" OR internet-based OR "tablet-based"
- "Rural" OR "Suburban" OR "Urban" OR "Village" AND audiology AND "teleaudiology" OR telepractice OR telehealth OR telemedicine OR "mobile-based" OR internet-based OR "tablet-based"
- "Hamlet" OR "Country" OR "Community-based" AND audiology AND "teleaudiology" OR telepractice OR telehealth OR telemedicine OR "mobile-based" OR internet-based OR "tablet-based"
- "School-based" OR "School" OR "District" OR "Remote care" AND audiology AND "teleaudiology" OR telepractice OR telehealth OR telemedicine OR "mobile-based" OR internet-based OR "tablet-based"
- "home-based" OR "Universities" OR "Clinic" AND audiology AND "teleaudiology" OR telepractice OR telehealth OR telemedicine OR "mobile-based" OR internet-based OR "tablet-based"
- "Pre-school" OR "Day care" OR "Primary schools" AND audiology AND "teleaudiology" OR telepractice OR telehealth OR telemedicine OR "mobile-based" OR internet-based OR "tablet-based"
