# Supplementary material for: Implementation factors influencing the sustained provision of tele-audiology services: insights from a combined methodology of scoping review and qualitative semistructured interviews
Source: BMJ Open. 2023 Oct 20;13(10):e075430. doi: 10.1136/bmjopen-2023-075430 (PMC10603431; doi:10.1136/bmjopen-2023-075430)
Supplement: Supplementary data [file bmjopen-2023-075430supp003.pdf]

Supplemental file 3

Initial Decision Profile

Study code:
Full Citation:

1. **Is it an implementation study?**

   ❑ Yes ❑ No ❑ Maybe

2. **Has it been published between January, 2010 - April, 2021?**

   ❑ Yes ❑ No ❑ Maybe

3. **Did the project span across a period of 2 or more years?**

   ❑ Yes ❑ No ❑ Maybe

4. **Is it in the field of Tele-audiology?**

   ❑ Yes ❑ No ❑ Maybe

5. **Does it involve screening, diagnostic and rehabilitative services?**

   ❑ Yes ❑ No ❑ Maybe

Notes:

❑ **ELIGIBLE** ❑ **NOT ELIGIBLE**
