# Supplementary material for: Implementation factors influencing the sustained provision of tele-audiology services: insights from a combined methodology of scoping review and qualitative semistructured interviews
Source: BMJ Open. 2023 Oct 20;13(10):e075430. doi: 10.1136/bmjopen-2023-075430 (PMC10603431; doi:10.1136/bmjopen-2023-075430)
Supplement: Supplementary data [file bmjopen-2023-075430supp004.pdf]

Appendix 7 - StaRI Checklist

<table>
<tr><th rowspan="2">Checklist item</th><th rowspan="2">Item number</th><th colspan="2">Emmett et al. (2019)</th><th colspan="2">Emmett et al. (2019)</th><th colspan="2">Robler et al. (2020)</th><th colspan="2">Dharmar et al. (2016)</th></tr>
<tr><th>Implementation strategy</th><th>Intervention</th><th>Implementation strategy</th><th>Intervention</th><th>Implementation strategy</th><th>Intervention</th><th>Implementation strategy</th><th>Intervention</th></tr>
<tr><td>Title</td><td>1</td><td colspan="2">?</td><td colspan="2">?</td><td colspan="2">?</td><td colspan="2">X</td></tr>
<tr><td>Abstract</td><td>2</td><td colspan="2">?</td><td colspan="2">?</td><td colspan="2">?</td><td colspan="2">X</td></tr>
<tr><td>Introduction</td><td>3</td><td colspan="2">✓</td><td colspan="2">✓</td><td colspan="2">✓</td><td colspan="2">✓</td></tr>
<tr><td>Rationale</td><td>4</td><td>X</td><td>✓</td><td>X</td><td>✓</td><td>?</td><td>✓</td><td>X</td><td>✓</td></tr>
<tr><td>Aims and objectives</td><td>5</td><td colspan="2">✓</td><td colspan="2">✓</td><td colspan="2">✓</td><td colspan="2">?</td></tr>
<tr><td>Design</td><td>6</td><td colspan="2">?</td><td colspan="2">?</td><td colspan="2">?</td><td colspan="2">?</td></tr>
<tr><td>Context</td><td>7</td><td colspan="2">✓</td><td colspan="2">✓</td><td colspan="2">✓</td><td colspan="2">?</td></tr>
<tr><td>Targeted ‘sites’</td><td>8</td><td>✓</td><td>✓</td><td>✓</td><td>✓</td><td>✓</td><td>✓</td><td>?</td><td>✓</td></tr>
<tr><td>Description</td><td>9</td><td>✓</td><td>✓</td><td>✓</td><td>✓</td><td>✓</td><td>✓</td><td>?</td><td>✓</td></tr>
<tr><td>Sub-groups</td><td>10</td><td colspan="2">N/A</td><td colspan="2">✓</td><td colspan="2">N/A</td><td colspan="2">N/A</td></tr>
<tr><td>Outcomes</td><td>11</td><td>✓</td><td>✓</td><td>✓</td><td>✓</td><td>✓</td><td>✓</td><td>?</td><td>✓</td></tr>
<tr><td>Process evaluation</td><td>12</td><td colspan="2">✓</td><td colspan="2">✓</td><td colspan="2">✓</td><td colspan="2">✓</td></tr>
<tr><td>Economic evaluation</td><td>13</td><td>X</td><td>X</td><td>X</td><td>X</td><td>X</td><td>X</td><td>X</td><td>X</td></tr>
<tr><td>Sample size</td><td>14</td><td colspan="2">✓</td><td colspan="2">✓</td><td colspan="2">✓</td><td colspan="2">✓</td></tr>
<tr><td>Analysis</td><td>15</td><td colspan="2">✓</td><td colspan="2">✓</td><td colspan="2">✓</td><td colspan="2">✓</td></tr>
<tr><td>Sub-group analyses</td><td>16</td><td colspan="2">N/A</td><td colspan="2">✓</td><td colspan="2">N/A</td><td colspan="2">N/A</td></tr>
<tr><td>Characteristics</td><td>17</td><td colspan="2">N/A</td><td colspan="2">N/A</td><td>✓</td><td>N/A</td><td>?</td><td>✓</td></tr>
<tr><td>Outcomes</td><td>18</td><td colspan="2">N/A</td><td colspan="2">N/A</td><td>✓</td><td>N/A</td><td>?</td><td>✓</td></tr>
<tr><td>Process outcomes</td><td>19</td><td colspan="2">N/A</td><td colspan="2">N/A</td><td colspan="2">X</td><td colspan="2">X</td></tr>
<tr><td>Economic evaluation</td><td>20</td><td colspan="2">N/A</td><td colspan="2">N/A</td><td>X</td><td>X</td><td>X</td><td>X</td></tr>
<tr><td>Sub-group analyses</td><td>21</td><td colspan="2">N/A</td><td colspan="2">N/A</td><td colspan="2">N/A</td><td colspan="2">✓</td></tr>
<tr><td>Fidelity/ adaptation</td><td>22</td><td colspan="2">N/A</td><td colspan="2">N/A</td><td>✓</td><td>N/A</td><td>X</td><td>X</td></tr>
<tr><td>Contextual changes</td><td>23</td><td colspan="2">N/A</td><td colspan="2">N/A</td><td colspan="2">✓</td><td colspan="2">X</td></tr>
<tr><td>Harms</td><td>24</td><td colspan="2">N/A</td><td colspan="2">N/A</td><td colspan="2">N/A</td><td colspan="2">N/A</td></tr>
<tr><td>Structured discussion</td><td>25</td><td colspan="2">N/A</td><td colspan="2">N/A</td><td colspan="2">✓</td><td colspan="2">✓</td></tr>
<tr><td>Implications</td><td>26</td><td colspan="2">N/A</td><td colspan="2">N/A</td><td>✓</td><td>N/A</td><td>X</td><td>X</td></tr>
<tr><td>Statements</td><td>27</td><td colspan="2">✓</td><td colspan="2">✓</td><td colspan="2">✓</td><td colspan="2">✓</td></tr>
</table>

Appendix 7 - StaRI Checklist

**Checklist item**	**Item number**	**Stuart (2016)**		**W. Campbell and Hyde (2010)**		**Hatton et al. (2019a)**		**Ramkumar et al. (2018a)**	
		**Implementation strategy**	**Intervention**	**Implementation strategy**	**Intervention**	**Implementation strategy**	**Intervention**	**Implementation strategy**	**Intervention**
Title	**1**	X		X		X		X	
Abstract	**2**	X		X		✓		✓	
Introduction	**3**	✓		✓		✓		✓	
Rationale	**4**	X	✓	X	✓	X	✓	X	✓
Aims and objectives	**5**	✓		?		✓		N/A	
Design	**6**	?		?		?		?	
Context	**7**	✓		✓		✓		X	
Targeted 'sites'	**8**	✓	✓	✓	✓	✓	✓	✓	✓
Description	**9**	✓	✓	✓	✓	✓	✓	✓	✓
Sub-groups	**10**	N/A		N/A		N/A		N/A	
Outcomes	**11**	X	✓	X	X	✓	✓	X	✓
Process evaluation	**12**	X		X		✓		N/A	
Economic evaluation	**13**	X	X	X	X	✓	✓	✓	✓
Sample size	**14**	?		✓		✓		✓	
Analysis	**15**	✓		X		✓		✓	
Sub-group analyses	**16**	N/A		N/A		N/A		N/A	
Characteristics	**17**	X	✓	✓	✓	✓	✓	N/A	✓
Outcomes	**18**	X	✓	X	X	✓	✓	N/A	✓
Process outcomes	**19**	X		X		✓		N/A	
Economic evaluation	**20**	X	X	X	X	✓	✓	✓	✓
Sub-group analyses	**21**	X		N/A		N/A		N/A	
Fidelity/ adaptation	**22**	X	X	X	X	✓	X	X	X
Contextual changes	**23**	✓		✓		✓		✓	
Harms	**24**	N/A		N/A		N/A		N/A	
Structured discussion	**25**	✓		✓		✓		✓	
Implications	**26**	X	X	✓	✓	✓	✓	✓	✓
Statements	**27**	✓		?		✓		✓	

Appendix 7 - StaRI Checklist

Checklist item	Item number	Ramkumar et al. (2019)		Hofstetter et al. (2010)		Kokesh, Ferguson, and Patricoski (2011)		Smith et al. (2012)	
		Implementation strategy	Intervention	Implementation strategy	Intervention	Implementation strategy	Intervention	Implementation strategy	Intervention
Title	1	✓		X		X		?	
Abstract	2	✓		X		X		?	
Introduction	3	✓		✓		✓		✓	
Rationale	4	X	✓	X	✓	X	✓	X	X
Aims and objectives	5	?		✓		X		?	
Design	6	?		?		?		?	
Context	7	✓		X		X		X	
Targeted 'sites'	8	✓	✓	✓	✓	✓	✓	✓	✓
Description	9	✓	✓	✓	✓	✓	✓	✓	✓
Sub-groups	10	N/A		N/A		✓		N/A	
Outcomes	11	✓	✓	✓	✓	✓	✓	✓	✓
Process evaluation	12	X		X		✓		X	
Economic evaluation	13	✓	✓	✓	✓	✓	✓	X	X
Sample size	14	✓		?		?		?	
Analysis	15	✓		✓		✓		✓	
Sub-group analyses	16	N/A		N/A		✓		N/A	
Characteristics	17	✓	✓	✓	✓	✓	✓	✓	✓
Outcomes	18	✓	✓	✓	✓	✓	✓	✓	✓
Process outcomes	19	X		X		✓		X	
Economic evaluation	20	✓	✓	✓	✓	✓	✓	X	X
Sub-group analyses	21	N/A		N/A		✓		N/A	
Fidelity/ adaptation	22	?	?	X	X	X	X	X	X
Contextual changes	23	✓		X		✓		X	
Harms	24	N/A		N/A		N/A		N/A	
Structured discussion	25	✓		✓		✓		✓	
Implications	26	?	?	X	X	?	?	X	X
Statements	27	✓		✓		✓		✓	

Appendix 7 - StaRI Checklist

**Checklist item**	**Item number**	**Smith et al. (2013)**		**Smith et al. (2015)**		**Gupta et al. (2020)**		**Ravi et al. (2020)**	
		**Implementation strategy**	**Intervention**	**Implementation strategy**	**Intervention**	**Implementation strategy**	**Intervention**	**Implementation strategy**	**Intervention**
Title	**1**	✓		?		X		X	
Abstract	**2**	✓		?		?		✓	
Introduction	**3**	✓		✓		✓		✓	
Rationale	**4**	X	X	X	X	X	✓	X	✓
Aims and objectives	**5**	?		?		?		✓	
Design	**6**	?		?		?		?	
Context	**7**	X		X		?		?	
Targeted ‘sites’	**8**	?	?	?	?	✓	✓	✓	✓
Description	**9**	✓	✓	?	?	✓	✓	✓	✓
Sub-groups	**10**	N/A		✓		N/A		N/A	
Outcomes	**11**	X	✓	X	✓	X	✓	✓	✓
Process evaluation	**12**	X		X		✓		?	
Economic evaluation	**13**	X	X	X	X	X	X	✓	✓
Sample size	**14**	?		?		?		✓	
Analysis	**15**	✓		✓		✓		✓	
Sub-group analyses	**16**	N/A		✓		N/A		N/A	
Characteristics	**17**	X	✓	X	✓	✓	✓	✓	✓
Outcomes	**18**	X	✓	X	✓	X	✓	✓	✓
Process outcomes	**19**	X		X		✓		?	
Economic evaluation	**20**	X	X	X	X	X	X	✓	✓
Sub-group analyses	**21**	N/A		✓		N/A		N/A	
Fidelity/ adaptation	**22**	X	X	X	X	✓	X		
Contextual changes	**23**	X		X		✓		✓	
Harms	**24**	N/A		N/A		N/A		N/A	
Structured discussion	**25**	✓		✓		✓		✓	
Implications	**26**	X	X	X	X	X	X	X	X
Statements	**27**	✓		✓		✓		✓	

Appendix 7 - StaRI Checklist

Checklist item	Item number	Dennis, Gladden, and Noe (2012)		Novak et al. (2016)		Luryi et al. (2020)		Skarżyński et al. (2018)	
		Implementation strategy	Intervention	Implementation strategy	Intervention	Implementation strategy	Intervention	Implementation strategy	Intervention
Title	1	X		X		✓		X	
Abstract	2	X		X		X		✓	
Introduction	3	✓		✓		✓		✓	
Rationale	4	X	✓	X	✓	X	✓	X	✓
Aims and objectives	5	✓		✓		?		X	
Design	6	?		?		?		?	
Context	7	✓		✓		?		?	
Targeted 'sites'	8	✓	✓	✓	✓	?	✓	✓	✓
Description	9	✓	✓	✓	✓	?	✓	✓	✓
Sub-groups	10	N/A		N/A		N/A		N/A	
Outcomes	11	✓	✓	✓	✓	?	✓	X	X
Process evaluation	12	X		✓		✓		✓	
Economic evaluation	13	✓	✓	X	X	X	X	X	X
Sample size	14	✓		✓		?		✓	
Analysis	15	✓		✓		✓		✓	
Sub-group analyses	16	N/A		N/A		N/A		N/A	
Characteristics	17	✓	✓	✓	✓	?	✓	X	✓
Outcomes	18	✓	✓	✓	✓	?	✓	X	X
Process outcomes	19	X		✓		✓		✓	
Economic evaluation	20	✓	✓	X	X	X	X	X	X
Sub-group analyses	21	N/A		N/A		N/A		N/A	
Fidelity/ adaptation	22	X	X	X	X	X	X	X	X
Contextual changes	23	✓		X		✓		✓	
Harms	24	N/A		N/A		N/A		N/A	
Structured discussion	25	✓		✓		✓		✓	
Implications	26	✓	✓	✓	✓	?	✓	✓	✓
Statements	27	?		✓		✓		✓	

Appendix 7 - StaRI Checklist

<table>
<tr><th rowspan="2">Checklist item</th><th rowspan="2">Item number</th><th colspan="2">Skarżyński et al. (2019)</th><th colspan="2">Houston (2011)</th><th colspan="2">Blaiser et al. (2012)</th></tr>
<tr><th>Implementation strategy</th><th>Intervention</th><th>Implementation strategy</th><th>Intervention</th><th>Implementation strategy</th><th>Intervention</th></tr>
<tr><td>Title</td><td>1</td><td colspan="2">X</td><td colspan="2">X</td><td colspan="2">X</td></tr>
<tr><td>Abstract</td><td>2</td><td colspan="2">X</td><td colspan="2">X</td><td colspan="2">X</td></tr>
<tr><td>Introduction</td><td>3</td><td colspan="2">✓</td><td colspan="2">✓</td><td colspan="2">✓</td></tr>
<tr><td>Rationale</td><td>4</td><td>X</td><td>✓</td><td>X</td><td>✓</td><td>X</td><td>✓</td></tr>
<tr><td>Aims and objectives</td><td>5</td><td colspan="2">?</td><td colspan="2">?</td><td colspan="2">?</td></tr>
<tr><td>Design</td><td>6</td><td colspan="2">?</td><td colspan="2">?</td><td colspan="2">?</td></tr>
<tr><td>Context</td><td>7</td><td colspan="2">?</td><td colspan="2">?</td><td colspan="2">?</td></tr>
<tr><td>Targeted ‘sites’</td><td>8</td><td>✓</td><td>✓</td><td>✓</td><td>✓</td><td>✓</td><td>✓</td></tr>
<tr><td>Description</td><td>9</td><td>✓</td><td>✓</td><td>✓</td><td>✓</td><td>✓</td><td>✓</td></tr>
<tr><td>Sub-groups</td><td>10</td><td colspan="2">N/A</td><td colspan="2">N/A</td><td colspan="2">N/A</td></tr>
<tr><td>Outcomes</td><td>11</td><td>X</td><td>X</td><td>X</td><td>X</td><td>X</td><td>X</td></tr>
<tr><td>Process evaluation</td><td>12</td><td colspan="2">✓</td><td colspan="2">X</td><td colspan="2">✓</td></tr>
<tr><td>Economic evaluation</td><td>13</td><td>X</td><td>X</td><td>X</td><td>X</td><td>X</td><td>X</td></tr>
<tr><td>Sample size</td><td>14</td><td colspan="2">✓</td><td colspan="2">X</td><td colspan="2">X</td></tr>
<tr><td>Analysis</td><td>15</td><td colspan="2">✓</td><td colspan="2">X</td><td colspan="2">X</td></tr>
<tr><td>Sub-group analyses</td><td>16</td><td colspan="2">N/A</td><td colspan="2">N/A</td><td colspan="2">N/A</td></tr>
<tr><td>Characteristics</td><td>17</td><td>X</td><td>✓</td><td>✓</td><td>✓</td><td>✓</td><td>✓</td></tr>
<tr><td>Outcomes</td><td>18</td><td>X</td><td>X</td><td>X</td><td>X</td><td>X</td><td>X</td></tr>
<tr><td>Process outcomes</td><td>19</td><td colspan="2">✓</td><td colspan="2">X</td><td colspan="2">✓</td></tr>
<tr><td>Economic evaluation</td><td>20</td><td>X</td><td>X</td><td>X</td><td>X</td><td>X</td><td>X</td></tr>
<tr><td>Sub-group analyses</td><td>21</td><td colspan="2">N/A</td><td colspan="2">N/A</td><td colspan="2">N/A</td></tr>
<tr><td>Fidelity/ adaptation</td><td>22</td><td>X</td><td>X</td><td>X</td><td>X</td><td>X</td><td>X</td></tr>
<tr><td>Contextual changes</td><td>23</td><td colspan="2">✓</td><td colspan="2">✓</td><td colspan="2">✓</td></tr>
<tr><td>Harms</td><td>24</td><td colspan="2">N/A</td><td colspan="2">N/A</td><td colspan="2">N/A</td></tr>
<tr><td>Structured discussion</td><td>25</td><td colspan="2">✓</td><td colspan="2">✓</td><td colspan="2">✓</td></tr>
<tr><td>Implications</td><td>26</td><td>✓</td><td>X</td><td>X</td><td>X</td><td>✓</td><td>✓</td></tr>
<tr><td>Statements</td><td>27</td><td colspan="2">✓</td><td colspan="2">?</td><td colspan="2">?</td></tr>
</table>

Appendix 7 - StaRI Checklist

Checklist item	Item number	Houston and Stredler-Brown (2012)		Broekelmann (2012)		Lalios (2012)		Galvan, Case, and Todd Houston (2014)	
		Implementation strategy	Intervention	Implementation strategy	Intervention	Implementation strategy	Intervention	Implementation strategy	Intervention
Title	**1**	X		X		X		X	
Abstract	**2**	X		X		X		X	
Introduction	**3**	✅		✅		✅		✅	
Rationale	**4**	X	✅	X	✅	X	✅	X	✅
Aims and objectives	**5**	?		?		?		?	
Design	**6**	?		?		?		?	
Context	**7**	?		?		?		?	
Targeted 'sites'	**8**	✅	✅	✅	✅	✅	✅	✅	✅
Description	**9**	✅	✅	✅	✅	✅	✅	✅	✅
Sub-groups	**10**	N/A		N/A		N/A		N/A	
Outcomes	**11**	X	X	X	X	X	X	X	X
Process evaluation	**12**	✅		✅		✅		X	
Economic evaluation	**13**	X	X	X	X	X	X	X	X
Sample size	**14**	X		X		X		X	
Analysis	**15**	X		X		X		X	
Sub-group analyses	**16**	N/A		N/A		N/A		N/A	
Characteristics	**17**	✅	✅	✅	✅	✅	✅	✅	✅
Outcomes	**18**	X	X	X	X	X	X	X	X
Process outcomes	**19**	X		X		✅		X	
Economic evaluation	**20**	X	X	X	X	X	X	X	X
Sub-group analyses	**21**	N/A		N/A		N/A		N/A	
Fidelity/ adaptation	**22**	X	X	X	X	X	X	X	X
Contextual changes	**23**	✅		✅		✅		✅	
Harms	**24**	N/A		N/A		N/A		N/A	
Structured discussion	**25**	✅		✅		✅		✅	
Implications	**26**	X	X	✅	✅	X	✅	X	X
Statements	**27**	?		?		?		?	

Appendix 7 - StaRI Checklist

Checklist item	Item number	McCarthy, Muñoz, and White (2010)		McCarthy (2011)		McCarthy (2012)		Gladden, Beck, and Chandler (2015)	
		Implementation strategy	Intervention	Implementation strategy	Intervention	Implementation strategy	Intervention	Implementation strategy	Intervention
Title	**1**	X		X		X		?	
Abstract	**2**	X		X		X		?	
Introduction	**3**	✓		✓		✓		✓	
Rationale	**4**	X	✓	X	✓	X	✓	X	✓
Aims and objectives	**5**	?		?		?		✓	
Design	**6**	?		?		?		?	
Context	**7**	?		✓		?		✓	
Targeted ‘sites’	**8**	✓	✓	✓	✓	✓	✓	✓	✓
Description	**9**	✓	✓	✓	✓	✓	✓	✓	✓
Sub-groups	**10**	N/A		N/A		N/A		✓	
Outcomes	**11**	X	X	✓	X	✓	X	✓	✓
Process evaluation	**12**	X		✓		✓		✓	
Economic evaluation	**13**	X	X	X	X	X	X	X	X
Sample size	**14**	X		X		X		✓	
Analysis	**15**	X		X		X		✓	
Sub-group analyses	**16**	N/A		N/A		N/A		✓	
Characteristics	**17**	✓	✓	✓	✓	✓	✓	✓	✓
Outcomes	**18**	X	X	✓	X	✓	X	✓	✓
Process outcomes	**19**	X		✓		✓		✓	
Economic evaluation	**20**	X	X	X	X	X	X	X	X
Sub-group analyses	**21**	N/A		N/A		N/A		✓	
Fidelity/ adaptation	**22**	X	X	X	X	X	X	✓	X
Contextual changes	**23**	✓		✓		✓		✓	
Harms	**24**	N/A		N/A		N/A		N/A	
Structured discussion	**25**	✓		✓		✓		✓	
Implications	**26**	✓	✓	✓	✓	✓	✓	✓	✓
Statements	**27**	?		?		?		?	

Appendix 7 - StaRI Checklist

**Checklist item**	**Item number**	**Ratanjee-Vanmali, Swanepoel, and Laplante-Lévesque (2020)**	
		**Implementation strategy**	**Intervention**
Title	**1**	X	
Abstract	**2**	X	
Introduction	**3**	✓	
Rationale	**4**	X	✓
Aims and objectives	**5**	✓	
Design	**6**	?	
Context	**7**	?	
Targeted 'sites'	**8**	X	✓
Description	**9**	X	✓
Sub-groups	**10**	N/A	
Outcomes	**11**	X	✓
Process evaluation	**12**	X	
Economic evaluation	**13**	X	X
Sample size	**14**	✓	
Analysis	**15**	✓	
Sub-group analyses	**16**	N/A	
Characteristics	**17**	X	✓
Outcomes	**18**	X	✓
Process outcomes	**19**	X	
Economic evaluation	**20**	X	X
Sub-group analyses	**21**	N/A	
Fidelity/ adaptation	**22**	X	X
Contextual changes	**23**	✓	
Harms	**24**	N/A	
Structured discussion	**25**	✓	
Implications	**26**	X	X
Statements	**27**	✓	
