# Supplementary material for: Implementation factors influencing the sustained provision of tele-audiology services: insights from a combined methodology of scoping review and qualitative semistructured interviews
Source: BMJ Open. 2023 Oct 20;13(10):e075430. doi: 10.1136/bmjopen-2023-075430 (PMC10603431; doi:10.1136/bmjopen-2023-075430)
Supplement: Supplementary data [file bmjopen-2023-075430supp005.pdf]

Supplemental file - 5

Implementation Factors influencing the sustained provision of tele-audiology projects – mapped to Implementation Outcomes Framework (Proctor et al., 2011)

**Themes**	**Facilitators**
**Acceptability**	**Patient's perspectives:**<br>● ***Improved accessibility to service provider and multiple locations (P19) (P8) (P8) (P5)***<br>● ***Reduced waiting time and test time (P7) (P19) (P19) (P8) (PS2)***<br>● ***Close proximity/ Reasonable/ Reduced travel time to testing facility (P19) (P14) (P5) (P14)***<br>● Elimination of occupational health and safety issues related to travel (P19)<br>● ***Consistency and regularity of sessions, reduced cancellations (P15) and thereby completion of testing with minimal visits (P14)***<br>● Improved quality of life (P14)<br>● Implemented in the child's natural environment and preparing children with hearing loss for inclusion in their community, home, or school (P16)<br>● ***Comfortable with the environment and communicating in remote assembly settings (P20) (P14) (P5)***<br>● Clinician engagement: introduction to lead audiologist, personalized care, confidence, knowledge and understanding (P3)<br>● ***Families strongly recommended to visit for face-to-face consultation prior to telepractice sessions; Either family travels to consultation site or Audiologist travels to remote site (P19) (P5)***<br>● ***Provision of hybrid model as an option to hesitant parents (P15) (P18)***<br>● ***Counseling, emotional support and reassurance via videoconferencing (P19) (P20) (P18)***<br>● ***Early-intervention specialist is able to directly observe the parent-child interactions and provide responsive feedback that can be applied immediately (P19) (P19) (P8)***<br>● ***Participation and assistance from other family members is encouraged which thereby enables empowerment of parents as primary participants (P19) (P15) (P15)***<br>● Parents and caregivers readily assume their role as the primary facilitator of their child's development, and subsequently parental abilities appear to improve at a faster rate (P17)<br>● ***Presence and motivation of a local reliable and accessible VHW/ technical staff (P19) (P8)***<br>● ***A safe, secure environment is necessary to maintain a trusting and effective therapeutic relationship since the patient needs to have a sense of security and their privacy respected (P16) (P5)***<br>● Effective parent-professional partnership can be established and maintained using this delivery model (P17)<br>● Families developing greater understanding, knowledge and confidence of child's condition (P19)<br>● High quality consultation experience (P20)

	● Having a support network reduces the feelings of isolation experienced by families and provides a continuing sense of solidarity for families (P19)<br>● Interfere with the rapport and personal interaction (P11)<br>● Additional effort required to learn technology and might be intimidated by technology (P15)<br>● Contact with a personnel was not as good as during a traditional visit (P14)<br>● Lip reading cues can be difficult to perceive via telepractice and additional supports may be necessary for these parents (P19)
**Adoption**	**Provider's perspectives:**<br>● Interest among the personnel to implement the program (P12)<br>● Early intervention and cultural shifts in attitude (P20)<br>● Reducing loss to follow up (P4)<br>● Increasing the efficacy, efficiency, and equity of the EHDI program is a motivator for providers (P4)<br>● Audiologist promoting technology literacy (P21)<br>● Increased awareness regarding hearing (P6)<br>● Self-referral by older adults for hearing testing (P6)<br>● Requests for testing hearing of older siblings (P6)<br>● Planning sessions takes time (P15)
**Feasibility**	**Human resources:**<br>● Tele Practitioner should have heightened awareness of anticipation and remote management skills to assist in obtaining the learning objectives of both the parent and child (P17)<br>● Audiologist needs to take a lead role in the development, implementation and refinement of the service model (P5)<br>● ***Collaboration, coordination and communication between all personnel involved can result in reliable patient outcomes (P11) (P12) (P5)***<br>● ***Designated personnel for each role; such as IT Specialist/ Tele-technician (P6) (P15)***<br>● The onus is entrusted to the personnel recruited (P6)<br>● Community health worker and coordinator must have good interpersonal communication skills and knowledge of the local language (P9)<br>● Training conducted in local language using demonstrations and handouts (P6)<br>● Knowledge and skill retention assessed periodically post-training in a structured format (P6)<br>● Regular training and supervision is recommended to improve health workers' ability to successfully screen (P6)<br>● ***Stationary, video training and detailed training on the equipment for complex procedures (P12) (P14)***<br>● Flexibility to adapt materials according to need (P12)<br>● Personnel have to adapt and problem-solve in real time during various situations (P12)<br>● Lack of trained tele-technicians, shortage of professionals (P15)

	● Need for faculty full-time equivalency (P12)<br><br>**Infrastructure - Provider:**<br>● Mobile telemedicine van with satellite connection and built in facilities for video conferencing, testing and A/C (P6)<br>● Generous space acquisition and infrastructure support (P12)<br>● Appropriate lighting and sound with limited environmental distractions as well as adequate physical space for parent, child, and materials within view of the camera is mandated (P17)<br>● Physical limitations of operating in a sound booth (P5)<br>● Small space, low light conditions (P5)<br><br>**Organization Acceptance:**<br>● The involvement of audiology graduate students in training (P3)<br>● High level administrative support (University presidents, deans, and department chairs) (P12)<br><br>**Planning and implementation:**<br>● ***Assessment of patient needs and internal resources for program start-up and sustainability (PS3) (P20)***<br>● ***A robust business model/plan/ proposal, internal and external needs assessment is critical that is unique to the project (P20) (P9)***<br>● ***A site-visit, first visit, pre-feasibility analysis, barrier study could be useful (P9) (P5)***<br>● ***A test run / test environment to simulate operational conditions and validate functional requirements (P18) (P5)***<br>● A pilot project at the remote site to assess operability and workflow issues (P5)<br>● Optimise existing infrastructure and personnel (P5)<br>● A service-level analysis of averted costs from the perspective of the provider to determine the allocation efficiency (P5)<br>● Systematic framework for collecting data, that monitors program satisfaction and tracking the efficacy of the program (P16)<br>● Careful planning and coordination (P11)<br>● Support and buy-in (ownership) from clinical staff (audiologists and other related professionals) and organizational leaders (P20)<br>● Development of an enterprise consciousness that includes systems-thinking and broader concerns beyond a single program (P20)<br>● A systematic nature of programme implementation, can enable surveillance capability which allows to monitor changes over time and be more responsive to changing health needs in the community (P8)<br>● Development of protocols, standards of care, best practices, and toolkits to guide implementation (P20)

	● Targeted evaluation plan (P20)<br>● Openness to feedback and flexibility that allows for modification of work processes (P20)<br>● Embrace and lead a transformational period with the highest level of integrity (P20)<br>● Conduct formal assessments every 6 months to monitor patient progress (P19)<br>● Prioritize ease of use and affordability (P1)<br>● Business process interoperability (common policies and support structures) need to be achieved across health authorities (P5)<br>● Absence of a solid model for telemedicine cost analysis (P3)<br><br>**Resource management**<br>● Involvement of all stakeholders for the design of service delivery model (P5)<br>● Determination of resources to achieve standardization, interoperability of technology, and connectivity (P20)<br>● ***Memorandum of Understanding (MOU), Telehealth Service Agreement (TSA), A letter of agreement that delineates project responsibilities, equipment, supply, cost and work sharing between stakeholders (P11) (P5)***<br>● Commitment to adequate resourcing, staff education and training (P20)<br>● Strong partnerships with industry, technical for resource management(information technology, biomedical), and administration staff (P20)<br>● Incorporate aspects of social networking as well as blogging and facilitates collaboration, information sharing, and parent training (P19)<br>● Use of creative and focused lesson plans (P16)<br>● Equipment re-allocation as families leave the program (P19)<br>● EMR system with digital flow sheets, which enables documentation of audiometric, hearing health history and hearing or communication handicap data (P12)<br>● Online options for HHC may be important avenues for adults with hearing loss (P21)<br><br>**Standard Operating Protocol:**<br>● ***Materials and educational package necessary for instruction sent to the families via the postal service or over the Internet (P19) (P15) (P18)***<br>● Documentation of multimedia data for future reference using store and forward telepractice (P7)<br>● ***Electronic data integration into electronic health records (P7) (P20)***<br>● Tracking of cases for time studies and administrative purposes (P7)<br>● ***Use of strict inclusion and candidature criteria (P15) (P18)***

	● Unique identifier (UIN) on all forms and communications to allow for data linkage across sources (P1)<br>● Monitor the use of telepractice program through chart reviews in the multi organisational electronic medical record (EMR) (P1)<br>● On-going assessments and surveys (P15)<br><br>**Logistics:**<br>● Flexibility for rescheduling (P19)<br>● Accommodates for time differences by offering extended operating hours (P19)<br>● When circumstances results in cancelled appointments, personnel can seamlessly shift their attention to other clinic priorities (P5)<br>● Problem solving between the audiometric technician and audiologist can be conducted with relative ease due to consistency between multiple sites (P5)<br>● Referrals managed within fewer sessions and with an overall shorter duration of contact events<br>● Flexibility to conduct testing at different sites (P10)<br><br>**Sociocultural aspects:**<br>● Mobilizing community participation and monitoring follow up (P6)<br>● Family and student interpreters for different languages (P12)<br>● Community involvement, participation and engagement (P1)<br>● ***Continuous and on-going consultation and collaboration with local council and community in an appropriate and culturally safe manner (P8) (P8) (P8) (P1)***<br>● ***Strong indigenous leadership (P8) (P8) (P1)***<br>● ***Close alignment with primary care services in the community (P8) (P8)***<br>● Collaboration with the family's local birth-to-3 program enabled a computer to be purchased through an early intervention family grant program (P17)<br><br>**Government laws:**<br>● A state/ National database to document, maintain records, track and follow up (P3)<br>● Not covered by practice acts and state laws (P11)<br>● Not being covered by malpractice liability (P11)<br>● Challenges with providing practitioners with credentials and privileges (P11)<br><br>**Privacy and confidentiality - Provider:**<br>● Secured and encrypted virtual private network (P13)

	● The client file is maintained under that agency's privacy policies and the applicable audiology governing policy. The host site may track the number of tests performed for statistical and billing purposes, but does not record client information (P4)<br>● Including biometric records - fingerprints, retina and iris patterns, voice waves, signatures, and pictures (P16)
**Implementation cost**	**Provider:**<br>● Having multiple sources of funding could be useful; including charitable donations and government (P19) (P20)<br>● Lower total cost outcome (P10)<br>● Funding for hiring IT support<br>● Services not reimbursed by insurance in some cases (P11)<br><br>**Patient/Beneficiary:**<br>● ***Reduced cost of travelling and testing (P12) (P14) (P14)***<br>● Financial assistance for travel expenses and free on-site accommodation for initial face-to-face consultation (P19)

References:

Proctor, E., Silmere, H., Raghavan, R., Hovmand, P., Aarons, G., Bunger, A., Griffey, R., & Hensley, M. (2011). Outcomes for implementation research: Conceptual distinctions, measurement challenges, and research agenda. *Administration and Policy in Mental Health and Mental Health Services Research*, *38*(2), 65–76. https://doi.org/10.1007/s10488-010-0319-7
