# Supplementary material for: Implementation factors influencing the sustained provision of tele-audiology services: insights from a combined methodology of scoping review and qualitative semistructured interviews
Source: BMJ Open. 2023 Oct 20;13(10):e075430. doi: 10.1136/bmjopen-2023-075430 (PMC10603431; doi:10.1136/bmjopen-2023-075430)
Supplement: Supplementary data [file bmjopen-2023-075430supp008.pdf]

Supplemental file 8

Table 1a: Summary of articles identified in tele-audiology

Code	Study title	Authors	Country/ *Area*	Participants	Focus area
A12010	eEHDI : Functions and Challenges	Campbell & Hyde (2010)	Ontario, Canada<br>*Rural areas*	Infants	Diagnostic audiological evaluation
A22010	The impact of telehealth on wait time for ENT specialty care	Hofstetter, Kokesh, Ferguson, & Hood (2010)	Alaska, United States of America<br>*Rural areas*	Mixed Population	Combination of diagnostic audiological and otorhinolaryngological evaluation
A32010	Teleintervention for Infants and Young Children Who Are Deaf or Hard-of-Hearing	McCarthy, Muñoz, & White (2010)	Sydney, Australia<br>*Rural areas*	Children	Aural re/habilitation
A42011	TeleIntervention: Improving Service Delivery to Young Children With Hearing Loss and Their Families Through Telepractice	Houston (2011)	Utah, United States of America<br>*Mixed areas*	Children	Aural re/habilitation
A52011	The Alaska experience using store-and-forward telemedicine for ENT care in Alaska	Kokesh, Ferguson, & Patricoski (2011)	Alaska, United States of America<br>*Rural areas*	Mixed Population	Combination of diagnostic audiological and otorhinolaryngological evaluation
A62011	Using technology to support children with sensory disability in remote areas: the RIDBC Teleschool Model	McCarthy (2011)	Sydney, Australia<br>*Rural areas*	Children	Aural re/habilitation
A72012	Telepractice Services at Sound Beginnings at Utah State University	Blaiser, Edwards, Behl, & Munoz (2012)	Utah, United States of America<br>*Mixed areas*	Children	Aural re/habilitation
A82012	ihear® Internet Therapy Program - A Program by St. Joseph Institute for the Deaf	Broekelmann (2012)	Missouri, United States of America<br>*Rural areas*	Children - Adults	Aural re/habilitation
A92012	Telepractice in the Department of Veterans Affairs	Dennis, Gladden, & Noe (2012)	Washington, United States of America<br>*Mixed areas*	Mixed Population	Hearing aid fitting and programming

A102012	A Model of Early Intervention for Children with Hearing Loss Provided through Telepractice	Houston & Stredler-Brown (2012)	Utah, United States of America<br>*Rural areas*	Children	Aural re/habilitation
A112012	ConnectHear TeleIntervention Program	Lalios (2012)	Wisconsin, United States of America<br>*Mixed areas*	Children - Adolescents	Aural re/habilitation
A122012	RIDBC Teleschool (TM): A hub of expertise	McCarthy (2012)	Sydney, Australia<br>*Rural areas*	Children	Aural re/habilitation
A132012	A mobile telemedicine-enabled ear screening service for Indigenous children in Queensland: activity and outcomes in the first three years	Smith, Armfield, Wu, Brown, & Perry (2012)	Queensland, Australia<br>*Rural areas*	Children	Combination of diagnostic audiological and otorhinolaryngological evaluation
A142013	Changes in paediatric hospital ENT service utilisation following the implementation of a mobile, indigenous health screening service	Smith et al. (2013)	Queensland, Australia<br>*Rural areas*	Children	Combination of diagnostic audiological and otorhinolaryngological evaluation
A152014	Listening and Learning: Using Telepractice to Serve Children and Adults with Hearing Loss	Galvan, Case, & Todd Houston (2014)	Ohio, United States of America<br>*Rural areas*	Mixed Population	Aural re/habilitation
A162015	Tele-audiology: Expanding access to hearing care and enhancing patient connectivity	Gladden, Beck, & Chandler (2015)	Washington, United States of America<br>*Rural areas*	Mixed Population	Comprehensive audiological services
A172015	Monitoring ear health through a telemedicine-supported health screening service in Queensland	Smith et al. (2015)	Queensland, Australia<br>*Rural areas*	Children - Adolescents	Combination of diagnostic audiological and otorhinolaryngological evaluation
A182016	Reducing Loss to Follow-Up with Tele-audiology Diagnostic Evaluations.	Dharmar et al. (2016)	California, United States of America<br>*Rural areas*	Infants	Diagnostic audiological evaluation
A192016	The Future of Healthcare Delivery : IPE / IPP Audiology and Nursing Student / Faculty Collaboration to	Novak, Cantu, Zappler, Coco, & Champlin (2016)	Texas, United States of America<br>*Urban areas*	Mixed Population	Hearing aid fitting and programming

	Deliver Hearing Aids to Vulnerable Adults via Telehealth				
A202016	Infant Diagnostic Evaluation via Teleaudiology Following Newborn Screening in Eastern North Carolina	Stuart (2016)	North Carolina, United States of America<br>*Rural areas*	Infants	Diagnostic audiological evaluation
A212018	Cost and outcome of a community-based paediatric hearing screening programme in rural India with application of tele-audiology for follow-up diagnostic hearing assessment	Ramkumar et al. (2018)	Tamil Nadu, India<br>*Rural areas*	Children	Diagnostic audiological evaluation
A222018	Comprehensive approach to the National Network of Teleaudiology in World Hearing Center in Kajetany, Poland	Skarżyński, Świerniak, Bruski, Ludwikowski, & Skarżyński (2018)	Kajetany, Poland<br>*Rural areas*	Mixed Population	Cochlear Implants fitting and mapping
A232019	Hearing Norton Sound: Mixed methods protocol of a community randomised trial to address childhood hearing loss in rural Alaska	Emmett, Robler, Gallo, et al. (2019)	Alaska, United States of America<br>*Rural areas*	Children - Adolescents	Hearing screening
A242019	Hearing Norton Sound: a community randomised trial protocol to address childhood hearing loss in rural Alaska.	Emmett, Robler, Wang, et al. (2019)	Alaska, United States of America<br>*Rural areas*	Children - Adolescents	Hearing screening
A252019	Comprehensive Community Screening of Otological Patients by Trained Technicians Using a Telemedicine Device: An Efficient and Cost-Effective Way to Triage Patients With Ear Diseases	Gupta et al. (2019)	Delhi, India<br>*Rural areas*	Mixed Population	Combination of diagnostic audiological and otorhinolaryngological evaluation
A262019	Telehealth-enabled auditory brainstem response testing for infants living in rural communities: the British Columbia Early Hearing Program experience	Hatton, Rowlandson, Beers, & Small (2019)	British Columbia, Canada<br>*Rural areas*	Infants	Diagnostic audiological evaluation

A272019	Implementation and evaluation of a rural community-based pediatric hearing screening program integrating in-person and tele-diagnostic auditory brainstem response (ABR)	Ramkumar, Nagarajan, Shankarnarayan, Kumaravelu, & Hall (2019)	Tamil Nadu, India *Rural areas*	Children	Diagnostic audiological evaluation
A282019	Telefitting Between Kajetany, Poland and Odessa, Ukraine for Cochlear Implants	Skarżyński, Swierniak, Ludwikowski, & Bruski (2019)	Kajetany, Poland *Rural areas*	Mixed Population	Cochlear Implant fitting and mapping
A292020	Cochlear Implant Mapping Through Telemedicine—A Feasibility Study	Luryi et al. (2020)	Connecticut, United States of America *Urban areas*	Geriatric	Cochlear Implant fitting and mapping
A302020	Digital Proficiency Is Not a Significant Barrier for Taking Up Hearing Services With a Hybrid Online and Face-to-Face Model	Ratanjee-Vanmali, Swanepoel, & Laplante-Lévesque (2020)	Durban, South Africa *Urban areas*	Adults	Comprehensive audiological services
A312020	Tele-Audiological Surveillance of Middle Ear Status among Individuals with Cleft Lip and/or Palate in Rural South India	Ravi et al. (2020)	Tamil Nadu, India *Rural areas*	Mixed Population	Combination of diagnostic audiological and otorhinolaryngological evaluation
A322020	Hearing Norton Sound: community involvement in the design of a mixed methods community randomized trial in 15 Alaska Native communities	Robler et al. (2020)	Alaska, United States of America *Rural areas*	Children - Adolescents	Hearing screening

Table 1b: Summary of articles identified in tele-audiology (Contd.)

Code	Study title	Authors	Settings	Personnel/ e-helper/telepractice assistant	Type of services
A12010	eEHDI : Functions and Challenges	Campbell & Hyde (2010)	Community clinic	Remote site Technician	Tone specific ABR tracings at 500 and 2000 Hz<br>Diagnostic otoacoustic emissions (OAE) at 1 – 4 kHz
A22010	The impact of telehealth on wait time for ENT specialty care	Hofstetter, Kokesh, Ferguson, & Hood (2010)	Community clinic	Initiating provider:<br>Audiologists<br>Physicians<br>Public health nurses<br>Dentists<br>Physician assistants<br>Staff of the infant learning program<br><br>Consulting provider:<br>ENT Specialist	Patient demographics<br>Clinical data<br>Images and results from diagnostic tests
A32010	Teleintervention for Infants and Young Children Who Are Deaf or Hard-of-Hearing	McCarthy, Muñoz, & White (2010)	Home-based	Specially trained early-intervention specialist	Early intervention services
A42011	TeleIntervention: Improving Service Delivery to Young Children With Hearing Loss and Their Families Through Telepractice	Houston (2011)	Home-based	Speech-language pathologist	AV Therapy: Listening and language skills<br>Parent coaching<br>Family centered practices
A52011	The Alaska experience using store-and-forward telemedicine for ENT care in Alaska	Kokesh, Ferguson, & Patricoski (2011)	Community clinics<br>Hospitals	Health care providers	History<br>Images of the tympanic membrane<br>Audiometry<br>Tympanometry

A62011	Using technology to support children with sensory disability in remote areas: the RIDBC Teleschool Model	McCarthy (2011)	Home-based	RIDBC Teleschool staff - professionals<br>Local professionals<br>Technical support person<br>Local technical staff	Early intervention services
A72012	Telepractice Services at Sound Beginnings at Utah State University	Blaiser, Edwards, Behl, & Munoz (2012)	Home-based	Speech-language pathologist or<br>Educators of the deaf	AV Therapy: Listening and language skills
A82012	ihear® Internet Therapy Program - A Program by St. Joseph Institute for the Deaf	Broekelmann (2012)	Home-based<br>School-based	ihear therapist:<br>Deaf educator/ Speech-language pathologist/ Special educator	Effective, individualized, and interactive therapy: listening and spoken language
A92012	Telepractice in the Department of Veterans Affairs	Dennis, Gladden, & Noe (2012)	Community clinics	Clinicians<br>Audiology assistants<br>Telehealth clinical technicians<br>Information technology (IT) experts<br>Biomedical staff	Remote hearing aid programming and verification via telehealth
A102012	A Model of Early Intervention for Children with Hearing Loss Provided through Telepractice	Houston & Stredler-Brown (2012)	Home-based	Speech-language pathologist	AV Therapy: Listening and language skills<br>Parent coaching<br>Family centered practices
A112012	ConnectHear TeleIntervention Program	Lalios (2012)	Home-based	LSLS certified professionals	AV Therapy
A122012	RIDBC Teleschool (TM): A hub of expertise	McCarthy (2012)	Home-based	14 full-time equivalent professionals, including teachers, speech-language pathologists, and audiologists<br><br>Program manager	Rehabilitation and therapy services<br><br>Early Intervention Program<br>School age program<br>Group sessions<br>Preschool/school site visits<br>Audiological assessments

				Administrative/technical assistant<br>Toy librarian<br>Information technology<br>Occupational therapists<br>Audiologists<br>Psychologists	In-person sessions with RIDBC professionals
A132012	A mobile telemedicine-enabled ear screening service for Indigenous children in Queensland: activity and outcomes in the first three years	Smith, Armfield, Wu, Brown, & Perry (2012)	Mobile facilities	Local Indigenous health worker	Patient information and clinical history<br>Digital images using video-otoscope<br>Hearing assessment<br>Health risk questionnaire<br>ENT Review
A142013	Changes in paediatric hospital ENT service utilisation following the implementation of a mobile, indigenous health screening service	Smith et al. (2013)	Mobile facilities	Local Indigenous health worker	Patient information and clinical history<br>Digital images using video-otoscope<br>Hearing assessment<br>Health risk questionnaire<br>ENT Review
A152014	Listening and Learning: Using Telepractice to Serve Children and Adults with Hearing Loss	Galvan, Case, & Todd Houston (2014)	Home-based	Two Listening & Spoken Language Specialists & Certified Auditory Verbal Therapists (LSLS Cert. AVTs)	AV Therapy: Listening and language skills<br>Adult Aural Rehabilitation
A162015	Tele-audiology: Expanding access to hearing care and enhancing patient connectivity	Gladden, Beck, & Chandler (2015)	Community clinics	Trained assistant<br>Teleprovider<br>Telepresenter	Remote Hearing aid programming<br>Remote Audiometry<br>PC-sharing application<br>Remote programming of hearing aids - Smartphone app<br>Automated audiometry<br>Remote Cochlear Implant Mapping

A172015	Monitoring ear health through a telemedicine-supported health screening service in Queensland	Smith et al. (2015)	Mobile facilities	Local Indigenous health worker	Patient information and clinical history<br>Digital images using video-otoscope<br>Hearing assessment<br>Health risk questionnaire<br>ENT Review
A182016	Reducing Loss to Follow-Up with Tele-audiology Diagnostic Evaluations.	Dharmar et al. (2016)	Hospital	Telepresenter	Patient history, visualization of external structures, video otoscopy, immittance (including high-frequency tympanometry and middle ear muscle reflexes), distortion product otoacoustic emissions, auditory brainstem response with air and bone conduction, and, when indicated, auditory steady-state response<br><br>All aspects conducted remotely<br><br>Parent/ Guardian Satisfaction Survey<br>Provider Survey
A192016	The Future of Healthcare Delivery : IPE / IPP Audiology and Nursing Student / Faculty Collaboration to Deliver Hearing Aids to Vulnerable Adults via Telehealth	Novak, Cantu, Zappler, Coco, & Champlin (2016)	Community clinic	Audiology students<br>Nursing students<br>Supervising faculty	Video otoscopy - nursing student in city 2<br>Survey - audiology student in city 1<br>Selection and fitting of hearing aids - nursing student in city 2<br>Remote programming of the hearing instruments - audiology student in city 1<br>Review the owner’s manual, educate the patient on hearing aid

					parts, battery type/insertion/removal, and use of the push button volume control - nursing student in city 2<br>Real ear probe microphone measurements - audiology student in city 1
A202016	Infant Diagnostic Evaluation via Teleaudiology Following Newborn Screening in Eastern North Carolina	Stuart (2016)	Birthing centers	Remote Audiologist/ Technician<br>Clinical Telehealth Manager	Initial communication by call - option of Telemedicine or nearest diagnostic centre given<br><br>The diagnostic protocol:<br>Patient history<br>Cursory otoscopy<br>Middle-ear analysis<br>Distortion product otoacoustic emissions (DPOAEs)<br>Auditory brainstem response (ABR).
A212018	Cost and outcome of a community-based paediatric hearing screening programme in rural India with application of tele-audiology for follow-up diagnostic hearing assessment	Ramkumar et al. (2018)	Community clinic	Technician, Village health workers	DPOAE by VHW<br>Tele-ABR, in-person ABR
A222018	Comprehensive approach to the National Network of Teleaudiology in World Hearing Center in Kajetany, Poland	Skarżyński, Świerniak, Bruski, Ludwikowski, & Skarżyński (2018)	Community clinic	A monitoring clinician<br>A clinic engineer<br>Support speech specialist	Tele-consultation procedures<br><br>Remote fitting /Telefitting: ART/NTR/NRI, eSRT Programming<br><br>Telerehabilitation
A232019	Hearing Norton Sound: Mixed methods protocol of a community	Emmett, Robler, Gallo, et al. (2019)	School-based	Local Clinic staff<br>Teachers	Focus groups and community events held leading up to the

	randomised trial to address childhood hearing loss in rural Alaska			Audiologist<br>ENT Specialist	randomised trial<br>Qualitative, semi-structured interviews to elicit stakeholder perspectives on the intervention<br><br>Hearing screening day:<br>School screen<br>mHealth screen<br>Audiometric assessment<br>HEAR-QL<br>Outcomes measured on screening day:<br>Sensitivity and specificity of screening<br>Hearing loss prevalence<br>Hearing-related quality of life (HEAR-QL)
A242019	Hearing Norton Sound: a community randomised trial protocol to address childhood hearing loss in rural Alaska.	Emmett, Robler, Wang, et al. (2019)	School-based	Local Clinic staff<br>Teachers<br>Audiologist<br>ENT Specialist	Focus groups and community events held leading up to the randomised trial<br>Qualitative, semi-structured interviews to elicit stakeholder perspectives on the intervention (explanatory sequential stage<br><br>Hearing screening day:<br>School screen<br>mHealth screen<br>Audiometric assessment<br>HEAR-QL<br><br>Outcomes measured on screening day:<br>Sensitivity and specificity of screening

					Hearing loss prevalence<br>Hearing-related quality of life (HEAR-QL)
A252019	Comprehensive Community Screening of Otological Patients by Trained Technicians Using a Telemedicine Device: An Efficient and Cost-Effective Way to Triage Patients With Ear Diseases	Gupta et al. (2019)	Construction sites	Trained community health care workers	Screening, diagnosis, medical management, surgical intervention, and rehabilitation using hearing aid.<br><br>Video-otoscopy using ENTraview smartphone otoscopy device<br>Air conduction threshold screening at 4 speech frequencies: 500 Hz, 1000 Hz, 2000 Hz, and 4000 Hz.
A262019	Telehealth-enabled auditory brainstem response testing for infants living in rural communities: the British Columbia Early Hearing Program experience	Hatton, Rowlandson, Beers, & Small (2019)	Community clinics	An audiologist, audiometric technician (established hearing screeners), and a telehealth project manager	AC and BC ABR<br><br>Optional<br>Tympanometry testing with a 1000-Hz probe tone in case of elevated ABR thresholds<br>OAEs in case of SNHL<br><br>TH-ABR programme evaluation<br>Cost and time effectiveness<br>Measure of ABR test efficiency<br>Parent satisfaction
A272019	Implementation and evaluation of a rural community-based pediatric hearing screening program integrating in-person and tele-diagnostic auditory brainstem response (ABR)	Ramkumar, Nagarajan, Shankarnarayan, Kumaravelu, & Hall (2019)	Community clinic	Tele-technician<br>Village health workers	DPOAE by VHW<br>Tele-ABR, in-person ABR

A282019	Telefitting Between Kajetany, Poland and Odessa, Ukraine for Cochlear Implants	Skarżyński, Swierniak, Ludwikowski, & Bruski (2019)	Community clinic	A monitoring clinician<br>A clinic engineer<br>Support speech specialist	Remote fitting /Telefitting:<br>ART/NTR/NRI, eSRT<br>Programming
A292020	Cochlear Implant Mapping Through Telemedicine—A Feasibility Study	Luryi et al. (2020)	Community clinic	Local trained audiologist	Routine surveillance<br>Regular speech perception testing with AzBio sentence lists
A302020	Digital Proficiency Is Not a Significant Barrier for Taking Up Hearing Services With a Hybrid Online and Face-to-Face Model	Ratanjee-Vanmali, Swanepoel, & Laplante-Lévesque (2020)	Virtual clinic, home or office, or a satellite site for the clinic	Clinic Audiologist	Online Hearing Screening Test<br>Measures of Readiness and Stages of Change<br>Mobile Device and Computer Proficiency Questionnaires<br>Pure-Tone Average—Better Ear
A312020	Tele-Audiological Surveillance of Middle Ear Status among Individuals with Cleft Lip and/or Palate in Rural South India	Ravi et al. (2020)	Home-based<br>Camp based	Community workers<br>ENT consultant<br>Audiologist	Video-otoscopy<br>Pure tone audiometry<br>Tympanometry
A322020	Hearing Norton Sound: community involvement in the design of a mixed methods community randomized trial in 15 Alaska Native communities	Robler et al. (2020)	School-based	Local Clinic staff<br>Teachers<br>Audiologist<br>ENT Specialist	Focus groups and community events held leading up to the randomised trial<br>Qualitative, semi-structured interviews to elicit stakeholder perspectives on the intervention (explanatory sequential stage<br><br>Hearing screening day:<br>School screen<br>mHealth screen<br>Audiometric assessment<br>HEAR-QL<br>Outcomes measured on screening day:<br>Sensitivity and specificity of screening

					Hearing loss prevalence<br>Hearing-related quality of life (HEAR-QL)
