# Supplementary material for: Implementation factors influencing the sustained provision of tele-audiology services: insights from a combined methodology of scoping review and qualitative semistructured interviews
Source: BMJ Open. 2023 Oct 20;13(10):e075430. doi: 10.1136/bmjopen-2023-075430 (PMC10603431; doi:10.1136/bmjopen-2023-075430)
Supplement: Supplementary data [file bmjopen-2023-075430supp009.pdf]

Supplemental File – 9

Reference quotes from the semi-structured interviews

**Acceptability**	
1.	85% *(of the patients)* reported that they were very satisfied. Around 12% reported that they were satisfied. Some of them were not satisfied, but when we analyzed (*patient perspectives)* deeper, we found that this was reported only at the beginning. They wanted to have the first session on-site, at the centre, which we did. Then we found that the satisfaction level was over 95%.<br><br>- Project implementer of an ongoing telepractice service delivery program in Poland
2.	Parents were the best ambassadors of the idea when they saw that the results from the rehabilitation service were the best. So they were convinced of the method.<br><br>- Project implementer of a community-based telepractice service delivery program in India
3.	We have some testimony of parents saying, "This is awesome and it's motivational and it's informational. And I love that I didn't have to take my kid to *abc (referring to a distant location)* and get evaluated, then travel back and then travel down for surgery and travel back; I love that I could use this technology. I didn't have to travel or leave my community and my child is getting quick care and being seen." And so we had a lot of positive reception around the technology in terms of the trial. Also, when we asked parents, "Do you want to be present for this follow-up?". The idea was to try and make it very quick and not make it a scheduled appointment - not pull parents out of work to get it done. Like they've referred and then they got to the clinic and they got seen within a five-minute appointment. And so we had a lot of parents who said, "Yes, do it because it's hard for me. I got multiple kids at home or I'm working. And so that really helps me to make sure my kid gets seen."<br><br>- Project implementer of an ongoing telepractice service delivery program in the US
4.	We felt it was essential to really capture narratives of the experience that we were investigating from community members and various stakeholders. One of the strengths of the trial was that we built a stakeholder group right away. There were multiple stakeholders on the ground, including individuals like myself who have served the region for a long time and who have become trusted members of that region. We interviewed teachers, special education teachers, school, administrators, hospital administrators, and community health workers, who are basically the on-the-ground eyes and ears in local community clinics - Parents and children, youth, elders in the community…… And so for us, that's been really helpful. We've also been working with the school district on the healthcare system to better refine the

	collaboration that's kind of required, to get this implemented without the research arm pushing it forward.<br><br>- Project implementer of an ongoing telepractice service delivery program in the US
5.	People were initially saying no. Then, initially, we did a lot of awareness activities. Wherever we planned to start the screening, for one week we put up posters, banners, and awareness activities. We went to the community and started with community dramas, for awareness, because nobody was ready to get screenings done. There were new dramas and other community activities done. There were 10- 20 days of awareness campaigns done so that they *(patients)* get convinced about getting the screening done because that behaviour has to be changed. The people over there need to understand what we are trying to do and what benefit they would get out of it. Then, we initiated these screenings door-to-door. Some people agreed based on that and more people followed. That’s when screening happened.<br><br>- Project implementer of a community-based telepractice service delivery program in India
6.	So the people are seeing the mobile phone screen. So they want to know what their tympanic membrane looks like. So everybody began to say, “sir, please do it for me”. Maybe in future, we can have visual feedback or a video about hearing and oral hygiene.<br><br>- Project implementer of an ongoing community-based telepractice service delivery program in South India
7.	We know that some of what we've done is not translatable to the general United States or even globally, but the *def (referring to the program)* is really a unique entity in that, healthcare is something that they are provided because their land has become part of the United States. So they receive *def* resources, and they had that as part of a single-payer system. So there was no cost to the parent or the end-user in terms of receiving this follow-up where potentially in another part of the United States this would potentially be something that would be billed to insurance or be a cost to a parent to receive.<br><br>- Project implementer of an ongoing telepractice service delivery program in the US
8.	One thing which we realized was that when you go to camps *(to conduct video-otoscopy)*, the patient expects something free of cost. So sometimes we ask the doctor if they can provide something in the camp. So ENTs always usually carry some kind of medication. Whatever is the medical treatment, the patients get it in the ground itself which is free of cost, and that also motivates them to come to the hospital.

	- Project implementer of a community-based telepractice service delivery program in India
9.	We have to do a better job of educating the patients and the consumers, the families that are accessing these services. We try to go through a lot of that orientation …... Making sure that we're setting appropriate expectations for the family that we're working with is very important.<br><br>- Project implementer of a long term telepractice service delivery program in the US
**Adoption**	
10.	Patients with some neurological issues, some rare diseases, are more demanding and in general, it is more difficult in terms of rehabilitation. So we try to help such patients on-site, in the clinic. For example, patients with mental disorders and after *(sustaining)* some trauma. If we have some risk group, we say, "No, sorry but you cannot use the telemedicine solution because the results would be not optimal."<br><br>- Project implementer of an ongoing telepractice service delivery program in Poland
11.	It's a lot of change management, which means you need the leadership teams and those clinics organizations and private practices to be wanting to change. It is a mind shift. It's the leadership team driving the vision to understand that you're not just adding technology to a patient journey. You're using technology to assist the patient and patient journey. You're not automating things for the sake of automating things. You're automating things so that the face-to-face appointments with the patient become a more enhanced conversation. For this model to be integrated into routine service delivery, each one can integrate the way that suits their context. But a mindset change and some amount of training to be able to make decisions on what might work best in terms of which components should be integrated are necessary.<br><br>- Project implementer of a hybrid telepractice service delivery program in South Africa
12.	Having the support of your institution or your hospital is critical - you need to know what you're doing so that they can get behind it. It can also be a way to reach the community, to get funding, to do other things - to demonstrate how you're serving the patient and the families that you're working with, in all of these different ways. So, the administrators certainly signed off on it; they saw the value in providing services to the families and the adults but also how it was going to enhance the training of the graduate students. I mean, that's our primary goal at the University - to teach and train the graduate students, so that when they leave they can become speech pathologists or audiologists. And so, it met all of these criteria - teaching, research and service

	delivery- these are the three areas that most universities are most concerned about. So we're able to demonstrate how having telepractice and having that lab satisfied all those areas.<br><br>- Project implementer of a long term telepractice service delivery program in the US
13.	The most interesting learning for me is the hesitancy of audiologists to embrace the model. I think that for me has been the most surprising, even through a COVID year. I think the audiologist's resistance and scepticism of such a model is interesting and maybe that's due to the fear of change. By no means, does the model remove the audiologist or remove the value of the audiologist; it is using technology to enhance the audiologist's value because you can take the conversation to a different level. You can use the time more efficiently.<br><br>- Project implementer of a hybrid telepractice service delivery program in South Africa
**Feasibility**	
14.	A lot of it has to do with management; there is planning, proper performance and looking for funds and adjusting the project according to the funds…….. I will advise future telepractice implementers to analyze the needs and the target group and to check in with the team which will be dedicated to the project, several times. Because there could be some gaps and misunderstandings, which could influence a non-successful project.<br><br>- Project implementer of an ongoing telepractice service delivery program in Poland
15.	This is a region that has a lot of middle ear diseases. We had to work with a school. We had to go to every school, we had to screen every child, and then the referral process. So, that was labour-intensive from a data collection standpoint for the study. Community health workers would say that “I don't want to do telemedicine because it's double the work. And, I'd rather just write it all down and then talk to a doctor on the phone.” But what we see is that we don't always get the right diagnosis when that happens.<br><br>- Project implementer of an ongoing telepractice service delivery program in the US
16.	They (*technical and support staff)* try to work, they try to develop themselves and they earn money. I think salary is the first incentive and the second is the possibility of development…... So they work in something, which is a little bit unique. Of course, the money factor is important. Mostly, the incentive is looking at results. And

	the pay is also a good motivator.<br><br>- Project implementer of an ongoing telepractice service delivery program in Poland
17.	It's important to get that local connection. So that the patient, whenever he has some issues or some queries, can go directly to that person and get that sorted - that local connect is really important. It's also really important that they (*local liaison)* are able to put that commitment to work towards the community because there are very less people who want to work in communities and rural areas.<br><br>- Project implementer of a community-based telepractice service delivery program in India
18.	There were other individuals that we had brought in the stakeholder team; several native individuals from the community who served as our community outreach specialists. So she *(a community outreach specialist)* took a lot of what we were trying to say and created infographics to explain the trial in a way that through visuals and narrative. And so, I think that a lot of that helped us to gain trust….. I think we didn't experience as many cultural barriers because we went in the correct way. We identified our stakeholders right away; we used individuals who were from the region at the very beginning of the formation of the research question, and also while moving forward. And that really helps just kind of have it blend in seamlessly. Having enough focus groups in the beginning, generating awareness, bringing insight into the design of the trial. And a documentary narrative really helped us in getting the story that different stakeholders are telling…. I didn't see a lot of cultural barriers. But I think that's because we brought those people in.<br><br>- Project implementer of an ongoing telepractice service delivery program in the US
19.	You need to have a team - not only consisting of doctors and engineers, but also speech therapists. So we talk to audiophonologists and a whole management team starting from nurses and people who are dedicated to patient service; they could even be students. And this is quite important - to have that organizational service. It can not work with only one or two specialists. So having a team who worked earlier in medical centres, who know how to cope with patients, and can be dedicated to such a project. And there is no must for them to have a medical higher graduation.<br><br>- Project implementer of an ongoing telepractice service delivery program in Poland
20.	In some instances, in some communities, it worked fabulously because the school and the clinic already do a lot of hand-in-hand care. Those communication channels were well developed and seamless.

	- Project implementer of an ongoing telepractice service delivery program in the US
21.	The program is unique and is growing. It was designed and came out of sheer need and it basically has local community members in a trained program. They complete some initial tests - to check if they have enough reading and basic math skills. And then once they're in the program, then it routes them through a series of certifications. And so there are five levels of community provider. So one, two, three, four, and five as a practitioner, and then they learn different skills along each way. And so they are so integrated into healthcare that it's unbelievable. I'm involved in the training of our onboarding audiologists. I give them several cultural materials to read. I want them to come in with a very humble sense of the culture. They go through a three-day orientation of the hospital, which also is pretty culturally intensive in terms of nativity and heritage. And then we do a lot of on-the-job training from a telemedicine perspective. So that's one of the first things they learn because we do so much of it.<br><br>- Project implementer of an ongoing telepractice service delivery program in the US
22.	The instrument has to be charged completely for hours together, continuously. We were able to use it only for a maximum of four hours continuously because it has to support the LED light source and the mobile phone. And also, it'll consume more power to transfer data to the cloud by using the network. So there was a challenge in using the instrument continuously. It was not very user friendly initially, but after some time we could adapt it after the initial phase. Also, the equipment had texts that were only in English. We requested the company to modify it into the local language so that the community-based health workers are able to also read. So these were the challenges we faced initially, these were the difficulties.<br><br>- Project implementer of an ongoing community-based telepractice service delivery in South India
23.	I think that maybe the one important thing is having connectivity and that's the biggest issue right now. There's still sort of blackout areas where there's just very little connectivity or very very slow connectivity and that makes telepractice sometimes impossible to do. Most families do have internet services to some degree, but we still have some families that do not have internet. And that's the biggest problem that we run into, but not the most frequent. To figure out how they can get access to the connectivity can be a challenge from time to time. That probably happens 1% of the time; 99% of the time, they do have connectivity.<br><br>- Project implementer of a long term telepractice service delivery program in the US

24.	Undersea cables and just the continued expanse of getting closer and closer to broadband in our region has been pretty incredible, and obviously essential for this type of stuff.<br><br>- Project implementer of an ongoing telepractice service delivery program in the US
25.	Because in remote areas, the internet is still not available sometimes, it's usually store and forward technology that's used because it's much better than waiting for the community workers to come back and do it. It helps us save battery to do much more screenings and operationally that's viable also.<br><br>- Project implementer of a community-based telepractice service delivery program in India
26.	We have to be available to where the patients are and what devices they're using and not what we want them to use. Being able to connect to what they already have is going to be critical and going forward anyone starting a telepractice or telehealth program needs to realize that and that's sort of one of the best practices.<br><br>- Project implementer of a long term telepractice service delivery program in the US
27.	What's unique about *yyy (referring to a state in the US)* is that we've depended upon telemedicine early on, and so unique to conducting this trial in rural *yyy (referring to a state in the US)* is that we were building upon an established network and an established infrastructure on telemedicine. And so the goal of the study was really to use those resources that were available to us so that we knew when it came time to a sustainable solution that will continue beyond the research it's possible. We tapped into already existing infrastructure that people understand and know how to use, which was an asset for us both from completion and also a sustainability point of view. We did tweak it significantly to make it feasible.<br><br>- Project implementer of an ongoing telepractice service delivery program in the US
28.	If it wasn't the patient themselves who had a phone, it was the significant other or the caretaker. So I think digital proficiency is not a barrier. I think that's the assumption we have as audiologists, but there's always a support network around. Not always, but most of the time, there's a support network around the patient where technology is available.<br><br>- Project implementer of a hybrid telepractice service delivery program in South Africa

29.	I think we have about seven states that have passed this legislature of COMPACT now and I think when we get to *zzz (referring to a district in the US)*, then the COMPACT will go into effect. If you are in your home state and you're licensed in one of those states as a member of the COMPACT, then you can practice in any of the other states that are in the COMPACT. Though it is not an answer for everyone yet, these kinds of ideas have been out there and people have been trying to push those at the state level to get some of the legislation passed.<br><br>- Project implementer of a long term telepractice service delivery program in the US
30.	There are about eight different bills in the US Congress to make some changes permanent; to have more access to telehealth and telepractice and to have reimbursement available. So hopefully, one positive thing that may come out of this pandemic is the availability of telehealth and telepractice, but also the reimbursement that would be there for the providers to be able to provide these services.<br><br>- Project implementer of a long term telepractice service delivery program in the US
**Implementation Cost**	
31.	There was an initial investment cost and we bought high-end digital audiometers, we had a website etc. But it was the funding that allowed us to start a digital-first company. So, those costs come into play if you're starting from the ground up.<br><br>- Project implementer of a hybrid telepractice service delivery program in South Africa
32.	After an issue, an instrument got repaired and we didn't get funds to repair the instrument immediately. So suddenly, the process of intervention for hearing disorders stopped in the project. After some time, we got the funds, and we were able to procure new equipment.<br><br>- Project implementer of an ongoing community-based telepractice service delivery program in South India
33.	So Medicaid in *xxx (referring to a state in the US)* did not reimburse telepractice. The only time that they would reimburse for telepractice was if the SLP or whoever was delivering the services to a public school district and working with a child who was on Medicaid and receiving those funds. And so, no other funding of telepractice before Covid.<br><br>- Project implementer of a long term telepractice service delivery program in the US

34.	(In) the beginning, funds for the National Network of Teleaudiology were partially funded by the Norwegian funds …… for European Union. So in the beginning, it was from them. Some of the local projects are co-financed through different scientific grants. Always, we need to have our internal (financial) part, which we need to finance from our sources, which come from the services (we provide). ………. and some of them it's on a rental basis. So we don't buy flat, but we rent …. equipment…. Really, it's not easy.<br><br>- Project implementer of an ongoing telepractice service delivery program in Poland
35.	The biggest challenge was some blockage from the local insurance government, which didn't want to give us more financial support. So we have had limitations concerning that and we still have them sometimes. So, in such cases, we need to tell patients that they need to choose a private solution that they need to pay for. So it's not very comfortable for them either.<br><br>- Project implementer of an ongoing telepractice service delivery program in Poland
36.	I worked hard with all the stakeholders from the hospital to ensure that that encounter was billable, because if you're doing something that's not billable from a hospital perspective, then it's not sustainable.<br><br>- Project implementer of an ongoing telepractice service delivery program in the US
**<u>Sustainability</u>**	
37.	The first thing that we did to address sustainability was really try to utilize what was already existing in terms of technology and resources, and considered all the things that may make that encounter billable, so that the hospital could continue to provide that resource following the research project…. from a sustainability piece, we involved people who are already established resources. So, we use the health aids that were already in the clinic; there was no extra resources there. And then we used special education teachers already doing the screenings in the school so that, once we were done, it wouldn't be too abnormal for this process to continue.<br><br>Project implementer of an ongoing telepractice service delivery program in the US
38.	Having the support of the faculty and administrators, I think you know has been very positive over the years….the administrators, the colleagues, the cochlear implant surgeons and the pediatric audiologists I work with, I think they've been very supportive as well with the same kind of mindset of that we need to find different

	ways of serving these patients… so those are the kind of things that I think (*that influence sustainability)* - having the support of your institution or your hospital is really critical and that you know what you're doing and they can get behind it.<br><br>Project implementer of a long term telepractice service delivery program in the US
