# Supplementary material for: Implementation factors influencing the sustained provision of tele-audiology services: insights from a combined methodology of scoping review and qualitative semistructured interviews
Source: BMJ Open. 2023 Oct 20;13(10):e075430. doi: 10.1136/bmjopen-2023-075430 (PMC10603431; doi:10.1136/bmjopen-2023-075430)
Supplement: Supplementary data [file bmjopen-2023-075430supp007.pdf]

Supplemental file 7
Interview Guides

Interview time: ________

Hello Dr./ Mr. /Ms. /Mrs.,
I am Vidya, I am an audiologist and SLP working as a professional since 2008, and have spent all of these years in telepractice for rural community based programs here in India. I am based at SRIHER, Chennai, India.

I thank you for giving consent to participate in the study.

This interview to understand in detail about your experience (both positive and negative), in implementing your project titled_____(project title)______________.

The interview will take between 30-45 minutes to be completed.

Before we begin, I would like to know if you have any questions that I can address/ clarify to you.

We will now begin the interview. I will start recording our conversation for the purpose of transcription and analysis of data.

Interview begins

Dear Dr./ Mr. /Ms. /Mrs.,
From reading your work "___________________", I have gathered that you examined ____
____.

Guides:

1. How would you describe your efforts towards working in the area of telepractice?
E.g as research initiatives to contribute to the understanding of telepractice/ work towards immediate translation to service delivery in your work location/ or anything else?

2. Could you briefly describe the current status of implementation of your telepractice service for individuals with autism?
Sustained/ On-going/ non-viable/ waiting for a,b,c to start as routine?

3. Could you describe the **resources** that influenced (either positively or negatively) the implementation/ attempt to implement the telepractice service?
- Financial/Economic
- Human - professional, e-helpers/ care takers/ technical help/ facilitator
- Infrastructure-
- Equipment/ technology
- Technical/internet

Were there any non-adaptable factors that may have affected the implementation?

4. What about **the end-users** Comfort/ Acceptance/ Perceptions/ Sociocultural?

Motivators/ influencers
Inherent patient related factors?

5. Organizational

- Acceptance
- Policy Making/decision
- SOP
- Infrastructure for implementation (e-bills/staff allocation)
- Provider comfort/ ease/ acceptance/ perceptions?

6. Ethics/ Legality

- Government laws
- Consent and other ethical considerations

7. Test specific/ Disorder Specific implementation factors

8. Sustainability- self sustaining without external project funding
