# Supplementary material for: Implementation factors influencing the sustained provision of tele-audiology services: insights from a combined methodology of scoping review and qualitative semistructured interviews
Source: BMJ Open. 2023 Oct 20;13(10):e075430. doi: 10.1136/bmjopen-2023-075430 (PMC10603431; doi:10.1136/bmjopen-2023-075430)
Supplement: Author's manuscript [file bmjopen-2023-075430.draft_revisions.pdf]

BMJ Open is committed to open peer review. As part of this commitment we make the peer review history of every article we publish publicly available.

When an article is published we post the peer reviewers' comments and the authors' responses online. We also post the versions of the paper that were used during peer review. These are the versions that the peer review comments apply to.

The versions of the paper that follow are the versions that were submitted during the peer review process. They are not the versions of record or the final published versions. They should not be cited or distributed as the published version of this manuscript.

BMJ Open is an open access journal and the full, final, typeset and author-corrected version of record of the manuscript is available on our site with no access controls, subscription charges or pay-per-view fees (http://bmjopen.bmj.com).

If you have any questions on BMJ Open's open peer review process please email
info.bmjopen@bmj.com

BMJ Open

Implementation Factors Influencing the Sustained Provision of Tele-Audiology Services

Journal:	*BMJ Open*
Manuscript ID	bmjopen-2023-075430
Article Type:	Original research
Date Submitted by the Author:	08-May-2023
Complete List of Authors:	Ramkumar, Vidya; Sri Ramachandra Institute of Higher Education and Research (Deemed to be University), Department of Audiology<br>Shankar, Varsha; Sri Ramachandra Institute of Higher Education and Research (Deemed to be University), Department of Audiology<br>Kumar, Shuba; Samarth NGO, Department of Social Sciences
Keywords:	Audiology < OTOLARYNGOLOGY, QUALITATIVE RESEARCH, Telemedicine < BIOTECHNOLOGY & BIOINFORMATICS, Feasibility Studies, COVID-19, Information technology < BIOTECHNOLOGY & BIOINFORMATICS

SCHOLARONE™
Manuscripts

I, the Submitting Author has the right to grant and does grant on behalf of all authors of the Work (as defined in the below author licence), an exclusive licence and/or a non-exclusive licence for contributions from authors who are: i) UK Crown employees; ii) where BMJ has agreed a CC-BY licence shall apply, and/or iii) in accordance with the terms applicable for US Federal Government officers or employees acting as part of their official duties; on a worldwide, perpetual, irrevocable, royalty-free basis to BMJ Publishing Group Ltd ("BMJ") its licensees and where the relevant Journal is co-owned by BMJ to the co-owners of the Journal, to publish the Work in this journal and any other BMJ products and to exploit all rights, as set out in our licence.

The Submitting Author accepts and understands that any supply made under these terms is made by BMJ to the Submitting Author unless you are acting as an employee on behalf of your employer or a postgraduate student of an affiliated institution which is paying any applicable article publishing charge ("APC") for Open Access articles. Where the Submitting Author wishes to make the Work available on an Open Access basis (and intends to pay the relevant APC), the terms of reuse of such Open Access shall be governed by a Creative Commons licence – details of these licences and which Creative Commons licence will apply to this Work are set out in our licence referred to above.

Other than as permitted in any relevant BMJ Author's Self Archiving Policies, I confirm this Work has not been accepted for publication elsewhere, is not being considered for publication elsewhere and does not duplicate material already published. I confirm all authors consent to publication of this Work and authorise the granting of this licence.

For peer review only

Implementation Factors Influencing the Sustained Provision of Tele-Audiology Services

Vidya Ramkumar*, Varsha Shankar, Shuba Kumar*****

* Professor, Department of Audiology, DBT - Wellcome Trust India Alliance, Intermediate Fellow in Clinical and Public Health Research, Sri Ramachandra Faculty of Audiology and Speech Language Pathology, Sri Ramachandra Institute of Higher Education and Research (DU), Chennai - 600 116, Tamil Nadu, India

vidya.ramkumar@sriramachandra.edu.in

**PhD Research Scholar, Department of Audiology, Sri Ramachandra Faculty of Audiology and Speech Language Pathology, Sri Ramachandra Institute of Higher Education and Research (DU), Chennai - 600 116, Tamil Nadu, India

varsha.shankar1997@sriramachandra.edu.in

***SAMARTH, Chennai, Tamil Nadu 600004, India

shubakumar@samarthngo.org

Corresponding author:

Dr. Vidya Ramkumar

Professor, Department of Audiology

DBT/Wellcome Trust India Alliance

Intermediate Fellow in Clinical and Public Health Research

Sri Ramachandra Faculty of Audiology and Speech Language Pathology

vidya.ramkumar@sriramachandra.edu.in

For peer review only - http://bmjopen.bmj.com/site/about/guidelines.xhtml

ABSTRACT

Objectives: The objectives of the current study were to (a) identify long-term tele-audiology services reported to be implemented beyond the research phase and determine whether they are sustained, (b) map the implementation process to StARI guidelines, and (c) map the factors that influenced its sustainability to the Implementation Outcomes Framework (1) to understand the gaps that must be addressed from an implementation research perspective.

Study design, setting and participants: This cross-sectional study includes a scoping review of studies reporting provision of tele-audiology services from around the world. Articles describing long-term tele-audiology implementation were included based on a set of inclusion and exclusion criteria. This was followed by semi-structured interviews (SSI) wherein six project implementers identified in the review were interviewed to obtain an in-depth understanding of factors that influenced sustainability of these tele-audiology services.

Results: Data was extracted from 32 studies that were mapped to 21 projects in tele-audiology. Provision of diagnostic and therapeutic services were predominantly carried out using synchronous/ real-time telepractice methods. The most commonly used model was the '*professional-facilitator-patient'* followed by the '*professional-patient'* model. None of the studies reported the incorporation of implementation research and/or outcome frameworks. Although, none of the long-term projects studied implementation outcomes systematically, factors that influenced sustainability of tele-audiology service delivery could be mapped to implementation outcomes of *acceptability*, *adoption, feasibility*, *implementation cost* and *sustainability*.

Conclusion: Implementation research and/or outcome framework should be used to guide the implementation processes, its evaluation and measurement of outcomes systematically in tele-audiology service delivery. When such frameworks are utilized, gaps in information regarding the context influencing implementation, reporting of fidelity, and adaptability measures can be addressed.

Keywords:

For peer review only - http://bmjopen.bmj.com/site/about/guidelines.xhtml

implementation research, implementation outcomes, tele-audiology, scoping review, semi-structured interviews, factors

Strengths and Limitations of the study:

- The study reports the provision of tele-audiology services globally.
- The method used in the current study is guided by appropriate guidelines e.g., Preferred Reporting Items for Systematic reviews and Meta-Analyses extension for Scoping Reviews (PRISMA-ScR), COnsolidated criteria for REporting Qualitative research (COREQ), and Standards for Reporting Implementation Studies (StaRI) guidelines to map the reported implementation process.
- The findings of the study can provide guidance to investigators planning future research on implementation of tele-audiology.

For peer review only

INTRODUCTION

Tele-audiology has the potential to address the gaps between demand and needs, especially where access to specialized services is limited (2,3). Tele-audiology has been explored over the last two decades for screening (4,5), diagnosis (6–8), and rehabilitation (9,10). The computerization of audiology equipment has further expanded the applications of tele-audiology (11). Several systematic and scoping reviews have examined telepractice applications for ear and hearing disorders and the role of patient-site facilitators in tele-audiology service delivery (12–16).

However, despite substantial research in tele-audiology, there is a gap between the positive research outcomes and its sustainable implementation in the real-world setting. Integration of evidence-based interventions into routine clinical and community settings requires adoption of suitable implementation strategies (1,17–20). Implementation research and outcome frameworks are helpful to achieve translation of research evidence to sustained implementation. Further, in order to achieve sustainability, outcomes suitable to the stage of implementation must be periodically monitored to make suitable adaptations (1). However, in general, implementation of allied health services has not been guided by implementation research (21).

Particularly with regard to tele-audiology services, there is a lack of data on sustained implementation efforts and the factors that influenced their sustainability. As a result, the current study's objectives were to(a) identify long-term tele-audiology services reported to be implemented beyond the research phase and determine whether they are sustained, (b) map the implementation process to StARI guidelines, and (c) map the factors that influenced its sustainability to Implementation Outcomes Framework (IOF) (1) to understand the gaps that must be addressed from an implementation research perspective.

METHODS

Ethical clearance was obtained from the Institutional Ethics Committee (Reference Number: CSP/20/NOV/87/191) for this study. Patients or the public were not involved in the design, or conduct, or reporting, or dissemination plans in the current study.

For peer review only - http://bmjopen.bmj.com/site/about/guidelines.xhtml

This study included a scoping review followed by a qualitative component involving semi-structured interviews. The scoping review was carried out to identify long-term tele-audiology services reported to be implemented beyond the research phase. The scoping review was also used to map the implementation process to StARI guidelines. Semi-structured interviews were used to determine whether or not these services are sustained. The findings from the scoping review and the semi-structured interviews were used to map the factors that influenced its sustainability to IOF (1).

Scoping Review

The Preferred Reporting Items for Systematic reviews and Meta-Analyses extension for Scoping Reviews guidelines (PRISMA-ScR) (22) was used to develop the scoping review protocol (supplemental file 1). The Population-Context-Concept framework was used to formulate the eligibility criteria which included search terms, strategies, quality appraisal criteria and extraction methods. Search phrases were finalised after an initial pilot was carried out (supplemental file 2). An initial decision profile (supplemental file 3) was designed and created for systematic documentation of the selection of articles.

Six electronic databases (PubMed, Cochrane Library, Web of Science, Scopus, Google Scholar and ProQuest) were searched. The Rayyan software (23) was utilised for duplicate removal and article selection process. Two reviewers were involved during this entire process, and any disagreements between the two were discussed until a consensus was established.

Studies describing tele-audiology service provision (including screening, diagnostic, or rehabilitative services) to individuals of all age groups, for two or more years, based on prior research evidence were included. Studies describing "long-term" or "routine" or "sustained" tele-audiology service delivery were included. Quasi-experimental trials, and community or field trials were also included. All telepractice modalities, including video conferencing, web-based, mobile applications, and remote computing, were considered. Only literature published in English between January 2010 and April 2021 was included.

For peer review only - http://bmjopen.bmj.com/site/about/guidelines.xhtml

Studies that compared in-person and telepractice measures, or reported validity of tests or tools but did not report or mention long-term implementation were excluded. When full texts were not available after reasonable efforts, articles were excluded.

Analysis

The search results were summarised using the Preferred Reporting Items for Systematic Reviews and Meta-Analyses (PRISMA) flow diagram. We extracted information on geographical distribution, the focus area of service delivery, method and model of tele-audiology service delivery. The Standards for Reporting Implementation Studies (StaRI) guidelines (24) was used to map the implementation strategies and components reported in the studies (supplemental file 4). When a StARI criteria was addressed, it was denoted with a check mark, when it was partially addressed, it was denoted with a "?", and when it was not addressed, it was denoted with a "X." When a criterion was not relevant, it was marked with "N/A." The contents of the articles were examined for reports of factors that influenced the sustainability of tele-audiology services. Any recommendations by the implementers were also considered as influencing factors (supplemental file 5). These were then mapped to the constructs of the IOF (1).

Semi-structured interviews (SSI)

The long-term and sustained tele-audiology service implementers identified in the scoping review were included for the semi-structured interviews. This was done to assess if these services are still sustained and also exemplify the data obtained from the scoping review on the factors that influenced the sustainability of these services. The COREQ (COnsolidated criteria for REporting Qualitative research) guidelines (25) were used to report the qualitative components of the study (supplemental file 6).

Research team:

Personal characteristics and relationship with participants: Both VR and VS conducted three interviews. VR has 10 years of experience performing qualitative research; VS had no prior experience. There was no specific relationship established by the interviewers with the participants prior to the interview.

For peer review only - http://bmjopen.bmj.com/site/about/guidelines.xhtml

Information about the interviewer's interest in the research topic, as well as purpose and description of the research study was provided in the e-consent form.

Study design:

Theoretical framework and development of interview guides: We used the thematic analytical approach (26,27) as our methodological orientation for this study. The interview guides were developed based on the constructs of the IOF (1) to which the influencing factors could be mapped to. It was then verified by two experts with experience in qualitative research and telepractice. The guides were verified for content, wording and relevancy to objectives. A copy of the interview guide has been provided (supplemental file 7).

Participant selection: Primary and corresponding authors of the studies identified in the scoping review were selected as the participants for the interviews using purposive sampling method. The corresponding authors were contacted, if they were unable to participate, we requested a referral to any of the other authors of the team. If we were unable to establish contact with the corresponding author, then the contact details of the remaining authors were searched and an effort was made to contact these authors.

E-mails were sent to all the authors along with the link to an e-consent form. In a couple of instances, the authors led the investigators to another team member. Reminders were provided for scheduling the interviews and the consent form remained available for three months. Out of the 21 authors identified in the scoping review, six authors expressed consent to participate in the study.

Setting: Since interviewees belonged to different countries around the world, all interviews were conducted using an encrypted online video-conferencing platform. Other than the investigators, no one else was present during the interview. Six authors (Male=3, Female=3) in their early, mid and advanced career levels participated in the interviews. Interviews were carried out between March and August 2021.

Data collection: The interview guides were initially piloted on a project implementer and suitable adaptations were made prior to data collection. Primary guides and additional probes were used to

For peer review only - http://bmjopen.bmj.com/site/about/guidelines.xhtml

obtain rich data. The interview guides were further informed and expanded upon based on each interview, thereby enabling deeper insights.

Informed e-consent was obtained from all the participants prior to the interviews. All interviews were audio-video recorded and field notes were also taken by the interviewers. All interviews were completed within 30 to 45 minutes. The "*A Priori Thematic Saturation*" (28) model was used to assess the adequacy of data collected.

Analysis:

Data Analysis: We conducted thematic analysis of verbatim transcription of all the audio and video recorded interviews using NVivo 12 software. Thematic analysis was carried out using a hybrid inductive-deductive approach (27,29). After familiarisation with the transcripts, both the authors coded two transcripts independently and updated the existing codebook by adding codes generated deductively from the interviews. Any coding differences were discussed and resolved. Upon fine-tuning, the remaining transcripts were coded based on the updated codebook. Unique and relevant information that were identified during this process were given new codes. We organised the codes based on similarity and frequency, which aided in categorising codes. We analysed these categories for important patterns in the data that were relevant to our study objectives and compiled them under themes. Following this analysis, we evaluated the themes for relevancy and credibility. We merged certain themes together for a more succinct representation of the findings whenever necessary.

Reporting: We presented an explanation of our study findings, which were substantiated with quotes placed under the respective themes with specific qualifiers for each participant. All quotes and findings were sent back to the interviewees for comments and corrections.

For peer review only

RESULTS

The results describing, project mapping, geographical distribution of projects, focus area of service delivery, method of telepractice, model of telepractice service delivery and mapping of implementation components to the StaRI guidelines are based on the findings from the scoping review. The section addressing the factors that influenced the sustainability of tele-audiology services include findings of both the scoping review and the SSIs.

The scoping review identified 32 articles that satisfied the inclusion criteria. The PRISMA flow diagram depicts the studies search results (Figure 1). A summary of the literature reviewed based on the scoping review's inclusion criteria was tabulated for analysis. The study code, study title, authors, countries, and focus area are all listed in Table 1a (supplemental file 8). Table 1b (supplemental file 8) describes the settings, participants, employees, and types of services.

Project mapping

Studies describing similar background content in the introduction, common implementation sites, broad objectives, and a contiguous group of investigators were mapped as a single project. This exercise was undertaken to make a realistic assessment of the implementation focus. Studies were considered independent projects when they could not be mapped under any other larger project. Twenty-one projects were identified based on this mapping exercise (Table 1) and each project was coded as P (project) and serial number, for ease of reference. The subsequent findings will be reported using these project codes.

For peer review only

For peer review only - http://bmjopen.bmj.com/site/about/guidelines.xhtml

Table 1: Summary of project mapping in tele-audiology

Focus area	Country	Project	Project code
Hearing Screening	United States of America	(30–32)	P1
Diagnostic audiological evaluation	Canada	(33)	P2
		(34)	P3
	Canada	(35)	P4
		(36)	P5
	India	(37,38)	P6
Diagnostic audiological with otorhinolaryngological evaluation	Canada	(39,40)	P7
	Australia	(41–43)	P8
	India	(44)	P9
		(45)	P10
Hearing aid fitting and programming	United States of America	(46)	P11
		(47)	P12
Cochlear implant fitting and mapping	United States of America	(48)	P13
	Poland	(49,50)	P14
Aural re/habilitation	United States of America	(51–53)	P15
		(54)	P16
		(55)	P17
		(56)	P18
	Australia	(57–59)	P19
Comprehensive audiological services	United States of America	(60)	P20
	South Africa	(61)	P21

For peer review only - http://bmjopen.bmj.com/site/about/guidelines.xhtml

Geographical distribution of projects

Tele-audiology was implemented in 12 projects in the United States of America (USA); two projects in Australia; three projects in India; two projects in Canada; one project each from Poland and South Africa. Projects implemented in almost all the countries (P1, P2, P3, P4, P5, P6, P7, P8, P9, P10, P14, P16, P18, P19, P20) were delivered to overcome access barriers for individuals in rural areas and/or remote locations. However, in the US there were few projects (P11, P12, P13, P15, P17, P21) that provided tele-audiology services to urban and semi-urban areas due to scheduling related issues (work, having other children to take care of, household responsibilities).

Focus area of tele-audiology service delivery

Tele-audiology was implemented in projects with a focus on ear and hearing screening, diagnostic audiological evaluations, a combination of diagnostic audiological with otorhinolaryngological evaluation, hearing aid fitting and programming, cochlear implant fitting and mapping as well as intervention services.

Mobile phone-based ear and hearing screening was carried out among school-aged children (P1). Telepractice was used to conduct diagnostic confirmation of hearing loss among infants in follow-up to hearing screening (P2; P3; P4; P5; P6). Tele-auditory brainstem response (ABR) and tele-distortion product otoacoustic emissions (DPOAEs) were performed in all these projects. Other diagnostic tests such as immittance including high-frequency tympanometry and middle ear muscle reflexes, and Auditory Steady-State Response (ASSR) were included only in a few projects (P2, P3).

Diagnostic audiological evaluation combined with otorhinolaryngological evaluation using telepractice was reported in four projects (P7, P8, P9, and P10). In these projects, basic ear (video-otoscopy) and hearing testing (audiometry and tympanometry) was provided as a routine service to individuals of all age groups.

For peer review only - http://bmjopen.bmj.com/site/about/guidelines.xhtml

Remote hearing aid fitting, verification and programming (P11, P12) as well as remote cochlear implant switch-on and mapping (P14) were conducted for all age groups. One project focused on cochlear implant mapping for geriatric patients alone (P13). In the projects where remote cochlear implant switch-on and mapping were conducted (P13, P14), tests such as acoustic reflex threshold/neural response telemetry/ neural response imaging and e-stapedial reflex threshold was carried out through telepractice. Telepractice was used for aural re/habilitation of both children and adults (P15, P16, P17, P18, and P19) to provide auditory-verbal therapy or some form of habilitation training to individuals with hearing loss.

Comprehensive audiological services to individuals of different age groups were provided with tests including audiometry, hearing aid fitting and programming, cochlear implant fitting and mapping in one project (P20). On the other hand, another project (P21) conducted only hearing screening and hearing aid reprogramming using telepractice, for adults. The diagnostic confirmation and hearing aid fitting was done in person.

Method of telepractice

Use of synchronous/real-time, asynchronous/ store and forward, or a combination/ hybrid method for providing tele-audiology services was identified from the scoping review.

In tele-audiology, the asynchronous method of service delivery was predominantly used for audiometric screening, video-otoscopy, and tympanometry (P1, P7, P8, P9, and P10). Client details, video-otoscopic images, and audiometric or tympanometry data were stored and forwarded to an audiologist or otolaryngologist for review. For tele-diagnostic evaluation of hearing among infants, synchronous methods (for both ABR and DPOAEs) or hybrid methods of synchronous (for ABR) and asynchronous (for DPOAEs) were used (P3, P4, P5, P6). In one project (P2) all diagnostic tests (video-otoscopy, middle ear analysis, DPOAEs, ABR and ASSR) were completed synchronously. Remote hearing aid fitting and programming (P11, P12, P20, and P21) and remote cochlear implant fitting and mapping (P13, P14), were conducted

For peer review only

synchronously. In one project (P12), even real-ear probe measures were conducted synchronously. Aural habilitation services like Auditory Verbal Therapy (AVT) were provided synchronously (P15, P16, P17, P18, and P19).

Model of telepractice service delivery

When an e-helper/telepractice assistant/ support staff supported patient care, it was categorised as "*professional-facilitator-patient*' model; when the professional delivered the service directly to the patient without intermediary personnel, it was categorised as '*professional-patient*' model; and '*professional-professional*' model involved a second audiologist or any other professional at the patient site.

In tele-audiology, irrespective of test procedures, the 'professional-facilitator-patient' model was used whenever services were provided to remote rural areas. In this case, a trained e-Helper/telepractice assistant/community-based health worker served as a facilitator for telepractice (P1, P2, P3, P4, P5, P6, P8, P9, P10, P11, P12, and P20) and their roles were defined in the project. This was the most commonly used model of telepractice service delivery. The 'professional-patient' model was predominantly used for services provided through video-conferencing such as AVT and habilitation of individuals with hearing loss (P15, P16, P17, P18, P19, and P21). The 'professional-professional' model was used for fitting cochlear implants (P13, P14) when both audiological and otorhinolaryngology services were provided (P7). The professional at the patient's end was an audiologist with no specialised training in cochlear implant aural rehabilitation, while the professional who provided services via telepractice was specialised in this area (P13). In one project (P14), a speech therapist was the professional available at the patient end to facilitate the tele-fitting of cochlear implants. Otolaryngology consultations were supported at the patient end by a variety of professionals including audiologists, physicians, public health nurses, dentists, and physician assistants, as a part of a routine telemedicine clinic (P7).

For peer review only - http://bmjopen.bmj.com/site/about/guidelines.xhtml

Mapping of the implementation process to StaRI guidelines (24)

We found that none of the long-term projects used any implementation framework to implement tele-audiology service delivery. Thus, we mapped the implementation process described in the studies to the StaRI guidelines. Only two study titles (37,42) and six study abstracts (36–38,42,45,49) identified their study as "implementation" research. None of the studies mentioned the use of an underlying theory/framework/model or relevant reporting guidelines. Eleven studies provided a detailed overview of the context influencing implementation (30–32,34–37,46,47,58,60).

The outcomes of the implementation strategy were specified in 14 studies (30–32,36,37,39–41,45–47,57,58,60). While 18 studies included process evaluation objectives (30–33,36,40,44,47–50,52–55,57,58,60), only 12 studies reported the process outcomes (36,40,44,47–50,53,55,57,58,60). The economic evaluation of the implementation strategy was described only in seven studies (36,37,39,40,45,46,62). Although description of fidelity to the planned implementation strategy and contextual adaptations was reported in four studies (32,36,44,60), specific measures to assess fidelity and adaptation were not described. Fourteen studies each addressed scalability (32,35,36,38,46,47,49,50,53,54,57–60) and sustainability (35,36,38,46–49,53–55,57–60)

Mapping of the influencing factors to the IOF (1)

The results of the scoping review (denoted using the project codes) and thematic analysis of the SSIs (substantiated using quotes) were combined to map the factors that influenced sustainability of tele-audiology services. The quotes are summarized (Supplemental file 9 - Table 2) under the respective overarching themes with specific qualifiers for each project implementer.

We found that none of the projects studied implementation outcomes systematically. However, studies highlighted the factors influencing the sustainability of tele-audiology service delivery based on their

For peer review only - http://bmjopen.bmj.com/site/about/guidelines.xhtml

experiences. These reports could be mapped to the implementation outcomes of *acceptability, adoption, feasibility, implementation cost, and sustainability* (1).

Acceptability:

The beneficiary/ patient perception related factors reported by the project implementers could be mapped to the early to late-stage implementation outcome of *acceptability.* Parents were hesitant to adopt tele-audiology in the beginning as they were apprehensive about the child's ability to build rapport with the therapist. In such a case, they were given a choice of a combined approach of in-person and telepractice sessions until they felt comfortable and confident (P5, P19) or continue in-person sessions till they expressed their willingness to engage in telepractice sessions (P15, P18). The families were provided with the opportunity to meet the therapist and participate in a practice video conference session before the commencement of telepractice sessions. Such a mindful approach was found to help address patient apprehensions (refer to quote 1). Parents also became ambassadors of the program when they saw the value in it (refer to quote 2).

Patients' or caregivers' perceptions and understanding of the advantages of tele-audiology service delivery was a key facilitator (P5, P7, P8, P14, P15, P19, and P20) (refer to quote 3). Continuous and ongoing engagement with communities in a culturally safe manner and building awareness regarding the service enhanced acceptance (P1, P8) (refer to quotes 4 and 5). Reduced travel cost associated with tele-audiology services was perceived as facilitatory for implementing such services (P12, P14). Real-time presence of the professional, technical team and support staff and reassurance by them, facilitated the acceptance of tele-audiology by patients/ caregivers (P15, P18, P19, and P20). Patient's acceptance of technology use in ear and hearing care improved when visual images of tests conducted (image of tympanic membrane) were provided (refer to quote 6). Acceptance was also conditional to the absence of any direct cost to the patient (refer to quote 7) or in case of subsidised service (refer to quote 8). Patients felt secure when data security, privacy and confidentiality were protected (P5, P16). Participation in tele-audiology sessions by other family members, who otherwise would not be able to do so, was further reassuring in some instances (P15, P19). Setting

For peer review only - http://bmjopen.bmj.com/site/about/guidelines.xhtml

appropriate expectations for the patient and ensuring clear communication prior to telepractice service delivery can enable acceptance (refer to quote 09).

Adoption:

Provider perspectives regarding uptake and utilization of tele-audiology service delivery could be mapped to the early-stage implementation outcome of *adoption*. Project implementers felt that the assessment of a patient's suitability for tele-audiology was crucial and it was important to clearly communicate to the patient when telepractice might not be the best option (refer to quote 10). Adoption of new service delivery requires change and this change needs to be supported and driven by the leadership with a vision for sustainability (refer to quote 11). Organisations see value in telepractice because of their threefold advantage: teaching, research and service delivery (refer to quote 12). Hesitancy among service providers to adopt tele-audiology was considered as a challenge to implementation (refer to quote 13).

Feasibility:

Factors related to the utility and practicality of implementing tele-audiology service delivery could be mapped to the early-stage implementation outcome of *feasibility.* Assessing needs, availability of internal resources, financial planning and a service delivery model that is flexible and adaptable to change was considered useful for seamless implementation of tele-audiology services (P9, P20) (refer to quote 14). Formulating a Memorandum of Understanding (MOU) and a Telehealth Service Agreement (TSA) before implementation was reported to be facilitatory (P5, P11). Integrated electronic health records were also recommended to ease tele-audiology service implementation (P7, P20).

Limited or non-availability of professionals and technicians trained in tele-audiology service delivery was reported to be a major barrier to implementation (P15). Retention of personnel employed in tele-audiology service delivery was a challenge as it was perceived to be labour-intensive (refer to quote 15). The uniqueness of telepractice draws personnel to work in such services; however, continuity is supported by monetary benefit, opportunities for career development and positive outcomes of services (refer to quote 16). In

For peer review only - http://bmjopen.bmj.com/site/about/guidelines.xhtml

providing remote care, establishing a local "point of contact" who is dedicated and committed to assisting telepractice service delivery was beneficial (refer to quote 17). Having a liaison from the community helped minimise and overcome cultural barriers (refer to quote 18). A dedicated team including professionals, and administrative and technical support staff was considered pertinent (P6, P19) (refer to quote 19). Reliable patient outcomes were associated with team-building, coordination and streamlined communication between all collaborators involved in telepractice service delivery (P5, P11, and P13) (refer to quote 20). Further, defining the roles and responsibilities of e-helper/ telepractice assistants with systematic culture-sensitive training was considered useful (P7, P20) (refer to quote 21).

Devices with a simple user interface, minimal skill requirement, display in local language and adequate battery back-up were essential to support remote community-based tele-audiology services (refer to quote 22). Poor internet bandwidth compromised service delivery in rural areas (P10, P15, P17) (refer to quote 23) and the use of undersea cables and broadband connections were regarded as more reliable (P5, P19) (refer to quote 24). High quality dedicated video conferencing equipment was a quality enhancer in tele-audiology service delivery (P14, P19, P20). In rural areas where issues in connectivity were expected, the store and forward method of tele-audiology was found beneficial (refer to quote 25).

Capitalising on available resources (patient's own devices, pre-existing infrastructure) facilitated seamless implementation (refer to quotes 26 and 27). Contrary to popular belief, digital proficiency was not reported to be a significant barrier to implementation, as it was felt that a supporting mechanism can be created (refer to quote 28).

Lack of acts, laws, malpractice liability and credentialing issues were also some of the reported challenges to tele-audiology service implementation (P11). In certain countries where state licensing was essential, unique licensing policies to deliver telepractice services across states was required (refer to quote 29). Some of these legislative policies gained popularity only after the COVID-19 pandemic and project implementers felt that these changes need to be made permanent (refer to quote 30).

Implementation cost:

For peer review only - http://bmjopen.bmj.com/site/about/guidelines.xhtml

Factors related to cost of setting-up or cost associated with continued tele-audiology services could be mapped to *implementation cost* which is an early to late-stage implementation outcome. Higher initial capital investment to set up tele-audiology service delivery and the need for specialised equipment to support such practice was reported as a barrier to implementation (P6, P12, P15, and P20) and was only possible with adequate funding (refer to quote 31). When there was a pause or temporary issue with the release of funds, it adversely affected service delivery (refer to quote 32). Even though tele-audiology has gained popularity and preference in the pandemic, it was not adequately supported financially by organisations in the past for routine service delivery (refer to quote 33). Multiple sources of funding including scientific grants, organisational funding, and charitable donations were required to ensure continuity of service (P19, P20) (refer to quote 34). On some occasions, lack of insurance reimbursement for tele-audiology services was a barrier (P11) (refer to quote 35).

When long-term cost outcomes or cost benefits of tele-audiology were evaluated, they found it to be a beneficial alternative from a societal perspective (P10, P15). Considerable efforts were taken to establish and self-sustain billable tele-audiology service delivery and these were very few (P21) (refer to quote 36).

Sustainability:

Factors reporting the maintenance of tele-audiology service delivery were mapped to the late-stage implementation outcome of *sustainability* and these were minimal. Out of the identified projects in tele-audiology, 14 projects (P1, P2, P3, P4, P5, P7, P9, P10, P11, P13, P14, P18, P19 and P20) reported service delivery to be ongoing or routine. The rest, even though long-term, were still in the research phase. Tele-audiology services were implemented in a more sustained manner in hearing screening programs for infants. For example, follow-up diagnostic evaluations were conducted using telepractice in the USA and Canada (P2, P3, P4, P5), ear and hearing screening among school children was conducted in the USA (P1) and diagnostic audiological with otorhinolaryngological evaluation for adults was conducted in the USA and in India (P7, P9, P10). The Veterans hospitals in the USA have implemented remote hearing-aid fitting and programming as well as cochlear implant fitting and mapping as a routine service (P11, P13, and P20). Another effort is the

For peer review only - http://bmjopen.bmj.com/site/about/guidelines.xhtml

National Network of Teleaudiology in Poland for cochlear implant switch-on and mapping (P14). Aural rehabilitation has also been implemented in a sustained manner in both the USA and Australia (P18, P19). Systematic pre-implementation feasibility, test runs and validation of functional requirements were useful components of sustainable implementation (P9, P5, and P18). Continued support from the organisation is crucial for sustainable telepractice service delivery. The lack of interlinking support structures across administrative health authorities was reported to be detrimental to sustainability (P5).

For peer review only

For peer review only - http://bmjopen.bmj.com/site/about/guidelines.xhtml

DISCUSSION

Through this study we identified long-term and sustained tele-audiology services and the factors that influenced their sustainability. The reported factors were then mapped to the IOF (1) to understand the gaps that must be addressed for better translation of research evidence to sustained implementation.

In general, we found that tele-audiology was predominantly implemented in remote/rural locations to overcome practical challenges associated with receiving in-person services. The most common application of tele-audiology was for diagnostic testing with ABR or OAE in infant hearing screening programs. This was done to reduce the loss to follow-up especially when hearing screening was conducted in primary health care clinics or rural communities (33,34,36,38). Aural re/habilitation for hearing loss was the next most common application, where only simple video-conferencing software was required. Remote hearing aid fitting and programming was minimally explored, possibly due to the limited availability of supportive features in products of a wider price range (63). There is a definite preference for using synchronous methods for conducting diagnostic tests or fitting hearing devices when audiologists' real-time judgement is more crucial (64). Whenever screening was done or images could be captured (like video-otoscopy or tympanometry), asynchronous methods were used. In such tests, real-time assessments by the professional were not considered to be critical for test completion. It was possible to provide telepractice services, such as aural re/habilitation, at homes using simple real-time videoconferencing which did not mandate specialised skills (65). In these cases, patient preparation was necessary and the parent/caregiver acted as a facilitator.

While the use of implementation research and/or outcome frameworks for translating evidence-based practice to clinical service delivery is gaining momentum, it is still underutilized in tele-audiology service implementations. None of the studies reported the use of an underlying implementation theory/framework/model and implementation outcomes were not studied systematically. More than half of the studies assessed process evaluation objectives, which were mostly related to patient satisfaction, but only a smaller subset reported its outcomes.

For peer review only

Only a few studies reported economic evaluations. We identified two projects (P1, P7) where tele-audiology services were provided as a part of comprehensive multi-specialty services. Impact evaluations of such implementations are likely to inform the value of capitalising on common infrastructure and shared human resources. The core component of the intervention (tele-audiology service) to which fidelity is expected and those aspects that may be adapted must be systematically reported to aid implementation. But, none of the studies reported the use of fidelity or adaption measures.

The factors reported to influence the sustainability of these services were mapped to the constructs of IOF. These factors were predominantly related to early-stage implementation outcomes of *feasibility* and early to late-stage implementation outcomes of *acceptability*. Limited factors could be mapped to the implementation outcomes of *adoption (early-stage), implementation cost (early to late stage)* and *sustainability (late stage)*.

With reference to feasibility, internet access is a basic prerequisite for tele-audiology. The internet's speed and bandwidth affect various aspects of tele-audiology implementation, including test selection, technique and model of service delivery (66). The use of broadband connectivity and undersea cables was reported to be useful by implementers, yet there seems to be a continued challenge in maintaining robust connections for the purpose of tele-audiological tests in remote/rural locations (67). In addition, legislative policies and guidelines influenced feasibility of implementation considerably. Several countries introduced technical guidelines and changes in legislative policies for telepractice only during the COVID-19 pandemic (68). However, supportive mechanisms such as integrated electronic health records are still not widely available at a national level (69).

Acceptability and perceived demand for tele-audiology services by both the patients/ beneficiaries and the providers seems essential, as they are primary stakeholders involved. The findings of the current study indicate that better administrative convergence is required between health authorities for implementation of

For peer review only - http://bmjopen.bmj.com/site/about/guidelines.xhtml

tele-audiology services. The provider's willingness and comfort in adapting their services using technology is crucial for sustainability of tele-audiology service delivery (70).

For adoption of tele-audiology within organisational structures, supportive organisational policies that recognise it as an important component of service delivery is required (71). This will in-turn impact various organisational aspects including the availability of human resources, collaborations and effective planning. Tele-audiology is unlikely to be sustained without a clear plan that defines roles and responsibilities (72).

With reference to implementation cost, our findings suggests that even though tele-audiology services were initiated through research grants, several of them were able to integrate these services into public-funded programs. This suggests that sustained implementation occurred when the need and usefulness of tele-audiology were accepted by public sector policy-makers. If research projects include economic evaluation, then this can in turn inform the allocation of funds by organisations to support sustained implementation (73).

Factors related to appropriateness, *fidelity* and *penetration,* which are early, mid and late-stage implementation outcomes respectively, were not reported. Information regarding the context influencing implementation, the outcomes of the implementation strategy, process evaluation objectives, process outcomes, economic evaluation, specific measures to assess adaptation and scalability were not described. When appropriate implementation research and outcome frameworks are used, the stages or processes involved in achieving sustainability are less likely to be missed.

The strength of the current study lies in the representation of tele-audiology implementation efforts in a wide range of countries that include low-middle income (India and South Africa) and high-income (USA, Canada, Australia and Poland) nations. We found that the factors influencing the sustainability of tele-audiology service delivery reported in both High-Income Countries (HICs) and Low-Middle Income Countries (LMICs) were similar, except with respect to insurance coverage for tele-practice, and the

For peer review only

availability of electronic or digital health records which emerged in the context of HICs and not LMICs. Therefore, we believe that the findings of this study will be useful to project implementers from different regions.

For peer review only

List of abbreviations:

Preferred Reporting Items for Systematic Reviews and Meta-Analyses - PRISMA

United States of America - USA

Distortion Product Otoacoustic Emissions - DPOAEs

Auditory Brainstem Response - ABR

Auditory Steady-State Response - ASSR

Memorandum of Understanding - MOU

Telehealth Service Agreement - TSA

Standards for Reporting Implementation Studies - StaRI

High-Income Countries - HICs

Low-Middle Income Countries - LMICs

For peer review only

ACKNOWLEDGMENTS:

Acknowledgements: We would like to thank all the researchers who consented to participate in the interviews sharing their valuable time for furthering the understanding in this area of research.

Competing interests: The Authors declare that there is no competing interest.

Funding: The authors disclosed receipt of the following financial support for the research, authorship, and/or publication of this article: This work was supported by the DBT/Wellcome Trust India Alliance Fellowship/Grant [IA/CPHI/19/1/504614] awarded to Vidya Ramkumar. The funders had no role in study design, data collection and analysis, decision to publish, or preparation of the manuscript.

Ethical Approval: Ethical clearance was obtained from the Institutional Ethics Committee (Reference Number: CSP/20/NOV/87/191) of Sri Ramachandra Institute of Higher Education and Research (DU) for this study.

Informed consent from participants: Informed written consent was obtained from all interview participants prior to the interviews.

Consent to publish statement: 'No objections' to publish the quotes from interviews was obtained from all participants.

Conflicts of Interest: The Authors declare that there is no conflict of interest.

Author Contribution: VR conceptualized the research topic, both VR and VS were involved in data curation, formal analysis, investigation, finalizing methodology, original draft preparation and reviewing & editing. VR was also integral in supervision, visualisation and acquisition of funding, resources and software. Both authors read and approved the final manuscript. SK was involved in methodology for coding, thematic analysis, and manuscript review and editing.

DATA AVAILABILITY STATEMENT:

For peer review only - http://bmjopen.bmj.com/site/about/guidelines.xhtml

The data that support the findings of this study are available on request from the corresponding author, VR. The data are not publicly available as the data underlying this study consists of transcripts of the qualitative interviews that identify the participants and cannot be sufficiently de-identified. To ensure confidentiality and data protection, we have only included relevant excerpts from the interviews with 'no objections' from the participants. We will only provide the transcripts to researchers/those that provide a proposal of how the data will be used by them. The corresponding author will ensure to remove identifiers from the particular data set and obtain 'no objections' from the participants before sharing the same.

For peer review only

For peer review only - http://bmjopen.bmj.com/site/about/guidelines.xhtml

References:

1. Proctor E, Silmere H, Raghavan R, Hovmand P, Aarons G, Bunger A, et al. Outcomes for implementation research: Conceptual distinctions, measurement challenges, and research agenda. Administration and Policy in Mental Health and Mental Health Services Research. 2011 Mar;38(2):65–76.
2. Waterworth CJ, Marella M, O'Donovan J, Bright T, Dowell R, Bhutta MF. Barriers to access to ear and hearing care services in low- and middle- income countries: A scoping review. https://doi.org/101080/1744169220222053734 [Internet]. 2022 Mar 23 [cited 2022 Jun 13];1–25. Available from: https://www.tandfonline.com/doi/abs/10.1080/17441692.2022.2053734
3. Mahmood M, Bhutta F, Bhutta MF. Models of service delivery for ear and hearing care in remote or resource-constrained environments. J Laryngol Otol [Internet]. 2019 Jan 1 [cited 2022 Jun 13];133(1):39–48. Available from: https://www.cambridge.org/core/journals/journal-of-laryngology-and-otology/article/models-of-service-delivery-for-ear-and-hearing-care-in-remote-or-resourceconstrained-environments/A79DDE056CDDEA54B09DCB561AE9BBC9
4. Botasso M, Sanches SGG, Bento RF, Samelli AG. Teleaudiometry as a screening method in school children. Clinics. 2015;70(4):283–8.
5. Monica SD, Ramkumar V, Krumm M, Raman N, Nagarajan R, Venkatesh L. School entry level tele-hearing screening in a town in South India – Lessons learnt. Int J Pediatr Otorhinolaryngol [Internet]. 2017;92:130–5. Available from: http://dx.doi.org/10.1016/j.ijporl.2016.11.021
6. Biagio L, Swanepoel DW, Adeyemo A, Hall JW, Vinck B. Asynchronous video-otoscopy with a telehealth facilitator. Telemedicine and e-Health. 2013;19(4):252–8.
7. Swanepoel DW, Koekemoer D, Clark J. Intercontinental hearing assessment - a study in tele-audiology. J Telemed Telecare. 2010;16(5):248–52.
8. Visagie A, Swanepoel DW, Eikelboom RH. Accuracy of remote hearing assessment in a rural community. Telemedicine and e-Health. 2015;21(11):930–7.
9. Cullington H, Kitterick P, Debold L, Weal M, Clarke N, Newberry E, et al. Personalised long-term follow-up of cochlear implant patients using remote care, compared with those on the standard care pathway: Study protocol for a feasibility randomised controlled trial. BMJ Open. 2016;6(5):1–9.
10. Beukes EW, Vlaescu G, Manchaiah V, Baguley DM, Allen PM, Kaldo V, et al. Development and technical functionality of an Internet-based intervention for tinnitus in the UK. Internet Interv [Internet]. 2016;6(August):6–15. Available from: http://dx.doi.org/10.1016/j.invent.2016.08.002
11. Krumm M. Audiology telemedicine. J Telemed Telecare. 2007;13(5):224–9.
12. Tao KFM, Brennan-Jones CG, Capobianco-Fava DM, Jayakody DMP, Friedland PL, Swanepoel DW, et al. Teleaudiology services for rehabilitation with hearing aids in adults: A systematic review. Journal of Speech, Language, and Hearing Research. 2018;61(7):1831–49.
13. Swanepoel DW, Clark JL, Koekemoer D, Hall JW, Krumm M, Ferrari D V., et al. Telehealth in audiology: The need and potential to reach underserved communities. Int J Audiol. 2010;49(3):195–202.
14. Coco L, Davidson A, Marrone N. The role of patient-site facilitators in teleaudiology: A scoping review. Am J Audiol. 2020;29(3 Special Issue):661–75.
15. Muñoz K, Nagaraj NK, Nichols N, Muñoz K, Nagaraj NK, Applied NN. Applied tele-audiology research in clinical practice during the past decade : a scoping review. Int J Audiol [Internet]. 2020;0(0):1–9. Available from: https://doi.org/10.1080/14992027.2020.1817994
16. Angral S, Varshney S, Aanand P, Raj R. Tele Otology in India: Last 10 Years—A Scopic Review. Indian Journal of Otolaryngology and Head and Neck Surgery [Internet]. 2021; Available from: https://doi.org/10.1007/s12070-021-02546-4
17. Glasgow RE, Harden SM, Gaglio B, Rabin B, Smith ML, Porter GC, et al. RE-AIM planning and evaluation framework: Adapting to new science and practice with a 20-year review. Vol. 7, Frontiers in Public Health. Frontiers Media S.A.; 2019.
18. Chambers DA, Glasgow RE, Stange KC. The dynamic sustainability framework: Addressing the paradox of sustainment amid ongoing change. Implementation Science. 2013 Oct 2;8(1).

For peer review only - http://bmjopen.bmj.com/site/about/guidelines.xhtml

19. Glasgow RE, Vinson C, Chambers D, Khoury MJ, Kaplan RM, Hunter C. National Institutes of Health Approaches to Dissemination and Implementation Science: Current and Future Directions. Am J Public Health. 2012;102(7):1274–81.
20. Damschroder LJ, Aron DC, Keith RE, Kirsh SR, Alexander JA, Lowery JC. Fostering implementation of health services research findings into practice: A consolidated framework for advancing implementation science. Implementation Science. 2009;4(1):1–15.
21. Campbell J, Theodoros D, Hartley N, Russell T, Gillespie N. Implementation factors are neglected in research investigating telehealth delivery of allied health services to rural children: A scoping review. J Telemed Telecare. 2019;
22. Tricco AC, Lillie E, Zarin W, O'Brien KK, Colquhoun H, Levac D, et al. PRISMA extension for scoping reviews (PRISMA-ScR): Checklist and explanation. Ann Intern Med. 2018;169(7):467–73.
23. Ouzzani M, Hammady H, Fedorowicz Z, Elmagarmid A. Rayyan---a web and mobile app for systematic reviews. Syst Rev. 2016;5(1):210.
24. Pinnock H, Barwick M, Carpenter CR, Eldridge S, Grandes G, Griffiths CJ, et al. Standards for Reporting Implementation Studies (StaRI) Statement. BMJ (Online). 2017;356(March):1–9.
25. Tong A, Sainsbury P, Craig J. Consolidated criteria for reporting qualitative research (COREQ): A 32-item checklist for interviews and focus groups. International Journal for Quality in Health Care. 2007;19(6):349–57.
26. Clarke V, Braun V. Teaching thematic analysis: Overcoming challenges and developing strategies for effective learning. The SAGE Encyclopedia of Qualitative Research Methods. 2012;
27. Braun V, Clarke V. Using thematic analysis in psychology. Qual Res Psychol. 2006;3(2):77–101.
28. Saunders B, Sim J, Kingstone T, Baker S, Waterfield J, Bartlam B, et al. Saturation in qualitative research: exploring its conceptualization and operationalization. Qual Quant. 2018;52(4):1893–907.
29. Swain J. A Hybrid Approach to Thematic Analysis in Qualitative Research: Using a Practical Example. A Hybrid Approach to Thematic Analysis in Qualitative Research: Using a Practical Example. 2018;(January 2018).
30. Emmett SD, Robler SK, Wang NY, Labrique A, Gallo JJ, Hofstetter P. Hearing Norton Sound: A community randomised trial protocol to address childhood hearing loss in rural Alaska. BMJ Open. 2019;9(1).
31. Emmett SD, Robler SK, Gallo JJ, Wang NY, Labrique A, Hofstetter P. Hearing Norton Sound: Mixed methods protocol of a community randomised trial to address childhood hearing loss in rural Alaska. BMJ Open. 2019;9(1):1–10.
32. Robler SK, Inglis SM, Gallo JJ, Parnell HE, Ivanoff P, Ryan S, et al. Hearing Norton Sound: community involvement in the design of a mixed methods community randomized trial in 15 Alaska Native communities. Res Involv Engagem. 2020;6(1):1–11.
33. Dharmar M, Simon A, Sadorra C, Friedland G, Sherwood J, Morrow H, et al. Reducing loss to follow-up with Tele-audiology diagnostic evaluations. Telemedicine Journal and e-Health. 2016;22(2):1–6.
34. Stuart A. Infant Diagnostic Evaluation Via Teleaudiology Following Newborn Screening In Eastern North Carolina. J Early Hear Detect Interv. 2016;1(2):63–71.
35. Campbell W, Hyde M. eEHDI : Functions and Challenges. A Sound Foundation Through Early Amplification: Proceedings of the Fourth International Conference. 2010;115–30, 273p.
36. Hatton JL, Rowlandson J, Beers A, Small S. Telehealth-enabled auditory brainstem response testing for infants living in rural communities: the British Columbia Early Hearing Program experience. Int J Audiol [Internet]. 2019;58(7):381–92. Available from: https://doi.org/10.1080/14992027.2019.1584681
37. Ramkumar V, Nagarajan R, Shankarnarayan VC, Kumaravelu S, Hall JW. Implementation and evaluation of a rural community-based pediatric hearing screening program integrating in-person and tele-diagnostic auditory brainstem response (ABR). BMC Health Serv Res. 2019;19(1):1–13.
38. Ramkumar V, John KR, Selvakumar K, Vanaja CS, Nagarajan R, Hall JW. Cost and outcome of a community-based paediatric hearing screening programme in rural India with application of tele-audiology for follow-up diagnostic hearing assessment. Int J Audiol [Internet]. 2018;57(6):407–14. Available from: https://doi.org/10.1080/14992027.2018.1442592
39. Hofstetter PJ, Kokesh J, Ferguson AS, Hood LJ. The impact of telehealth on wait time for ENT specialty care. Telemed J E Health. 2010;16(5):551–6.

For peer review only - http://bmjopen.bmj.com/site/about/guidelines.xhtml

40. Kokesh J, Ferguson AS, Patricoski C. The alaska experience using store-and-forward telemedicine for ENT care in alaska. Otolaryngol Clin North Am [Internet]. 2011;44(6):1359–74. Available from: http://dx.doi.org/10.1016/j.otc.2011.08.010
41. Smith AC, Armfield NR, Wu WI, Brown CA, Perry C. A mobile telemedicine-enabled ear screening service for Indigenous children in Queensland: Activity and outcomes in the first three years. J Telemed Telecare. 2012;18(8):485–9.
42. Smith AC, Armfield NR, Wu WI, Brown CA, Mickan B, Perry C. Changes in paediatric hospital ENT service utilisation following the implementation of a mobile, indigenous health screening service. J Telemed Telecare. 2013;19(7):397–400.
43. Smith AC, Brown C, Bradford N, Caffery LJ, Perry C, Armfield NR. Monitoring ear health through a telemedicine-supported health screening service in Queensland. J Telemed Telecare. 2015;21(8):427–30.
44. Gupta N, Baghotia KS, Rabha M, Sachdeva S, Sahai G, Bhatnagar K, et al. Comprehensive Community Screening of Otological Patients by Trained Technicians Using a Telemedicine Device: An Efficient and Cost-Effective Way to Triage Patients With Ear Diseases. Ear Nose Throat J. 2020;0–5.
45. Ravi P, Ramkumar V, Rajendran A, Saravanam PK, Balasubramaniyan S, Nagarajan R. Tele-audiological surveillance of middle ear status among individuals with cleft lip and/or palate in rural South India. J Am Acad Audiol. 2020;31(3):185–94.
46. Dennis KC, Gladden CF, Noe CM. Telepractice in the Department of Veterans Affairs. Hearing Review [Internet]. 2012;19(11):44–50. Available from: http://search.ebscohost.com/login.aspx?direct=true&db=ccm&AN=2011707530&site=ehost-live&scope=cite
47. Novak R, Cantu AG, Zappler A, Coco L, Champlin C. The Future of Healthcare Delivery : IPE / IPP Audiology and Nursing Student / Faculty Collaboration to Deliver Hearing Aids to Vulnerable Adults via Telehealth. 2016;1(1).
48. Luryi AL, Tower JI, Preston J, Burkland A, Trueheart CE, Hildrew DM. Cochlear Implant Mapping Through Telemedicine - A Feasibility Study. Otology and Neurotology. 2020;41(3):e330–3.
49. Skarżyński PH, Świerniak W, Bruski Ł, Ludwikowski M, Skarżyński H. Comprehensive approach to the National Network of Teleaudiology in World Hearing Center in Kajetany, Poland. Finnish Journal of eHealth and eWelfare. 2018;10(4):354–63.
50. Skarżyński PH, Swierniak W, Ludwikowski M, Bruski Ł. Telefitting Betwen Kajetany , Poland and Odessa , Ukraine for Cochlear Implants. 2019;1–6.
51. Houston KT. TeleIntervention: Improving Service Delivery to Young Children With Hearing Loss and Their Families Through Telepractice. Perspect Hear Hear Disord Child. 2011;21(2):66–72.
52. Houston KT, Stredler-Brown A. A model of early intervention. 2012. p. 283–96.
53. Blaiser KM, Edwards M, Behl D, Munoz KF. Telepractice Services at Sound Beginnings at Utah State University [Internet]. Vol. 112, The Volta Review. 2012. p. 365–72. Available from: https://login.iris.etsu.edu:3443/login?url=http://search.proquest.com/docview/1430172127?accountid=10771
54. Broekelmann C. ihear® Internet Therapy Program - A Program by St. Joseph Institute for the Deaf. 2012.
55. Lalios AP. ConnectHear TeleIntervention Program. 2012. p. 357–64.
56. Galvan C, Case E, Todd Houston K. Listening and Learning: Using Telepractice to Serve Children and Adults with Hearing Loss. Perspectives on Telepractice. 2014;4(1):11–22.
57. McCarthy M. RIDBC Teleschool: A Hub of Expertise. 2012.
58. McCarthy M. Using technology to support children with sensory disability in remote areas: The RIDBC teleschool model. Telecommunications Journal of Australia. 2011;61(2):1–7.
59. McCarthy M, Muñoz K, White KR. Teleintervention for infants and young children who are deaf or hard-of-hearing. Pediatrics. 2010;126(SUPPL. 1).
60. Gladden C, Beck L, Chandler D. Tele-audiology: Expanding access to hearing care and enhancing patient connectivity. J Am Acad Audiol. 2015;26(9):792–9.
61. Ratanjee-Vanmali H, Swanepoel DW, Laplante-Lévesque A. Patient uptake, experience, and satisfaction using web-based and face-to-face hearing health services: Process evaluation study. J Med Internet Res. 2020;22(3).

For peer review only - http://bmjopen.bmj.com/site/about/guidelines.xhtml

62. Ramkumar V, Vanaja CS, Hall JW, Selvakumar K, Nagarajan R. Validation of DPOAE screening conducted by village health workers in a rural community with real-time click evoked tele-auditory brainstem response. Int J Audiol [Internet]. 2018;57(5):370–5. Available from: https://doi.org/10.1080/14992027.2018.1425001
63. Tao KFM, Brennan-Jones CG, Capobianco-Fava DM, Jayakody DMP, Friedland PL, Swanepoel DW, et al. Teleaudiology services for rehabilitation with hearing aids in adults: A systematic review. Journal of Speech, Language, and Hearing Research. 2018;61(7):1831–49.
64. Krumm M. A review of contemporary tele-audiology literature. J Hear Sci. 2016;6(3):9–21.
65. Banbury A, Nancarrow S, Dart J, Gray L, Parkinson L. Telehealth interventions delivering home-based support group videoconferencing: Systematic review. J Med Internet Res. 2018;20(2).
66. Behl DD, Kahn G. Provider perspectives on telepractice for serving families of children who are deaf or hard of hearing. Int J Telerehabil. 2015;7(1):1–11.
67. D'Onofrio KL, Zeng FG. Tele-Audiology: Current State and Future Directions. Front Digit Health. 2022;3(January):1–11.
68. Hao Y, Zhang S, Conner A, Lee NY. The evolution of telepractice use during the covid-19 pandemic: Perspectives of pediatric speech-language pathologists. Int J Environ Res Public Health. 2021;18(22).
69. Kumar M, Mostafa J. Research evidence on strategies enabling integration of electronic health records in the health care systems of low- and middle-income countries: A literature review. International Journal of Health Planning and Management. 2019;34(2):e1016–25.
70. Singh G, Pichora-Fuller MK, Malkowski M, Boretzki M, Launer S. A survey of the attitudes of practitioners toward teleaudiology. Int J Audiol. 2014;53(12):850–60.
71. Cason J, Cohn ER. Telepractice: An Overview and Best Practices. Perspect Augment altern commun. 2014;23(1):4–17.
72. Thomas EE, Taylor ML, Ward EC, Hwang R, Cook R, Ross JA, et al. Beyond forced telehealth adoption: A framework to sustain telehealth among allied health services. J Telemed Telecare. 2022;
73. Turner HC, Archer RA, Downey LE, Isaranuwatchai W, Chalkidou K, Jit M, et al. An Introduction to the Main Types of Economic Evaluations Used for Informing Priority Setting and Resource Allocation in Healthcare : Key Features , Uses , and Limitations. 2021;9(August):1–17.

For peer review only

For peer review only - http://bmjopen.bmj.com/site/about/guidelines.xhtml

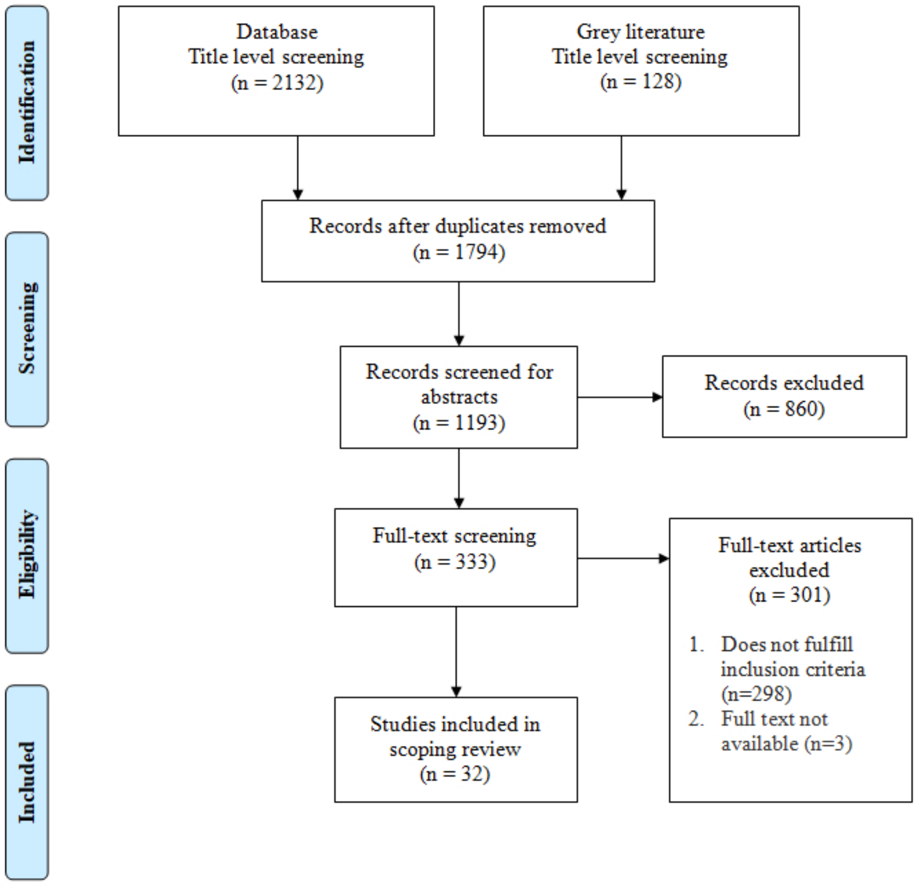

Figure 1: PRISMA flow diagram representing the study's search process

256x236mm (72 x 72 DPI)

Preferred Reporting Items for Systematic reviews and Meta-Analyses extension for Scoping Reviews (PRISMA-ScR) Checklist

SECTION	ITEM	PRISMA-ScR CHECKLIST ITEM	REPORTED ON PAGE #
**TITLE**			
Title	1	Identify the report as a scoping review.	
**ABSTRACT**			
Structured summary	2	Provide a structured summary that includes (as applicable): background, objectives, eligibility criteria, sources of evidence, charting methods, results, and conclusions that relate to the review questions and objectives.	
**INTRODUCTION**			
Rationale	3	Describe the rationale for the review in the context of what is already known. Explain why the review questions/objectives lend themselves to a scoping review approach.	
Objectives	4	Provide an explicit statement of the questions and objectives being addressed with reference to their key elements (e.g., population or participants, concepts, and context) or other relevant key elements used to conceptualize the review questions and/or objectives.	
**METHODS**			
Protocol and registration	5	Indicate whether a review protocol exists; state if and where it can be accessed (e.g., a Web address); and if available, provide registration information, including the registration number.	
Eligibility criteria	6	Specify characteristics of the sources of evidence used as eligibility criteria (e.g., years considered, language, and publication status), and provide a rationale.	
Information sources*	7	Describe all information sources in the search (e.g., databases with dates of coverage and contact with authors to identify additional sources), as well as the date the most recent search was executed.	
Search	8	Present the full electronic search strategy for at least 1 database, including any limits used, such that it could be repeated.	
Selection of sources of evidence†	9	State the process for selecting sources of evidence (i.e., screening and eligibility) included in the scoping review.	
Data charting process‡	10	Describe the methods of charting data from the included sources of evidence (e.g., calibrated forms or forms that have been tested by the team before their use, and whether data charting was done independently or in duplicate) and any processes for obtaining and confirming data from investigators.	
Data items	11	List and define all variables for which data were sought and any assumptions and simplifications made.	
Critical appraisal of individual sources of evidence§	12	If done, provide a rationale for conducting a critical appraisal of included sources of evidence; describe the methods used and how this information was used in any data synthesis (if appropriate).	
Synthesis of results	13	Describe the methods of handling and summarizing the data that were charted.	

St. Michael's
Inspired Care. Inspiring Science.

For peer review only - http://bmjopen.bmj.com/site/about/guidelines.xhtml

SECTION	ITEM	PRISMA-ScR CHECKLIST ITEM	REPORTED ON PAGE #
**RESULTS**			
Selection of sources of evidence	14	Give numbers of sources of evidence screened, assessed for eligibility, and included in the review, with reasons for exclusions at each stage, ideally using a flow diagram.	
Characteristics of sources of evidence	15	For each source of evidence, present characteristics for which data were charted and provide the citations.	
Critical appraisal within sources of evidence	16	If done, present data on critical appraisal of included sources of evidence (see item 12).	
Results of individual sources of evidence	17	For each included source of evidence, present the relevant data that were charted that relate to the review questions and objectives.	
Synthesis of results	18	Summarize and/or present the charting results as they relate to the review questions and objectives.	
**DISCUSSION**			
Summary of evidence	19	Summarize the main results (including an overview of concepts, themes, and types of evidence available), link to the review questions and objectives, and consider the relevance to key groups.	
Limitations	20	Discuss the limitations of the scoping review process.	
Conclusions	21	Provide a general interpretation of the results with respect to the review questions and objectives, as well as potential implications and/or next steps.	
**FUNDING**			
Funding	22	Describe sources of funding for the included sources of evidence, as well as sources of funding for the scoping review. Describe the role of the funders of the scoping review.	

JBI = Joanna Briggs Institute; PRISMA-ScR = Preferred Reporting Items for Systematic reviews and Meta-Analyses extension for Scoping Reviews.

* Where *sources of evidence* (see second footnote) are compiled from, such as bibliographic databases, social media platforms, and Web sites.

† A more inclusive/heterogeneous term used to account for the different types of evidence or data sources (e.g., quantitative and/or qualitative research, expert opinion, and policy documents) that may be eligible in a scoping review as opposed to only studies. This is not to be confused with *information sources* (see first footnote).

‡ The frameworks by Arksey and O'Malley (6) and Levac and colleagues (7) and the JBI guidance (4, 5) refer to the process of data extraction in a scoping review as data charting.

§ The process of systematically examining research evidence to assess its validity, results, and relevance before using it to inform a decision. This term is used for items 12 and 19 instead of "risk of bias" (which is more applicable to systematic reviews of interventions) to include and acknowledge the various sources of evidence that may be used in a scoping review (e.g., quantitative and/or qualitative research, expert opinion, and policy document).

From: Tricco AC, Lillie E, Zarin W, O'Brien KK, Colquhoun H, Levac D, et al. PRISMA Extension for Scoping Reviews (PRISMAScR): Checklist and Explanation. Ann Intern Med. 2018;169:467–473. doi: 10.7326/M18-0850.

For peer review only - http://bmjopen.bmj.com/site/about/guidelines.xhtml

Supplemental file 2
Search terms used for the article selection

Telepractice Terms	Audiology	Age groups
Teleaudiology<br>Telepractice<br>Telehealth<br>Telemedicine<br>Telerehabilitation<br>Teletherapy<br>e-health<br>m-health<br>Internet based<br>Computer based<br>Web based<br>Tablet based<br>Mobile based	Audiology<br>Aural rehabilitation<br>Auditory verbal therapy<br>Ear care<br>Hearing care<br>Hearing health<br>Hearing loss	Pediatric<br>Paediatric<br>Infant<br>Children<br>Teenage<br>Adolescent<br>Adults<br>Geriatric<br>Old age
**Service type**	**Service Mode**	**Implementation terms**
Evaluation<br>Diagnostics<br>Assessment<br>Treatment<br>Management<br>Rehabilitation<br>Therapy	Synchronous<br>Asynchronous<br>Hybrid<br>Store and forward<br>Videoconference<br>Videoconferencing<br>Real time	Implementation<br>Adoption<br>Sustainability<br>Acceptance<br>Readiness<br>Perspectives<br>Perceptions
**Hearing disorders**	**Area and settings**	
Auditory Brainstem Response<br>Audiometry<br>Tympanometry<br>Auditory steady state response<br>Video-otoscopy<br>Cochlear Implant<br>Hearing aids<br>Hearing aid trial<br>Hearing aid fitting<br>Cochlear implant mapping<br>Tinnitus<br>Vestibular<br>Middle ear diseases	Rural<br>Suburban<br>Urban<br>Village<br>Hamlet<br>Country<br>Community based<br>School based<br>School district<br>Remote care<br>Home based<br>Universities<br>Clinic<br>Pre-school<br>Day care centres<br>Primary schools	

For peer review only

Supplemental file 3
Initial Decision Profile

Study code:
Full Citation:

1. **Is it an implementation study?**

   ❑ Yes ❑ No ❑ Maybe

2. **Has it been published between January, 2010 - April, 2021?**

   ❑ Yes ❑ No ❑ Maybe

3. **Did the project span across a period of 2 or more years?**

   ❑ Yes ❑ No ❑ Maybe

4. **Is it in the field of Tele-audiology?**

   ❑ Yes ❑ No ❑ Maybe

5. **Does it involve screening, diagnostic and rehabilitative services?**

   ❑ Yes ❑ No ❑ Maybe

Notes:

❑ **ELIGIBLE** ❑ **NOT ELIGIBLE**

For peer review only

Appendix 7 - StaRI Checklist

Checklist item	Item number	Emmett et al. (2019)		Emmett et al. (2019)		Robler et al. (2020)		Dharmar et al. (2016)	
		Implementation strategy	Intervention	Implementation strategy	Intervention	Implementation strategy	Intervention	Implementation strategy	Intervention
Title	1	?		?		?		X	
Abstract	2	?		?		?		X	
Introduction	3	✓		✓		✓		✓	
Rationale	4	X	✓	X	✓	?	✓	X	✓
Aims and objectives	5	✓		✓		✓		?	
Design	6	?		?		?		?	
Context	7	✓		✓		✓		?	
Targeted 'sites'	8	✓	✓	✓	✓	✓	✓	?	✓
Description	9	✓	✓	✓	✓	✓	✓	?	✓
Sub-groups	10	N/A		✓		N/A		N/A	
Outcomes	11	✓	✓	✓	✓	✓	✓	?	✓
Process evaluation	12	✓		✓		✓		✓	
Economic evaluation	13	X	X	X	X	X	X	X	X
Sample size	14	✓		✓		✓		✓	
Analysis	15	✓		✓		✓		✓	
Sub-group analyses	16	N/A		✓		N/A		N/A	
Characteristics	17	N/A		N/A		✓	N/A	?	✓
Outcomes	18	N/A		N/A		✓	N/A	?	✓
Process outcomes	19	N/A		N/A		X		X	
Economic evaluation	20	N/A		N/A		X	X	X	X
Sub-group analyses	21	N/A		N/A		N/A		✓	
Fidelity/ adaptation	22	N/A		N/A		✓	N/A	X	X
Contextual changes	23	N/A		N/A		✓		X	
Harms	24	N/A		N/A		N/A		N/A	
Structured discussion	25	N/A		N/A		✓		✓	
Implications	26	N/A		N/A		✓	N/A	X	X
Statements	27	✓		✓		✓		✓	

Appendix 7 - StaRI Checklist

<table>
<tr><th rowspan="2">Checklist item</th><th rowspan="2">Item number</th><th colspan="2">Stuart (2016)</th><th colspan="2">W. Campbell and Hyde (2010)</th><th colspan="2">Hatton et al. (2019a)</th><th colspan="2">Ramkumar et al. (2018a)</th></tr>
<tr><th>Implementation strategy</th><th>Intervention</th><th>Implementation strategy</th><th>Intervention</th><th>Implementation strategy</th><th>Intervention</th><th>Implementation strategy</th><th>Intervention</th></tr>
<tr><td>Title</td><td>1</td><td colspan="2">X</td><td colspan="2">X</td><td colspan="2">X</td><td colspan="2">X</td></tr>
<tr><td>Abstract</td><td>2</td><td colspan="2">X</td><td colspan="2">X</td><td colspan="2">✓</td><td colspan="2">✓</td></tr>
<tr><td>Introduction</td><td>3</td><td colspan="2">✓</td><td colspan="2">✓</td><td colspan="2">✓</td><td colspan="2">✓</td></tr>
<tr><td>Rationale</td><td>4</td><td>X</td><td>✓</td><td>X</td><td>✓</td><td>X</td><td>✓</td><td>X</td><td>✓</td></tr>
<tr><td>Aims and objectives</td><td>5</td><td colspan="2">✓</td><td colspan="2">?</td><td colspan="2">✓</td><td colspan="2">N/A</td></tr>
<tr><td>Design</td><td>6</td><td colspan="2">?</td><td colspan="2">?</td><td colspan="2">?</td><td colspan="2">?</td></tr>
<tr><td>Context</td><td>7</td><td colspan="2">✓</td><td colspan="2">✓</td><td colspan="2">✓</td><td colspan="2">X</td></tr>
<tr><td>Targeted ‘sites’</td><td>8</td><td>✓</td><td>✓</td><td>✓</td><td>✓</td><td>✓</td><td>✓</td><td>✓</td><td>✓</td></tr>
<tr><td>Description</td><td>9</td><td>✓</td><td>✓</td><td>✓</td><td>✓</td><td>✓</td><td>✓</td><td>✓</td><td>✓</td></tr>
<tr><td>Sub-groups</td><td>10</td><td colspan="2">N/A</td><td colspan="2">N/A</td><td colspan="2">N/A</td><td colspan="2">N/A</td></tr>
<tr><td>Outcomes</td><td>11</td><td>X</td><td>✓</td><td>X</td><td>X</td><td>✓</td><td>✓</td><td>X</td><td>✓</td></tr>
<tr><td>Process evaluation</td><td>12</td><td colspan="2">X</td><td colspan="2">X</td><td colspan="2">✓</td><td colspan="2">N/A</td></tr>
<tr><td>Economic evaluation</td><td>13</td><td>X</td><td>X</td><td>X</td><td>X</td><td>✓</td><td>✓</td><td>✓</td><td>✓</td></tr>
<tr><td>Sample size</td><td>14</td><td colspan="2">?</td><td colspan="2">✓</td><td colspan="2">✓</td><td colspan="2">✓</td></tr>
<tr><td>Analysis</td><td>15</td><td colspan="2">✓</td><td colspan="2">X</td><td colspan="2">✓</td><td colspan="2">✓</td></tr>
<tr><td>Sub-group analyses</td><td>16</td><td colspan="2">N/A</td><td colspan="2">N/A</td><td colspan="2">N/A</td><td colspan="2">N/A</td></tr>
<tr><td>Characteristics</td><td>17</td><td>X</td><td>✓</td><td>✓</td><td>✓</td><td>✓</td><td>✓</td><td>N/A</td><td>✓</td></tr>
<tr><td>Outcomes</td><td>18</td><td>X</td><td>✓</td><td>X</td><td>X</td><td>✓</td><td>✓</td><td>N/A</td><td>✓</td></tr>
<tr><td>Process outcomes</td><td>19</td><td colspan="2">X</td><td colspan="2">X</td><td colspan="2">✓</td><td colspan="2">N/A</td></tr>
<tr><td>Economic evaluation</td><td>20</td><td>X</td><td>X</td><td>X</td><td>X</td><td>✓</td><td>✓</td><td>✓</td><td>✓</td></tr>
<tr><td>Sub-group analyses</td><td>21</td><td colspan="2">X</td><td colspan="2">N/A</td><td colspan="2">N/A</td><td colspan="2">N/A</td></tr>
<tr><td>Fidelity/ adaptation</td><td>22</td><td>X</td><td>X</td><td>X</td><td>X</td><td>✓</td><td>X</td><td>X</td><td>X</td></tr>
<tr><td>Contextual changes</td><td>23</td><td colspan="2">✓</td><td colspan="2">✓</td><td colspan="2">✓</td><td colspan="2">✓</td></tr>
<tr><td>Harms</td><td>24</td><td colspan="2">N/A</td><td colspan="2">N/A</td><td colspan="2">N/A</td><td colspan="2">N/A</td></tr>
<tr><td>Structured discussion</td><td>25</td><td colspan="2">✓</td><td colspan="2">✓</td><td colspan="2">✓</td><td colspan="2">✓</td></tr>
<tr><td>Implications</td><td>26</td><td>X</td><td>X</td><td>✓</td><td>✓</td><td>✓</td><td>✓</td><td>✓</td><td>✓</td></tr>
<tr><td>Statements</td><td>27</td><td colspan="2">✓</td><td colspan="2">?</td><td colspan="2">✓</td><td colspan="2">✓</td></tr>
</table>

Checklist item	Item number	Ramkumar et al. (2019)		Hofstetter et al. (2010)		Kokesh, Ferguson, and Patricoski (2011)		Smith et al. (2012)	
		Implementation strategy	Intervention	Implementation strategy	Intervention	Implementation strategy	Intervention	Implementation strategy	Intervention
Title	1	✓		X		X		?	
Abstract	2	✓		X		X		?	
Introduction	3	✓		✓		✓		✓	
Rationale	4	X	✓	X	✓	X	✓	X	X
Aims and objectives	5	?		✓		X		?	
Design	6	?		?		?		?	
Context	7	✓		X		X		X	
Targeted ‘sites’	8	✓	✓	✓	✓	✓	✓	✓	✓
Description	9	✓	✓	✓	✓	✓	✓	✓	✓
Sub-groups	10	N/A		N/A		✓		N/A	
Outcomes	11	✓	✓	✓	✓	✓	✓	✓	✓
Process evaluation	12	X		X		✓		X	
Economic evaluation	13	✓	✓	✓	✓	✓	✓	X	X
Sample size	14	✓		?		?		?	
Analysis	15	✓		✓		✓		✓	
Sub-group analyses	16	N/A		N/A		✓		N/A	
Characteristics	17	✓	✓	✓	✓	✓	✓	✓	✓
Outcomes	18	✓	✓	✓	✓	✓	✓	✓	✓
Process outcomes	19	X		X		✓		X	
Economic evaluation	20	✓	✓	✓	✓	✓	✓	X	X
Sub-group analyses	21	N/A		N/A		✓		N/A	
Fidelity/ adaptation	22	?	?	X	X	X	X	X	X
Contextual changes	23	✓		X		✓		X	
Harms	24	N/A		N/A		N/A		N/A	
Structured discussion	25	✓		✓		✓		✓	
Implications	26	?	?	X	X	?	?	X	X
Statements	27	✓		✓		✓		✓	

Appendix 7 - StaRI Checklist

<table>
<tr><th rowspan="2">Checklist item</th><th rowspan="2">Item number</th><th colspan="2">Smith et al. (2013)</th><th colspan="2">Smith et al. (2015)</th><th colspan="2">Gupta et al. (2020)</th><th colspan="2">Ravi et al. (2020)</th></tr>
<tr><th>Implementation strategy</th><th>Intervention</th><th>Implementation strategy</th><th>Intervention</th><th>Implementation strategy</th><th>Intervention</th><th>Implementation strategy</th><th>Intervention</th></tr>
<tr><td>Title</td><td>1</td><td colspan="2">✓</td><td colspan="2">?</td><td colspan="2">X</td><td colspan="2">X</td></tr>
<tr><td>Abstract</td><td>2</td><td colspan="2">✓</td><td colspan="2">?</td><td colspan="2">?</td><td colspan="2">✓</td></tr>
<tr><td>Introduction</td><td>3</td><td colspan="2">✓</td><td colspan="2">✓</td><td colspan="2">✓</td><td colspan="2">✓</td></tr>
<tr><td>Rationale</td><td>4</td><td>X</td><td>X</td><td>X</td><td>X</td><td>X</td><td>✓</td><td>X</td><td>✓</td></tr>
<tr><td>Aims and objectives</td><td>5</td><td colspan="2">?</td><td colspan="2">?</td><td colspan="2">?</td><td colspan="2">✓</td></tr>
<tr><td>Design</td><td>6</td><td colspan="2">?</td><td colspan="2">?</td><td colspan="2">?</td><td colspan="2">?</td></tr>
<tr><td>Context</td><td>7</td><td colspan="2">X</td><td colspan="2">X</td><td colspan="2">?</td><td colspan="2">?</td></tr>
<tr><td>Targeted ‘sites’</td><td>8</td><td>?</td><td>?</td><td>?</td><td>?</td><td>✓</td><td>✓</td><td>✓</td><td>✓</td></tr>
<tr><td>Description</td><td>9</td><td>✓</td><td>✓</td><td>?</td><td>?</td><td>✓</td><td>✓</td><td>✓</td><td>✓</td></tr>
<tr><td>Sub-groups</td><td>10</td><td colspan="2">N/A</td><td colspan="2">✓</td><td colspan="2">N/A</td><td colspan="2">N/A</td></tr>
<tr><td>Outcomes</td><td>11</td><td>X</td><td>✓</td><td>X</td><td>✓</td><td>X</td><td>✓</td><td>✓</td><td>✓</td></tr>
<tr><td>Process evaluation</td><td>12</td><td colspan="2">X</td><td colspan="2">X</td><td colspan="2">✓</td><td colspan="2">?</td></tr>
<tr><td>Economic evaluation</td><td>13</td><td>X</td><td>X</td><td>X</td><td>X</td><td>X</td><td>X</td><td>✓</td><td>✓</td></tr>
<tr><td>Sample size</td><td>14</td><td colspan="2">?</td><td colspan="2">?</td><td colspan="2">?</td><td colspan="2">✓</td></tr>
<tr><td>Analysis</td><td>15</td><td colspan="2">✓</td><td colspan="2">✓</td><td colspan="2">✓</td><td colspan="2">✓</td></tr>
<tr><td>Sub-group analyses</td><td>16</td><td colspan="2">N/A</td><td colspan="2">✓</td><td colspan="2">N/A</td><td colspan="2">N/A</td></tr>
<tr><td>Characteristics</td><td>17</td><td>X</td><td>✓</td><td>X</td><td>✓</td><td>✓</td><td>✓</td><td>✓</td><td>✓</td></tr>
<tr><td>Outcomes</td><td>18</td><td>X</td><td>✓</td><td>X</td><td>✓</td><td>X</td><td>✓</td><td>✓</td><td>✓</td></tr>
<tr><td>Process outcomes</td><td>19</td><td colspan="2">X</td><td colspan="2">X</td><td colspan="2">✓</td><td colspan="2">?</td></tr>
<tr><td>Economic evaluation</td><td>20</td><td>X</td><td>X</td><td>X</td><td>X</td><td>X</td><td>X</td><td>✓</td><td>✓</td></tr>
<tr><td>Sub-group analyses</td><td>21</td><td colspan="2">N/A</td><td colspan="2">✓</td><td colspan="2">N/A</td><td colspan="2">N/A</td></tr>
<tr><td>Fidelity/ adaptation</td><td>22</td><td>X</td><td>X</td><td>X</td><td>X</td><td>✓</td><td>X</td><td></td><td></td></tr>
<tr><td>Contextual changes</td><td>23</td><td colspan="2">X</td><td colspan="2">X</td><td colspan="2">✓</td><td colspan="2">✓</td></tr>
<tr><td>Harms</td><td>24</td><td colspan="2">N/A</td><td colspan="2">N/A</td><td colspan="2">N/A</td><td colspan="2">N/A</td></tr>
<tr><td>Structured discussion</td><td>25</td><td colspan="2">✓</td><td colspan="2">✓</td><td colspan="2">✓</td><td colspan="2">✓</td></tr>
<tr><td>Implications</td><td>26</td><td>X</td><td>X</td><td>X</td><td>X</td><td>X</td><td>X</td><td>X</td><td>X</td></tr>
<tr><td>Statements</td><td>27</td><td colspan="2">✓</td><td colspan="2">✓</td><td colspan="2">✓</td><td colspan="2">✓</td></tr>
</table>

Appendix 7 - StaRI Checklist

<table>
<tr><th rowspan="2">Checklist item</th><th rowspan="2">Item number</th><th colspan="2">Dennis, Gladden, and Noe (2012)</th><th colspan="2">Novak et al. (2016)</th><th colspan="2">Luryi et al. (2020)</th><th colspan="2">Skarżyński et al. (2018)</th></tr>
<tr><th>Implementation strategy</th><th>Intervention</th><th>Implementation strategy</th><th>Intervention</th><th>Implementation strategy</th><th>Intervention</th><th>Implementation strategy</th><th>Intervention</th></tr>
<tr><td>Title</td><td>1</td><td colspan="2">X</td><td colspan="2">X</td><td colspan="2">✓</td><td colspan="2">X</td></tr>
<tr><td>Abstract</td><td>2</td><td colspan="2">X</td><td colspan="2">X</td><td colspan="2">X</td><td colspan="2">✓</td></tr>
<tr><td>Introduction</td><td>3</td><td colspan="2">✓</td><td colspan="2">✓</td><td colspan="2">✓</td><td colspan="2">✓</td></tr>
<tr><td>Rationale</td><td>4</td><td>X</td><td>✓</td><td>X</td><td>✓</td><td>X</td><td>✓</td><td>X</td><td>✓</td></tr>
<tr><td>Aims and objectives</td><td>5</td><td colspan="2">✓</td><td colspan="2">✓</td><td colspan="2">?</td><td colspan="2">X</td></tr>
<tr><td>Design</td><td>6</td><td colspan="2">?</td><td colspan="2">?</td><td colspan="2">?</td><td colspan="2">?</td></tr>
<tr><td>Context</td><td>7</td><td colspan="2">✓</td><td colspan="2">✓</td><td colspan="2">?</td><td colspan="2">?</td></tr>
<tr><td>Targeted ‘sites’</td><td>8</td><td>✓</td><td>✓</td><td>✓</td><td>✓</td><td>?</td><td>✓</td><td>✓</td><td>✓</td></tr>
<tr><td>Description</td><td>9</td><td>✓</td><td>✓</td><td>✓</td><td>✓</td><td>?</td><td>✓</td><td>✓</td><td>✓</td></tr>
<tr><td>Sub-groups</td><td>10</td><td colspan="2">N/A</td><td colspan="2">N/A</td><td colspan="2">N/A</td><td colspan="2">N/A</td></tr>
<tr><td>Outcomes</td><td>11</td><td>✓</td><td>✓</td><td>✓</td><td>✓</td><td>?</td><td>✓</td><td>X</td><td>X</td></tr>
<tr><td>Process evaluation</td><td>12</td><td colspan="2">X</td><td colspan="2">✓</td><td colspan="2">✓</td><td colspan="2">✓</td></tr>
<tr><td>Economic evaluation</td><td>13</td><td>✓</td><td>✓</td><td>X</td><td>X</td><td>X</td><td>X</td><td>X</td><td>X</td></tr>
<tr><td>Sample size</td><td>14</td><td colspan="2">✓</td><td colspan="2">✓</td><td colspan="2">?</td><td colspan="2">✓</td></tr>
<tr><td>Analysis</td><td>15</td><td colspan="2">✓</td><td colspan="2">✓</td><td colspan="2">✓</td><td colspan="2">✓</td></tr>
<tr><td>Sub-group analyses</td><td>16</td><td colspan="2">N/A</td><td colspan="2">N/A</td><td colspan="2">N/A</td><td colspan="2">N/A</td></tr>
<tr><td>Characteristics</td><td>17</td><td>✓</td><td>✓</td><td>✓</td><td>✓</td><td>?</td><td>✓</td><td>X</td><td>✓</td></tr>
<tr><td>Outcomes</td><td>18</td><td>✓</td><td>✓</td><td>✓</td><td>✓</td><td>?</td><td>✓</td><td>X</td><td>X</td></tr>
<tr><td>Process outcomes</td><td>19</td><td colspan="2">X</td><td colspan="2">✓</td><td colspan="2">✓</td><td colspan="2">✓</td></tr>
<tr><td>Economic evaluation</td><td>20</td><td>✓</td><td>✓</td><td>X</td><td>X</td><td>X</td><td>X</td><td>X</td><td>X</td></tr>
<tr><td>Sub-group analyses</td><td>21</td><td colspan="2">N/A</td><td colspan="2">N/A</td><td colspan="2">N/A</td><td colspan="2">N/A</td></tr>
<tr><td>Fidelity/ adaptation</td><td>22</td><td>X</td><td>X</td><td>X</td><td>X</td><td>X</td><td>X</td><td>X</td><td>X</td></tr>
<tr><td>Contextual changes</td><td>23</td><td colspan="2">✓</td><td colspan="2">X</td><td colspan="2">✓</td><td colspan="2">✓</td></tr>
<tr><td>Harms</td><td>24</td><td colspan="2">N/A</td><td colspan="2">N/A</td><td colspan="2">N/A</td><td colspan="2">N/A</td></tr>
<tr><td>Structured discussion</td><td>25</td><td colspan="2">✓</td><td colspan="2">✓</td><td colspan="2">✓</td><td colspan="2">✓</td></tr>
<tr><td>Implications</td><td>26</td><td>✓</td><td>✓</td><td>✓</td><td>✓</td><td>?</td><td>✓</td><td>✓</td><td>✓</td></tr>
<tr><td>Statements</td><td>27</td><td colspan="2">?</td><td colspan="2">✓</td><td colspan="2">✓</td><td colspan="2">✓</td></tr>
</table>

<table>
<tr><th rowspan="2">Checklist item</th><th rowspan="2">Item number</th><th colspan="2">Skarżyński et al. (2019)</th><th colspan="2">Houston (2011)</th><th colspan="2">Blaiser et al. (2012)</th></tr>
<tr><th>Implementation strategy</th><th>Intervention</th><th>Implementation strategy</th><th>Intervention</th><th>Implementation strategy</th><th>Intervention</th></tr>
<tr><td>Title</td><td>1</td><td colspan="2">X</td><td colspan="2">X</td><td colspan="2">X</td></tr>
<tr><td>Abstract</td><td>2</td><td colspan="2">X</td><td colspan="2">X</td><td colspan="2">X</td></tr>
<tr><td>Introduction</td><td>3</td><td colspan="2">✓</td><td colspan="2">✓</td><td colspan="2">✓</td></tr>
<tr><td>Rationale</td><td>4</td><td>X</td><td>✓</td><td>X</td><td>✓</td><td>X</td><td>✓</td></tr>
<tr><td>Aims and objectives</td><td>5</td><td colspan="2">?</td><td colspan="2">?</td><td colspan="2">?</td></tr>
<tr><td>Design</td><td>6</td><td colspan="2">?</td><td colspan="2">?</td><td colspan="2">?</td></tr>
<tr><td>Context</td><td>7</td><td colspan="2">?</td><td colspan="2">?</td><td colspan="2">?</td></tr>
<tr><td>Targeted ‘sites’</td><td>8</td><td>✓</td><td>✓</td><td>✓</td><td>✓</td><td>✓</td><td>✓</td></tr>
<tr><td>Description</td><td>9</td><td>✓</td><td>✓</td><td>✓</td><td>✓</td><td>✓</td><td>✓</td></tr>
<tr><td>Sub-groups</td><td>10</td><td colspan="2">N/A</td><td colspan="2">N/A</td><td colspan="2">N/A</td></tr>
<tr><td>Outcomes</td><td>11</td><td>X</td><td>X</td><td>X</td><td>X</td><td>X</td><td>X</td></tr>
<tr><td>Process evaluation</td><td>12</td><td colspan="2">✓</td><td colspan="2">X</td><td colspan="2">✓</td></tr>
<tr><td>Economic evaluation</td><td>13</td><td>X</td><td>X</td><td>X</td><td>X</td><td>X</td><td>X</td></tr>
<tr><td>Sample size</td><td>14</td><td colspan="2">✓</td><td colspan="2">X</td><td colspan="2">X</td></tr>
<tr><td>Analysis</td><td>15</td><td colspan="2">✓</td><td colspan="2">X</td><td colspan="2">X</td></tr>
<tr><td>Sub-group analyses</td><td>16</td><td colspan="2">N/A</td><td colspan="2">N/A</td><td colspan="2">N/A</td></tr>
<tr><td>Characteristics</td><td>17</td><td>X</td><td>✓</td><td>✓</td><td>✓</td><td>✓</td><td>✓</td></tr>
<tr><td>Outcomes</td><td>18</td><td>X</td><td>X</td><td>X</td><td>X</td><td>X</td><td>X</td></tr>
<tr><td>Process outcomes</td><td>19</td><td colspan="2">✓</td><td colspan="2">X</td><td colspan="2">✓</td></tr>
<tr><td>Economic evaluation</td><td>20</td><td>X</td><td>X</td><td>X</td><td>X</td><td>X</td><td>X</td></tr>
<tr><td>Sub-group analyses</td><td>21</td><td colspan="2">N/A</td><td colspan="2">N/A</td><td colspan="2">N/A</td></tr>
<tr><td>Fidelity/ adaptation</td><td>22</td><td>X</td><td>X</td><td>X</td><td>X</td><td>X</td><td>X</td></tr>
<tr><td>Contextual changes</td><td>23</td><td colspan="2">✓</td><td colspan="2">✓</td><td colspan="2">✓</td></tr>
<tr><td>Harms</td><td>24</td><td colspan="2">N/A</td><td colspan="2">N/A</td><td colspan="2">N/A</td></tr>
<tr><td>Structured discussion</td><td>25</td><td colspan="2">✓</td><td colspan="2">✓</td><td colspan="2">✓</td></tr>
<tr><td>Implications</td><td>26</td><td>✓</td><td>X</td><td>X</td><td>X</td><td>✓</td><td>✓</td></tr>
<tr><td>Statements</td><td>27</td><td colspan="2">✓</td><td colspan="2">?</td><td colspan="2">?</td></tr>
</table>

Appendix 7 - StaRI Checklist

Checklist item	Item number	Houston and Stredler-Brown (2012)		Broekelmann (2012)		Lalios (2012)		Galvan, Case, and Todd Houston (2014)	
		Implementation strategy	Intervention	Implementation strategy	Intervention	Implementation strategy	Intervention	Implementation strategy	Intervention
Title	**1**	X		X		X		X	
Abstract	**2**	X		X		X		X	
Introduction	**3**	✓		✓		✓		✓	
Rationale	**4**	X	✓	X	✓	X	✓	X	✓
Aims and objectives	**5**	?		?		?		?	
Design	**6**	?		?		?		?	
Context	**7**	?		?		?		?	
Targeted 'sites'	**8**	✓	✓	✓	✓	✓	✓	✓	✓
Description	**9**	✓	✓	✓	✓	✓	✓	✓	✓
Sub-groups	**10**	N/A		N/A		N/A		N/A	
Outcomes	**11**	X	X	X	X	X	X	X	X
Process evaluation	**12**	✓		✓		✓		X	
Economic evaluation	**13**	X	X	X	X	X	X	X	X
Sample size	**14**	X		X		X		X	
Analysis	**15**	X		X		X		X	
Sub-group analyses	**16**	N/A		N/A		N/A		N/A	
Characteristics	**17**	✓	✓	✓	✓	✓	✓	✓	✓
Outcomes	**18**	X	X	X	X	X	X	X	X
Process outcomes	**19**	X		X		✓		X	
Economic evaluation	**20**	X	X	X	X	X	X	X	X
Sub-group analyses	**21**	N/A		N/A		N/A		N/A	
Fidelity/ adaptation	**22**	X	X	X	X	X	X	X	X
Contextual changes	**23**	✓		✓		✓		✓	
Harms	**24**	N/A		N/A		N/A		N/A	
Structured discussion	**25**	✓		✓		✓		✓	
Implications	**26**	X	X	✓	✓	X	✓	X	X
Statements	**27**	?		?		?		?	

<table>
<tr><th rowspan="2">Checklist item</th><th rowspan="2">Item number</th><th colspan="2">McCarthy, Muñoz, and White (2010)</th><th colspan="2">McCarthy (2011)</th><th colspan="2">McCarthy (2012)</th><th colspan="2">Gladden, Beck, and Chandler (2015)</th></tr>
<tr><th>Implementation strategy</th><th>Intervention</th><th>Implementation strategy</th><th>Intervention</th><th>Implementation strategy</th><th>Intervention</th><th>Implementation strategy</th><th>Intervention</th></tr>
<tr><td>Title</td><td>1</td><td colspan="2">X</td><td colspan="2">X</td><td colspan="2">X</td><td colspan="2">?</td></tr>
<tr><td>Abstract</td><td>2</td><td colspan="2">X</td><td colspan="2">X</td><td colspan="2">X</td><td colspan="2">?</td></tr>
<tr><td>Introduction</td><td>3</td><td colspan="2">✓</td><td colspan="2">✓</td><td colspan="2">✓</td><td colspan="2">✓</td></tr>
<tr><td>Rationale</td><td>4</td><td>X</td><td>✓</td><td>X</td><td>✓</td><td>X</td><td>✓</td><td>X</td><td>✓</td></tr>
<tr><td>Aims and objectives</td><td>5</td><td colspan="2">?</td><td colspan="2">?</td><td colspan="2">?</td><td colspan="2">✓</td></tr>
<tr><td>Design</td><td>6</td><td colspan="2">?</td><td colspan="2">?</td><td colspan="2">?</td><td colspan="2">?</td></tr>
<tr><td>Context</td><td>7</td><td colspan="2">?</td><td colspan="2">✓</td><td colspan="2">?</td><td colspan="2">✓</td></tr>
<tr><td>Targeted ‘sites’</td><td>8</td><td>✓</td><td>✓</td><td>✓</td><td>✓</td><td>✓</td><td>✓</td><td>✓</td><td>✓</td></tr>
<tr><td>Description</td><td>9</td><td>✓</td><td>✓</td><td>✓</td><td>✓</td><td>✓</td><td>✓</td><td>✓</td><td>✓</td></tr>
<tr><td>Sub-groups</td><td>10</td><td colspan="2">N/A</td><td colspan="2">N/A</td><td colspan="2">N/A</td><td colspan="2">✓</td></tr>
<tr><td>Outcomes</td><td>11</td><td>X</td><td>X</td><td>✓</td><td>X</td><td>✓</td><td>X</td><td>✓</td><td>✓</td></tr>
<tr><td>Process evaluation</td><td>12</td><td colspan="2">X</td><td colspan="2">✓</td><td colspan="2">✓</td><td colspan="2">✓</td></tr>
<tr><td>Economic evaluation</td><td>13</td><td>X</td><td>X</td><td>X</td><td>X</td><td>X</td><td>X</td><td>X</td><td>X</td></tr>
<tr><td>Sample size</td><td>14</td><td colspan="2">X</td><td colspan="2">X</td><td colspan="2">X</td><td colspan="2">✓</td></tr>
<tr><td>Analysis</td><td>15</td><td colspan="2">X</td><td colspan="2">X</td><td colspan="2">X</td><td colspan="2">✓</td></tr>
<tr><td>Sub-group analyses</td><td>16</td><td colspan="2">N/A</td><td colspan="2">N/A</td><td colspan="2">N/A</td><td colspan="2">✓</td></tr>
<tr><td>Characteristics</td><td>17</td><td>✓</td><td>✓</td><td>✓</td><td>✓</td><td>✓</td><td>✓</td><td>✓</td><td>✓</td></tr>
<tr><td>Outcomes</td><td>18</td><td>X</td><td>X</td><td>✓</td><td>X</td><td>✓</td><td>X</td><td>✓</td><td>✓</td></tr>
<tr><td>Process outcomes</td><td>19</td><td colspan="2">X</td><td colspan="2">✓</td><td colspan="2">✓</td><td colspan="2">✓</td></tr>
<tr><td>Economic evaluation</td><td>20</td><td>X</td><td>X</td><td>X</td><td>X</td><td>X</td><td>X</td><td>X</td><td>X</td></tr>
<tr><td>Sub-group analyses</td><td>21</td><td colspan="2">N/A</td><td colspan="2">N/A</td><td colspan="2">N/A</td><td colspan="2">✓</td></tr>
<tr><td>Fidelity/ adaptation</td><td>22</td><td>X</td><td>X</td><td>X</td><td>X</td><td>X</td><td>X</td><td>✓</td><td>X</td></tr>
<tr><td>Contextual changes</td><td>23</td><td colspan="2">✓</td><td colspan="2">✓</td><td colspan="2">✓</td><td colspan="2">✓</td></tr>
<tr><td>Harms</td><td>24</td><td colspan="2">N/A</td><td colspan="2">N/A</td><td colspan="2">N/A</td><td colspan="2">N/A</td></tr>
<tr><td>Structured discussion</td><td>25</td><td colspan="2">✓</td><td colspan="2">✓</td><td colspan="2">✓</td><td colspan="2">✓</td></tr>
<tr><td>Implications</td><td>26</td><td>✓</td><td>✓</td><td>✓</td><td>✓</td><td>✓</td><td>✓</td><td>✓</td><td>✓</td></tr>
<tr><td>Statements</td><td>27</td><td colspan="2">?</td><td colspan="2">?</td><td colspan="2">?</td><td colspan="2">?</td></tr>
</table>

Appendix 7 - StaRI Checklist

<table>
<tr><th rowspan="2">Checklist item</th><th rowspan="2">Item number</th><th colspan="2">Ratanjee-Vanmali, Swanepoel, and Laplante-Lévesque (2020)</th></tr>
<tr><th>Implementation strategy</th><th>Intervention</th></tr>
<tr><td>Title</td><td>1</td><td colspan="2">X</td></tr>
<tr><td>Abstract</td><td>2</td><td colspan="2">X</td></tr>
<tr><td>Introduction</td><td>3</td><td colspan="2">✓</td></tr>
<tr><td>Rationale</td><td>4</td><td>X</td><td>✓</td></tr>
<tr><td>Aims and objectives</td><td>5</td><td colspan="2">✓</td></tr>
<tr><td>Design</td><td>6</td><td colspan="2">?</td></tr>
<tr><td>Context</td><td>7</td><td colspan="2">?</td></tr>
<tr><td>Targeted ‘sites’</td><td>8</td><td>X</td><td>✓</td></tr>
<tr><td>Description</td><td>9</td><td>X</td><td>✓</td></tr>
<tr><td>Sub-groups</td><td>10</td><td colspan="2">N/A</td></tr>
<tr><td>Outcomes</td><td>11</td><td>X</td><td>✓</td></tr>
<tr><td>Process evaluation</td><td>12</td><td colspan="2">X</td></tr>
<tr><td>Economic evaluation</td><td>13</td><td>X</td><td>X</td></tr>
<tr><td>Sample size</td><td>14</td><td colspan="2">✓</td></tr>
<tr><td>Analysis</td><td>15</td><td colspan="2">✓</td></tr>
<tr><td>Sub-group analyses</td><td>16</td><td colspan="2">N/A</td></tr>
<tr><td>Characteristics</td><td>17</td><td>X</td><td>✓</td></tr>
<tr><td>Outcomes</td><td>18</td><td>X</td><td>✓</td></tr>
<tr><td>Process outcomes</td><td>19</td><td colspan="2">X</td></tr>
<tr><td>Economic evaluation</td><td>20</td><td>X</td><td>X</td></tr>
<tr><td>Sub-group analyses</td><td>21</td><td colspan="2">N/A</td></tr>
<tr><td>Fidelity/ adaptation</td><td>22</td><td>X</td><td>X</td></tr>
<tr><td>Contextual changes</td><td>23</td><td colspan="2">✓</td></tr>
<tr><td>Harms</td><td>24</td><td colspan="2">N/A</td></tr>
<tr><td>Structured discussion</td><td>25</td><td colspan="2">✓</td></tr>
<tr><td>Implications</td><td>26</td><td>X</td><td>X</td></tr>
<tr><td>Statements</td><td>27</td><td colspan="2">✓</td></tr>
</table>

Supplemental file - 5

Implementation Factors influencing the sustained provision of tele-audiology projects – mapped to Implementation Outcomes Framework (Proctor et al., 2011)

Themes	Facilitators
**Acceptability**	**Patient's perspectives:**<br>● ***Improved accessibility to service provider and multiple locations (P19) (P8) (P8) (P5)***<br>● ***Reduced waiting time and test time (P7) (P19) (P19) (P8) (PS2)***<br>● ***Close proximity/ Reasonable/ Reduced travel time to testing facility (P19) (P14) (P5) (P14)***<br>● Elimination of occupational health and safety issues related to travel (P19)<br>● ***Consistency and regularity of sessions, reduced cancellations (P15) and thereby completion of testing with minimal visits (P14)***<br>● Improved quality of life (P14)<br>● Implemented in the child's natural environment and preparing children with hearing loss for inclusion in their community, home, or school (P16)<br>● ***Comfortable with the environment and communicating in remote assembly settings (P20) (P14) (P5)***<br>● Clinician engagement: introduction to lead audiologist, personalized care, confidence, knowledge and understanding (P3)<br>● ***Families strongly recommended to visit for face-to-face consultation prior to telepractice sessions; Either family travels to consultation site or Audiologist travels to remote site (P19) (P5)***<br>● ***Provision of hybrid model as an option to hesitant parents (P15) (P18)***<br>● ***Counseling, emotional support and reassurance via videoconferencing (P19) (P20) (P18)***<br>● ***Early-intervention specialist is able to directly observe the parent-child interactions and provide responsive feedback that can be applied immediately (P19) (P19) (P8)***<br>● ***Participation and assistance from other family members is encouraged which thereby enables empowerment of parents as primary participants (P19) (P15) (P15)***<br>● Parents and caregivers readily assume their role as the primary facilitator of their child's development, and subsequently parental abilities appear to improve at a faster rate (P17)<br>● ***Presence and motivation of a local reliable and accessible VHW/ technical staff (P19) (P8)***<br>● ***A safe, secure environment is necessary to maintain a trusting and effective therapeutic relationship since the patient needs to have a sense of security and their privacy respected (P16) (P5)***<br>● Effective parent-professional partnership can be established and maintained using this delivery model (P17)<br>● Families developing greater understanding, knowledge and confidence of child's condition (P19)<br>● High quality consultation experience (P20)

For peer review only

	• Having a support network reduces the feelings of isolation experienced by families and provides a continuing sense of solidarity for families (P19)<br>• Interfere with the rapport and personal interaction (P11)<br>• Additional effort required to learn technology and might be intimidated by technology (P15)<br>• Contact with a personnel was not as good as during a traditional visit (P14)<br>• Lip reading cues can be difficult to perceive via telepractice and additional supports may be necessary for these parents (P19)
**Adoption**	**Provider's perspectives:**<br>• Interest among the personnel to implement the program (P12)<br>• Early intervention and cultural shifts in attitude (P20)<br>• Reducing loss to follow up (P4)<br>• Increasing the efficacy, efficiency, and equity of the EHDI program is a motivator for providers (P4)<br>• Audiologist promoting technology literacy (P21)<br>• Increased awareness regarding hearing (P6)<br>• Self-referral by older adults for hearing testing (P6)<br>• Requests for testing hearing of older siblings (P6)<br>• Planning sessions takes time (P15)
**Feasibility**	**Human resources:**<br>• Tele Practitioner should have heightened awareness of anticipation and remote management skills to assist in obtaining the learning objectives of both the parent and child (P17)<br>• Audiologist needs to take a lead role in the development, implementation and refinement of the service model (P5)<br>• ***Collaboration, coordination and communication between all personnel involved can result in reliable patient outcomes (P11) (P12) (P5)***<br>• ***Designated personnel for each role; such as IT Specialist/ Tele-technician (P6) (P15)***<br>• The onus is entrusted to the personnel recruited (P6)<br>• Community health worker and coordinator must have good interpersonal communication skills and knowledge of the local language (P9)<br>• Training conducted in local language using demonstrations and handouts (P6)<br>• Knowledge and skill retention assessed periodically post-training in a structured format (P6)<br>• Regular training and supervision is recommended to improve health workers' ability to successfully screen (P6)<br>• ***Stationary, video training and detailed training on the equipment for complex procedures (P12) (P14)***<br>• Flexibility to adapt materials according to need (P12)<br>• Personnel have to adapt and problem-solve in real time during various situations (P12)<br>• Lack of trained tele-technicians, shortage of professionals (P15)

	• Need for faculty full-time equivalency (P12)<br><br>**<u>Infrastructure - Provider:</u>**<br>• Mobile telemedicine van with satellite connection and built in facilities for video conferencing, testing and A/C (P6)<br>• Generous space acquisition and infrastructure support (P12)<br>• Appropriate lighting and sound with limited environmental distractions as well as adequate physical space for parent, child, and materials within view of the camera is mandated (P17)<br>• Physical limitations of operating in a sound booth (P5)<br>• Small space, low light conditions (P5)<br><br>**<u>Organization Acceptance:</u>**<br>• The involvement of audiology graduate students in training (P3)<br>• High level administrative support (University presidents, deans, and department chairs) (P12)<br><br>**<u>Planning and implementation:</u>**<br>• ***Assessment of patient needs and internal resources for program start-up and sustainability (PS3) (P20)***<br>• ***A robust business model/plan/ proposal, internal and external needs assessment is critical that is unique to the project (P20) (P9)***<br>• ***A site-visit, first visit, pre-feasibility analysis, barrier study could be useful (P9) (P5)***<br>• ***A test run / test environment to simulate operational conditions and validate functional requirements (P18) (P5)***<br>• A pilot project at the remote site to assess operability and workflow issues (P5)<br>• Optimise existing infrastructure and personnel (P5)<br>• A service-level analysis of averted costs from the perspective of the provider to determine the allocation efficiency (P5)<br>• Systematic framework for collecting data, that monitors program satisfaction and tracking the efficacy of the program (P16)<br>• Careful planning and coordination (P11)<br>• Support and buy-in (ownership) from clinical staff (audiologists and other related professionals) and organizational leaders (P20)<br>• Development of an enterprise consciousness that includes systems-thinking and broader concerns beyond a single program (P20)<br>• A systematic nature of programme implementation, can enable surveillance capability which allows to monitor changes over time and be more responsive to changing health needs in the community (P8)<br>• Development of protocols, standards of care, best practices, and toolkits to guide implementation (P20)

For peer review only

	• Targeted evaluation plan (P20)<br>• Openness to feedback and flexibility that allows for modification of work processes (P20)<br>• Embrace and lead a transformational period with the highest level of integrity (P20)<br>• Conduct formal assessments every 6 months to monitor patient progress (P19)<br>• Prioritize ease of use and affordability (P1)<br>• Business process interoperability (common policies and support structures) need to be achieved across health authorities (P5)<br>• Absence of a solid model for telemedicine cost analysis (P3)<br><br>**Resource management**<br>• Involvement of all stakeholders for the design of service delivery model (P5)<br>• Determination of resources to achieve standardization, interoperability of technology, and connectivity (P20)<br>• ***Memorandum of Understanding (MOU), Telehealth Service Agreement (TSA), A letter of agreement that delineates project responsibilities, equipment, supply, cost and work sharing between stakeholders (P11) (P5)***<br>• Commitment to adequate resourcing, staff education and training (P20)<br>• Strong partnerships with industry, technical for resource management(information technology, biomedical), and administration staff (P20)<br>• Incorporate aspects of social networking as well as blogging and facilitates collaboration, information sharing, and parent training (P19)<br>• Use of creative and focused lesson plans (P16)<br>• Equipment re-allocation as families leave the program (P19)<br>• EMR system with digital flow sheets, which enables documentation of audiometric, hearing health history and hearing or communication handicap data (P12)<br>• Online options for HHC may be important avenues for adults with hearing loss (P21)<br><br>**Standard Operating Protocol:**<br>• ***Materials and educational package necessary for instruction sent to the families via the postal service or over the Internet (P19) (P15) (P18)***<br>• Documentation of multimedia data for future reference using store and forward telepractice (P7)<br>• ***Electronic data integration into electronic health records (P7) (P20)***<br>• Tracking of cases for time studies and administrative purposes (P7)<br>• ***Use of strict inclusion and candidature criteria (P15) (P18)***

For peer review only

	• Unique identifier (UIN) on all forms and communications to allow for data linkage across sources (P1)<br>• Monitor the use of telepractice program through chart reviews in the multi organisational electronic medical record (EMR) (P1)<br>• On-going assessments and surveys (P15)<br><br>**Logistics:**<br>• Flexibility for rescheduling (P19)<br>• Accommodates for time differences by offering extended operating hours (P19)<br>• When circumstances results in cancelled appointments, personnel can seamlessly shift their attention to other clinic priorities (P5)<br>• Problem solving between the audiometric technician and audiologist can be conducted with relative ease due to consistency between multiple sites (P5)<br>• Referrals managed within fewer sessions and with an overall shorter duration of contact events<br>• Flexibility to conduct testing at different sites (P10)<br><br>**Sociocultural aspects:**<br>• Mobilizing community participation and monitoring follow up (P6)<br>• Family and student interpreters for different languages (P12)<br>• Community involvement, participation and engagement (P1)<br>• ***Continuous and on-going consultation and collaboration with local council and community in an appropriate and culturally safe manner (P8) (P8) (P8) (P1)***<br>• ***Strong indigenous leadership (P8) (P8) (P1)***<br>• ***Close alignment with primary care services in the community (P8) (P8)***<br>• Collaboration with the family's local birth-to-3 program enabled a computer to be purchased through an early intervention family grant program (P17)<br><br>**Government laws:**<br>• A state/ National database to document, maintain records, track and follow up (P3)<br>• Not covered by practice acts and state laws (P11)<br>• Not being covered by malpractice liability (P11)<br>• Challenges with providing practitioners with credentials and privileges (P11)<br><br>**Privacy and confidentiality - Provider:**<br>• Secured and encrypted virtual private network (P13)

For peer review only

	• The client file is maintained under that agency's privacy policies and the applicable audiology governing policy. The host site may track the number of tests performed for statistical and billing purposes, but does not record client information (P4)<br>• Including biometric records - fingerprints, retina and iris patterns, voice waves, signatures, and pictures (P16)
**Implementation cost**	**Provider:**<br>• Having multiple sources of funding could be useful; including charitable donations and government (P19) (P20)<br>• Lower total cost outcome (P10)<br>• Funding for hiring IT support<br>• Services not reimbursed by insurance in some cases (P11)<br><br>**Patient/Beneficiary:**<br>• ***Reduced cost of travelling and testing (P12) (P14) (P14)***<br>• Financial assistance for travel expenses and free on-site accommodation for initial face-to-face consultation (P19)

References:

Proctor, E., Silmere, H., Raghavan, R., Hovmand, P., Aarons, G., Bunger, A., Griffey, R., & Hensley, M. (2011). Outcomes for implementation research: Conceptual distinctions, measurement challenges, and research agenda. *Administration and Policy in Mental Health and Mental Health Services Research*, *38*(2), 65–76. https://doi.org/10.1007/s10488-010-0319-7

For peer review only

Supplemental file 6
COREQ (COnsolidated criteria for REporting Qualitative research) Checklist

A checklist of items that should be included in reports of qualitative research. You must report the page number in your manuscript where you consider each of the items listed in this checklist. If you have not included this information, either revise your manuscript accordingly before submitting or note N/A.

Topic	Item No.	Guide Questions/Description	Reported on Page No.
**Domain 1: Research team and reflexivity**			
Personal characteristics			
Interviewer/facilitator	1	Which author/s conducted the interview or focus group?	7
Credentials	2	What were the researcher's credentials? E.g. PhD, MD	1
Occupation	3	What was their occupation at the time of the study?	1
Gender	4	Was the researcher male or female?	N/A
Experience and training	5	What experience or training did the researcher have?	7
Relationship with Participants			
Relationship established	6	Was a relationship established prior to study commencement?	7
Participant knowledge of the interviewer	7	What did the participants know about the researcher? e.g. personal goals, reasons for doing the research	7
Interviewer characteristics	8	What characteristics were reported about the inter viewer/facilitator? e.g. Bias, assumptions, reasons and interests in the research topic	7
**Domain 2: Study design**			
Theoretical framework			
Methodological orientation and Theory	9	What methodological orientation was stated to underpin the study? e.g. grounded theory, discourse analysis, ethnography, phenomenology, content analysis	7
Participant selection			
Sampling	10	How were participants selected? e.g. purposive, convenience, consecutive, snowball	7
Method of approach	11	How were participants approached? e.g. face-to-face, telephone, mail, email	7
Sample size	12	How many participants were in the study?	7
Non-participation	13	How many people refused to participate or dropped out? Reasons?	7
Setting			
Setting of data collection	14	Where was the data collected? e.g. home, clinic, workplace	7
Presence of non-participants	15	Was anyone else present besides the participants and researchers?	7
Description of sample	16	What are the important characteristics of the sample? e.g. demographic data, date	7
Data collection			
Interview guide	17	Were questions, prompts, guides provided by the authors? Was it pilot tested?	8
Repeat interviews	18	Were repeat inter views carried out? If yes, how many?	8
Audio/visual recording	19	Did the research use audio or visual recording to collect the data?	8
Field notes	20	Were field notes made during and/or after the inter view or focus group?	8
Duration	21	What was the duration of the inter views or focus group?	8
Data saturation	22	Was data saturation discussed?	8

For peer review only

Transcripts returned	23	Were transcripts returned to participants for comment and/or	8

For peer review only

Topi c	Item No.	Guide Questions/Description	Reported on Page No.
		correction?	
**Domain 3: analysis and findings**			
Data analysis			
Number of data coders	24	How many data coders coded the data?	8
Description of the coding tree	25	Did authors provide a description of the coding tree?	8
Derivation of themes	26	Were themes identified in advance or derived from the data?	8
Software	27	What software, if applicable, was used to manage the data?	8
Participant checking	28	Did participants provide feedback on the findings?	8
Reporting			
Quotations presented	29	Were participant quotations presented to illustrate the themes/findings? Was each quotation identified? e.g. participant number	8
Data and findings consistent	30	Was there consistency between the data presented and the findings?	8
Clarity of major themes	31	Were major themes clearly presented in the findings?	8
Clarity of minor themes	32	Is there a description of diverse cases or discussion of minor themes?	8

Developed from: Tong A, Sainsbury P, Craig J. Consolidated criteria for reporting qualitative research (COREQ): a 32-item checklist for interviews and focus groups. *International Journal for Quality in Health Care*. 2007. Volume 19, Number 6: pp. 349 – 357

Once you have completed this checklist, please save a copy and upload it as part of your submission. DO NOT include thischecklist as part of the main manuscript document. It must be uploaded as a separate file.

For peer review only

Supplemental file 7
Interview Guides

Interview time: _________

Hello Dr./ Mr. /Ms. /Mrs.,
I am Vidya, I am an audiologist and SLP working as a professional since 2008, and have spent all of these years in telepractice for rural community based programs here in India. I am based at SRIHER, Chennai, India.

I thank you for giving consent to participate in the study.

This interview to understand in detail about your experience (both positive and negative), in implementing your project titled_____(project title)______________.

The interview will take between 30-45 minutes to be completed.

Before we begin, I would like to know if you have any questions that I can address/ clarify to you.

We will now begin the interview. I will start recording our conversation for the purpose of transcription and analysis of data.

Interview begins

Dear Dr./ Mr. /Ms. /Mrs.,
From reading your work "___________________", I have gathered that you examined ____ ____.

Guides:

1. How would you describe your efforts towards working in the area of telepractice?
E.g as research initiatives to contribute to the understanding of telepractice/ work towards immediate translation to service delivery in your work location/ or anything else?

2. Could you briefly describe the current status of implementation of your telepractice service for individuals with autism?
Sustained/ On-going/ non-viable/ waiting for a,b,c to start as routine?

3. Could you describe the **resources** that influenced (either positively or negatively) the implementation/ attempt to implement the telepractice service?
- Financial/Economic
- Human - professional, e-helpers/ care takers/ technical help/ facilitator
- Infrastructure-
- Equipment/ technology
- Technical/internet

Were there any non-adaptable factors that may have affected the implementation?

4. What about **the end-users** Comfort/ Acceptance/ Perceptions/ Sociocultural?

For peer review only

Motivators/ influencers
Inherent patient related factors?

5. **Organizational**

- Acceptance
- Policy Making/decision
- SOP
- Infrastructure for implementation (e-bills/staff allocation)
- Provider comfort/ ease/ acceptance/ perceptions?

6. **Ethics/ Legality**

- Government laws
- Consent and other ethical considerations

7. **Test specific/ Disorder Specific implementation factors**

8. **Sustainability- self sustaining without external project funding**

For peer review only

Supplemental file 8

Table 1a: Summary of articles identified in tele-audiology

Code	Study title	Authors	Country/ *Area*	Participants	Focus area
A12010	eEHDI : Functions and Challenges	Campbell & Hyde (2010)	Ontario, Canada *Rural areas*	Infants	Diagnostic audiological evaluation
A22010	The impact of telehealth on wait time for ENT specialty care	Hofstetter, Kokesh, Ferguson, & Hood (2010)	Alaska, United States of America *Rural areas*	Mixed Population	Combination of diagnostic audiological and otorhinolaryngological evaluation
A32010	Teleintervention for Infants and Young Children Who Are Deaf or Hard-of-Hearing	McCarthy, Muñoz, & White (2010)	Sydney, Australia *Rural areas*	Children	Aural re/habilitation
A42011	TeleIntervention: Improving Service Delivery to Young Children With Hearing Loss and Their Families Through Telepractice	Houston (2011)	Utah, United States of America *Mixed areas*	Children	Aural re/habilitation
A52011	The Alaska experience using store-and-forward telemedicine for ENT care in Alaska	Kokesh, Ferguson, & Patricoski (2011)	Alaska, United States of America *Rural areas*	Mixed Population	Combination of diagnostic audiological and otorhinolaryngological evaluation
A62011	Using technology to support children with sensory disability in remote areas: the RIDBC Teleschool Model	McCarthy (2011)	Sydney, Australia *Rural areas*	Children	Aural re/habilitation
A72012	Telepractice Services at Sound Beginnings at Utah State University	Blaiser, Edwards, Behl, & Munoz (2012)	Utah, United States of America *Mixed areas*	Children	Aural re/habilitation
A82012	ihear® Internet Therapy Program - A Program by St. Joseph Institute for the Deaf	Broekelmann (2012)	Missouri, United States of America *Rural areas*	Children - Adults	Aural re/habilitation
A92012	Telepractice in the Department of Veterans Affairs	Dennis, Gladden, & Noe (2012)	Washington, United States of America *Mixed areas*	Mixed Population	Hearing aid fitting and programming

For peer review only

A102012	A Model of Early Intervention for Children with Hearing Loss Provided through Telepractice	Houston & Stredler-Brown (2012)	Utah, United States of America<br>*Rural areas*	Children	Aural re/habilitation
A112012	ConnectHear TeleIntervention Program	Lalios (2012)	Wisconsin, United States of America<br>*Mixed areas*	Children - Adolescents	Aural re/habilitation
A122012	RIDBC Teleschool (TM): A hub of expertise	McCarthy (2012)	Sydney, Australia<br>*Rural areas*	Children	Aural re/habilitation
A132012	A mobile telemedicine-enabled ear screening service for Indigenous children in Queensland: activity and outcomes in the first three years	Smith, Armfield, Wu, Brown, & Perry (2012)	Queensland, Australia<br>*Rural areas*	Children	Combination of diagnostic audiological and otorhinolaryngological evaluation
A142013	Changes in paediatric hospital ENT service utilisation following the implementation of a mobile, indigenous health screening service	Smith et al. (2013)	Queensland, Australia<br>*Rural areas*	Children	Combination of diagnostic audiological and otorhinolaryngological evaluation
A152014	Listening and Learning: Using Telepractice to Serve Children and Adults with Hearing Loss	Galvan, Case, & Todd Houston (2014)	Ohio, United States of America<br>*Rural areas*	Mixed Population	Aural re/habilitation
A162015	Tele-audiology: Expanding access to hearing care and enhancing patient connectivity	Gladden, Beck, & Chandler (2015)	Washington, United States of America<br>*Rural areas*	Mixed Population	Comprehensive audiological services
A172015	Monitoring ear health through a telemedicine-supported health screening service in Queensland	Smith et al. (2015)	Queensland, Australia<br>*Rural areas*	Children - Adolescents	Combination of diagnostic audiological and otorhinolaryngological evaluation
A182016	Reducing Loss to Follow-Up with Tele-audiology Diagnostic Evaluations.	Dharmar et al. (2016)	California, United States of America<br>*Rural areas*	Infants	Diagnostic audiological evaluation
A192016	The Future of Healthcare Delivery : IPE / IPP Audiology and Nursing Student / Faculty Collaboration to	Novak, Cantu, Zappler, Coco, & Champlin (2016)	Texas, United States of America<br>*Urban areas*	Mixed Population	Hearing aid fitting and programming

For peer review only

	Deliver Hearing Aids to Vulnerable Adults via Telehealth				
A202016	Infant Diagnostic Evaluation via Teleaudiology Following Newborn Screening in Eastern North Carolina	Stuart (2016)	North Carolina, United States of America<br>*Rural areas*	Infants	Diagnostic audiological evaluation
A212018	Cost and outcome of a community-based paediatric hearing screening programme in rural India with application of tele-audiology for follow-up diagnostic hearing assessment	Ramkumar et al. (2018)	Tamil Nadu, India<br>*Rural areas*	Children	Diagnostic audiological evaluation
A222018	Comprehensive approach to the National Network of Teleaudiology in World Hearing Center in Kajetany, Poland	Skarżyński, Świerniak, Bruski, Ludwikowski, & Skarżyński (2018)	Kajetany, Poland<br>*Rural areas*	Mixed Population	Cochlear Implants fitting and mapping
A232019	Hearing Norton Sound: Mixed methods protocol of a community randomised trial to address childhood hearing loss in rural Alaska	Emmett, Robler, Gallo, et al. (2019)	Alaska, United States of America<br>*Rural areas*	Children - Adolescents	Hearing screening
A242019	Hearing Norton Sound: a community randomised trial protocol to address childhood hearing loss in rural Alaska.	Emmett, Robler, Wang, et al. (2019)	Alaska, United States of America<br>*Rural areas*	Children - Adolescents	Hearing screening
A252019	Comprehensive Community Screening of Otological Patients by Trained Technicians Using a Telemedicine Device: An Efficient and Cost-Effective Way to Triage Patients With Ear Diseases	Gupta et al. (2019)	Delhi, India<br>*Rural areas*	Mixed Population	Combination of diagnostic audiological and otorhinolaryngological evaluation
A262019	Telehealth-enabled auditory brainstem response testing for infants living in rural communities: the British Columbia Early Hearing Program experience	Hatton, Rowlandson, Beers, & Small (2019)	British Columbia, Canada<br>*Rural areas*	Infants	Diagnostic audiological evaluation

For peer review only

A272019	Implementation and evaluation of a rural community-based pediatric hearing screening program integrating in-person and tele-diagnostic auditory brainstem response (ABR)	Ramkumar, Nagarajan, Shankarnarayan, Kumaravelu, & Hall (2019)	Tamil Nadu, India *Rural areas*	Children	Diagnostic audiological evaluation
A282019	Telefitting Between Kajetany, Poland and Odessa, Ukraine for Cochlear Implants	Skarżyński, Swierniak, Ludwikowski, & Bruski (2019)	Kajetany, Poland *Rural areas*	Mixed Population	Cochlear Implant fitting and mapping
A292020	Cochlear Implant Mapping Through Telemedicine—A Feasibility Study	Luryi et al. (2020)	Connecticut, United States of America *Urban areas*	Geriatric	Cochlear Implant fitting and mapping
A302020	Digital Proficiency Is Not a Significant Barrier for Taking Up Hearing Services With a Hybrid Online and Face-to-Face Model	Ratanjee-Vanmali, Swanepoel, & Laplante-Lévesque (2020)	Durban, South Africa *Urban areas*	Adults	Comprehensive audiological services
A312020	Tele-Audiological Surveillance of Middle Ear Status among Individuals with Cleft Lip and/or Palate in Rural South India	Ravi et al. (2020)	Tamil Nadu, India *Rural areas*	Mixed Population	Combination of diagnostic audiological and otorhinolaryngological evaluation
A322020	Hearing Norton Sound: community involvement in the design of a mixed methods community randomized trial in 15 Alaska Native communities	Robler et al. (2020)	Alaska, United States of America *Rural areas*	Children - Adolescents	Hearing screening

For peer review only

Table 1b: Summary of articles identified in tele-audiology (Contd.)

Code	Study title	Authors	Settings	Personnel/ e-helper/telepractice assistant	Type of services
A12010	eEHDI : Functions and Challenges	Campbell & Hyde (2010)	Community clinic	Remote site Technician	Tone specific ABR tracings at 500 and 2000 Hz<br>Diagnostic otoacoustic emissions (OAE) at 1 – 4 kHz
A22010	The impact of telehealth on wait time for ENT specialty care	Hofstetter, Kokesh, Ferguson, & Hood (2010)	Community clinic	Initiating provider:<br>Audiologists<br>Physicians<br>Public health nurses<br>Dentists<br>Physician assistants<br>Staff of the infant learning program<br><br>Consulting provider:<br>ENT Specialist	Patient demographics<br>Clinical data<br>Images and results from diagnostic tests
A32010	Teleintervention for Infants and Young Children Who Are Deaf or Hard-of-Hearing	McCarthy, Muñoz, & White (2010)	Home-based	Specially trained early-intervention specialist	Early intervention services
A42011	TeleIntervention: Improving Service Delivery to Young Children With Hearing Loss and Their Families Through Telepractice	Houston (2011)	Home-based	Speech-language pathologist	AV Therapy: Listening and language skills<br>Parent coaching<br>Family centered practices
A52011	The Alaska experience using store-and-forward telemedicine for ENT care in Alaska	Kokesh, Ferguson, & Patricoski (2011)	Community clinics<br>Hospitals	Health care providers	History<br>Images of the tympanic membrane<br>Audiometry<br>Tympanometry

For peer review only

A62011	Using technology to support children with sensory disability in remote areas: the RIDBC Teleschool Model	McCarthy (2011)	Home-based	RIDBC Teleschool staff - professionals<br>Local professionals<br>Technical support person<br>Local technical staff	Early intervention services
A72012	Telepractice Services at Sound Beginnings at Utah State University	Blaiser, Edwards, Behl, & Munoz (2012)	Home-based	Speech-language pathologist or<br>Educators of the deaf	AV Therapy: Listening and language skills
A82012	ihear® Internet Therapy Program - A Program by St. Joseph Institute for the Deaf	Broekelmann (2012)	Home-based<br>School-based	ihear therapist:<br>Deaf educator/ Speech-language pathologist/<br>Special educator	Effective, individualized, and interactive therapy: listening and spoken language
A92012	Telepractice in the Department of Veterans Affairs	Dennis, Gladden, & Noe (2012)	Community clinics	Clinicians<br>Audiology assistants<br>Telehealth clinical technicians<br>Information technology (IT) experts<br>Biomedical staff	Remote hearing aid programming and verification via telehealth
A102012	A Model of Early Intervention for Children with Hearing Loss Provided through Telepractice	Houston & Stredler-Brown (2012)	Home-based	Speech-language pathologist	AV Therapy: Listening and language skills<br>Parent coaching<br>Family centered practices
A112012	ConnectHear TeleIntervention Program	Lalios (2012)	Home-based	LSLS certified professionals	AV Therapy
A122012	RIDBC Teleschool (TM): A hub of expertise	McCarthy (2012)	Home-based	14 full-time equivalent professionals, including teachers, speech-language pathologists, and audiologists<br><br>Program manager	Rehabilitation and therapy services<br><br>Early Intervention Program<br>School age program<br>Group sessions<br>Preschool/school site visits<br>Audiological assessments

				Administrative/technical assistant<br>Toy librarian<br>Information technology<br>Occupational therapists<br>Audiologists<br>Psychologists	In-person sessions with RIDBC professionals
A132012	A mobile telemedicine-enabled ear screening service for Indigenous children in Queensland: activity and outcomes in the first three years	Smith, Armfield, Wu, Brown, & Perry (2012)	Mobile facilities	Local Indigenous health worker	Patient information and clinical history<br>Digital images using video-otoscope<br>Hearing assessment<br>Health risk questionnaire<br>ENT Review
A142013	Changes in paediatric hospital ENT service utilisation following the implementation of a mobile, indigenous health screening service	Smith et al. (2013)	Mobile facilities	Local Indigenous health worker	Patient information and clinical history<br>Digital images using video-otoscope<br>Hearing assessment<br>Health risk questionnaire<br>ENT Review
A152014	Listening and Learning: Using Telepractice to Serve Children and Adults with Hearing Loss	Galvan, Case, & Todd Houston (2014)	Home-based	Two Listening & Spoken Language Specialists & Certified Auditory Verbal Therapists (LSLS Cert. AVTs)	AV Therapy: Listening and language skills<br>Adult Aural Rehabilitation
A162015	Tele-audiology: Expanding access to hearing care and enhancing patient connectivity	Gladden, Beck, & Chandler (2015)	Community clinics	Trained assistant<br>Teleprovider<br>Telepresenter	Remote Hearing aid programming<br>Remote Audiometry<br>PC-sharing application<br>Remote programming of hearing aids - Smartphone app<br>Automated audiometry<br>Remote Cochlear Implant Mapping

A172015	Monitoring ear health through a telemedicine-supported health screening service in Queensland	Smith et al. (2015)	Mobile facilities	Local Indigenous health worker	Patient information and clinical history<br>Digital images using video-otoscope<br>Hearing assessment<br>Health risk questionnaire<br>ENT Review
A182016	Reducing Loss to Follow-Up with Tele-audiology Diagnostic Evaluations.	Dharmar et al. (2016)	Hospital	Telepresenter	Patient history, visualization of external structures, video otoscopy, immittance (including high-frequency tympanometry and middle ear muscle reflexes), distortion product otoacoustic emissions, auditory brainstem response with air and bone conduction, and, when indicated, auditory steady-state response<br><br>All aspects conducted remotely<br><br>Parent/ Guardian Satisfaction Survey<br>Provider Survey
A192016	The Future of Healthcare Delivery : IPE / IPP Audiology and Nursing Student / Faculty Collaboration to Deliver Hearing Aids to Vulnerable Adults via Telehealth	Novak, Cantu, Zappler, Coco, & Champlin (2016)	Community clinic	Audiology students<br>Nursing students<br>Supervising faculty	Video otoscopy - nursing student in city 2<br>Survey - audiology student in city 1<br>Selection and fitting of hearing aids - nursing student in city 2<br>Remote programming of the hearing instruments - audiology student in city 1<br>Review the owner’s manual, educate the patient on hearing aid

For peer review only

					parts, battery type/insertion/removal, and use of the push button volume control - nursing student in city 2<br>Real ear probe microphone measurements - audiology student in city 1
A202016	Infant Diagnostic Evaluation via Teleaudiology Following Newborn Screening in Eastern North Carolina	Stuart (2016)	Birthing centers	Remote Audiologist/ Technician<br>Clinical Telehealth Manager	Initial communication by call - option of Telemedicine or nearest diagnostic centre given<br><br>The diagnostic protocol:<br>Patient history<br>Cursory otoscopy<br>Middle-ear analysis<br>Distortion product otoacoustic emissions (DPOAEs)<br>Auditory brainstem response (ABR).
A212018	Cost and outcome of a community-based paediatric hearing screening programme in rural India with application of tele-audiology for follow-up diagnostic hearing assessment	Ramkumar et al. (2018)	Community clinic	Technician, Village health workers	DPOAE by VHW<br>Tele-ABR, in-person ABR
A222018	Comprehensive approach to the National Network of Teleaudiology in World Hearing Center in Kajetany, Poland	Skarżyński, Świerniak, Bruski, Ludwikowski, & Skarżyński (2018)	Community clinic	A monitoring clinician<br>A clinic engineer<br>Support speech specialist	Tele-consultation procedures<br><br>Remote fitting /Telefitting: ART/NTR/NRI, eSRT<br>Programming<br><br>Telerehabilitation
A232019	Hearing Norton Sound: Mixed methods protocol of a community	Emmett, Robler, Gallo, et al. (2019)	School-based	Local Clinic staff<br>Teachers	Focus groups and community events held leading up to the

For peer review only

	randomised trial to address childhood hearing loss in rural Alaska			Audiologist<br>ENT Specialist	randomised trial<br>Qualitative, semi-structured interviews to elicit stakeholder perspectives on the intervention<br><br>Hearing screening day:<br>School screen<br>mHealth screen<br>Audiometric assessment<br>HEAR-QL<br>Outcomes measured on screening day:<br>Sensitivity and specificity of screening<br>Hearing loss prevalence<br>Hearing-related quality of life (HEAR-QL)
A242019	Hearing Norton Sound: a community randomised trial protocol to address childhood hearing loss in rural Alaska.	Emmett, Robler, Wang, et al. (2019)	School-based	Local Clinic staff<br>Teachers<br>Audiologist<br>ENT Specialist	Focus groups and community events held leading up to the randomised trial<br>Qualitative, semi-structured interviews to elicit stakeholder perspectives on the intervention (explanatory sequential stage<br><br>Hearing screening day:<br>School screen<br>mHealth screen<br>Audiometric assessment<br>HEAR-QL<br><br>Outcomes measured on screening day:<br>Sensitivity and specificity of screening

For peer review only

					Hearing loss prevalence<br>Hearing-related quality of life (HEAR-QL)
A252019	Comprehensive Community Screening of Otological Patients by Trained Technicians Using a Telemedicine Device: An Efficient and Cost-Effective Way to Triage Patients With Ear Diseases	Gupta et al. (2019)	Construction sites	Trained community health care workers	Screening, diagnosis, medical management, surgical intervention, and rehabilitation using hearing aid.<br><br>Video-otoscopy using ENTraview smartphone otoscopy device<br>Air conduction threshold screening at 4 speech frequencies: 500 Hz, 1000 Hz, 2000 Hz, and 4000 Hz.
A262019	Telehealth-enabled auditory brainstem response testing for infants living in rural communities: the British Columbia Early Hearing Program experience	Hatton, Rowlandson, Beers, & Small (2019)	Community clinics	An audiologist, audiometric technician (established hearing screeners), and a telehealth project manager	AC and BC ABR<br><br>Optional<br>Tympanometry testing with a 1000-Hz probe tone in case of elevated ABR thresholds<br>OAEs in case of SNHL<br><br>TH-ABR programme evaluation<br>Cost and time effectiveness<br>Measure of ABR test efficiency<br>Parent satisfaction
A272019	Implementation and evaluation of a rural community-based pediatric hearing screening program integrating in-person and tele-diagnostic auditory brainstem response (ABR)	Ramkumar, Nagarajan, Shankarnarayan, Kumaravelu, & Hall (2019)	Community clinic	Tele-technician<br>Village health workers	DPOAE by VHW<br>Tele-ABR, in-person ABR

For peer review only

A282019	Telefitting Between Kajetany, Poland and Odessa, Ukraine for Cochlear Implants	Skarżyński, Swierniak, Ludwikowski, & Bruski (2019)	Community clinic	A monitoring clinician<br>A clinic engineer<br>Support speech specialist	Remote fitting /Telefitting:<br>ART/NTR/NRI, eSRT<br>Programming
A292020	Cochlear Implant Mapping Through Telemedicine—A Feasibility Study	Luryi et al. (2020)	Community clinic	Local trained audiologist	Routine surveillance<br>Regular speech perception testing with AzBio sentence lists
A302020	Digital Proficiency Is Not a Significant Barrier for Taking Up Hearing Services With a Hybrid Online and Face-to-Face Model	Ratanjee-Vanmali, Swanepoel, & Laplante-Lévesque (2020)	Virtual clinic, home or office, or a satellite site for the clinic	Clinic Audiologist	Online Hearing Screening Test<br>Measures of Readiness and Stages of Change<br>Mobile Device and Computer Proficiency Questionnaires<br>Pure-Tone Average—Better Ear
A312020	Tele-Audiological Surveillance of Middle Ear Status among Individuals with Cleft Lip and/or Palate in Rural South India	Ravi et al. (2020)	Home-based<br>Camp based	Community workers<br>ENT consultant<br>Audiologist	Video-otoscopy<br>Pure tone audiometry<br>Tympanometry
A322020	Hearing Norton Sound: community involvement in the design of a mixed methods community randomized trial in 15 Alaska Native communities	Robler et al. (2020)	School-based	Local Clinic staff<br>Teachers<br>Audiologist<br>ENT Specialist	Focus groups and community events held leading up to the randomised trial<br>Qualitative, semi-structured interviews to elicit stakeholder perspectives on the intervention (explanatory sequential stage<br><br>Hearing screening day:<br>School screen<br>mHealth screen<br>Audiometric assessment<br>HEAR-QL<br>Outcomes measured on screening day:<br>Sensitivity and specificity of screening

For peer review only

					Hearing loss prevalence<br>Hearing-related quality of life (HEAR-QL)

For peer review only

Supplemental File – 9

Table 2: Reference quotes from the semi-structured interviews

**Acceptability**	
1.	85% *(of the patients)* reported that they were very satisfied. Around 12% reported that they were satisfied. Some of them were not satisfied, but when we analyzed (*patient perspectives)* deeper, we found that this was reported only at the beginning. They wanted to have the first session on-site, at the centre, which we did. Then we found that the satisfaction level was over 95%.<br><br>- Project implementer of an ongoing telepractice service delivery program in Poland
2.	Parents were the best ambassadors of the idea when they saw that the results from the rehabilitation service were the best. So they were convinced of the method.<br><br>- Project implementer of a community-based telepractice service delivery program in India
3.	We have some testimony of parents saying, "This is awesome and it's motivational and it's informational. And I love that I didn't have to take my kid to *abc (referring to a distant location)* and get evaluated, then travel back and then travel down for surgery and travel back; I love that I could use this technology. I didn't have to travel or leave my community and my child is getting quick care and being seen." And so we had a lot of positive reception around the technology in terms of the trial. Also, when we asked parents, "Do you want to be present for this follow-up?". The idea was to try and make it very quick and not make it a scheduled appointment - not pull parents out of work to get it done. Like they've referred and then they got to the clinic and they got seen within a five-minute appointment. And so we had a lot of parents who said, "Yes, do it because it's hard for me. I got multiple kids at home or I'm working. And so that really helps me to make sure my kid gets seen."<br><br>- Project implementer of an ongoing telepractice service delivery program in the US
4.	We felt it was essential to really capture narratives of the experience that we were investigating from community members and various stakeholders. One of the strengths of the trial was that we built a stakeholder group right away. There were multiple stakeholders on the ground, including individuals like myself who have served the region for a long time and who have become trusted members of that region. We interviewed teachers, special education teachers, school, administrators, hospital administrators, and community health workers, who are basically the on-the-ground eyes and ears in local community clinics - Parents and children, youth, elders in the community…… And so for us, that's been really helpful. We've also been working with the school district on the healthcare system to better refine the

For peer review only

	collaboration that's kind of required, to get this implemented without the research arm pushing it forward.<br><br>- Project implementer of an ongoing telepractice service delivery program in the US
5.	People were initially saying no. Then, initially, we did a lot of awareness activities. Wherever we planned to start the screening, for one week we put up posters, banners, and awareness activities. We went to the community and started with community dramas, for awareness, because nobody was ready to get screenings done. There were new dramas and other community activities done. There were 10- 20 days of awareness campaigns done so that they *(patients)* get convinced about getting the screening done because that behaviour has to be changed. The people over there need to understand what we are trying to do and what benefit they would get out of it. Then, we initiated these screenings door-to-door. Some people agreed based on that and more people followed. That's when screening happened.<br><br>- Project implementer of a community-based telepractice service delivery program in India
6.	So the people are seeing the mobile phone screen. So they want to know what their tympanic membrane looks like. So everybody began to say, "sir, please do it for me". Maybe in future, we can have visual feedback or a video about hearing and oral hygiene.<br><br>- Project implementer of an ongoing community-based telepractice service delivery program in South India
7.	We know that some of what we've done is not translatable to the general United States or even globally, but the *def (referring to the program)* is really a unique entity in that, healthcare is something that they are provided because their land has become part of the United States. So they receive *def* resources, and they had that as part of a single-payer system. So there was no cost to the parent or the end-user in terms of receiving this follow-up where potentially in another part of the United States this would potentially be something that would be billed to insurance or be a cost to a parent to receive.<br><br>- Project implementer of an ongoing telepractice service delivery program in the US
8.	One thing which we realized was that when you go to camps *(to conduct video-otoscopy)*, the patient expects something free of cost. So sometimes we ask the doctor if they can provide something in the camp. So ENTs always usually carry some kind of medication. Whatever is the medical treatment, the patients get it in the ground itself which is free of cost, and that also motivates them to come to the hospital.

For peer review only

	- Project implementer of a community-based telepractice service delivery program in India
9.	We have to do a better job of educating the patients and the consumers, the families that are accessing these services. We try to go through a lot of that orientation …... Making sure that we're setting appropriate expectations for the family that we're working with is very important.<br><br>- Project implementer of a long term telepractice service delivery program in the US
**Adoption**	
10.	Patients with some neurological issues, some rare diseases, are more demanding and in general, it is more difficult in terms of rehabilitation. So we try to help such patients on-site, in the clinic. For example, patients with mental disorders and after *(sustaining)* some trauma. If we have some risk group, we say, "No, sorry but you cannot use the telemedicine solution because the results would be not optimal."<br><br>- Project implementer of an ongoing telepractice service delivery program in Poland
11.	It's a lot of change management, which means you need the leadership teams and those clinics organizations and private practices to be wanting to change. It is a mind shift. It's the leadership team driving the vision to understand that you're not just adding technology to a patient journey. You're using technology to assist the patient and patient journey. You're not automating things for the sake of automating things. You're automating things so that the face-to-face appointments with the patient become a more enhanced conversation. For this model to be integrated into routine service delivery, each one can integrate the way that suits their context. But a mindset change and some amount of training to be able to make decisions on what might work best in terms of which components should be integrated are necessary.<br><br>- Project implementer of a hybrid telepractice service delivery program in South Africa
12.	Having the support of your institution or your hospital is critical - you need to know what you're doing so that they can get behind it. It can also be a way to reach the community, to get funding, to do other things - to demonstrate how you're serving the patient and the families that you're working with, in all of these different ways. So, the administrators certainly signed off on it; they saw the value in providing services to the families and the adults but also how it was going to enhance the training of the graduate students. I mean, that's our primary goal at the University - to teach and train the graduate students, so that when they leave they can become speech pathologists or audiologists. And so, it met all of these criteria - teaching, research and service

For peer review only

	delivery- these are the three areas that most universities are most concerned about. So we're able to demonstrate how having telepractice and having that lab satisfied all those areas.<br><br>- Project implementer of a long term telepractice service delivery program in the US
13.	The most interesting learning for me is the hesitancy of audiologists to embrace the model. I think that for me has been the most surprising, even through a COVID year. I think the audiologist's resistance and scepticism of such a model is interesting and maybe that's due to the fear of change. By no means, does the model remove the audiologist or remove the value of the audiologist; it is using technology to enhance the audiologist's value because you can take the conversation to a different level. You can use the time more efficiently.<br><br>- Project implementer of a hybrid telepractice service delivery program in South Africa
**Feasibility**	
14.	A lot of it has to do with management; there is planning, proper performance and looking for funds and adjusting the project according to the funds…….. I will advise future telepractice implementers to analyze the needs and the target group and to check in with the team which will be dedicated to the project, several times. Because there could be some gaps and misunderstandings, which could influence a non-successful project.<br><br>- Project implementer of an ongoing telepractice service delivery program in Poland
15.	This is a region that has a lot of middle ear diseases. We had to work with a school. We had to go to every school, we had to screen every child, and then the referral process. So, that was labour-intensive from a data collection standpoint for the study. Community health workers would say that "I don't want to do telemedicine because it's double the work. And, I'd rather just write it all down and then talk to a doctor on the phone." But what we see is that we don't always get the right diagnosis when that happens.<br><br>- Project implementer of an ongoing telepractice service delivery program in the US
16.	They (*technical and support staff)* try to work, they try to develop themselves and they earn money. I think salary is the first incentive and the second is the possibility of development……. So they work in something, which is a little bit unique. Of course, the money factor is important. Mostly, the incentive is looking at results. And

For peer review only

	the pay is also a good motivator.<br><br>- Project implementer of an ongoing telepractice service delivery program in Poland
17.	It's important to get that local connection. So that the patient, whenever he has some issues or some queries, can go directly to that person and get that sorted - that local connect is really important. It's also really important that they (*local liaison)* are able to put that commitment to work towards the community because there are very less people who want to work in communities and rural areas.<br><br>- Project implementer of a community-based telepractice service delivery program in India
18.	There were other individuals that we had brought in the stakeholder team; several native individuals from the community who served as our community outreach specialists. So she *(a community outreach specialist)* took a lot of what we were trying to say and created infographics to explain the trial in a way that through visuals and narrative. And so, I think that a lot of that helped us to gain trust….. I think we didn't experience as many cultural barriers because we went in the correct way. We identified our stakeholders right away; we used individuals who were from the region at the very beginning of the formation of the research question, and also while moving forward. And that really helps just kind of have it blend in seamlessly. Having enough focus groups in the beginning, generating awareness, bringing insight into the design of the trial. And a documentary narrative really helped us in getting the story that different stakeholders are telling…. I didn't see a lot of cultural barriers. But I think that's because we brought those people in.<br><br>- Project implementer of an ongoing telepractice service delivery program in the US
19.	You need to have a team - not only consisting of doctors and engineers, but also speech therapists. So we talk to audiophonologists and a whole management team starting from nurses and people who are dedicated to patient service; they could even be students. And this is quite important - to have that organizational service. It can not work with only one or two specialists. So having a team who worked earlier in medical centres, who know how to cope with patients, and can be dedicated to such a project. And there is no must for them to have a medical higher graduation.<br><br>- Project implementer of an ongoing telepractice service delivery program in Poland
20.	In some instances, in some communities, it worked fabulously because the school and the clinic already do a lot of hand-in-hand care. Those communication channels were well developed and seamless.

For peer review only

	- Project implementer of an ongoing telepractice service delivery program in the US
21.	The program is unique and is growing. It was designed and came out of sheer need and it basically has local community members in a trained program. They complete some initial tests - to check if they have enough reading and basic math skills. And then once they're in the program, then it routes them through a series of certifications. And so there are five levels of community provider. So one, two, three, four, and five as a practitioner, and then they learn different skills along each way. And so they are so integrated into healthcare that it's unbelievable. I'm involved in the training of our onboarding audiologists. I give them several cultural materials to read. I want them to come in with a very humble sense of the culture. They go through a three-day orientation of the hospital, which also is pretty culturally intensive in terms of nativity and heritage. And then we do a lot of on-the-job training from a telemedicine perspective. So that's one of the first things they learn because we do so much of it.<br><br>- Project implementer of an ongoing telepractice service delivery program in the US
22.	The instrument has to be charged completely for hours together, continuously. We were able to use it only for a maximum of four hours continuously because it has to support the LED light source and the mobile phone. And also, it'll consume more power to transfer data to the cloud by using the network. So there was a challenge in using the instrument continuously. It was not very user friendly initially, but after some time we could adapt it after the initial phase. Also, the equipment had texts that were only in English. We requested the company to modify it into the local language so that the community-based health workers are able to also read. So these were the challenges we faced initially, these were the difficulties.<br><br>- Project implementer of an ongoing community-based telepractice service delivery in South India
23.	I think that maybe the one important thing is having connectivity and that's the biggest issue right now. There's still sort of blackout areas where there's just very little connectivity or very very slow connectivity and that makes telepractice sometimes impossible to do. Most families do have internet services to some degree, but we still have some families that do not have internet. And that's the biggest problem that we run into, but not the most frequent. To figure out how they can get access to the connectivity can be a challenge from time to time. That probably happens 1% of the time; 99% of the time, they do have connectivity.<br><br>- Project implementer of a long term telepractice service delivery program in the US

For peer review only

24.	Undersea cables and just the continued expanse of getting closer and closer to broadband in our region has been pretty incredible, and obviously essential for this type of stuff.<br><br>- Project implementer of an ongoing telepractice service delivery program in the US
25.	Because in remote areas, the internet is still not available sometimes, it's usually store and forward technology that's used because it's much better than waiting for the community workers to come back and do it. It helps us save battery to do much more screenings and operationally that's viable also.<br><br>- Project implementer of a community-based telepractice service delivery program in India
26.	We have to be available to where the patients are and what devices they're using and not what we want them to use. Being able to connect to what they already have is going to be critical and going forward anyone starting a telepractice or telehealth program needs to realize that and that's sort of one of the best practices.<br><br>- Project implementer of a long term telepractice service delivery program in the US
27.	What's unique about *yyy (referring to a state in the US)* is that we've depended upon telemedicine early on, and so unique to conducting this trial in rural *yyy (referring to a state in the US)* is that we were building upon an established network and an established infrastructure on telemedicine. And so the goal of the study was really to use those resources that were available to us so that we knew when it came time to a sustainable solution that will continue beyond the research it's possible. We tapped into already existing infrastructure that people understand and know how to use, which was an asset for us both from completion and also a sustainability point of view. We did tweak it significantly to make it feasible.<br><br>- Project implementer of an ongoing telepractice service delivery program in the US
28.	If it wasn't the patient themselves who had a phone, it was the significant other or the caretaker. So I think digital proficiency is not a barrier. I think that's the assumption we have as audiologists, but there's always a support network around. Not always, but most of the time, there's a support network around the patient where technology is available.<br><br>- Project implementer of a hybrid telepractice service delivery program in South Africa

For peer review only

29.	I think we have about seven states that have passed this legislature of COMPACT now and I think when we get to *zzz (referring to a district in the US)*, then the COMPACT will go into effect. If you are in your home state and you're licensed in one of those states as a member of the COMPACT, then you can practice in any of the other states that are in the COMPACT. Though it is not an answer for everyone yet, these kinds of ideas have been out there and people have been trying to push those at the state level to get some of the legislation passed.<br><br>- Project implementer of a long term telepractice service delivery program in the US
30.	There are about eight different bills in the US Congress to make some changes permanent; to have more access to telehealth and telepractice and to have reimbursement available. So hopefully, one positive thing that may come out of this pandemic is the availability of telehealth and telepractice, but also the reimbursement that would be there for the providers to be able to provide these services.<br><br>- Project implementer of a long term telepractice service delivery program in the US
**<u>Implementation Cost</u>**	
31.	There was an initial investment cost and we bought high-end digital audiometers, we had a website etc. But it was the funding that allowed us to start a digital-first company. So, those costs come into play if you're starting from the ground up.<br><br>- Project implementer of a hybrid telepractice service delivery program in South Africa
32.	After an issue, an instrument got repaired and we didn't get funds to repair the instrument immediately. So suddenly, the process of intervention for hearing disorders stopped in the project. After some time, we got the funds, and we were able to procure new equipment.<br><br>- Project implementer of an ongoing community-based telepractice service delivery program in South India
33.	So Medicaid in *xxx (referring to a state in the US)* did not reimburse telepractice. The only time that they would reimburse for telepractice was if the SLP or whoever was delivering the services to a public school district and working with a child who was on Medicaid and receiving those funds. And so, no other funding of telepractice before Covid.<br><br>- Project implementer of a long term telepractice service delivery program in the US

For peer review only

34.	(In) the beginning, funds for the National Network of Teleaudiology were partially funded by the Norwegian funds …… for European Union. So in the beginning, it was from them. Some of the local projects are co-financed through different scientific grants. Always, we need to have our internal (financial) part, which we need to finance from our sources, which come from the services (we provide). ………. and some of them it's on a rental basis. So we don't buy flat, but we rent …. equipment…. Really, it's not easy.<br><br>- Project implementer of an ongoing telepractice service delivery program in Poland
35.	The biggest challenge was some blockage from the local insurance government, which didn't want to give us more financial support. So we have had limitations concerning that and we still have them sometimes. So, in such cases, we need to tell patients that they need to choose a private solution that they need to pay for. So it's not very comfortable for them either.<br><br>- Project implementer of an ongoing telepractice service delivery program in Poland
36.	I worked hard with all the stakeholders from the hospital to ensure that that encounter was billable, because if you're doing something that's not billable from a hospital perspective, then it's not sustainable.<br><br>- Project implementer of an ongoing telepractice service delivery program in the US

For peer review only

BMJ Open

Implementation Factors Influencing the Sustained Provision of Tele-Audiology Services: Insights from a Combined Methodology of Scoping Review and Qualitative Semi-structured Interviews

Journal:	*BMJ Open*
Manuscript ID	bmjopen-2023-075430.R1
Article Type:	Original research
Date Submitted by the Author:	20-Sep-2023
Complete List of Authors:	Ramkumar, Vidya; Sri Ramachandra Institute of Higher Education and Research (Deemed to be University), Department of Audiology<br>Shankar, Varsha; Sri Ramachandra Institute of Higher Education and Research (Deemed to be University), Department of Audiology<br>Kumar, Shuba; Samarth NGO, Department of Social Sciences
**Primary Subject Heading**:	Health services research
Secondary Subject Heading:	Ear, nose and throat/otolaryngology, Evidence based practice
Keywords:	Audiology < OTOLARYNGOLOGY, QUALITATIVE RESEARCH, Telemedicine < BIOTECHNOLOGY & BIOINFORMATICS, Feasibility Studies, COVID-19, Information technology < BIOTECHNOLOGY & BIOINFORMATICS

SCHOLARONE™
Manuscripts

I, the Submitting Author has the right to grant and does grant on behalf of all authors of the Work (as defined in the below author licence), an exclusive licence and/or a non-exclusive licence for contributions from authors who are: i) UK Crown employees; ii) where BMJ has agreed a CC-BY licence shall apply, and/or iii) in accordance with the terms applicable for US Federal Government officers or employees acting as part of their official duties; on a worldwide, perpetual, irrevocable, royalty-free basis to BMJ Publishing Group Ltd ("BMJ") its licensees and where the relevant Journal is co-owned by BMJ to the co-owners of the Journal, to publish the Work in this journal and any other BMJ products and to exploit all rights, as set out in our licence.

The Submitting Author accepts and understands that any supply made under these terms is made by BMJ to the Submitting Author unless you are acting as an employee on behalf of your employer or a postgraduate student of an affiliated institution which is paying any applicable article publishing charge ("APC") for Open Access articles. Where the Submitting Author wishes to make the Work available on an Open Access basis (and intends to pay the relevant APC), the terms of reuse of such Open Access shall be governed by a Creative Commons licence – details of these licences and which Creative Commons licence will apply to this Work are set out in our licence referred to above.

Other than as permitted in any relevant BMJ Author's Self Archiving Policies, I confirm this Work has not been accepted for publication elsewhere, is not being considered for publication elsewhere and does not duplicate material already published. I confirm all authors consent to publication of this Work and authorise the granting of this licence.

For peer review only

Implementation Factors Influencing the Sustained Provision of Tele-Audiology Services: Insights from a Combined Methodology of Scoping Review and Qualitative Semi-structured Interviews

Vidya Ramkumar*, Varsha Shankar, Shuba Kumar*****

* Professor, Department of Audiology, DBT - Wellcome Trust India Alliance, Intermediate Fellow in Clinical and Public Health Research, Sri Ramachandra Faculty of Audiology and Speech Language Pathology, Sri Ramachandra Institute of Higher Education and Research (DU), Chennai - 600 116, Tamil Nadu, India

vidya.ramkumar@sriramachandra.edu.in

**PhD Research Scholar, Department of Audiology, Sri Ramachandra Faculty of Audiology and Speech Language Pathology, Sri Ramachandra Institute of Higher Education and Research (DU), Chennai - 600 116, Tamil Nadu, India

varsha.shankar1997@sriramachandra.edu.in

***SAMARTH, Chennai, Tamil Nadu 600004, India

shubakumar@samarthngo.org

Corresponding author:

Dr. Vidya Ramkumar

Professor, Department of Audiology

DBT/Wellcome Trust India Alliance

Intermediate Fellow in Clinical and Public Health Research

Sri Ramachandra Faculty of Audiology and Speech Language Pathology

vidya.ramkumar@sriramachandra.edu.in

For peer review only

ABSTRACT

Objectives: The objectives of the current study were to (a) identify long-term tele-audiology services reported to be implemented beyond the research phase and determine whether they are sustained, (b) map the implementation process to StARI guidelines, and (c) map the factors that influenced its sustainability to the Implementation Outcomes Framework (IOF) to understand the gaps from an implementation research perspective.

Study design, setting and participants: This cross-sectional study included a scoping review of articles describing long-term tele-audiology services from around the world to determine the factors influencing the implementation. Six electronic databases (PubMed, Cochrane Library, Web of Science, Scopus, Google Scholar and ProQuest) were searched for literature published between 2010 and 2023. This was followed by semi-structured interviews (SSIs) which were guided by the IOF. Six project implementers were interviewed to obtain an in-depth understanding of factors that influenced sustainability of these tele-audiology services. Thematic analysis of the interview transcripts was carried out using a hybrid inductive-deductive approach.

Results: Data was extracted from 32 tele-audiology studies included in the review, which were then mapped to 21 projects. The findings of the scoping review reveal that tele-audiology services were predominantly provided using synchronous telepractice methods. The '*professional-facilitator-patient'* model was most commonly used. None of the studies reported the use of implementation research and/or outcome frameworks. Factors that influenced sustainability of tele-audiology services were identified from the combined results of the scoping review and the SSIs. These factors could be mapped to implementation outcomes of *acceptability*, *adoption, feasibility*, *implementation cost* and *sustainability*.

Conclusion: Implementation research and/or outcome framework should be used to guide the implementation processes, its evaluation and measurement of outcomes systematically in tele-audiology service delivery. When such frameworks are utilized, gaps in information regarding the context influencing implementation, reporting of fidelity, and adaptability measures can be addressed.

Keywords:

For peer review only

implementation research, implementation outcomes, tele-audiology, scoping review, semi-structured interviews, factors

Strengths and Limitations of the study:

- This study is guided by appropriate guidelines e.g., (PRISMA-ScR, COREQ, StaRI).
- Appropriate frameworks were used to guide the scoping review (population-concept-context) and the SSIs (IOF).
- Relevant data analysis methods (Thematic Analytical Approach, A Priori Thematic Saturation model, Hybrid inductive-deductive approach) were used for the SSIs.
- The limitations of the current study include the lack of a quality appraisal process.
- The SSIs were conducted on only few implementers who were able to participate in the study.

For peer review only

For peer review only - http://bmjopen.bmj.com/site/about/guidelines.xhtml

INTRODUCTION

Tele-audiology refers to the provision of audiology services using Information Communication Technology (ICT). Tele-audiology has the potential to address the gaps between demand and needs, especially where access to specialized services is limited (1,2). Tele-audiology has been explored over the last two decades for screening (3,4), diagnosis (5–7), and rehabilitation (8,9). The computerization of audiology equipment has further expanded the applications of tele-audiology (10). More recently, web portals and databases (11,12) have been developed to improve documentation and follow-up services in ear and hearing care. Tele-audiology can be conducted using synchronous mode, asynchronous mode, remote monitoring and mobile applications (13). Several systematic and scoping reviews have examined telepractice applications for ear and hearing disorders and the role of patient-site facilitators in tele-audiology service delivery (14–18).

However, despite substantial research in tele-audiology, there is a gap between the positive research outcomes and its sustainable implementation in the real-world setting. Integration of evidence-based interventions into routine clinical and community settings requires adoption of suitable implementation strategies, which forms the foundation of implementation research (19–23). Further, in order to achieve sustainability, outcomes suitable to the stage of implementation must be periodically monitored to make suitable adaptations (22). Implementation research and outcome frameworks help achieve translation of research evidence to sustained implementation (24). For example, the Implementation Outcomes Framework provides a model of eight conceptually distinct implementation outcomes (22). However, in general, implementation of allied health services has not been guided by implementation research (25).

Particularly with regard to tele-audiology services, there is a lack of data on sustained implementation efforts and the factors that influenced their sustainability. As a result, the current study's objectives were to(a) identify long-term tele-audiology services reported to be implemented beyond the research phase and determine whether they are sustained, (b) map the implementation process to StARI guidelines, and (c) map the factors that influenced its sustainability to Implementation Outcomes Framework (IOF) (22) to understand the gaps that must be addressed from an implementation research perspective.

For peer review only - http://bmjopen.bmj.com/site/about/guidelines.xhtml

METHODS

Ethical clearance was obtained from the Sri Ramachandra Institute of Higher Education and Research (DU) Institutional Ethics Committee (Reference Number: CSP/20/NOV/87/191) for this study.

Patient and Public Involvement: Patients or the public were not involved in the design, or conduct, or reporting, or dissemination plans in the current study.

This study included a scoping review followed by a qualitative component involving semi-structured interviews. The scoping review was carried out to identify long-term tele-audiology services reported to be implemented beyond the research phase. The scoping review was also used to map the implementation process to StARI guidelines. Semi-structured interviews (SSIs) were used to determine whether or not these services are sustained. The findings from the scoping review and the SSIs were used to map the factors that influenced its sustainability to IOF (22).

Scoping Review

The Preferred Reporting Items for Systematic reviews and Meta-Analyses extension for Scoping Reviews guidelines (PRISMA-ScR) (26) was used to develop the scoping review protocol (supplemental file 1). The Population-Context-Concept framework was used to formulate the eligibility criteria which included search terms, strategies, quality appraisal criteria and extraction methods. Search strategies were finalised after an initial pilot was carried out (supplemental file 2). An initial decision profile (supplemental file 3) was designed and created for systematic documentation of the selection of articles.

Six electronic databases (PubMed, Cochrane Library, Web of Science, Scopus, Google Scholar and ProQuest) were searched. The Rayyan software (27) was utilised for duplicate removal and article selection process. Two reviewers were involved during this entire process, and any disagreements between the two were discussed until a consensus was established.

Studies describing tele-audiology service provision (including screening, diagnostic, or rehabilitative services) to individuals of all age groups, based on prior research evidence were included. Studies describing

For peer review only

"long-term" or "routine" or "sustained" tele-audiology service delivery, for two or more years, were only included. Quasi-experimental trials, and community or field trials were also included. All telepractice modalities, including video conferencing, web-based, mobile applications, and remote computing, were considered. Only literature published in English between 2010 and 2023 was considered.

Studies that compared in-person and telepractice measures, or reported validity of tests or tools but did not report or mention long-term implementation were excluded. When full texts were not available after reasonable efforts, articles were excluded.

Analysis

The search results were summarised using the Preferred Reporting Items for Systematic Reviews and Meta-Analyses (PRISMA) flow diagram. We extracted information on geographical distribution, the focus area of service delivery, method and model of tele-audiology service delivery. The Standards for Reporting Implementation Studies (StaRI) guidelines (28) was used to map the implementation strategies and components reported in the studies (supplemental file 4). When a StARI criteria was addressed, it was denoted with a check mark, when it was partially addressed, it was denoted with a "?", and when it was not addressed, it was denoted with a "X." When a criterion was not relevant, it was marked with "N/A." The contents of the articles were examined for reports of factors that influenced the sustainability of tele-audiology services. Any recommendations by the implementers were also considered as influencing factors (supplemental file 5). These were then mapped to the constructs of the IOF (22).

Semi-structured interviews (SSI)

The long-term and sustained tele-audiology service implementers identified in the scoping review were included for the SSIs. This was done to assess if these services are still sustained and also exemplify the data obtained from the scoping review on the factors that influenced the sustainability of these services. The COREQ (COnsolidated criteria for REporting Qualitative research) guidelines (29) were used to report the qualitative components of the study (supplemental file 6).

For peer review only

Research team:

Personal characteristics and relationship with participants: Both VR and VS conducted three interviews. VR has 10 years of experience performing qualitative research; VS had no prior experience. There was no specific relationship established by the interviewers with the participants prior to the interview. Information about the interviewer's interest in the research topic, as well as purpose and description of the research study was provided in the e-consent form.

Study design

Theoretical framework and development of interview guides: The thematic analytical approach is useful to identify, analyse, and report patterns in the data obtained (30,31); thus, in order to better understand the factors that influenced the sustainability of the tele-audiology services across different implementers, we used this approach as our methodological orientation for this study. The interview guides were developed based on the constructs of the IOF (22) to which the influencing factors could be mapped to. It was then verified by two experts with experience in qualitative research and telepractice. The guides were verified for content, wording and relevancy to objectives. A copy of the interview guide has been provided (supplemental file 7).

Participant selection: Sampling frame included for the SSIs were the primary and corresponding authors of all the papers identified in the scoping review. The corresponding authors were first contacted; if they were unable to participate, we requested a referral to any of the other authors of the team. If we were unable to establish contact with the corresponding author, then an effort was made to contact other authors.

E-mails were sent to all the authors along with the link to an e-consent form. In a couple of instances, the authors led the investigators to another team member. Reminders were provided for scheduling the interviews and the consent form remained available for three months. Out of the 21 authors identified in the scoping review, six authors in their early, mid and advanced career levels, consented to participate in the study. The participant characteristics are described in table 1.

For peer review only - http://bmjopen.bmj.com/site/about/guidelines.xhtml

Table 1: Description of SSI participants

Code	Description of participant	Gender	Experience levels	Country
01	Project implementer of a long-term telepractice service delivery program	Male	Advanced	USA
02	Project implementer of an ongoing telepractice service delivery program	Female	Mid-career level	USA
03	Project implementer of an ongoing telepractice service delivery program	Male	Mid-career level	Poland
04	Project implementer of a community-based telepractice service delivery program	Female	Early-career level	India
05	Project implementer of a community-based telepractice service delivery program	Male	Early-career level	India
06	Project implementer of a hybrid telepractice service delivery program	Female	Early-career level	South Africa

3 *Setting:* Since interviewees belonged to different countries around the world, all interviews were
4 conducted using an encrypted online video-conferencing platform. Other than the investigators, no one else
5 was present during the interview. Interviews were carried out between March and August 2021.

6 *Data collection:* The interview guides were initially piloted on a project implementer and suitable
7 adaptations were made prior to data collection. Primary guides and additional probes were used to obtain rich
8 data. The interview guides were further informed and expanded upon based on each interview, thereby
9 enabling deeper insights.

10 Informed e-consent was obtained from all the participants prior to the interviews. All interviews
11 were audio-video recorded and field notes were also taken by the interviewers. All interviews were completed
12 within 30 to 45 minutes. The "*A Priori Thematic Saturation*" (32) model was used in which the adequacy of
13 data collected was determined when informational redundancy across interviews was apparent.

14 <u>*Analysis:*</u>

15 *Data Analysis:* We conducted thematic analysis of verbatim transcription of all the audio and
16 video recorded interviews using NVivo 12 software. Thematic analysis was carried out using a hybrid
17 inductive-deductive approach by reading through the transcripts and looking for patterns to identify themes
18 (31,33). After familiarisation with the transcripts, both the authors coded two transcripts independently and
19 updated the existing codebook by adding codes generated deductively from the interviews. Any coding
20 differences were discussed and resolved. Upon fine-tuning, the remaining transcripts were coded based on the

For peer review only - http://bmjopen.bmj.com/site/about/guidelines.xhtml

updated codebook. Unique and relevant information that were identified during this process were given new codes. We organised the codes based on similarity and frequency, which aided in categorising codes. We analysed these categories for important patterns in the data that were relevant to our study objectives and compiled them under themes. Following this analysis, we evaluated the themes for relevancy and credibility. We merged certain themes together for a more succinct representation of the findings whenever necessary.

Reporting: We presented an explanation of our study findings, which were substantiated with quotes placed under the respective themes with specific qualifiers for each participant. All quotes and findings were sent back to the interviewees for comments and corrections.

For peer review only

For peer review only - http://bmjopen.bmj.com/site/about/guidelines.xhtml

1 **RESULTS**

2 The results describing, project mapping, geographical distribution of projects, focus area of service delivery, method of telepractice, model of telepractice service delivery and mapping of implementation components to the StaRI guidelines are based on the findings from the scoping review. The section addressing the factors that influenced the sustainability of tele-audiology services include findings of both the scoping review and the SSIs.

7 The scoping review identified 32 articles that satisfied the inclusion criteria. The PRISMA flow diagram depicts the studies search results (Figure 1). A summary of the literature reviewed based on the scoping review's inclusion criteria was tabulated for analysis. The study code, study title, authors, countries, and focus area are all listed in Supplemental file 8 (table 1a). The settings, participants, employees, and types of services are described in Supplemental file 8 (table 1b).

12 ***Project mapping***

13 Studies describing similar background content in the introduction, common implementation sites, broad objectives, and a contiguous group of investigators were mapped as a single project. This exercise was undertaken to make a realistic assessment of the implementation focus. Studies were considered independent projects when they could not be mapped under any other larger project. Twenty-one projects were identified based on this mapping exercise (Table 2) and each project was coded as P (project) and serial number, for ease of reference. The subsequent findings will be reported using these project codes.

For peer review only - http://bmjopen.bmj.com/site/about/guidelines.xhtml

Table 2: Summary of project mapping in tele-audiology

Focus area	Country	Project	Project code
Hearing Screening	United States of America	Emmett et al. (2019)<br>Emmett et al. (2019)<br>Robler et al. (2020) (34–36)	P1
Diagnostic audiological evaluation	Canada	Dharmar et al. (2016) (37)	P2
		Stuart (2016) (38)	P3
	Canada	Campbell and Hyde (2010) (39)	P4
		Hatton et al. (2019a) (40)	P5
	India	Ramkumar et al. (2018a)<br>Ramkumar et al. (2019) (41,42)	P6
Diagnostic audiological with otorhinolaryngological evaluation	Canada	Hofstetter et al. (2010)<br>Kokesh, Ferguson, and Patricoski (2011) (43,44)	P7
	Australia	Smith et al. (2012)<br>Smith et al. (2013)<br>Smith et al. (2015) (45–47)	P8
	India	Gupta et al. (2020) (48)	P9
		Ravi et al. (2020) (49)	P10
Hearing aid fitting and programming	United States of America	Dennis, Gladden, and Noe (2012) (50)	P11
		Novak et al. (2016) (51)	P12
Cochlear implant fitting and mapping	United States of America	Luryi et al. (2020) (52)	P13
	Poland	Skarżyński et al. (2018)<br>Skarżyński et al. (2019) (53,54)	P14
Aural re/habilitation	United States of America	Houston (2011)<br>Blaiser et al. (2012)<br>Houston and Stredler-Brown (2012) (55–57)	P15
		Broekelmann (2012) (58)	P16
		Lalios (2012) (59)	P17
		Galvan, Case, and Todd Houston (2014) (60)	P18
	Australia	McCarthy, Muñoz, and White (2010)<br>McCarthy (2011)<br>McCarthy (2012) (61–63)	P19
Comprehensive audiological services	United States of America	Gladden, Beck, and Chandler (2015) (64)	P20
	South Africa	Ratanjee-Vanmali, Swanepoel, and Laplante-Lévesque (2020) (65)	P21

For peer review only - http://bmjopen.bmj.com/site/about/guidelines.xhtml

Geographical distribution of projects

Tele-audiology was implemented in 12 projects in the United States of America (USA); two projects in Australia; three projects in India; two projects in Canada; one project each from Poland and South Africa. Projects implemented in almost all the countries (P1-10; P14; P16; P18-20) were delivered to overcome access barriers for individuals in rural areas and/or remote locations. However, in the US there were six projects (P11-13; P15; P17; P21) that provided tele-audiology services to urban and semi-urban areas due to scheduling related issues (work, having other children to take care of, household responsibilities).

Focus area of tele-audiology service delivery

Tele-audiology was implemented in projects with a focus on ear and hearing screening, diagnostic audiological evaluations, a combination of diagnostic audiological with otorhinolaryngological evaluation, hearing aid fitting and programming, cochlear implant fitting and mapping as well as intervention services.

Mobile phone-based ear and hearing screening was carried out among school-aged children (P1). Telepractice was used to conduct diagnostic confirmation of hearing loss among infants in follow-up to hearing screening (P2-6). Tele-auditory brainstem response (ABR) and tele-distortion product otoacoustic emissions (DPOAEs) were performed in all these projects. Other diagnostic tests such as immittance including high-frequency tympanometry and middle ear muscle reflexes, and Auditory Steady-State Response (ASSR) were included only in a few projects (P2, P3).

Diagnostic audiological evaluation combined with otorhinolaryngological evaluation using telepractice was reported in four projects (P7-10). In these projects, basic ear (video-otoscopy) and hearing testing (audiometry and tympanometry) was provided as a routine service to individuals of all age groups.

Remote hearing aid fitting, verification and programming (P11, P12) as well as remote cochlear implant switch-on and mapping (P14) were conducted for all age groups. One project focused on cochlear

For peer review only - http://bmjopen.bmj.com/site/about/guidelines.xhtml

implant mapping for geriatric patients alone (P13). In the projects where remote cochlear implant switch-on and mapping were conducted (P13, P14), tests such as acoustic reflex threshold/neural response telemetry/ neural response imaging and e-stapedial reflex threshold was carried out through telepractice. Telepractice was used for aural re/habilitation of both children and adults (P15-19) to provide auditory-verbal therapy or some form of habilitation training to individuals with hearing loss.

Comprehensive audiological services to individuals of different age groups were provided with tests including audiometry, hearing aid fitting and programming, cochlear implant fitting and mapping in one project (P20). On the other hand, another project (P21) conducted only hearing screening and hearing aid reprogramming using telepractice, for adults. The diagnostic confirmation and hearing aid fitting was done in person.

Method of telepractice

Use of synchronous/real-time, asynchronous/ store and forward, or a combination/ hybrid method for providing tele-audiology services was identified from the scoping review.

In tele-audiology, the asynchronous method of service delivery was predominantly used for audiometric screening, video-otoscopy, and tympanometry (P1; P7-10). Client details, video-otoscopic images, and audiometric or tympanometry data were stored and forwarded to an audiologist or otolaryngologist for review. For tele-diagnostic evaluation of hearing among infants, synchronous methods (for both ABR and DPOAEs) or hybrid methods of synchronous (for ABR) and asynchronous (for DPOAEs) were used (P3-6). In one project (P2) all diagnostic tests (video-otoscopy, middle ear analysis, DPOAEs, ABR and ASSR) were completed synchronously. Remote hearing aid fitting and programming (P11, P12, P20, P21) and remote cochlear implant fitting and mapping (P13, P14), were conducted synchronously. In one project (P12), even real-ear probe measures were conducted synchronously. Aural habilitation services like Auditory Verbal Therapy (AVT) were provided synchronously (P15-19).

For peer review only - http://bmjopen.bmj.com/site/about/guidelines.xhtml

Model of telepractice service delivery

When an e-helper/telepractice assistant/ support staff supported patient care, it was categorised as "*professional-facilitator-patient*' model; when the professional delivered the service directly to the patient without intermediary personnel, it was categorised as '*professional-patient*' model; and '*professional-professional-patient*' model involved a second audiologist or any other professional at the patient site.

In tele-audiology, irrespective of test procedures, the 'professional-facilitator-patient' model was used whenever services were provided to remote rural areas. In this case, a trained e-Helper/telepractice assistant/community-based health worker served as a facilitator for telepractice (P1-6; P8-12; and P20) and their roles were defined in the project. This was the most commonly used model of telepractice service delivery. The 'professional-patient' model was predominantly used for services provided through video-conferencing such as AVT and habilitation of individuals with hearing loss (P15-19; P21). The 'professional-professional-patient' model was used for fitting cochlear implants (P13, P14) when both audiological and otorhinolaryngology services were provided (P7). The professional at the patient's end was an audiologist with no specialised training in cochlear implant aural rehabilitation, while the professional who provided services via telepractice was specialised in this area (P13). In one project (P14), a speech therapist was the professional available at the patient end to facilitate the tele-fitting of cochlear implants. Otolaryngology consultations were supported at the patient end by a variety of professionals including audiologists, physicians, public health nurses, dentists, and physician assistants, as a part of a routine telemedicine clinic (P7).

Mapping of the implementation process to StaRI guidelines (28)

We found that none of the long-term projects used any implementation framework to implement tele-audiology service delivery. Thus, we mapped the implementation process described in the studies to the StaRI guidelines. Only two study titles (41,46) and six study abstracts (40–42,46,49,53) identified their study as "implementation" research. None of the studies mentioned the use of an underlying

For peer review only - http://bmjopen.bmj.com/site/about/guidelines.xhtml

theory/framework/model or relevant reporting guidelines. Eleven studies provided a detailed overview of the context influencing implementation (34–36,38–41,50,51,62,64).

The outcomes of the implementation strategy were specified in 14 studies (34–36,40,41,43–45,49–51,61,62,64). While 18 studies included process evaluation objectives (34–37,40,44,48,51–54,56–59,61,62,64), only 12 studies reported the process outcomes (40,44,48,51–54,57,59,61,62,64). The economic evaluation of the implementation strategy was described only in seven studies (40,41,43,44,49,50,66). Although description of fidelity to the planned implementation strategy and contextual adaptations was reported in four studies (36,40,48,64), specific measures to assess fidelity and adaptation were not described. Fourteen studies each addressed scalability (36,39,40,42,50,51,53,54,57,58,61–64) and sustainability (39,40,42,50–53,57–59,61–64)

Mapping of the influencing factors to the IOF (22)

The results of the scoping review (denoted using the project codes) and thematic analysis of the SSIs (substantiated using quotes) were combined to map the factors that influenced sustainability of tele-audiology services. The quotes are summarized (Supplemental file 9) under the respective overarching themes with specific qualifiers for each project implementer.

We found that none of the projects studied implementation outcomes systematically. However, studies highlighted the factors influencing the sustainability of tele-audiology service delivery based on their experiences. These reports could be mapped to the implementation outcomes of *acceptability, adoption, feasibility, implementation cost, and sustainability* (22).

Acceptability:

The beneficiary/ patient perception related factors reported by the project implementers could be mapped to the early to late-stage implementation outcome of *acceptability.* Parents were hesitant to adopt tele-audiology in the beginning as they were apprehensive about the child's ability to build rapport with the

For peer review only - http://bmjopen.bmj.com/site/about/guidelines.xhtml

therapist. In such a case, they were given a choice of a combined approach of in-person and telepractice sessions until they felt comfortable and confident (P5, P19) or continue in-person sessions till they expressed their willingness to engage in telepractice sessions (P15, P18). The families were provided with the opportunity to meet the therapist and participate in a practice video conference session before the commencement of telepractice sessions. Such a mindful approach was found to help address patient apprehensions (refer to quote 1). Parents also became ambassadors of the program when they saw the value in it (refer to quote 2).

Patients' or caregivers' perceptions and understanding of the advantages of tele-audiology service delivery was a key facilitator (P5, P7, P8, P14, P15, P19, P20) (refer to quote 3). Continuous and ongoing engagement with communities in a culturally safe manner and building awareness regarding the service enhanced acceptance (P1, P8) (refer to quotes 4 and 5). Reduced travel cost associated with tele-audiology services was perceived as facilitatory for implementing such services (P12, P14). Real-time presence of the professional, technical team and support staff and reassurance by them, facilitated the acceptance of tele-audiology by patients/ caregivers (P15, P18, P19-20). Patient's acceptance of technology use in ear and hearing care improved when visual images of tests conducted (image of tympanic membrane) were provided (refer to quote 6). Acceptance was also conditional to the absence of any direct cost to the patient (refer to quote 7) or in case of subsidised service (refer to quote 8). Patients felt secure when data security, privacy and confidentiality were protected (P5, P16). Participation in tele-audiology sessions by other family members, who otherwise would not be able to do so, was further reassuring in some instances (P15, P19). Setting appropriate expectations for the patient and ensuring clear communication prior to telepractice service delivery can enable acceptance (refer to quote 09).

Adoption:

Provider perspectives regarding uptake and utilization of tele-audiology service delivery could be mapped to the early-stage implementation outcome of *adoption*. Project implementers felt that the assessment of a patient's suitability for tele-audiology was crucial and it was important to clearly communicate to the patient

For peer review only - http://bmjopen.bmj.com/site/about/guidelines.xhtml

when telepractice might not be the best option (refer to quote 10). Adoption of new service delivery requires change and this change needs to be supported and driven by the leadership with a vision for sustainability (refer to quote 11). Organisations see value in telepractice because of their threefold advantage: teaching, research and service delivery (refer to quote 12). Hesitancy among service providers to adopt tele-audiology was considered as a challenge to implementation (refer to quote 13).

Feasibility:

Factors related to the utility and practicality of implementing tele-audiology service delivery could be mapped to the early-stage implementation outcome of *feasibility.* Assessing needs, availability of internal resources, financial planning and a service delivery model that is flexible and adaptable to change was considered useful for seamless implementation of tele-audiology services (P9, P20) (refer to quote 14). Formulating a Memorandum of Understanding (MOU) and a Telehealth Service Agreement (TSA) before implementation was reported to be facilitatory (P5, P11). Integrated electronic health records were also recommended to ease tele-audiology service implementation (P7, P20).

Limited or non-availability of professionals and technicians trained in tele-audiology service delivery was reported to be a major barrier to implementation (P15). Retention of personnel employed in tele-audiology service delivery was a challenge as it was perceived to be labour-intensive (refer to quote 15). The uniqueness of telepractice draws personnel to work in such services; however, continuity is supported by monetary benefit, opportunities for career development and positive outcomes of services (refer to quote 16). In providing remote care, establishing a local "point of contact" who is dedicated and committed to assisting telepractice service delivery was beneficial (refer to quote 17). Having a liaison from the community helped minimise and overcome cultural barriers (refer to quote 18). A dedicated team including professionals, and administrative and technical support staff was considered pertinent (P6, P19) (refer to quote 19). Reliable patient outcomes were associated with team-building, coordination and streamlined communication between all collaborators involved in telepractice service delivery (P5, P11, and P13) (refer to quote 20). Further,

For peer review only - http://bmjopen.bmj.com/site/about/guidelines.xhtml

defining the roles and responsibilities of e-helper/ telepractice assistants with systematic culture-sensitive training was considered useful (P7, P20) (refer to quote 21).

Devices with a simple user interface, minimal skill requirement, display in local language and adequate battery back-up were essential to support remote community-based tele-audiology services (refer to quote 22). Poor internet bandwidth compromised service delivery in rural areas (P10, P15, P17) (refer to quote 23) and the use of undersea cables and broadband connections were regarded as more reliable (P5, P19) (refer to quote 24). High quality dedicated video conferencing equipment was a quality enhancer in tele-audiology service delivery (P14, P19, P20). In rural areas where issues in connectivity were expected, the store and forward method of tele-audiology was found beneficial (refer to quote 25).

Capitalising on available resources (patient's own devices, pre-existing infrastructure) facilitated seamless implementation (refer to quotes 26 and 27). Contrary to popular belief, digital proficiency was not reported to be a significant barrier to implementation, as it was felt that a supporting mechanism can be created (refer to quote 28).

Lack of acts, laws, malpractice liability and credentialing issues were also some of the reported challenges to tele-audiology service implementation (P11). In certain countries where state licensing was essential, unique licensing policies to deliver telepractice services across states was required (refer to quote 29). Some of these legislative policies gained popularity only after the COVID-19 pandemic and project implementers felt that these changes need to be made permanent (refer to quote 30).

Implementation cost:

Factors related to cost of setting-up or cost associated with continued tele-audiology services could be mapped to *implementation cost* which is an early to late-stage implementation outcome. Higher initial capital investment to set up tele-audiology service delivery and the need for specialised equipment to support such practice was reported as a barrier to implementation (P6, P12, P15, and P20) and was only possible with adequate funding (refer to quote 31). When there was a pause or temporary issue with the release of funds, it

For peer review only - http://bmjopen.bmj.com/site/about/guidelines.xhtml

adversely affected service delivery (refer to quote 32). Even though tele-audiology has gained popularity and preference in the pandemic, it was not adequately supported financially by organisations in the past for routine service delivery (refer to quote 33). Multiple sources of funding including scientific grants, organisational funding, and charitable donations were required to ensure continuity of service (P19, P20) (refer to quote 34). On some occasions, lack of insurance reimbursement for tele-audiology services was a barrier (P11) (refer to quote 35).

When long-term cost outcomes or cost benefits of tele-audiology were evaluated, they found it to be a beneficial alternative from a societal perspective (P10, P15). Considerable efforts were taken to establish and self-sustain billable tele-audiology service delivery and these were very few (P21) (refer to quote 36).

Sustainability:

Factors reporting the maintenance of tele-audiology service delivery were mapped to the late-stage implementation outcome of *sustainability* and these were minimal. Out of the identified projects in tele-audiology, 14 projects (P1-5, P7, P9-11, P13, P14, P18-20) reported service delivery to be ongoing or routine. The rest, even though long-term, were still in the research phase. Tele-audiology services were implemented in a more sustained manner in hearing screening programs for infants. For example, follow-up diagnostic evaluations were conducted using telepractice in the USA and Canada (P2-5), ear and hearing screening among school children was conducted in the USA (P1) and diagnostic audiological with otorhinolaryngological evaluation for adults was conducted in the USA and in India (P7, P9, P10). The Veterans hospitals in the USA have implemented remote hearing-aid fitting and programming as well as cochlear implant fitting and mapping as a routine service (P11, P13, and P20). Another effort is the National Network of Teleaudiology in Poland for cochlear implant switch-on and mapping (P14). Aural rehabilitation has also been implemented in a sustained manner in both the USA and Australia (P18, P19). Systematic pre-implementation feasibility, test runs and validation of functional requirements were useful components of sustainable implementation (P9, P5, and P18). Using existing resources to ensure implementation even after the research phase was considered facilitatory (refer to quote 37). Continued support from the organisation

For peer review only - http://bmjopen.bmj.com/site/about/guidelines.xhtml

was considered crucial for sustainable telepractice service delivery (refer to quote 38). The lack of interlinking support structures across administrative health authorities was reported to be detrimental to sustainability (P5).

For peer review only

For peer review only - http://bmjopen.bmj.com/site/about/guidelines.xhtml

DISCUSSION

Through this study, we identified tele-audiology programs that provided long-term or sustained services for at least two years. We identified the factors that influenced their sustainability which we then mapped to the IOF (22) to understand the gaps that must be addressed for improved translation of research evidence to sustained implementation.

In general, we found that tele-audiology was predominantly implemented in remote/rural locations to overcome practical challenges associated with receiving in-person services. The most common application of tele-audiology was for diagnostic testing with ABR or OAE in infant hearing screening programs. This was done to reduce the loss to follow-up especially when hearing screening was conducted in primary health care clinics or rural communities (37,38,40,42). Aural re/habilitation for hearing loss was the next most common application, where only simple video-conferencing software was required. Remote hearing aid fitting and programming was minimally explored, possibly due to the limited availability of supportive features in products of a wider price range (67). There is a definite preference for using synchronous methods for conducting diagnostic tests or fitting hearing devices when audiologists' real-time judgement is more crucial (68). Whenever screening was done or images could be captured (like video-otoscopy or tympanometry), asynchronous methods were used. In such tests, real-time assessments by the professional were not considered to be critical for test completion. It was possible to provide telepractice services, such as aural re/habilitation, at homes using simple real-time videoconferencing which did not mandate specialised skills (69). In these cases, patient preparation was necessary and the parent/caregiver acted as a facilitator.

None of the studies included in the current review reported the use of an underlying implementation theory/framework/model and implementation outcomes were not studied systematically. More than half of the studies assessed process evaluation objectives, which were mostly related to patient satisfaction, but only a smaller subset reported its outcomes. In addition, only a few studies reported economic evaluations. We identified two projects (P1, P7) where tele-audiology services were provided as a part of comprehensive multi-specialty services. Impact evaluations of such implementations are likely to inform the value of capitalising

For peer review only - http://bmjopen.bmj.com/site/about/guidelines.xhtml

on common infrastructure and shared human resources. The core component of the intervention (tele-audiology service) to which fidelity is expected and those aspects that may be adapted must be systematically reported to aid implementation. But, none of the studies reported the use of fidelity or adaption measures.

While the use of implementation research and/or outcome frameworks for translating evidence-based practice to clinical service delivery is gaining momentum, it is still underutilized in tele-audiology service implementations. Only one recent study reported the use of implementation science framework to investigate the feasibility outcomes of a 17-week tele-audiology hearing aid service (70). Similar efforts for long-term implementation will offer deeper insights on sustainability aspects.

The factors reported to influence the sustainability of these services were mapped to the constructs of IOF. These factors were predominantly related to early-stage implementation outcomes of *feasibility* and early to late-stage implementation outcomes of *acceptability*. Limited factors could be mapped to the implementation outcomes of *adoption (early-stage), implementation cost (early to late stage)* and *sustainability (late stage).*

With reference to feasibility, internet access is a basic prerequisite for tele-audiology. The internet's speed and bandwidth affect various aspects of tele-audiology implementation, including test selection, technique and model of service delivery (71). The use of broadband connectivity and undersea cables was reported to be useful by implementers, yet there seems to be a continued challenge in maintaining robust connections for the purpose of tele-audiological tests in remote/rural locations (72). In addition, legislative policies and guidelines influenced feasibility of implementation considerably. Several countries introduced technical guidelines and changes in legislative policies for telepractice only during the COVID-19 pandemic (73). However, supportive mechanisms such as integrated electronic health records are still not widely available at a national level (74).

Acceptability and perceived demand for tele-audiology services by both the patients/ beneficiaries and the providers seems essential, as they are primary stakeholders involved. The findings of the current study indicate that better administrative convergence is required between health authorities for implementation of

For peer review only - http://bmjopen.bmj.com/site/about/guidelines.xhtml

tele-audiology services. The provider's willingness and comfort in adapting their services using technology is crucial for sustainability of tele-audiology service delivery (75).

For adoption of tele-audiology within organisational structures, supportive organisational policies that recognise it as an important component of service delivery is required (76). This will in-turn impact various organisational aspects including the availability of human resources, collaborations and effective planning. Tele-audiology is unlikely to be sustained without a clear plan that defines roles and responsibilities (77).

With reference to implementation cost, our findings suggests that even though tele-audiology services were initiated through research grants, several of them were able to integrate these services into public-funded programs. This suggests that sustained implementation occurred when the need and usefulness of tele-audiology were accepted by public sector policy-makers. If research projects include economic evaluation, then this can in turn inform the allocation of funds by organisations to support sustained implementation (78).

Factors related to appropriateness, *fidelity* and *penetration,* which are early, mid and late-stage implementation outcomes respectively, were not reported. Information regarding the context influencing implementation, the outcomes of the implementation strategy, process evaluation objectives, process outcomes, economic evaluation, specific measures to assess adaptation and scalability were not described. When appropriate implementation research and outcome frameworks are used, the stages or processes involved in achieving sustainability are less likely to be missed.

The strength of the current study lies in the representation of tele-audiology implementation efforts in a wide range of countries that include low-middle income (India and South Africa) and high-income (USA, Canada, Australia and Poland) nations. We found that the factors influencing the sustainability of tele-audiology service delivery reported in both High-Income Countries (HICs) and Low-Middle Income Countries (LMICs) were similar, except with respect to insurance coverage for tele-practice, and the availability of electronic or digital health records which emerged in the context of HICs and not LMICs. Thus, we believe that the findings of this study will be useful to tele-audiology project implementers from different regions.

For peer review only - http://bmjopen.bmj.com/site/about/guidelines.xhtml

There are certain limitations which could influence the scope of the current review. The study lacked a quality appraisal process and there is a possibility that articles published in non-native English-speaking languages could have been missed. Only six tele-audiology project implementers consented to participate in the SSIs, resulting in a small sample size.

For peer review only

For peer review only - http://bmjopen.bmj.com/site/about/guidelines.xhtml

List of abbreviations:

Information Communication Technology – ICT

Semi-structured Interview - SSI

Preferred Reporting Items for Systematic Reviews and Meta-Analyses - PRISMA

United States of America - USA

Distortion Product Otoacoustic Emissions - DPOAEs

Auditory Brainstem Response - ABR

Auditory Steady-State Response - ASSR

Memorandum of Understanding - MOU

Telehealth Service Agreement - TSA

Standards for Reporting Implementation Studies - StaRI

High-Income Countries - HICs

Low-Middle Income Countries - LMICs

For peer review only

For peer review only - http://bmjopen.bmj.com/site/about/guidelines.xhtml

ACKNOWLEDGMENTS:

Acknowledgements: We would like to thank all the researchers who consented to participate in the interviews sharing their valuable time for furthering the understanding in this area of research.

Competing interests: The Authors declare that there is no competing interest.

Funding: The authors disclosed receipt of the following financial support for the research, authorship, and/or publication of this article: This work was supported by the DBT/Wellcome Trust India Alliance Fellowship/Grant [IA/CPHI/19/1/504614] awarded to Vidya Ramkumar. The funders had no role in study design, data collection and analysis, decision to publish, or preparation of the manuscript.

Ethical Approval: Ethical clearance was obtained from the Institutional Ethics Committee (Reference Number: CSP/20/NOV/87/191) of Sri Ramachandra Institute of Higher Education and Research (DU) for this study.

Informed consent from participants: Informed written consent was obtained from all interview participants prior to the interviews.

Consent to publish statement: 'No objections' to publish the quotes from interviews was obtained from all participants.

Conflicts of Interest: The Authors declare that there is no conflict of interest.

Author Contribution: VR conceptualized the research topic, both VR and VS were involved in data curation, formal analysis, investigation, finalizing methodology, original draft preparation and reviewing & editing. VR was also integral in supervision, visualisation and acquisition of funding, resources and software. Both authors read and approved the final manuscript. SK was involved in methodology for coding, thematic analysis, and manuscript review and editing.

DATA AVAILABILITY STATEMENT:

For peer review only - http://bmjopen.bmj.com/site/about/guidelines.xhtml

The data that support the findings of this study are available on request from the corresponding author, VR. The data are not publicly available as the data underlying this study consists of transcripts of the qualitative interviews that identify the participants and cannot be sufficiently de-identified. To ensure confidentiality and data protection, we have only included relevant excerpts from the interviews with 'no objections' from the participants. We will only provide the transcripts to researchers/those that provide a proposal of how the data will be used by them. The corresponding author will ensure to remove identifiers from the particular data set and obtain 'no objections' from the participants before sharing the same.

For peer review only

References:

1. Waterworth CJ, Marella M, O'Donovan J, Bright T, Dowell R, Bhutta MF. Barriers to access to ear and hearing care services in low- and middle- income countries: A scoping review. https://doi.org/101080/1744169220222053734 [Internet]. 2022 Mar 23 [cited 2022 Jun 13];1–25. Available from: https://www.tandfonline.com/doi/abs/10.1080/17441692.2022.2053734
2. Mahmood M, Bhutta F, Bhutta MF. Models of service delivery for ear and hearing care in remote or resource-constrained environments. J Laryngol Otol [Internet]. 2019 Jan 1 [cited 2022 Jun 13];133(1):39–48. Available from: https://www.cambridge.org/core/journals/journal-of-laryngology-and-otology/article/models-of-service-delivery-for-ear-and-hearing-care-in-remote-or-resourceconstrained-environments/A79DDE056CDDEA54B09DCB561AE9BBC9
3. Botasso M, Sanches SGG, Bento RF, Samelli AG. Teleaudiometry as a screening method in school children. Clinics. 2015;70(4):283–8.
4. Monica SD, Ramkumar V, Krumm M, Raman N, Nagarajan R, Venkatesh L. School entry level tele-hearing screening in a town in South India – Lessons learnt. Int J Pediatr Otorhinolaryngol [Internet]. 2017;92:130–5. Available from: http://dx.doi.org/10.1016/j.ijporl.2016.11.021
5. Biagio L, Swanepoel DW, Adeyemo A, Hall JW, Vinck B. Asynchronous video-otoscopy with a telehealth facilitator. Telemedicine and e-Health. 2013;19(4):252–8.
6. Swanepoel DW, Koekemoer D, Clark J. Intercontinental hearing assessment - a study in tele-audiology. J Telemed Telecare. 2010;16(5):248–52.
7. Visagie A, Swanepoel DW, Eikelboom RH. Accuracy of remote hearing assessment in a rural community. Telemedicine and e-Health. 2015;21(11):930–7.
8. Cullington H, Kitterick P, Debold L, Weal M, Clarke N, Newberry E, et al. Personalised long-term follow-up of cochlear implant patients using remote care, compared with those on the standard care pathway: Study protocol for a feasibility randomised controlled trial. BMJ Open. 2016;6(5):1–9.
9. Beukes EW, Vlaescu G, Manchaiah V, Baguley DM, Allen PM, Kaldo V, et al. Development and technical functionality of an Internet-based intervention for tinnitus in the UK. Internet Interv [Internet]. 2016;6(August):6–15. Available from: http://dx.doi.org/10.1016/j.invent.2016.08.002
10. Krumm M. Audiology telemedicine. J Telemed Telecare. 2007;13(5):224–9.
11. Landolfi E, Continisio GI, Vecchio V Del, Serra N, Burattini E, Conson M, et al. NeonaTal Assisted TelerehAbilitation (T.A.T.A. Web App) for Hearing-Impaired Children: A Family-Centered Care Model for Early Intervention in Congenital Hearing Loss. Audiol Res. 2022 Apr 1;12(2):182–90.
12. Alenezi EMA, Veselinović T, Tao KFM, Altamimi AAH, Tran TT, Herbert H, et al. Ear Portal: An urban-based ear, nose, and throat, and audiology referral telehealth portal to improve access to specialist ear-health services for children. J Telemed Telecare. 2023;
13. Jacobs PG, Saunders GH. New opportunities and challenges for teleaudiology within department of veterans affairs. J Rehabil Res Dev. 2014;51(5):vii–xii.
14. Tao KFM, Brennan-Jones CG, Capobianco-Fava DM, Jayakody DMP, Friedland PL, Swanepoel DW, et al. Teleaudiology services for rehabilitation with hearing aids in adults: A systematic review. Journal of Speech, Language, and Hearing Research. 2018;61(7):1831–49.
15. Swanepoel DW, Clark JL, Koekemoer D, Hall JW, Krumm M, Ferrari D V., et al. Telehealth in audiology: The need and potential to reach underserved communities. Int J Audiol. 2010;49(3):195–202.
16. Coco L, Davidson A, Marrone N. The role of patient-site facilitators in teleaudiology: A scoping review. Am J Audiol. 2020;29(3 Special Issue):661–75.
17. Muñoz K, Nagaraj NK, Nichols N, Muñoz K, Nagaraj NK, Applied NN. Applied tele-audiology research in clinical practice during the past decade : a scoping review. Int J Audiol [Internet]. 2020;0(0):1–9. Available from: https://doi.org/10.1080/14992027.2020.1817994

For peer review only - http://bmjopen.bmj.com/site/about/guidelines.xhtml

18. Angral S, Varshney S, Aanand P, Raj R. Tele Otology in India: Last 10 Years—A Scopic Review. Indian Journal of Otolaryngology and Head and Neck Surgery [Internet]. 2021; Available from: https://doi.org/10.1007/s12070-021-02546-4
19. Glasgow RE, Harden SM, Gaglio B, Rabin B, Smith ML, Porter GC, et al. RE-AIM planning and evaluation framework: Adapting to new science and practice with a 20-year review. Vol. 7, Frontiers in Public Health. Frontiers Media S.A.; 2019.
20. Chambers DA, Glasgow RE, Stange KC. The dynamic sustainability framework: Addressing the paradox of sustainment amid ongoing change. Implementation Science. 2013 Oct 2;8(1).
21. Glasgow RE, Vinson C, Chambers D, Khoury MJ, Kaplan RM, Hunter C. National Institutes of Health Approaches to Dissemination and Implementation Science: Current and Future Directions. Am J Public Health. 2012;102(7):1274–81.
22. Proctor E, Silmere H, Raghavan R, Hovmand P, Aarons G, Bunger A, et al. Outcomes for implementation research: Conceptual distinctions, measurement challenges, and research agenda. Administration and Policy in Mental Health and Mental Health Services Research. 2011 Mar;38(2):65–76.
23. Damschroder LJ, Aron DC, Keith RE, Kirsh SR, Alexander JA, Lowery JC. Fostering implementation of health services research findings into practice: A consolidated framework for advancing implementation science. Implementation Science. 2009;4(1):1–15.
24. Moullin JC, Dickson KS, Stadnick NA, Albers B, Nilsen P, Broder-Fingert S, et al. Ten recommendations for using implementation frameworks in research and practice. Implement Sci Commun. 2020 Dec;1(1).
25. Campbell J, Theodoros D, Hartley N, Russell T, Gillespie N. Implementation factors are neglected in research investigating telehealth delivery of allied health services to rural children: A scoping review. J Telemed Telecare. 2019;
26. Tricco AC, Lillie E, Zarin W, O'Brien KK, Colquhoun H, Levac D, et al. PRISMA extension for scoping reviews (PRISMA-ScR): Checklist and explanation. Ann Intern Med. 2018;169(7):467–73.
27. Ouzzani M, Hammady H, Fedorowicz Z, Elmagarmid A. Rayyan---a web and mobile app for systematic reviews. Syst Rev. 2016;5(1):210.
28. Pinnock H, Barwick M, Carpenter CR, Eldridge S, Grandes G, Griffiths CJ, et al. Standards for Reporting Implementation Studies (StaRI) Statement. BMJ (Online). 2017;356(March):1–9.
29. Tong A, Sainsbury P, Craig J. Consolidated criteria for reporting qualitative research (COREQ): A 32-item checklist for interviews and focus groups. International Journal for Quality in Health Care. 2007;19(6):349–57.
30. Clarke V, Braun V. Teaching thematic analysis: Overcoming challenges and developing strategies for effective learning. The SAGE Encyclopedia of Qualitative Research Methods. 2012;
31. Braun V, Clarke V. Using thematic analysis in psychology. Qual Res Psychol. 2006;3(2):77–101.
32. Saunders B, Sim J, Kingstone T, Baker S, Waterfield J, Bartlam B, et al. Saturation in qualitative research: exploring its conceptualization and operationalization. Qual Quant. 2018;52(4):1893–907.
33. Swain J. A Hybrid Approach to Thematic Analysis in Qualitative Research: Using a Practical Example. A Hybrid Approach to Thematic Analysis in Qualitative Research: Using a Practical Example. 2018;(January 2018).
34. Emmett SD, Robler SK, Wang NY, Labrique A, Gallo JJ, Hofstetter P. Hearing Norton Sound: A community randomised trial protocol to address childhood hearing loss in rural Alaska. BMJ Open. 2019;9(1).
35. Emmett SD, Robler SK, Gallo JJ, Wang NY, Labrique A, Hofstetter P. Hearing Norton Sound: Mixed methods protocol of a community randomised trial to address childhood hearing loss in rural Alaska. BMJ Open. 2019;9(1):1–10.
36. Robler SK, Inglis SM, Gallo JJ, Parnell HE, Ivanoff P, Ryan S, et al. Hearing Norton Sound: community involvement in the design of a mixed methods community randomized trial in 15 Alaska Native communities. Res Involv Engagem. 2020;6(1):1–11.

For peer review only

37. Dharmar M, Simon A, Sadorra C, Friedland G, Sherwood J, Morrow H, et al. Reducing loss to follow-up with Tele-audiology diagnostic evaluations. Telemedicine Journal and e-Health. 2016;22(2):1–6.
38. Stuart A. Infant Diagnostic Evaluation Via Teleaudiology Following Newborn Screening In Eastern North Carolina. J Early Hear Detect Interv. 2016;1(2):63–71.
39. Campbell W, Hyde M. eEHDI : Functions and Challenges. A Sound Foundation Through Early Amplification: Proceedings of the Fourth International Conference. 2010;115–30, 273p.
40. Hatton JL, Rowlandson J, Beers A, Small S. Telehealth-enabled auditory brainstem response testing for infants living in rural communities: the British Columbia Early Hearing Program experience. Int J Audiol [Internet]. 2019;58(7):381–92. Available from: https://doi.org/10.1080/14992027.2019.1584681
41. Ramkumar V, Nagarajan R, Shankarnarayan VC, Kumaravelu S, Hall JW. Implementation and evaluation of a rural community-based pediatric hearing screening program integrating in-person and tele-diagnostic auditory brainstem response (ABR). BMC Health Serv Res. 2019;19(1):1–13.
42. Ramkumar V, John KR, Selvakumar K, Vanaja CS, Nagarajan R, Hall JW. Cost and outcome of a community-based paediatric hearing screening programme in rural India with application of tele-audiology for follow-up diagnostic hearing assessment. Int J Audiol [Internet]. 2018;57(6):407–14. Available from: https://doi.org/10.1080/14992027.2018.1442592
43. Hofstetter PJ, Kokesh J, Ferguson AS, Hood LJ. The impact of telehealth on wait time for ENT specialty care. Telemed J E Health. 2010;16(5):551–6.
44. Kokesh J, Ferguson AS, Patricoski C. The alaska experience using store-and-forward telemedicine for ENT care in alaska. Otolaryngol Clin North Am [Internet]. 2011;44(6):1359–74. Available from: http://dx.doi.org/10.1016/j.otc.2011.08.010
45. Smith AC, Armfield NR, Wu WI, Brown CA, Perry C. A mobile telemedicine-enabled ear screening service for Indigenous children in Queensland: Activity and outcomes in the first three years. J Telemed Telecare. 2012;18(8):485–9.
46. Smith AC, Armfield NR, Wu WI, Brown CA, Mickan B, Perry C. Changes in paediatric hospital ENT service utilisation following the implementation of a mobile, indigenous health screening service. J Telemed Telecare. 2013;19(7):397–400.
47. Smith AC, Brown C, Bradford N, Caffery LJ, Perry C, Armfield NR. Monitoring ear health through a telemedicine-supported health screening service in Queensland. J Telemed Telecare. 2015;21(8):427–30.
48. Gupta N, Baghotia KS, Rabha M, Sachdeva S, Sahai G, Bhatnagar K, et al. Comprehensive Community Screening of Otological Patients by Trained Technicians Using a Telemedicine Device: An Efficient and Cost-Effective Way to Triage Patients With Ear Diseases. Ear Nose Throat J. 2020;0–5.
49. Ravi P, Ramkumar V, Rajendran A, Saravanam PK, Balasubramaniyan S, Nagarajan R. Tele-audiological surveillance of middle ear status among individuals with cleft lip and/or palate in rural South India. J Am Acad Audiol. 2020;31(3):185–94.
50. Dennis KC, Gladden CF, Noe CM. Telepractice in the Department of Veterans Affairs. Hearing Review [Internet]. 2012;19(11):44–50. Available from: http://search.ebscohost.com/login.aspx?direct=true&db=ccm&AN=2011707530&site=ehost-live&scope=cite
51. Novak R, Cantu AG, Zappler A, Coco L, Champlin C. The Future of Healthcare Delivery : IPE / IPP Audiology and Nursing Student / Faculty Collaboration to Deliver Hearing Aids to Vulnerable Adults via Telehealth. 2016;1(1).
52. Luryi AL, Tower JI, Preston J, Burkland A, Trueheart CE, Hildrew DM. Cochlear Implant Mapping Through Telemedicine - A Feasibility Study. Otology and Neurotology. 2020;41(3):e330–3.
53. Skarżyński PH, Świerniak W, Bruski Ł, Ludwikowski M, Skarżyński H. Comprehensive approach to the National Network of Teleaudiology in World Hearing Center in Kajetany, Poland. Finnish Journal of eHealth and eWelfare. 2018;10(4):354–63.

For peer review only - http://bmjopen.bmj.com/site/about/guidelines.xhtml

54. Skarżyński PH, Swierniak W, Ludwikowski M, Bruski Ł. Telefitting Betwen Kajetany , Poland and Odessa , Ukraine for Cochlear Implants. 2019;1–6.
55. Houston KT. TeleIntervention: Improving Service Delivery to Young Children With Hearing Loss and Their Families Through Telepractice. Perspect Hear Hear Disord Child. 2011;21(2):66–72.
56. Houston KT, Stredler-Brown A. A model of early intervention. 2012. p. 283–96.
57. Blaiser KM, Edwards M, Behl D, Munoz KF. Telepractice Services at Sound Beginnings at Utah State University [Internet]. Vol. 112, The Volta Review. 2012. p. 365–72. Available from: https://login.iris.etsu.edu:3443/login?url=http://search.proquest.com/docview/1430172127?accountid=10771
58. Broekelmann C. ihear® Internet Therapy Program - A Program by St. Joseph Institute for the Deaf. 2012.
59. Lalios AP. ConnectHear TeleIntervention Program. 2012. p. 357–64.
60. Galvan C, Case E, Todd Houston K. Listening and Learning: Using Telepractice to Serve Children and Adults with Hearing Loss. Perspectives on Telepractice. 2014;4(1):11–22.
61. McCarthy M. RIDBC Teleschool: A Hub of Expertise. 2012.
62. McCarthy M. Using technology to support children with sensory disability in remote areas: The RIDBC teleschool model. Telecommunications Journal of Australia. 2011;61(2):1–7.
63. McCarthy M, Muñoz K, White KR. Teleintervention for infants and young children who are deaf or hard-of-hearing. Pediatrics. 2010;126(SUPPL. 1).
64. Gladden C, Beck L, Chandler D. Tele-audiology: Expanding access to hearing care and enhancing patient connectivity. J Am Acad Audiol. 2015;26(9):792–9.
65. Ratanjee-Vanmali H, Swanepoel DW, Laplante-Lévesque A. Patient uptake, experience, and satisfaction using web-based and face-to-face hearing health services: Process evaluation study. J Med Internet Res. 2020;22(3).
66. Ramkumar V, Vanaja CS, Hall JW, Selvakumar K, Nagarajan R. Validation of DPOAE screening conducted by village health workers in a rural community with real-time click evoked tele-auditory brainstem response. Int J Audiol [Internet]. 2018;57(5):370–5. Available from: https://doi.org/10.1080/14992027.2018.1425001
67. Tao KFM, Brennan-Jones CG, Capobianco-Fava DM, Jayakody DMP, Friedland PL, Swanepoel DW, et al. Teleaudiology services for rehabilitation with hearing aids in adults: A systematic review. Journal of Speech, Language, and Hearing Research. 2018;61(7):1831–49.
68. Krumm M. A review of contemporary tele-audiology literature. J Hear Sci. 2016;6(3):9–21.
69. Banbury A, Nancarrow S, Dart J, Gray L, Parkinson L. Telehealth interventions delivering home-based support group videoconferencing: Systematic review. J Med Internet Res. 2018;20(2).
70. Coco L, Carvajal S, Navarro C, Piper R, Marrone N. Community Health Workers as Patient-Site Facilitators in Adult Hearing Aid Services via Synchronous Teleaudiology: Feasibility Results from the Conexiones Randomized Controlled Trial. Ear Hear. 2023 Jan 1;44(1):28–42.
71. Behl DD, Kahn G. Provider perspectives on telepractice for serving families of children who are deaf or hard of hearing. Int J Telerehabil. 2015;7(1):1–11.
72. D'Onofrio KL, Zeng FG. Tele-Audiology: Current State and Future Directions. Front Digit Health. 2022;3(January):1–11.
73. Hao Y, Zhang S, Conner A, Lee NY. The evolution of telepractice use during the covid-19 pandemic: Perspectives of pediatric speech-language pathologists. Int J Environ Res Public Health. 2021;18(22).
74. Kumar M, Mostafa J. Research evidence on strategies enabling integration of electronic health records in the health care systems of low- and middle-income countries: A literature review. International Journal of Health Planning and Management. 2019;34(2):e1016–25.
75. Singh G, Pichora-Fuller MK, Malkowski M, Boretzki M, Launer S. A survey of the attitudes of practitioners toward teleaudiology. Int J Audiol. 2014;53(12):850–60.

For peer review only

76. Cason J, Cohn ER. Telepractice: An Overview and Best Practices. Perspect Augment altern commun. 2014;23(1):4–17.
77. Thomas EE, Taylor ML, Ward EC, Hwang R, Cook R, Ross JA, et al. Beyond forced telehealth adoption: A framework to sustain telehealth among allied health services. J Telemed Telecare. 2022;
78. Turner HC, Archer RA, Downey LE, Isaranuwatchai W, Chalkidou K, Jit M, et al. An Introduction to the Main Types of Economic Evaluations Used for Informing Priority Setting and Resource Allocation in Healthcare : Key Features , Uses , and Limitations. 2021;9(August):1–17.

Figure Legend:
Figure 1 - Flow chart

For peer review only

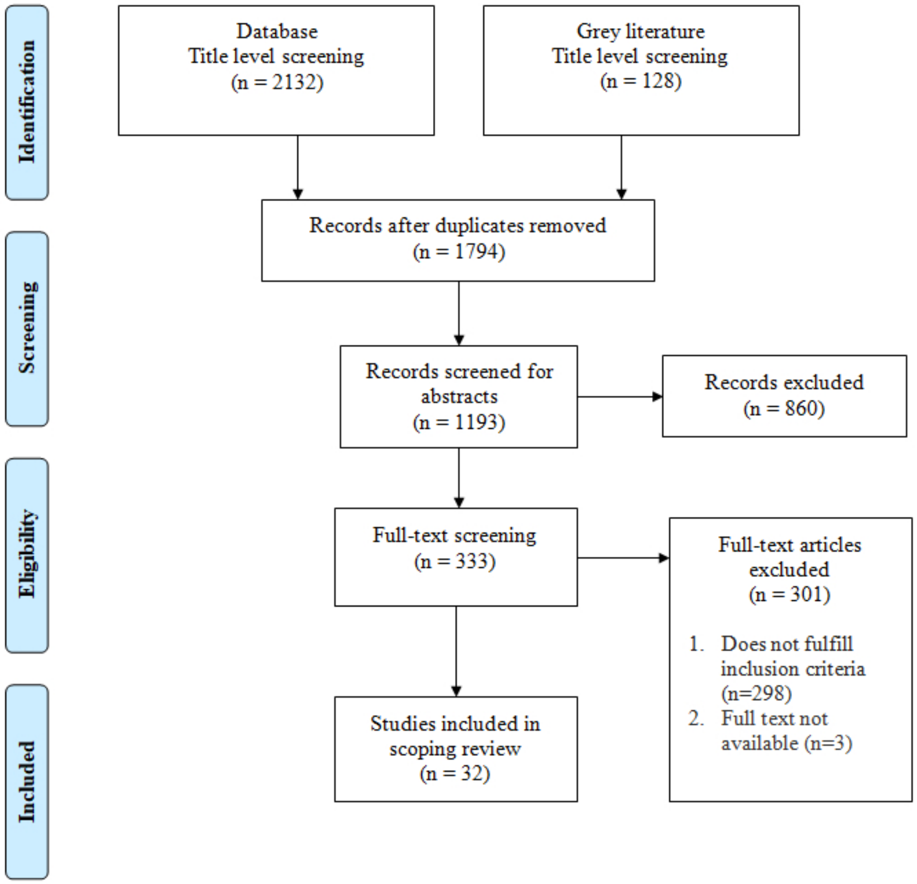

Figure 1: PRISMA flow diagram representing the study's search process

256x236mm (72 x 72 DPI)

Preferred Reporting Items for Systematic reviews and Meta-Analyses extension for Scoping Reviews (PRISMA-ScR) Checklist

SECTION	ITEM	PRISMA-ScR CHECKLIST ITEM	REPORTED ON PAGE #
**TITLE**			
Title	1	Identify the report as a scoping review.	
**ABSTRACT**			
Structured summary	2	Provide a structured summary that includes (as applicable): background, objectives, eligibility criteria, sources of evidence, charting methods, results, and conclusions that relate to the review questions and objectives.	
**INTRODUCTION**			
Rationale	3	Describe the rationale for the review in the context of what is already known. Explain why the review questions/objectives lend themselves to a scoping review approach.	
Objectives	4	Provide an explicit statement of the questions and objectives being addressed with reference to their key elements (e.g., population or participants, concepts, and context) or other relevant key elements used to conceptualize the review questions and/or objectives.	
**METHODS**			
Protocol and registration	5	Indicate whether a review protocol exists; state if and where it can be accessed (e.g., a Web address); and if available, provide registration information, including the registration number.	
Eligibility criteria	6	Specify characteristics of the sources of evidence used as eligibility criteria (e.g., years considered, language, and publication status), and provide a rationale.	
Information sources*	7	Describe all information sources in the search (e.g., databases with dates of coverage and contact with authors to identify additional sources), as well as the date the most recent search was executed.	
Search	8	Present the full electronic search strategy for at least 1 database, including any limits used, such that it could be repeated.	
Selection of sources of evidence†	9	State the process for selecting sources of evidence (i.e., screening and eligibility) included in the scoping review.	
Data charting process‡	10	Describe the methods of charting data from the included sources of evidence (e.g., calibrated forms or forms that have been tested by the team before their use, and whether data charting was done independently or in duplicate) and any processes for obtaining and confirming data from investigators.	
Data items	11	List and define all variables for which data were sought and any assumptions and simplifications made.	
Critical appraisal of individual sources of evidence§	12	If done, provide a rationale for conducting a critical appraisal of included sources of evidence; describe the methods used and how this information was used in any data synthesis (if appropriate).	
Synthesis of results	13	Describe the methods of handling and summarizing the data that were charted.	

SECTION	ITEM	PRISMA-ScR CHECKLIST ITEM	REPORTED ON PAGE #
**RESULTS**			
Selection of sources of evidence	14	Give numbers of sources of evidence screened, assessed for eligibility, and included in the review, with reasons for exclusions at each stage, ideally using a flow diagram.	
Characteristics of sources of evidence	15	For each source of evidence, present characteristics for which data were charted and provide the citations.	
Critical appraisal within sources of evidence	16	If done, present data on critical appraisal of included sources of evidence (see item 12).	
Results of individual sources of evidence	17	For each included source of evidence, present the relevant data that were charted that relate to the review questions and objectives.	
Synthesis of results	18	Summarize and/or present the charting results as they relate to the review questions and objectives.	
**DISCUSSION**			
Summary of evidence	19	Summarize the main results (including an overview of concepts, themes, and types of evidence available), link to the review questions and objectives, and consider the relevance to key groups.	
Limitations	20	Discuss the limitations of the scoping review process.	
Conclusions	21	Provide a general interpretation of the results with respect to the review questions and objectives, as well as potential implications and/or next steps.	
**FUNDING**			
Funding	22	Describe sources of funding for the included sources of evidence, as well as sources of funding for the scoping review. Describe the role of the funders of the scoping review.	

JBI = Joanna Briggs Institute; PRISMA-ScR = Preferred Reporting Items for Systematic reviews and Meta-Analyses extension for Scoping Reviews.

* Where *sources of evidence* (see second footnote) are compiled from, such as bibliographic databases, social media platforms, and Web sites.

† A more inclusive/heterogeneous term used to account for the different types of evidence or data sources (e.g., quantitative and/or qualitative research, expert opinion, and policy documents) that may be eligible in a scoping review as opposed to only studies. This is not to be confused with *information sources* (see first footnote).

‡ The frameworks by Arksey and O'Malley (6) and Levac and colleagues (7) and the JBI guidance (4, 5) refer to the process of data extraction in a scoping review as data charting.

§ The process of systematically examining research evidence to assess its validity, results, and relevance before using it to inform a decision. This term is used for items 12 and 19 instead of "risk of bias" (which is more applicable to systematic reviews of interventions) to include and acknowledge the various sources of evidence that may be used in a scoping review (e.g., quantitative and/or qualitative research, expert opinion, and policy document).

From: Tricco AC, Lillie E, Zarin W, O'Brien KK, Colquhoun H, Levac D, et al. PRISMA Extension for Scoping Reviews (PRISMAScR): Checklist and Explanation. Ann Intern Med. 2018;169:467–473. doi: 10.7326/M18-0850.

For peer review only

Supplemental file 2
Search strategies used for the article selection

- ((((((((((((((((telehealth[Title]) OR (telepractice[Title])) OR (teleaudiology[Title])) OR (telemedicine[Title])) OR (telerehabilitation[Title])) OR (teletherapy[Title])) OR (mhealth[Title])) OR (ehealth[Title])) OR (internet-based[Title])) OR (computer-based[Title])) OR (web-based[Title])) OR (tablet-based[Title])) OR (mobile-based[Title])) OR (application[Title])) AND (Audiology[Title])) OR (Aural rehabilitation[Title])) OR (teleaudiology[Title]) Filters: English, from 2010 - 2023
- ((((((((((((((((((((((((telehealth[Title]) OR (telepractice[Title])) OR (teleaudiology[Title])) OR (telemedicine[Title])) OR (telerehabilitation[Title])) OR (teletherapy[Title])) OR (mhealth[Title])) OR (ehealth[Title])) OR (internet-based[Title])) OR (computer-based[Title])) OR (web-based[Title])) OR (tablet-based[Title])) OR (mobile-based[Title])) OR (application[Title])) AND (Audiology[Title])) OR (Aural rehabilitation[Title])) OR (teleaudiology[Title])) OR (Auditory verbal therapy)) OR (hearing loss)) OR (hearing screening)) OR (ear care)) AND (synchronous)) OR (asynchronous)) AND (Adoption)) OR (implementation) Filters: English, from 2010 - 2024
- (((((((((((((((((((((Teleaudiology[Title]) OR (Telepractice[Title])) OR (Telehealth[Title])) OR (Telemedicine[Title])) OR (Telerehabilitation[Title])) OR (Teletherapy[Title])) OR (e-health[Title])) OR (m-health[Title])) OR (Internet-based[Title])) OR (Computer-based[Title])) OR (Web-based[Title])) OR (Tablet-based[Title])) OR (Mobile-based[Title])) AND (tele-audiology[MeSH Terms])) AND (Implementation[Title/Abstract])) OR (Adoption[Title/Abstract])) OR (sustainability[Title/Abstract])) OR (acceptance[Title/Abstract])) OR (perspectives[Title/Abstract])) OR (perceptions[Title/Abstract])) OR (readiness[Title/Abstract])) AND (teleaudiology[Title]) Filters: English
- (((((((Synchronous[Title]) OR (Asynchronous[Title])) OR (Hybrid[Title])) OR ("Store and forward"[Title])) OR (Videoconference[Title])) OR (Videoconferencing[Title])) OR (Real-time[Title])) AND (Teleaudiology[Title]) Filters: English
- (((((((((((((((((Telepractice[Title]) OR (Teleaudiology[Title])) OR (telehealth[Title])) OR (telemedicine[Title])) OR (teletherapy[Title])) OR (telerehabilitation[Title])) OR (e-health[Title])) OR (m-health[Title])) OR (Internet-based[Title])) OR (Computer-based[Title])) OR (Web-based[Title])) OR (Tablet-based[Title])) OR (Mobile-based[Title])) AND ("Auditory Brainstem Response"[Title])) OR (Audiometry[Title])) OR (Tympanometry[Title])) OR ("Auditory steady state response"[Title])) OR (Videotoscopy[Title]) Filters: English, from 2010 - 2023
- (((((((((((((((((((((Telepractice[Title]) OR (Teleaudiology[Title])) OR (telehealth[Title])) OR (telemedicine[Title])) OR (teletherapy[Title])) OR (telerehabilitation[Title])) OR (e-health[Title])) OR (m-health[Title])) OR (Internet-based[Title])) OR (Computer-based[Title])) OR (Web-based[Title])) OR (Tablet-based[Title])) OR (Mobile-based[Title])) AND (Cochlear Implant[Title])) OR (Hearing aids[Title])) OR (hearing aid trial[Title])) OR (hearing aid programming[Title])) OR (hearing aid trial[Title])) OR (Tinnitus[Title])) OR (Vestibular[Title])) OR (Middle ear diseases[Title])) OR (Cochlear implant mapping[Title]) Filters: English, from 2010 - 2023
- (((((((((((((((((((Teleaudiology[Title/Abstract]) OR (Telehealth[Title/Abstract])) OR (Telepractice[Title/Abstract])) OR (Telemedicine[Title/Abstract])) OR (Telerehabilitation[Title/Abstract])) OR (Teletherapy[Title/Abstract])) OR (e-health[Title/Abstract])) OR (m-health[Title/Abstract])) OR (Internet-based[Title/Abstract])) OR (Computer-based[Title/Abstract])) OR (Web-based[Title/Abstract])) OR (Tablet-based[Title/Abstract])) OR (Mobile-

based[Title/Abstract])) AND (Audiology[Title/Abstract])) OR (Aural rehabilitation[Title])) OR (Auditory verbal therapy[Title])) OR (Ear care[Title])) OR (Hearing care[Title])) OR (Hearing health[Title])) OR (Hearing loss[Title]) Filters: English, from 2010 - 2023

- (((((((((((((((((((Teleaudiology[Title]) OR (Telepractice[Title])) OR (Telehealth[Title])) OR (Telemedicine[Title])) OR (Telerehabilitation[Title])) OR (Teletherapy[Title])) OR (e-health[Title])) OR (m-health[Title])) OR (Internet-based[Title])) OR (Computer-based[Title])) OR (Web-based[Title])) OR (Tablet-based[Title])) OR (Mobile-based[Title])) AND (Evaluation[Title])) OR (Diagnostics[Title])) OR (Assessment[Title])) OR (Treatment[Title])) OR (Management[Title])) OR (Rehabilitation[Title])) OR (Therapy[Title]) Filters: English, from 2008 - 2024
- ((((((((((((((((((((((((((("Teleaudiology"[Title]) OR (Telepractice[Title])) OR (Telehealth[Title])) OR (Telemedicine[Title])) OR (Telerehabilitation[Title])) OR (Teletherapy[Title])) OR (e-health[Title])) OR (m-health[Title])) OR (Internet-based[Title])) OR (Computer-based[Title])) OR (Web-based[Title])) OR (Tablet-based[Title])) OR (Mobile-based[Title]))) AND (Synchronous[Title])) OR (Asynchronous[Title])) OR (Hybrid[Title])) OR ("Store and forward"[Title])) OR (Videoconference[Title])) OR (Videoconferencing[Title])) OR ("Real time"[Title]) Filters: English, from 2010 - 2024
- ((((((("Teleaudiology"[Title]) AND (Implementation[Title])) OR (Adoption[Title])) OR (Sustainability[Title])) OR (Acceptance[Title])) OR (Readiness[Title])) OR (Perspectives[Title])) OR (Perceptions[Title]) Filters: English, from 2010 – 2024
- ((Audiology) AND (implementation)) AND (teleaudiology)
- telehealth OR tele audiology OR telepractice AND audiology OR "aural rehabilitation" Filters: from 2010 - 2023
- telehealth OR (teleaudiology) OR telepractice AND audiology OR "aural rehabilitation" AND (implementation OR adoption) Filters: English, from 2010 - 2023
- telehealth OR teleaudiology OR telepractice OR Telemedicine AND audiology OR "aural rehabilitation" AND implementation OR adoption Filters: English, from 2008 – 2024
- (TITLE-ABS-KEY (teleaudiology) AND TITLE-ABS-KEY (implementation) OR TITLE-ABS-KEY (adoption))
- (TITLE-ABS-KEY (teleaudiology) OR TITLE-ABS-KEY (telepractice) OR TITLE-ABS-KEY (telehealth) AND TITLE-ABS-KEY (evaluation) AND TITLE-ABS-KEY (aural AND rehabilitation) AND TITLE-ABS-KEY (implementation) OR TITLE-ABS-KEY (adoption)) AND PUBYEAR > 2010 AND PUBYEAR < 2024
- (TITLE-ABS-KEY (teleaudiology) OR TITLE-ABS-KEY (telepractice) OR TITLE-ABS-KEY (telehealth) AND TITLE-ABS-KEY (aural AND rehabilitation) AND TITLE-ABS-KEY (implementation)) AND PUBYEAR > 2013 AND PUBYEAR < 2024
- (TITLE-ABS-KEY (teleaudiology) AND TITLE-ABS-KEY (implementation) OR ALL (sustainability) AND ALL (synchronous) OR ALL (asynchronous)) AND PUBYEAR > 2009
- (TITLE-ABS-KEY (teleaudiology) AND TITLE-ABS-KEY (implementation) OR ALL (acceptance) AND ALL (evaluation)) AND PUBYEAR > 2009
- (TITLE-ABS-KEY (teleaudiology) AND TITLE-ABS-KEY (implementation) OR ALL (readiness) AND ALL (evaluation) AND ALL (audiology)) AND PUBYEAR > 2009

For peer review only

- (TITLE-ABS-KEY (teleaudiology) AND TITLE-ABS-KEY (implementation) OR ALL (perspectives) OR ALL (perceptions)) AND PUBYEAR > 2010 AND PUBYEAR < 2024
- (TITLE-ABS-KEY (teleaudiology) AND ALL (audiology) AND ALL (pediatric) OR ALL (children)) AND PUBYEAR > 2009 AND PUBYEAR < 2024
- (TITLE-ABS-KEY (telemedicine) OR TITLE-ABS-KEY (telehealth) OR ALL (telepractice) AND ALL (audiology) OR ALL (aural)) AND PUBYEAR > 2009
- telepractice AND audiology AND evaluation OR newborn
- telepractice AND audiology AND evaluation OR newborn
- (TITLE-ABS-KEY (telerehabilitation) AND TITLE-ABS-KEY (audiology) OR TITLE-ABS-KEY (aural AND rehabilitation) OR TITLE-ABS-KEY (auditory AND verbal) AND TITLE-ABS-KEY (pediatric) OR TITLE-ABS-KEY (paediatric)) AND PUBYEAR > 2009
- (TITLE-ABS-KEY (telerehabilitation) OR TITLE-ABS-KEY (teletherapy) AND TITLE-ABS-KEY (audiology) AND TITLE-ABS-KEY (pediatric) OR TITLE-ABS-KEY (paediatric) OR TITLE-ABS-KEY (child) OR TITLE-ABS-KEY (children)) AND PUBYEAR > 2009
- (TITLE-ABS-KEY (telerehabilitation) OR TITLE-ABS-KEY (teletherapy) OR TITLE-ABS-KEY (mhealth) OR TITLE-ABS-KEY (ehealth) AND TITLE-ABS-KEY (teenage) OR TITLE-ABS-KEY (adolescent) AND TITLE-ABS-KEY (hearing AND loss) OR TITLE-ABS-KEY (hearing AND care) OR TITLE-ABS-KEY (hearing AND health)) AND PUBYEAR > 2009
- (TITLE-ABS-KEY (internet) OR TITLE-ABS-KEY (computer) OR TITLE-ABS-KEY (web) OR TITLE-ABS-KEY (tablet AND based) OR TITLE-ABS-KEY (mobile AND based) AND TITLE-ABS-KEY (adults) OR TITLE-ABS-KEY (geriatric) OR TITLE-ABS-KEY (old AND age) AND TITLE-ABS-KEY (ear AND care)) AND PUBYEAR > 2009
- (TITLE-ABS-KEY (teleaudiology) AND TITLE-ABS-KEY (internet AND based) OR TITLE-ABS-KEY (computer AND based) OR TITLE-ABS-KEY (web AND based) OR TITLE-ABS-KEY (tablet AND based) OR TITLE-ABS-KEY (mobile AND based)) AND PUBYEAR > 2009
- (TITLE-ABS-KEY (teleaudiology) AND TITLE-ABS-KEY (internet AND based) OR TITLE-ABS-KEY (computer AND based) OR TITLE-ABS-KEY (web AND based) OR TITLE-ABS-KEY (tablet AND based) OR TITLE-ABS-KEY (mobile AND based) AND TITLE-ABS-KEY (diagnostics) OR TITLE-ABS-KEY (assessments)) AND PUBYEAR > 2009
- (TITLE-ABS-KEY (teleaudiology) AND TITLE-ABS-KEY (internet AND based) OR TITLE-ABS-KEY (computer AND based) OR TITLE-ABS-KEY (web AND based) OR TITLE-ABS-KEY (tablet AND based) OR TITLE-ABS-KEY (mobile AND based) AND TITLE-ABS-KEY (treatment) OR TITLE-ABS-KEY (management) OR TITLE-ABS-KEY (rehabilitation) OR TITLE-ABS-KEY (therapy)) AND PUBYEAR > 2009
- (TITLE-ABS-KEY (teleaudiology) AND TITLE-ABS-KEY (hybrid) OR TITLE-ABS-KEY (store) OR TITLE-ABS-KEY (forward) OR TITLE-ABS-KEY (videoconference) OR TITLE-ABS-KEY (videoconferencing) OR TITLE-ABS-KEY (real AND time)) AND PUBYEAR > 2009
- (TITLE-ABS-KEY (teleaudiology) AND TITLE-ABS-KEY (auditory AND brainstem AND response) OR TITLE-ABS-KEY (audiometry) OR TITLE-ABS-KEY (tympanometry) OR TITLE-ABS-KEY (auditory AND steady AND state AND response)) AND PUBYEAR > 2009

For peer review only

- (TITLE-ABS-KEY (teleaudiology) AND TITLE-ABS-KEY (video-otoscopy) OR TITLE-ABS-KEY (cochlear AND implant) OR TITLE-ABS-KEY (hearing AND aids) OR TITLE-ABS-KEY (hearing AND aid AND trial) AND TITLE-ABS-KEY (hearing AND aid AND fitting)) AND PUBYEAR > 2009
- (TITLE-ABS-KEY (teleaudiology) AND TITLE-ABS-KEY (cochlear AND implant AND mapping) OR TITLE-ABS-KEY (tinnitus) OR TITLE-ABS-KEY (vestibular) OR TITLE-ABS-KEY (middle AND ear AND diseases)) AND PUBYEAR > 2009
- (TITLE-ABS-KEY (teleaudiology) OR TITLE-ABS-KEY (telepractice) OR TITLE-ABS-KEY (telehealth) AND TITLE-ABS-KEY (rural) OR TITLE-ABS-KEY (suburban) OR TITLE-ABS-KEY (urban) OR TITLE-ABS-KEY (village) OR TITLE-ABS-KEY (hamlet) OR TITLE-ABS-KEY (country) AND TITLE-ABS-KEY (audiology)) AND PUBYEAR > 2009
- (TITLE-ABS-KEY (teleaudiology) AND TITLE-ABS-KEY (community) OR TITLE-ABS-KEY (school) OR TITLE-ABS-KEY (district) OR TITLE-ABS-KEY (remote) OR TITLE-ABS-KEY (home)) AND PUBYEAR > 2009
- (TITLE-ABS-KEY (teleaudiology) AND TITLE-ABS-KEY (universities) OR TITLE-ABS-KEY (clinic) OR TITLE-ABS-KEY (pre-school) OR TITLE-ABS-KEY (day AND care) OR TITLE-ABS-KEY (primary)) AND PUBYEAR > 2009
- teleaudiology OR telepractice OR telehealth AND audiology AND implementation OR adoption OR sustainability
- teleaudiology AND audiology AND implementation OR adoption OR sustainability
- teleaudiology OR telepractice OR telehealth OR telemedicine AND audiology AND implementation OR adoption OR sustainability
- teleaudiology OR telepractice OR telehealth OR telemedicine OR "m-health" OR "e-health" AND audiology AND implementation OR adoption OR sustainability
- teleaudiology OR telepractice OR telehealth OR telemedicine OR "m-health" OR "e-health" OR "computer-based" OR "Web-based" AND audiology OR aural rehabilitation AND implementation OR adoption OR sustainability
- teleaudiology OR telepractice OR telehealth OR telemedicine OR "mobile-based" OR internet-based OR "tablet-based" OR "m-health" OR "e-health" OR "computer-based" OR "Web-based" AND audiology OR aural rehabilitation AND implementation OR adoption OR sustain
- "teleaudiology" OR telepractice OR telehealth OR telemedicine OR "mobile-based" OR internet-based OR "tablet-based" OR "m-health" OR "e-health" OR "computer-based" OR "Web-based" AND audiology OR aural rehabilitation OR "hearing loss" OR "auditory verbal t
- "teleaudiology" OR telepractice OR telehealth OR telemedicine OR "mobile-based" OR internet-based OR "tablet-based" OR "m-health" OR "e-health" OR "computer-based" OR "Web-based" AND audiology OR aural rehabilitation OR "hearing loss" OR "ear care"
- "teleaudiology" OR telepractice OR telehealth OR telemedicine OR "mobile-based" OR internet-based OR "tablet-based" OR "m-health" OR "e-health" OR "computer-based" OR "Web-based" AND audiology OR aural rehabilitation OR "hearing loss" OR "hearing health"
- "teleaudiology" OR telepractice OR telehealth OR telemedicine OR "mobile-based" OR internet-based OR "tablet-based" OR "m-health" OR "e-health" OR "computer-based" OR "Web-based" AND audiology OR aural rehabilitation OR "hearing loss" OR "hearing care"

For peer review only

- "teleaudiology" OR telepractice OR telehealth OR telemedicine OR "mobile-based" OR internet-based OR "tablet-based" OR "m-health" OR "e-health" OR "computer-based" OR "Web-based" AND audiology OR aural rehabilitation AND "Evaluation" OR "Diagnostics" OR "A
- "teleaudiology" OR telepractice OR telehealth OR telemedicine OR "mobile-based" OR internet-based OR "tablet-based" OR "m-health" OR "e-health" OR "computer-based" OR "Web-based" AND audiology OR aural rehabilitation AND "Treatment" OR "Management"
- "teleaudiology" OR telepractice OR telehealth OR telemedicine OR "mobile-based" OR internet-based OR "tablet-based" OR "m-health" OR "e-health" OR "computer-based" OR "Web-based" AND audiology OR aural rehabilitation AND therapy OR rehabilitation
- "teleaudiology" OR telepractice OR telehealth OR telemedicine OR "mobile-based" OR internet-based OR "tablet-based" OR "m-health" OR "e-health" OR "computer-based" OR "Web-based" AND audiology OR aural rehabilitation AND implementation
- "teleaudiology" OR telepractice OR telehealth OR telemedicine OR "mobile-based" OR internet-based OR "tablet-based" OR "m-health" OR "e-health" OR "computer-based" OR "Web-based" AND audiology OR aural rehabilitation AND implementation OR adoption
- Audiology OR aural rehabilitation AND sustainability OR acceptance AND "teleaudiology" OR telepractice OR telehealth OR telemedicine OR "mobile-based" OR internet-based OR "tablet-based" OR "m-health" OR "e-health" OR "computer-based" OR "Web-based"
- Audiology OR aural rehabilitation AND Readiness OR Perspectives OR Perceptions AND "teleaudiology" OR telepractice OR telehealth OR telemedicine OR "mobile-based" OR internet-based OR "tablet-based" OR "m-health" OR "e-health" OR "computer-based" OR "Web-b
- Pediatric OR Paediatric AND "teleaudiology" OR telepractice OR telehealth OR telemedicine OR "mobile-based" OR internet-based OR "tablet-based" OR "m-health" OR "e-health" OR "computer-based" OR "Web-b
- Infant OR children AND audiology AND "teleaudiology" OR telepractice OR telehealth OR telemedicine OR "mobile-based" OR internet-based OR "tablet-based" OR "m-health" OR "e-health" OR "computer-based" OR "Web-b
- Teenage OR Adolescent AND audiology AND "teleaudiology" OR telepractice OR telehealth OR telemedicine OR "mobile-based" OR internet-based OR "tablet-based" OR "m-health" OR "e-health" OR "computer-based" OR "Web-b
- Adults OR Geriatric OR Old age AND audiology AND "teleaudiology" OR telepractice OR telehealth OR telemedicine OR "mobile-based" OR internet-based OR "tablet-based" OR "m-health" OR "e-health" OR "computer-based" OR "Web-b
- Synchronous OR Asynchronous OR Hybrid OR "Store and forward" OR Videoconference OR Videoconferencing OR "Real-time" AND audiology AND "teleaudiology" OR telepractice OR telehealth OR telemedicine OR "mobile-based" OR internet-based OR "tablet-based" OR "m-

For peer review only

- "Auditory Brainstem Response" OR Audiometry AND audiology AND "teleaudiology" OR telepractice OR telehealth OR telemedicine OR "mobile-based" OR internet-based OR "tablet-based" OR "m-
- "Tympanometry" OR "Auditory steady state response" AND audiology AND "teleaudiology" OR telepractice OR telehealth OR telemedicine OR "mobile-based" OR internet-based OR "tablet-based"
- "Video-otoscopy" OR "Cochlear Implant" AND audiology AND "teleaudiology" OR telepractice OR telehealth OR telemedicine OR "mobile-based" OR internet-based OR "tablet-based"
- "Hearing aids" OR "Hearing aid trial" OR " Hearing aid fitting" AND audiology AND "teleaudiology" OR telepractice OR telehealth OR telemedicine OR "mobile-based" OR internet-based OR "tablet-based"
- "Cochlear implant mapping" OR "Tinnitus" OR "Vestibular" OR "Middle ear diseases" AND audiology AND "teleaudiology" OR telepractice OR telehealth OR telemedicine OR "mobile-based" OR internet-based OR "tablet-based"
- "Rural" OR "Suburban" OR "Urban" OR "Village" AND audiology AND "teleaudiology" OR telepractice OR telehealth OR telemedicine OR "mobile-based" OR internet-based OR "tablet-based"
- "Hamlet" OR "Country" OR "Community-based" AND audiology AND "teleaudiology" OR telepractice OR telehealth OR telemedicine OR "mobile-based" OR internet-based OR "tablet-based"
- "School-based" OR "School" OR "District" OR "Remote care" AND audiology AND "teleaudiology" OR telepractice OR telehealth OR telemedicine OR "mobile-based" OR internet-based OR "tablet-based"
- "home-based" OR "Universities" OR "Clinic" AND audiology AND "teleaudiology" OR telepractice OR telehealth OR telemedicine OR "mobile-based" OR internet-based OR "tablet-based"
- "Pre-school" OR "Day care" OR "Primary schools" AND audiology AND "teleaudiology" OR telepractice OR telehealth OR telemedicine OR "mobile-based" OR internet-based OR "tablet-based"

For peer review only

Supplemental file 3

Initial Decision Profile

Study code:

Full Citation:

1. **Is it an implementation study?**

   ❑ Yes ❑ No ❑ Maybe

2. **Has it been published between January, 2010 - April, 2021?**

   ❑ Yes ❑ No ❑ Maybe

3. **Did the project span across a period of 2 or more years?**

   ❑ Yes ❑ No ❑ Maybe

4. **Is it in the field of Tele-audiology?**

   ❑ Yes ❑ No ❑ Maybe

5. **Does it involve screening, diagnostic and rehabilitative services?**

   ❑ Yes ❑ No ❑ Maybe

Notes:

❑ **ELIGIBLE** ❑ **NOT ELIGIBLE**

For peer review only

Appendix 7 - StaRI Checklist

<table>
<tr><th rowspan="2">Checklist item</th><th rowspan="2">Item number</th><th colspan="2">Emmett et al. (2019)</th><th colspan="2">Emmett et al. (2019)</th><th colspan="2">Robler et al. (2020)</th><th colspan="2">Dharmar et al. (2016)</th></tr>
<tr><th>Implementation strategy</th><th>Intervention</th><th>Implementation strategy</th><th>Intervention</th><th>Implementation strategy</th><th>Intervention</th><th>Implementation strategy</th><th>Intervention</th></tr>
<tr><td>Title</td><td>1</td><td colspan="2">?</td><td colspan="2">?</td><td colspan="2">?</td><td colspan="2">X</td></tr>
<tr><td>Abstract</td><td>2</td><td colspan="2">?</td><td colspan="2">?</td><td colspan="2">?</td><td colspan="2">X</td></tr>
<tr><td>Introduction</td><td>3</td><td colspan="2">✓</td><td colspan="2">✓</td><td colspan="2">✓</td><td colspan="2">✓</td></tr>
<tr><td>Rationale</td><td>4</td><td>X</td><td>✓</td><td>X</td><td>✓</td><td>?</td><td>✓</td><td>X</td><td>✓</td></tr>
<tr><td>Aims and objectives</td><td>5</td><td colspan="2">✓</td><td colspan="2">✓</td><td colspan="2">✓</td><td colspan="2">?</td></tr>
<tr><td>Design</td><td>6</td><td colspan="2">?</td><td colspan="2">?</td><td colspan="2">?</td><td colspan="2">?</td></tr>
<tr><td>Context</td><td>7</td><td colspan="2">✓</td><td colspan="2">✓</td><td colspan="2">✓</td><td colspan="2">?</td></tr>
<tr><td>Targeted ‘sites’</td><td>8</td><td>✓</td><td>✓</td><td>✓</td><td>✓</td><td>✓</td><td>✓</td><td>?</td><td>✓</td></tr>
<tr><td>Description</td><td>9</td><td>✓</td><td>✓</td><td>✓</td><td>✓</td><td>✓</td><td>✓</td><td>?</td><td>✓</td></tr>
<tr><td>Sub-groups</td><td>10</td><td colspan="2">N/A</td><td colspan="2">✓</td><td colspan="2">N/A</td><td colspan="2">N/A</td></tr>
<tr><td>Outcomes</td><td>11</td><td>✓</td><td>✓</td><td>✓</td><td>✓</td><td>✓</td><td>✓</td><td>?</td><td>✓</td></tr>
<tr><td>Process evaluation</td><td>12</td><td colspan="2">✓</td><td colspan="2">✓</td><td colspan="2">✓</td><td colspan="2">✓</td></tr>
<tr><td>Economic evaluation</td><td>13</td><td>X</td><td>X</td><td>X</td><td>X</td><td>X</td><td>X</td><td>X</td><td>X</td></tr>
<tr><td>Sample size</td><td>14</td><td colspan="2">✓</td><td colspan="2">✓</td><td colspan="2">✓</td><td colspan="2">✓</td></tr>
<tr><td>Analysis</td><td>15</td><td colspan="2">✓</td><td colspan="2">✓</td><td colspan="2">✓</td><td colspan="2">✓</td></tr>
<tr><td>Sub-group analyses</td><td>16</td><td colspan="2">N/A</td><td colspan="2">✓</td><td colspan="2">N/A</td><td colspan="2">N/A</td></tr>
<tr><td>Characteristics</td><td>17</td><td colspan="2">N/A</td><td colspan="2">N/A</td><td>✓</td><td>N/A</td><td>?</td><td>✓</td></tr>
<tr><td>Outcomes</td><td>18</td><td colspan="2">N/A</td><td colspan="2">N/A</td><td>✓</td><td>N/A</td><td>?</td><td>✓</td></tr>
<tr><td>Process outcomes</td><td>19</td><td colspan="2">N/A</td><td colspan="2">N/A</td><td colspan="2">X</td><td colspan="2">X</td></tr>
<tr><td>Economic evaluation</td><td>20</td><td colspan="2">N/A</td><td colspan="2">N/A</td><td>X</td><td>X</td><td>X</td><td>X</td></tr>
<tr><td>Sub-group analyses</td><td>21</td><td colspan="2">N/A</td><td colspan="2">N/A</td><td colspan="2">N/A</td><td colspan="2">✓</td></tr>
<tr><td>Fidelity/ adaptation</td><td>22</td><td colspan="2">N/A</td><td colspan="2">N/A</td><td>✓</td><td>N/A</td><td>X</td><td>X</td></tr>
<tr><td>Contextual changes</td><td>23</td><td colspan="2">N/A</td><td colspan="2">N/A</td><td colspan="2">✓</td><td colspan="2">X</td></tr>
<tr><td>Harms</td><td>24</td><td colspan="2">N/A</td><td colspan="2">N/A</td><td colspan="2">N/A</td><td colspan="2">N/A</td></tr>
<tr><td>Structured discussion</td><td>25</td><td colspan="2">N/A</td><td colspan="2">N/A</td><td colspan="2">✓</td><td colspan="2">✓</td></tr>
<tr><td>Implications</td><td>26</td><td colspan="2">N/A</td><td colspan="2">N/A</td><td>✓</td><td>N/A</td><td>X</td><td>X</td></tr>
<tr><td>Statements</td><td>27</td><td colspan="2">✓</td><td colspan="2">✓</td><td colspan="2">✓</td><td colspan="2">✓</td></tr>
</table>

Appendix 7 - StaRI Checklist

Checklist item	Item number	Stuart (2016)		W. Campbell and Hyde (2010)		Hatton et al. (2019a)		Ramkumar et al. (2018a)	
		Implementation strategy	Intervention	Implementation strategy	Intervention	Implementation strategy	Intervention	Implementation strategy	Intervention
Title	**1**	X		X		X		X	
Abstract	**2**	X		X		✓		✓	
Introduction	**3**	✓		✓		✓		✓	
Rationale	**4**	X	✓	X	✓	X	✓	X	✓
Aims and objectives	**5**	✓		?		✓		N/A	
Design	**6**	?		?		?		?	
Context	**7**	✓		✓		✓		X	
Targeted 'sites'	**8**	✓	✓	✓	✓	✓	✓	✓	✓
Description	**9**	✓	✓	✓	✓	✓	✓	✓	✓
Sub-groups	**10**	N/A		N/A		N/A		N/A	
Outcomes	**11**	X	✓	X	X	✓	✓	X	✓
Process evaluation	**12**	X		X		✓		N/A	
Economic evaluation	**13**	X	X	X	X	✓	✓	✓	✓
Sample size	**14**	?		✓		✓		✓	
Analysis	**15**	✓		X		✓		✓	
Sub-group analyses	**16**	N/A		N/A		N/A		N/A	
Characteristics	**17**	X	✓	✓	✓	✓	✓	N/A	✓
Outcomes	**18**	X	✓	X	X	✓	✓	N/A	✓
Process outcomes	**19**	X		X		✓		N/A	
Economic evaluation	**20**	X	X	X	X	✓	✓	✓	✓
Sub-group analyses	**21**	X		N/A		N/A		N/A	
Fidelity/ adaptation	**22**	X	X	X	X	✓	X	X	X
Contextual changes	**23**	✓		✓		✓		✓	
Harms	**24**	N/A		N/A		N/A		N/A	
Structured discussion	**25**	✓		✓		✓		✓	
Implications	**26**	X	X	✓	✓	✓	✓	✓	✓
Statements	**27**	✓		?		✓		✓	

Appendix 7 - StaRI Checklist

Checklist item	Item number	Ramkumar et al. (2019)		Hofstetter et al. (2010)		Kokesh, Ferguson, and Patricoski (2011)		Smith et al. (2012)	
		Implementation strategy	Intervention	Implementation strategy	Intervention	Implementation strategy	Intervention	Implementation strategy	Intervention
Title	1	✓		X		X		?	
Abstract	2	✓		X		X		?	
Introduction	3	✓		✓		✓		✓	
Rationale	4	X	✓	X	✓	X	✓	X	X
Aims and objectives	5	?		✓		X		?	
Design	6	?		?		?		?	
Context	7	✓		X		X		X	
Targeted 'sites'	8	✓	✓	✓	✓	✓	✓	✓	✓
Description	9	✓	✓	✓	✓	✓	✓	✓	✓
Sub-groups	10	N/A		N/A		✓		N/A	
Outcomes	11	✓	✓	✓	✓	✓	✓	✓	✓
Process evaluation	12	X		X		✓		X	
Economic evaluation	13	✓	✓	✓	✓	✓	✓	X	X
Sample size	14	✓		?		?		?	
Analysis	15	✓		✓		✓		✓	
Sub-group analyses	16	N/A		N/A		✓		N/A	
Characteristics	17	✓	✓	✓	✓	✓	✓	✓	✓
Outcomes	18	✓	✓	✓	✓	✓	✓	✓	✓
Process outcomes	19	X		X		✓		X	
Economic evaluation	20	✓	✓	✓	✓	✓	✓	X	X
Sub-group analyses	21	N/A		N/A		✓		N/A	
Fidelity/ adaptation	22	?	?	X	X	X	X	X	X
Contextual changes	23	✓		X		✓		X	
Harms	24	N/A		N/A		N/A		N/A	
Structured discussion	25	✓		✓		✓		✓	
Implications	26	?	?	X	X	?	?	X	X
Statements	27	✓		✓		✓		✓	

Appendix 7 - StaRI Checklist

Checklist item	Item number	Smith et al. (2013)		Smith et al. (2015)		Gupta et al. (2020)		Ravi et al. (2020)	
		**Implementation strategy**	**Intervention**	**Implementation strategy**	**Intervention**	**Implementation strategy**	**Intervention**	**Implementation strategy**	**Intervention**
Title	**1**	✓		?		X		X	
Abstract	**2**	✓		?		?		✓	
Introduction	**3**	✓		✓		✓		✓	
Rationale	**4**	X	X	X	X	X	✓	X	✓
Aims and objectives	**5**	?		?		?		✓	
Design	**6**	?		?		?		?	
Context	**7**	X		X		?		?	
Targeted 'sites'	**8**	?	?	?	?	✓	✓	✓	✓
Description	**9**	✓	✓	?	?	✓	✓	✓	✓
Sub-groups	**10**	N/A		✓		N/A		N/A	
Outcomes	**11**	X	✓	X	✓	X	✓	✓	✓
Process evaluation	**12**	X		X		✓		?	
Economic evaluation	**13**	X	X	X	X	X	X	✓	✓
Sample size	**14**	?		?		?		✓	
Analysis	**15**	✓		✓		✓		✓	
Sub-group analyses	**16**	N/A		✓		N/A		N/A	
Characteristics	**17**	X	✓	X	✓	✓	✓	✓	✓
Outcomes	**18**	X	✓	X	✓	X	✓	✓	✓
Process outcomes	**19**	X		X		✓		?	
Economic evaluation	**20**	X	X	X	X	X	X	✓	✓
Sub-group analyses	**21**	N/A		✓		N/A		N/A	
Fidelity/ adaptation	**22**	X	X	X	X	✓	X		
Contextual changes	**23**	X		X		✓		✓	
Harms	**24**	N/A		N/A		N/A		N/A	
Structured discussion	**25**	✓		✓		✓		✓	
Implications	**26**	X	X	X	X	X	X	X	X
Statements	**27**	✓		✓		✓		✓	

Appendix 7 - StaRI Checklist

<table>
<tr><th rowspan="2">Checklist item</th><th rowspan="2">Item number</th><th colspan="2">Dennis, Gladden, and Noe (2012)</th><th colspan="2">Novak et al. (2016)</th><th colspan="2">Luryi et al. (2020)</th><th colspan="2">Skarżyński et al. (2018)</th></tr>
<tr><th>Implementation strategy</th><th>Intervention</th><th>Implementation strategy</th><th>Intervention</th><th>Implementation strategy</th><th>Intervention</th><th>Implementation strategy</th><th>Intervention</th></tr>
<tr><td>Title</td><td>1</td><td colspan="2">X</td><td colspan="2">X</td><td colspan="2">✓</td><td colspan="2">X</td></tr>
<tr><td>Abstract</td><td>2</td><td colspan="2">X</td><td colspan="2">X</td><td colspan="2">X</td><td colspan="2">✓</td></tr>
<tr><td>Introduction</td><td>3</td><td colspan="2">✓</td><td colspan="2">✓</td><td colspan="2">✓</td><td colspan="2">✓</td></tr>
<tr><td>Rationale</td><td>4</td><td>X</td><td>✓</td><td>X</td><td>✓</td><td>X</td><td>✓</td><td>X</td><td>✓</td></tr>
<tr><td>Aims and objectives</td><td>5</td><td colspan="2">✓</td><td colspan="2">✓</td><td colspan="2">?</td><td colspan="2">X</td></tr>
<tr><td>Design</td><td>6</td><td colspan="2">?</td><td colspan="2">?</td><td colspan="2">?</td><td colspan="2">?</td></tr>
<tr><td>Context</td><td>7</td><td colspan="2">✓</td><td colspan="2">✓</td><td colspan="2">?</td><td colspan="2">?</td></tr>
<tr><td>Targeted ‘sites’</td><td>8</td><td>✓</td><td>✓</td><td>✓</td><td>✓</td><td>?</td><td>✓</td><td>✓</td><td>✓</td></tr>
<tr><td>Description</td><td>9</td><td>✓</td><td>✓</td><td>✓</td><td>✓</td><td>?</td><td>✓</td><td>✓</td><td>✓</td></tr>
<tr><td>Sub-groups</td><td>10</td><td colspan="2">N/A</td><td colspan="2">N/A</td><td colspan="2">N/A</td><td colspan="2">N/A</td></tr>
<tr><td>Outcomes</td><td>11</td><td>✓</td><td>✓</td><td>✓</td><td>✓</td><td>?</td><td>✓</td><td>X</td><td>X</td></tr>
<tr><td>Process evaluation</td><td>12</td><td colspan="2">X</td><td colspan="2">✓</td><td colspan="2">✓</td><td colspan="2">✓</td></tr>
<tr><td>Economic evaluation</td><td>13</td><td>✓</td><td>✓</td><td>X</td><td>X</td><td>X</td><td>X</td><td>X</td><td>X</td></tr>
<tr><td>Sample size</td><td>14</td><td colspan="2">✓</td><td colspan="2">✓</td><td colspan="2">?</td><td colspan="2">✓</td></tr>
<tr><td>Analysis</td><td>15</td><td colspan="2">✓</td><td colspan="2">✓</td><td colspan="2">✓</td><td colspan="2">✓</td></tr>
<tr><td>Sub-group analyses</td><td>16</td><td colspan="2">N/A</td><td colspan="2">N/A</td><td colspan="2">N/A</td><td colspan="2">N/A</td></tr>
<tr><td>Characteristics</td><td>17</td><td>✓</td><td>✓</td><td>✓</td><td>✓</td><td>?</td><td>✓</td><td>X</td><td>✓</td></tr>
<tr><td>Outcomes</td><td>18</td><td>✓</td><td>✓</td><td>✓</td><td>✓</td><td>?</td><td>✓</td><td>X</td><td>X</td></tr>
<tr><td>Process outcomes</td><td>19</td><td colspan="2">X</td><td colspan="2">✓</td><td colspan="2">✓</td><td colspan="2">✓</td></tr>
<tr><td>Economic evaluation</td><td>20</td><td>✓</td><td>✓</td><td>X</td><td>X</td><td>X</td><td>X</td><td>X</td><td>X</td></tr>
<tr><td>Sub-group analyses</td><td>21</td><td colspan="2">N/A</td><td colspan="2">N/A</td><td colspan="2">N/A</td><td colspan="2">N/A</td></tr>
<tr><td>Fidelity/ adaptation</td><td>22</td><td>X</td><td>X</td><td>X</td><td>X</td><td>X</td><td>X</td><td>X</td><td>X</td></tr>
<tr><td>Contextual changes</td><td>23</td><td colspan="2">✓</td><td colspan="2">X</td><td colspan="2">✓</td><td colspan="2">✓</td></tr>
<tr><td>Harms</td><td>24</td><td colspan="2">N/A</td><td colspan="2">N/A</td><td colspan="2">N/A</td><td colspan="2">N/A</td></tr>
<tr><td>Structured discussion</td><td>25</td><td colspan="2">✓</td><td colspan="2">✓</td><td colspan="2">✓</td><td colspan="2">✓</td></tr>
<tr><td>Implications</td><td>26</td><td>✓</td><td>✓</td><td>✓</td><td>✓</td><td>?</td><td>✓</td><td>✓</td><td>✓</td></tr>
<tr><td>Statements</td><td>27</td><td colspan="2">?</td><td colspan="2">✓</td><td colspan="2">✓</td><td colspan="2">✓</td></tr>
</table>

Appendix 7 - StaRI Checklist

<table>
<tr><th rowspan="2">Checklist item</th><th rowspan="2">Item number</th><th colspan="2">Skarżyński et al. (2019)</th><th colspan="2">Houston (2011)</th><th colspan="2">Blaiser et al. (2012)</th></tr>
<tr><th>Implementation strategy</th><th>Intervention</th><th>Implementation strategy</th><th>Intervention</th><th>Implementation strategy</th><th>Intervention</th></tr>
<tr><td>Title</td><td>1</td><td colspan="2">X</td><td colspan="2">X</td><td colspan="2">X</td></tr>
<tr><td>Abstract</td><td>2</td><td colspan="2">X</td><td colspan="2">X</td><td colspan="2">X</td></tr>
<tr><td>Introduction</td><td>3</td><td colspan="2">✓</td><td colspan="2">✓</td><td colspan="2">✓</td></tr>
<tr><td>Rationale</td><td>4</td><td>X</td><td>✓</td><td>X</td><td>✓</td><td>X</td><td>✓</td></tr>
<tr><td>Aims and objectives</td><td>5</td><td colspan="2">?</td><td colspan="2">?</td><td colspan="2">?</td></tr>
<tr><td>Design</td><td>6</td><td colspan="2">?</td><td colspan="2">?</td><td colspan="2">?</td></tr>
<tr><td>Context</td><td>7</td><td colspan="2">?</td><td colspan="2">?</td><td colspan="2">?</td></tr>
<tr><td>Targeted ‘sites’</td><td>8</td><td>✓</td><td>✓</td><td>✓</td><td>✓</td><td>✓</td><td>✓</td></tr>
<tr><td>Description</td><td>9</td><td>✓</td><td>✓</td><td>✓</td><td>✓</td><td>✓</td><td>✓</td></tr>
<tr><td>Sub-groups</td><td>10</td><td colspan="2">N/A</td><td colspan="2">N/A</td><td colspan="2">N/A</td></tr>
<tr><td>Outcomes</td><td>11</td><td>X</td><td>X</td><td>X</td><td>X</td><td>X</td><td>X</td></tr>
<tr><td>Process evaluation</td><td>12</td><td colspan="2">✓</td><td colspan="2">X</td><td colspan="2">✓</td></tr>
<tr><td>Economic evaluation</td><td>13</td><td>X</td><td>X</td><td>X</td><td>X</td><td>X</td><td>X</td></tr>
<tr><td>Sample size</td><td>14</td><td colspan="2">✓</td><td colspan="2">X</td><td colspan="2">X</td></tr>
<tr><td>Analysis</td><td>15</td><td colspan="2">✓</td><td colspan="2">X</td><td colspan="2">X</td></tr>
<tr><td>Sub-group analyses</td><td>16</td><td colspan="2">N/A</td><td colspan="2">N/A</td><td colspan="2">N/A</td></tr>
<tr><td>Characteristics</td><td>17</td><td>X</td><td>✓</td><td>✓</td><td>✓</td><td>✓</td><td>✓</td></tr>
<tr><td>Outcomes</td><td>18</td><td>X</td><td>X</td><td>X</td><td>X</td><td>X</td><td>X</td></tr>
<tr><td>Process outcomes</td><td>19</td><td colspan="2">✓</td><td colspan="2">X</td><td colspan="2">✓</td></tr>
<tr><td>Economic evaluation</td><td>20</td><td>X</td><td>X</td><td>X</td><td>X</td><td>X</td><td>X</td></tr>
<tr><td>Sub-group analyses</td><td>21</td><td colspan="2">N/A</td><td colspan="2">N/A</td><td colspan="2">N/A</td></tr>
<tr><td>Fidelity/ adaptation</td><td>22</td><td>X</td><td>X</td><td>X</td><td>X</td><td>X</td><td>X</td></tr>
<tr><td>Contextual changes</td><td>23</td><td colspan="2">✓</td><td colspan="2">✓</td><td colspan="2">✓</td></tr>
<tr><td>Harms</td><td>24</td><td colspan="2">N/A</td><td colspan="2">N/A</td><td colspan="2">N/A</td></tr>
<tr><td>Structured discussion</td><td>25</td><td colspan="2">✓</td><td colspan="2">✓</td><td colspan="2">✓</td></tr>
<tr><td>Implications</td><td>26</td><td>✓</td><td>X</td><td>X</td><td>X</td><td>✓</td><td>✓</td></tr>
<tr><td>Statements</td><td>27</td><td colspan="2">✓</td><td colspan="2">?</td><td colspan="2">?</td></tr>
</table>

Appendix 7 - StaRI Checklist

Checklist item	Item number	Houston and Stredler-Brown (2012)		Broekelmann (2012)		Lalios (2012)		Galvan, Case, and Todd Houston (2014)	
		Implementation strategy	Intervention	Implementation strategy	Intervention	Implementation strategy	Intervention	Implementation strategy	Intervention
Title	1	X		X		X		X	
Abstract	2	X		X		X		X	
Introduction	3	✓		✓		✓		✓	
Rationale	4	X	✓	X	✓	X	✓	X	✓
Aims and objectives	5	?		?		?		?	
Design	6	?		?		?		?	
Context	7	?		?		?		?	
Targeted ‘sites’	8	✓	✓	✓	✓	✓	✓	✓	✓
Description	9	✓	✓	✓	✓	✓	✓	✓	✓
Sub-groups	10	N/A		N/A		N/A		N/A	
Outcomes	11	X	X	X	X	X	X	X	X
Process evaluation	12	✓		✓		✓		X	
Economic evaluation	13	X	X	X	X	X	X	X	X
Sample size	14	X		X		X		X	
Analysis	15	X		X		X		X	
Sub-group analyses	16	N/A		N/A		N/A		N/A	
Characteristics	17	✓	✓	✓	✓	✓	✓	✓	✓
Outcomes	18	X	X	X	X	X	X	X	X
Process outcomes	19	X		X		✓		X	
Economic evaluation	20	X	X	X	X	X	X	X	X
Sub-group analyses	21	N/A		N/A		N/A		N/A	
Fidelity/ adaptation	22	X	X	X	X	X	X	X	X
Contextual changes	23	✓		✓		✓		✓	
Harms	24	N/A		N/A		N/A		N/A	
Structured discussion	25	✓		✓		✓		✓	
Implications	26	X	X	✓	✓	X	✓	X	X
Statements	27	?		?		?		?	

Appendix 7 - StaRI Checklist

<table>
<tr><th rowspan="2">Checklist item</th><th rowspan="2">Item number</th><th colspan="2">McCarthy, Muñoz, and White (2010)</th><th colspan="2">McCarthy (2011)</th><th colspan="2">McCarthy (2012)</th><th colspan="2">Gladden, Beck, and Chandler (2015)</th></tr>
<tr><th>Implementation strategy</th><th>Intervention</th><th>Implementation strategy</th><th>Intervention</th><th>Implementation strategy</th><th>Intervention</th><th>Implementation strategy</th><th>Intervention</th></tr>
<tr><td>Title</td><td>1</td><td colspan="2">X</td><td colspan="2">X</td><td colspan="2">X</td><td colspan="2">?</td></tr>
<tr><td>Abstract</td><td>2</td><td colspan="2">X</td><td colspan="2">X</td><td colspan="2">X</td><td colspan="2">?</td></tr>
<tr><td>Introduction</td><td>3</td><td colspan="2">✓</td><td colspan="2">✓</td><td colspan="2">✓</td><td colspan="2">✓</td></tr>
<tr><td>Rationale</td><td>4</td><td>X</td><td>✓</td><td>X</td><td>✓</td><td>X</td><td>✓</td><td>X</td><td>✓</td></tr>
<tr><td>Aims and objectives</td><td>5</td><td colspan="2">?</td><td colspan="2">?</td><td colspan="2">?</td><td colspan="2">✓</td></tr>
<tr><td>Design</td><td>6</td><td colspan="2">?</td><td colspan="2">?</td><td colspan="2">?</td><td colspan="2">?</td></tr>
<tr><td>Context</td><td>7</td><td colspan="2">?</td><td colspan="2">✓</td><td colspan="2">?</td><td colspan="2">✓</td></tr>
<tr><td>Targeted ‘sites’</td><td>8</td><td>✓</td><td>✓</td><td>✓</td><td>✓</td><td>✓</td><td>✓</td><td>✓</td><td>✓</td></tr>
<tr><td>Description</td><td>9</td><td>✓</td><td>✓</td><td>✓</td><td>✓</td><td>✓</td><td>✓</td><td>✓</td><td>✓</td></tr>
<tr><td>Sub-groups</td><td>10</td><td colspan="2">N/A</td><td colspan="2">N/A</td><td colspan="2">N/A</td><td colspan="2">✓</td></tr>
<tr><td>Outcomes</td><td>11</td><td>X</td><td>X</td><td>✓</td><td>X</td><td>✓</td><td>X</td><td>✓</td><td>✓</td></tr>
<tr><td>Process evaluation</td><td>12</td><td colspan="2">X</td><td colspan="2">✓</td><td colspan="2">✓</td><td colspan="2">✓</td></tr>
<tr><td>Economic evaluation</td><td>13</td><td>X</td><td>X</td><td>X</td><td>X</td><td>X</td><td>X</td><td>X</td><td>X</td></tr>
<tr><td>Sample size</td><td>14</td><td colspan="2">X</td><td colspan="2">X</td><td colspan="2">X</td><td colspan="2">✓</td></tr>
<tr><td>Analysis</td><td>15</td><td colspan="2">X</td><td colspan="2">X</td><td colspan="2">X</td><td colspan="2">✓</td></tr>
<tr><td>Sub-group analyses</td><td>16</td><td colspan="2">N/A</td><td colspan="2">N/A</td><td colspan="2">N/A</td><td colspan="2">✓</td></tr>
<tr><td>Characteristics</td><td>17</td><td>✓</td><td>✓</td><td>✓</td><td>✓</td><td>✓</td><td>✓</td><td>✓</td><td>✓</td></tr>
<tr><td>Outcomes</td><td>18</td><td>X</td><td>X</td><td>✓</td><td>X</td><td>✓</td><td>X</td><td>✓</td><td>✓</td></tr>
<tr><td>Process outcomes</td><td>19</td><td colspan="2">X</td><td colspan="2">✓</td><td colspan="2">✓</td><td colspan="2">✓</td></tr>
<tr><td>Economic evaluation</td><td>20</td><td>X</td><td>X</td><td>X</td><td>X</td><td>X</td><td>X</td><td>X</td><td>X</td></tr>
<tr><td>Sub-group analyses</td><td>21</td><td colspan="2">N/A</td><td colspan="2">N/A</td><td colspan="2">N/A</td><td colspan="2">✓</td></tr>
<tr><td>Fidelity/ adaptation</td><td>22</td><td>X</td><td>X</td><td>X</td><td>X</td><td>X</td><td>X</td><td>✓</td><td>X</td></tr>
<tr><td>Contextual changes</td><td>23</td><td colspan="2">✓</td><td colspan="2">✓</td><td colspan="2">✓</td><td colspan="2">✓</td></tr>
<tr><td>Harms</td><td>24</td><td colspan="2">N/A</td><td colspan="2">N/A</td><td colspan="2">N/A</td><td colspan="2">N/A</td></tr>
<tr><td>Structured discussion</td><td>25</td><td colspan="2">✓</td><td colspan="2">✓</td><td colspan="2">✓</td><td colspan="2">✓</td></tr>
<tr><td>Implications</td><td>26</td><td>✓</td><td>✓</td><td>✓</td><td>✓</td><td>✓</td><td>✓</td><td>✓</td><td>✓</td></tr>
<tr><td>Statements</td><td>27</td><td colspan="2">?</td><td colspan="2">?</td><td colspan="2">?</td><td colspan="2">?</td></tr>
</table>

<table>
<tr><th rowspan="2">Checklist item</th><th rowspan="2">Item number</th><th colspan="2">Ratanjee-Vanmali, Swanepoel, and Laplante-Lévesque (2020)</th></tr>
<tr><th>Implementation strategy</th><th>Intervention</th></tr>
<tr><td>Title</td><td>1</td><td colspan="2">X</td></tr>
<tr><td>Abstract</td><td>2</td><td colspan="2">X</td></tr>
<tr><td>Introduction</td><td>3</td><td colspan="2">✓</td></tr>
<tr><td>Rationale</td><td>4</td><td>X</td><td>✓</td></tr>
<tr><td>Aims and objectives</td><td>5</td><td colspan="2">✓</td></tr>
<tr><td>Design</td><td>6</td><td colspan="2">?</td></tr>
<tr><td>Context</td><td>7</td><td colspan="2">?</td></tr>
<tr><td>Targeted ‘sites’</td><td>8</td><td>X</td><td>✓</td></tr>
<tr><td>Description</td><td>9</td><td>X</td><td>✓</td></tr>
<tr><td>Sub-groups</td><td>10</td><td colspan="2">N/A</td></tr>
<tr><td>Outcomes</td><td>11</td><td>X</td><td>✓</td></tr>
<tr><td>Process evaluation</td><td>12</td><td colspan="2">X</td></tr>
<tr><td>Economic evaluation</td><td>13</td><td>X</td><td>X</td></tr>
<tr><td>Sample size</td><td>14</td><td colspan="2">✓</td></tr>
<tr><td>Analysis</td><td>15</td><td colspan="2">✓</td></tr>
<tr><td>Sub-group analyses</td><td>16</td><td colspan="2">N/A</td></tr>
<tr><td>Characteristics</td><td>17</td><td>X</td><td>✓</td></tr>
<tr><td>Outcomes</td><td>18</td><td>X</td><td>✓</td></tr>
<tr><td>Process outcomes</td><td>19</td><td colspan="2">X</td></tr>
<tr><td>Economic evaluation</td><td>20</td><td>X</td><td>X</td></tr>
<tr><td>Sub-group analyses</td><td>21</td><td colspan="2">N/A</td></tr>
<tr><td>Fidelity/ adaptation</td><td>22</td><td>X</td><td>X</td></tr>
<tr><td>Contextual changes</td><td>23</td><td colspan="2">✓</td></tr>
<tr><td>Harms</td><td>24</td><td colspan="2">N/A</td></tr>
<tr><td>Structured discussion</td><td>25</td><td colspan="2">✓</td></tr>
<tr><td>Implications</td><td>26</td><td>X</td><td>X</td></tr>
<tr><td>Statements</td><td>27</td><td colspan="2">✓</td></tr>
</table>

Supplemental file - 5
Implementation Factors influencing the sustained provision of tele-audiology projects – mapped to Implementation Outcomes Framework (Proctor et al., 2011)

Themes	Facilitators
**Acceptability**	**Patient's perspectives:**<br>• ***Improved accessibility to service provider and multiple locations (P19) (P8) (P8) (P5)***<br>• ***Reduced waiting time and test time (P7) (P19) (P19) (P8) (PS2)***<br>• ***Close proximity/ Reasonable/ Reduced travel time to testing facility (P19) (P14) (P5) (P14)***<br>• Elimination of occupational health and safety issues related to travel (P19)<br>• ***Consistency and regularity of sessions, reduced cancellations (P15) and thereby completion of testing with minimal visits (P14)***<br>• Improved quality of life (P14)<br>• Implemented in the child's natural environment and preparing children with hearing loss for inclusion in their community, home, or school (P16)<br>• ***Comfortable with the environment and communicating in remote assembly settings (P20) (P14) (P5)***<br>• Clinician engagement: introduction to lead audiologist, personalized care, confidence, knowledge and understanding (P3)<br>• ***Families strongly recommended to visit for face-to-face consultation prior to telepractice sessions; Either family travels to consultation site or Audiologist travels to remote site (P19) (P5)***<br>• ***Provision of hybrid model as an option to hesitant parents (P15) (P18)***<br>• ***Counseling, emotional support and reassurance via videoconferencing (P19) (P20) (P18)***<br>• ***Early-intervention specialist is able to directly observe the parent-child interactions and provide responsive feedback that can be applied immediately (P19) (P19) (P8)***<br>• ***Participation and assistance from other family members is encouraged which thereby enables empowerment of parents as primary participants (P19) (P15) (P15)***<br>• Parents and caregivers readily assume their role as the primary facilitator of their child's development, and subsequently parental abilities appear to improve at a faster rate (P17)<br>• ***Presence and motivation of a local reliable and accessible VHW/ technical staff (P19) (P8)***<br>• ***A safe, secure environment is necessary to maintain a trusting and effective therapeutic relationship since the patient needs to have a sense of security and their privacy respected (P16) (P5)***<br>• Effective parent-professional partnership can be established and maintained using this delivery model (P17)<br>• Families developing greater understanding, knowledge and confidence of child's condition (P19)<br>• High quality consultation experience (P20)

For peer review only

	• Having a support network reduces the feelings of isolation experienced by families and provides a continuing sense of solidarity for families (P19)<br>• Interfere with the rapport and personal interaction (P11)<br>• Additional effort required to learn technology and might be intimidated by technology (P15)<br>• Contact with a personnel was not as good as during a traditional visit (P14)<br>• Lip reading cues can be difficult to perceive via telepractice and additional supports may be necessary for these parents (P19)
**Adoption**	**Provider's perspectives:**<br>• Interest among the personnel to implement the program (P12)<br>• Early intervention and cultural shifts in attitude (P20)<br>• Reducing loss to follow up (P4)<br>• Increasing the efficacy, efficiency, and equity of the EHDI program is a motivator for providers (P4)<br>• Audiologist promoting technology literacy (P21)<br>• Increased awareness regarding hearing (P6)<br>• Self-referral by older adults for hearing testing (P6)<br>• Requests for testing hearing of older siblings (P6)<br>• Planning sessions takes time (P15)
**Feasibility**	**Human resources:**<br>• Tele Practitioner should have heightened awareness of anticipation and remote management skills to assist in obtaining the learning objectives of both the parent and child (P17)<br>• Audiologist needs to take a lead role in the development, implementation and refinement of the service model (P5)<br>• ***Collaboration, coordination and communication between all personnel involved can result in reliable patient outcomes (P11) (P12) (P5)***<br>• ***Designated personnel for each role; such as IT Specialist/ Tele-technician (P6) (P15)***<br>• The onus is entrusted to the personnel recruited (P6)<br>• Community health worker and coordinator must have good interpersonal communication skills and knowledge of the local language (P9)<br>• Training conducted in local language using demonstrations and handouts (P6)<br>• Knowledge and skill retention assessed periodically post-training in a structured format (P6)<br>• Regular training and supervision is recommended to improve health workers' ability to successfully screen (P6)<br>• ***Stationary, video training and detailed training on the equipment for complex procedures (P12) (P14)***<br>• Flexibility to adapt materials according to need (P12)<br>• Personnel have to adapt and problem-solve in real time during various situations (P12)<br>• Lack of trained tele-technicians, shortage of professionals (P15)

For peer review only - http://bmjopen.bmj.com/site/about/guidelines.xhtml

	• Need for faculty full-time equivalency (P12)<br><br>**<u>Infrastructure - Provider:</u>**<br>• Mobile telemedicine van with satellite connection and built in facilities for video conferencing, testing and A/C (P6)<br>• Generous space acquisition and infrastructure support (P12)<br>• Appropriate lighting and sound with limited environmental distractions as well as adequate physical space for parent, child, and materials within view of the camera is mandated (P17)<br>• Physical limitations of operating in a sound booth (P5)<br>• Small space, low light conditions (P5)<br><br>**<u>Organization Acceptance:</u>**<br>• The involvement of audiology graduate students in training (P3)<br>• High level administrative support (University presidents, deans, and department chairs) (P12)<br><br>**<u>Planning and implementation:</u>**<br>• ***Assessment of patient needs and internal resources for program start-up and sustainability (PS3) (P20)***<br>• ***A robust business model/plan/ proposal, internal and external needs assessment is critical that is unique to the project (P20) (P9)***<br>• ***A site-visit, first visit, pre-feasibility analysis, barrier study could be useful (P9) (P5)***<br>• ***A test run / test environment to simulate operational conditions and validate functional requirements (P18) (P5)***<br>• A pilot project at the remote site to assess operability and workflow issues (P5)<br>• Optimise existing infrastructure and personnel (P5)<br>• A service-level analysis of averted costs from the perspective of the provider to determine the allocation efficiency (P5)<br>• Systematic framework for collecting data, that monitors program satisfaction and tracking the efficacy of the program (P16)<br>• Careful planning and coordination (P11)<br>• Support and buy-in (ownership) from clinical staff (audiologists and other related professionals) and organizational leaders (P20)<br>• Development of an enterprise consciousness that includes systems-thinking and broader concerns beyond a single program (P20)<br>• A systematic nature of programme implementation, can enable surveillance capability which allows to monitor changes over time and be more responsive to changing health needs in the community (P8)<br>• Development of protocols, standards of care, best practices, and toolkits to guide implementation (P20)

For peer review only

	• Targeted evaluation plan (P20)<br>• Openness to feedback and flexibility that allows for modification of work processes (P20)<br>• Embrace and lead a transformational period with the highest level of integrity (P20)<br>• Conduct formal assessments every 6 months to monitor patient progress (P19)<br>• Prioritize ease of use and affordability (P1)<br>• Business process interoperability (common policies and support structures) need to be achieved across health authorities (P5)<br>• Absence of a solid model for telemedicine cost analysis (P3)<br><br>**Resource management**<br>• Involvement of all stakeholders for the design of service delivery model (P5)<br>• Determination of resources to achieve standardization, interoperability of technology, and connectivity (P20)<br>• ***Memorandum of Understanding (MOU), Telehealth Service Agreement (TSA), A letter of agreement that delineates project responsibilities, equipment, supply, cost and work sharing between stakeholders (P11) (P5)***<br>• Commitment to adequate resourcing, staff education and training (P20)<br>• Strong partnerships with industry, technical for resource management(information technology, biomedical), and administration staff (P20)<br>• Incorporate aspects of social networking as well as blogging and facilitates collaboration, information sharing, and parent training (P19)<br>• Use of creative and focused lesson plans (P16)<br>• Equipment re-allocation as families leave the program (P19)<br>• EMR system with digital flow sheets, which enables documentation of audiometric, hearing health history and hearing or communication handicap data (P12)<br>• Online options for HHC may be important avenues for adults with hearing loss (P21)<br><br>**Standard Operating Protocol:**<br>• ***Materials and educational package necessary for instruction sent to the families via the postal service or over the Internet (P19) (P15) (P18)***<br>• Documentation of multimedia data for future reference using store and forward telepractice (P7)<br>• ***Electronic data integration into electronic health records (P7) (P20)***<br>• Tracking of cases for time studies and administrative purposes (P7)<br>• ***Use of strict inclusion and candidature criteria (P15) (P18)***

For peer review only

	• Unique identifier (UIN) on all forms and communications to allow for data linkage across sources (P1)<br>• Monitor the use of telepractice program through chart reviews in the multi organisational electronic medical record (EMR) (P1)<br>• On-going assessments and surveys (P15)<br><br>**Logistics:**<br>• Flexibility for rescheduling (P19)<br>• Accommodates for time differences by offering extended operating hours (P19)<br>• When circumstances results in cancelled appointments, personnel can seamlessly shift their attention to other clinic priorities (P5)<br>• Problem solving between the audiometric technician and audiologist can be conducted with relative ease due to consistency between multiple sites (P5)<br>• Referrals managed within fewer sessions and with an overall shorter duration of contact events<br>• Flexibility to conduct testing at different sites (P10)<br><br>**Sociocultural aspects:**<br>• Mobilizing community participation and monitoring follow up (P6)<br>• Family and student interpreters for different languages (P12)<br>• Community involvement, participation and engagement (P1)<br>• ***Continuous and on-going consultation and collaboration with local council and community in an appropriate and culturally safe manner (P8) (P8) (P8) (P1)***<br>• ***Strong indigenous leadership (P8) (P8) (P1)***<br>• ***Close alignment with primary care services in the community (P8) (P8)***<br>• Collaboration with the family's local birth-to-3 program enabled a computer to be purchased through an early intervention family grant program (P17)<br><br>**Government laws:**<br>• A state/ National database to document, maintain records, track and follow up (P3)<br>• Not covered by practice acts and state laws (P11)<br>• Not being covered by malpractice liability (P11)<br>• Challenges with providing practitioners with credentials and privileges (P11)<br><br>**Privacy and confidentiality - Provider:**<br>• Secured and encrypted virtual private network (P13)

For peer review only

	• The client file is maintained under that agency's privacy policies and the applicable audiology governing policy. The host site may track the number of tests performed for statistical and billing purposes, but does not record client information (P4)<br>• Including biometric records - fingerprints, retina and iris patterns, voice waves, signatures, and pictures (P16)
**Implementation cost**	**Provider:**<br>• Having multiple sources of funding could be useful; including charitable donations and government (P19) (P20)<br>• Lower total cost outcome (P10)<br>• Funding for hiring IT support<br>• Services not reimbursed by insurance in some cases (P11)<br><br>**Patient/Beneficiary:**<br>• ***Reduced cost of travelling and testing (P12) (P14) (P14)***<br>• Financial assistance for travel expenses and free on-site accommodation for initial face-to-face consultation (P19)

References:

Proctor, E., Silmere, H., Raghavan, R., Hovmand, P., Aarons, G., Bunger, A., Griffey, R., & Hensley, M. (2011). Outcomes for implementation research: Conceptual distinctions, measurement challenges, and research agenda. *Administration and Policy in Mental Health and Mental Health Services Research*, *38*(2), 65–76. https://doi.org/10.1007/s10488-010-0319-7

For peer review only

Supplemental file 6
COREQ (COnsolidated criteria for REporting Qualitative research) Checklist

A checklist of items that should be included in reports of qualitative research. You must report the page number in your manuscript where you consider each of the items listed in this checklist. If you have not included this information, either revise your manuscript accordingly before submitting or note N/A.

Topic	Item No.	Guide Questions/Description	Reported on Page No.
**Domain 1: Research team and reflexivity**			
Personal characteristics			
Interviewer/facilitator	1	Which author/s conducted the interview or focus group?	7
Credentials	2	What were the researcher's credentials? E.g. PhD, MD	1
Occupation	3	What was their occupation at the time of the study?	1
Gender	4	Was the researcher male or female?	N/A
Experience and training	5	What experience or training did the researcher have?	7
Relationship with Participants			
Relationship established	6	Was a relationship established prior to study commencement?	7
Participant knowledge of the interviewer	7	What did the participants know about the researcher? e.g. personal goals, reasons for doing the research	7
Interviewer characteristics	8	What characteristics were reported about the inter viewer/facilitator? e.g. Bias, assumptions, reasons and interests in the research topic	7
**Domain 2: Study design**			
Theoretical framework			
Methodological orientation and Theory	9	What methodological orientation was stated to underpin the study? e.g. grounded theory, discourse analysis, ethnography, phenomenology, content analysis	7
Participant selection			
Sampling	10	How were participants selected? e.g. purposive, convenience, consecutive, snowball	7
Method of approach	11	How were participants approached? e.g. face-to-face, telephone, mail, email	7
Sample size	12	How many participants were in the study?	7
Non-participation	13	How many people refused to participate or dropped out? Reasons?	7
Setting			
Setting of data collection	14	Where was the data collected? e.g. home, clinic, workplace	7
Presence of non-participants	15	Was anyone else present besides the participants and researchers?	7
Description of sample	16	What are the important characteristics of the sample? e.g. demographic data, date	7
Data collection			
Interview guide	17	Were questions, prompts, guides provided by the authors? Was it pilot tested?	8
Repeat interviews	18	Were repeat inter views carried out? If yes, how many?	8
Audio/visual recording	19	Did the research use audio or visual recording to collect the data?	8
Field notes	20	Were field notes made during and/or after the inter view or focus group?	8
Duration	21	What was the duration of the inter views or focus group?	8
Data saturation	22	Was data saturation discussed?	8

For peer review only - http://bmjopen.bmj.com/site/about/guidelines.xhtml

Transcripts returned	23	Were transcripts returned to participants for comment and/or	8

For peer review only

Topic	Item No.	Guide Questions/Description	Reported on Page No.
		correction?	
**Domain 3: analysis and findings**			
Data analysis			
Number of data coders	24	How many data coders coded the data?	8
Description of the coding tree	25	Did authors provide a description of the coding tree?	8
Derivation of themes	26	Were themes identified in advance or derived from the data?	8
Software	27	What software, if applicable, was used to manage the data?	8
Participant checking	28	Did participants provide feedback on the findings?	8
Reporting			
Quotations presented	29	Were participant quotations presented to illustrate the themes/findings? Was each quotation identified? e.g. participant number	8
Data and findings consistent	30	Was there consistency between the data presented and the findings?	8
Clarity of major themes	31	Were major themes clearly presented in the findings?	8
Clarity of minor themes	32	Is there a description of diverse cases or discussion of minor themes?	8

Developed from: Tong A, Sainsbury P, Craig J. Consolidated criteria for reporting qualitative research (COREQ): a 32-item checklist for interviews and focus groups. *International Journal for Quality in Health Care*. 2007. Volume 19, Number 6: pp. 349 – 357

Once you have completed this checklist, please save a copy and upload it as part of your submission. DO NOT include thischecklist as part of the main manuscript document. It must be uploaded as a separate file.

For peer review only

Supplemental file 7
Interview Guides

Interview time: _________

Hello Dr./ Mr. /Ms. /Mrs.,
I am Vidya, I am an audiologist and SLP working as a professional since 2008, and have spent all of these years in telepractice for rural community based programs here in India. I am based at SRIHER, Chennai, India.

I thank you for giving consent to participate in the study.

This interview to understand in detail about your experience (both positive and negative), in implementing your project titled_____(project title)______________.

The interview will take between 30-45 minutes to be completed.

Before we begin, I would like to know if you have any questions that I can address/ clarify to you.

We will now begin the interview. I will start recording our conversation for the purpose of transcription and analysis of data.

Interview begins

Dear Dr./ Mr. /Ms. /Mrs.,
From reading your work "___________________", I have gathered that you examined ____
____.

Guides:

1. How would you describe your efforts towards working in the area of telepractice?
E.g as research initiatives to contribute to the understanding of telepractice/ work towards immediate translation to service delivery in your work location/ or anything else?

2. Could you briefly describe the current status of implementation of your telepractice service for individuals with autism?
Sustained/ On-going/ non-viable/ waiting for a,b,c to start as routine?

3. Could you describe the **resources** that influenced (either positively or negatively) the implementation/ attempt to implement the telepractice service?
- Financial/Economic
- Human - professional, e-helpers/ care takers/ technical help/ facilitator
- Infrastructure-
- Equipment/ technology
- Technical/internet

Were there any non-adaptable factors that may have affected the implementation?

4. What about **the end-users** Comfort/ Acceptance/ Perceptions/ Sociocultural?

For peer review only

Motivators/ influencers
Inherent patient related factors?

5. **<u>Organizational</u>**

- Acceptance
- Policy Making/decision
- SOP
- Infrastructure for implementation (e-bills/staff allocation)
- Provider comfort/ ease/ acceptance/ perceptions?

6. **<u>Ethics/ Legality</u>**

- Government laws
- Consent and other ethical considerations

7. **<u>Test specific/ Disorder Specific implementation factors</u>**

8. **<u>Sustainability- self sustaining without external project funding</u>**

For peer review only

Supplemental file 8

Table 1a: Summary of articles identified in tele-audiology

Code	Study title	Authors	Country/ *Area*	Participants	Focus area
A12010	eEHDI : Functions and Challenges	Campbell & Hyde (2010)	Ontario, Canada *Rural areas*	Infants	Diagnostic audiological evaluation
A22010	The impact of telehealth on wait time for ENT specialty care	Hofstetter, Kokesh, Ferguson, & Hood (2010)	Alaska, United States of America *Rural areas*	Mixed Population	Combination of diagnostic audiological and otorhinolaryngological evaluation
A32010	Teleintervention for Infants and Young Children Who Are Deaf or Hard-of-Hearing	McCarthy, Muñoz, & White (2010)	Sydney, Australia *Rural areas*	Children	Aural re/habilitation
A42011	TeleIntervention: Improving Service Delivery to Young Children With Hearing Loss and Their Families Through Telepractice	Houston (2011)	Utah, United States of America *Mixed areas*	Children	Aural re/habilitation
A52011	The Alaska experience using store-and-forward telemedicine for ENT care in Alaska	Kokesh, Ferguson, & Patricoski (2011)	Alaska, United States of America *Rural areas*	Mixed Population	Combination of diagnostic audiological and otorhinolaryngological evaluation
A62011	Using technology to support children with sensory disability in remote areas: the RIDBC Teleschool Model	McCarthy (2011)	Sydney, Australia *Rural areas*	Children	Aural re/habilitation
A72012	Telepractice Services at Sound Beginnings at Utah State University	Blaiser, Edwards, Behl, & Munoz (2012)	Utah, United States of America *Mixed areas*	Children	Aural re/habilitation
A82012	ihear® Internet Therapy Program - A Program by St. Joseph Institute for the Deaf	Broekelmann (2012)	Missouri, United States of America *Rural areas*	Children - Adults	Aural re/habilitation
A92012	Telepractice in the Department of Veterans Affairs	Dennis, Gladden, & Noe (2012)	Washington, United States of America *Mixed areas*	Mixed Population	Hearing aid fitting and programming

For peer review only

A102012	A Model of Early Intervention for Children with Hearing Loss Provided through Telepractice	Houston & Stredler-Brown (2012)	Utah, United States of America<br>*Rural areas*	Children	Aural re/habilitation
A112012	ConnectHear TeleIntervention Program	Lalios (2012)	Wisconsin, United States of America<br>*Mixed areas*	Children - Adolescents	Aural re/habilitation
A122012	RIDBC Teleschool (TM): A hub of expertise	McCarthy (2012)	Sydney, Australia<br>*Rural areas*	Children	Aural re/habilitation
A132012	A mobile telemedicine-enabled ear screening service for Indigenous children in Queensland: activity and outcomes in the first three years	Smith, Armfield, Wu, Brown, & Perry (2012)	Queensland, Australia<br>*Rural areas*	Children	Combination of diagnostic audiological and otorhinolaryngological evaluation
A142013	Changes in paediatric hospital ENT service utilisation following the implementation of a mobile, indigenous health screening service	Smith et al. (2013)	Queensland, Australia<br>*Rural areas*	Children	Combination of diagnostic audiological and otorhinolaryngological evaluation
A152014	Listening and Learning: Using Telepractice to Serve Children and Adults with Hearing Loss	Galvan, Case, & Todd Houston (2014)	Ohio, United States of America<br>*Rural areas*	Mixed Population	Aural re/habilitation
A162015	Tele-audiology: Expanding access to hearing care and enhancing patient connectivity	Gladden, Beck, & Chandler (2015)	Washington, United States of America<br>*Rural areas*	Mixed Population	Comprehensive audiological services
A172015	Monitoring ear health through a telemedicine-supported health screening service in Queensland	Smith et al. (2015)	Queensland, Australia<br>*Rural areas*	Children - Adolescents	Combination of diagnostic audiological and otorhinolaryngological evaluation
A182016	Reducing Loss to Follow-Up with Tele-audiology Diagnostic Evaluations.	Dharmar et al. (2016)	California, United States of America<br>*Rural areas*	Infants	Diagnostic audiological evaluation
A192016	The Future of Healthcare Delivery : IPE / IPP Audiology and Nursing Student / Faculty Collaboration to	Novak, Cantu, Zappler, Coco, & Champlin (2016)	Texas, United States of America<br>*Urban areas*	Mixed Population	Hearing aid fitting and programming

For peer review only

	Deliver Hearing Aids to Vulnerable Adults via Telehealth				
A202016	Infant Diagnostic Evaluation via Teleaudiology Following Newborn Screening in Eastern North Carolina	Stuart (2016)	North Carolina, United States of America<br>*Rural areas*	Infants	Diagnostic audiological evaluation
A212018	Cost and outcome of a community-based paediatric hearing screening programme in rural India with application of tele-audiology for follow-up diagnostic hearing assessment	Ramkumar et al. (2018)	Tamil Nadu, India<br>*Rural areas*	Children	Diagnostic audiological evaluation
A222018	Comprehensive approach to the National Network of Teleaudiology in World Hearing Center in Kajetany, Poland	Skarżyński, Świerniak, Bruski, Ludwikowski, & Skarżyński (2018)	Kajetany, Poland<br>*Rural areas*	Mixed Population	Cochlear Implants fitting and mapping
A232019	Hearing Norton Sound: Mixed methods protocol of a community randomised trial to address childhood hearing loss in rural Alaska	Emmett, Robler, Gallo, et al. (2019)	Alaska, United States of America<br>*Rural areas*	Children - Adolescents	Hearing screening
A242019	Hearing Norton Sound: a community randomised trial protocol to address childhood hearing loss in rural Alaska.	Emmett, Robler, Wang, et al. (2019)	Alaska, United States of America<br>*Rural areas*	Children - Adolescents	Hearing screening
A252019	Comprehensive Community Screening of Otological Patients by Trained Technicians Using a Telemedicine Device: An Efficient and Cost-Effective Way to Triage Patients With Ear Diseases	Gupta et al. (2019)	Delhi, India<br>*Rural areas*	Mixed Population	Combination of diagnostic audiological and otorhinolaryngological evaluation
A262019	Telehealth-enabled auditory brainstem response testing for infants living in rural communities: the British Columbia Early Hearing Program experience	Hatton, Rowlandson, Beers, & Small (2019)	British Columbia, Canada<br>*Rural areas*	Infants	Diagnostic audiological evaluation

For peer review only

A272019	Implementation and evaluation of a rural community-based pediatric hearing screening program integrating in-person and tele-diagnostic auditory brainstem response (ABR)	Ramkumar, Nagarajan, Shankarnarayan, Kumaravelu, & Hall (2019)	Tamil Nadu, India *Rural areas*	Children	Diagnostic audiological evaluation
A282019	Telefitting Between Kajetany, Poland and Odessa, Ukraine for Cochlear Implants	Skarżyński, Swierniak, Ludwikowski, & Bruski (2019)	Kajetany, Poland *Rural areas*	Mixed Population	Cochlear Implant fitting and mapping
A292020	Cochlear Implant Mapping Through Telemedicine—A Feasibility Study	Luryi et al. (2020)	Connecticut, United States of America *Urban areas*	Geriatric	Cochlear Implant fitting and mapping
A302020	Digital Proficiency Is Not a Significant Barrier for Taking Up Hearing Services With a Hybrid Online and Face-to-Face Model	Ratanjee-Vanmali, Swanepoel, & Laplante-Lévesque (2020)	Durban, South Africa *Urban areas*	Adults	Comprehensive audiological services
A312020	Tele-Audiological Surveillance of Middle Ear Status among Individuals with Cleft Lip and/or Palate in Rural South India	Ravi et al. (2020)	Tamil Nadu, India *Rural areas*	Mixed Population	Combination of diagnostic audiological and otorhinolaryngological evaluation
A322020	Hearing Norton Sound: community involvement in the design of a mixed methods community randomized trial in 15 Alaska Native communities	Robler et al. (2020)	Alaska, United States of America *Rural areas*	Children - Adolescents	Hearing screening

For peer review only

Table 1b: Summary of articles identified in tele-audiology (Contd.)

Code	Study title	Authors	Settings	Personnel/ e-helper/telepractice assistant	Type of services
A12010	eEHDI : Functions and Challenges	Campbell & Hyde (2010)	Community clinic	Remote site Technician	Tone specific ABR tracings at 500 and 2000 Hz<br>Diagnostic otoacoustic emissions (OAE) at 1 – 4 kHz
A22010	The impact of telehealth on wait time for ENT specialty care	Hofstetter, Kokesh, Ferguson, & Hood (2010)	Community clinic	Initiating provider:<br>Audiologists<br>Physicians<br>Public health nurses<br>Dentists<br>Physician assistants<br>Staff of the infant learning program<br><br>Consulting provider:<br>ENT Specialist	Patient demographics<br>Clinical data<br>Images and results from diagnostic tests
A32010	Teleintervention for Infants and Young Children Who Are Deaf or Hard-of-Hearing	McCarthy, Muñoz, & White (2010)	Home-based	Specially trained early-intervention specialist	Early intervention services
A42011	TeleIntervention: Improving Service Delivery to Young Children With Hearing Loss and Their Families Through Telepractice	Houston (2011)	Home-based	Speech-language pathologist	AV Therapy: Listening and language skills<br>Parent coaching<br>Family centered practices
A52011	The Alaska experience using store-and-forward telemedicine for ENT care in Alaska	Kokesh, Ferguson, & Patricoski (2011)	Community clinics<br>Hospitals	Health care providers	History<br>Images of the tympanic membrane<br>Audiometry<br>Tympanometry

For peer review only

A62011	Using technology to support children with sensory disability in remote areas: the RIDBC Teleschool Model	McCarthy (2011)	Home-based	RIDBC Teleschool staff - professionals<br>Local professionals<br>Technical support person<br>Local technical staff	Early intervention services
A72012	Telepractice Services at Sound Beginnings at Utah State University	Blaiser, Edwards, Behl, & Munoz (2012)	Home-based	Speech-language pathologist or<br>Educators of the deaf	AV Therapy: Listening and language skills
A82012	ihear® Internet Therapy Program - A Program by St. Joseph Institute for the Deaf	Broekelmann (2012)	Home-based<br>School-based	ihear therapist:<br>Deaf educator/ Speech-language pathologist/<br>Special educator	Effective, individualized, and interactive therapy: listening and spoken language
A92012	Telepractice in the Department of Veterans Affairs	Dennis, Gladden, & Noe (2012)	Community clinics	Clinicians<br>Audiology assistants<br>Telehealth clinical technicians<br>Information technology (IT) experts<br>Biomedical staff	Remote hearing aid programming and verification via telehealth
A102012	A Model of Early Intervention for Children with Hearing Loss Provided through Telepractice	Houston & Stredler-Brown (2012)	Home-based	Speech-language pathologist	AV Therapy: Listening and language skills<br>Parent coaching<br>Family centered practices
A112012	ConnectHear TeleIntervention Program	Lalios (2012)	Home-based	LSLS certified professionals	AV Therapy
A122012	RIDBC Teleschool (TM): A hub of expertise	McCarthy (2012)	Home-based	14 full-time equivalent professionals, including teachers, speech-language pathologists, and audiologists<br><br>Program manager	Rehabilitation and therapy services<br><br>Early Intervention Program<br>School age program<br>Group sessions<br>Preschool/school site visits<br>Audiological assessments

For peer review only

				Administrative/technical assistant<br>Toy librarian<br>Information technology<br>Occupational therapists<br>Audiologists<br>Psychologists	In-person sessions with RIDBC professionals
A132012	A mobile telemedicine-enabled ear screening service for Indigenous children in Queensland: activity and outcomes in the first three years	Smith, Armfield, Wu, Brown, & Perry (2012)	Mobile facilities	Local Indigenous health worker	Patient information and clinical history<br>Digital images using video-otoscope<br>Hearing assessment<br>Health risk questionnaire<br>ENT Review
A142013	Changes in paediatric hospital ENT service utilisation following the implementation of a mobile, indigenous health screening service	Smith et al. (2013)	Mobile facilities	Local Indigenous health worker	Patient information and clinical history<br>Digital images using video-otoscope<br>Hearing assessment<br>Health risk questionnaire<br>ENT Review
A152014	Listening and Learning: Using Telepractice to Serve Children and Adults with Hearing Loss	Galvan, Case, & Todd Houston (2014)	Home-based	Two Listening & Spoken Language Specialists & Certified Auditory Verbal Therapists (LSLS Cert. AVTs)	AV Therapy: Listening and language skills<br>Adult Aural Rehabilitation
A162015	Tele-audiology: Expanding access to hearing care and enhancing patient connectivity	Gladden, Beck, & Chandler (2015)	Community clinics	Trained assistant<br>Teleprovider<br>Telepresenter	Remote Hearing aid programming<br>Remote Audiometry<br>PC-sharing application<br>Remote programming of hearing aids - Smartphone app<br>Automated audiometry<br>Remote Cochlear Implant Mapping

For peer review only

A172015	Monitoring ear health through a telemedicine-supported health screening service in Queensland	Smith et al. (2015)	Mobile facilities	Local Indigenous health worker	Patient information and clinical history<br>Digital images using video-otoscope<br>Hearing assessment<br>Health risk questionnaire<br>ENT Review
A182016	Reducing Loss to Follow-Up with Tele-audiology Diagnostic Evaluations.	Dharmar et al. (2016)	Hospital	Telepresenter	Patient history, visualization of external structures, video otoscopy, immittance (including high-frequency tympanometry and middle ear muscle reflexes), distortion product otoacoustic emissions, auditory brainstem response with air and bone conduction, and, when indicated, auditory steady-state response<br><br>All aspects conducted remotely<br><br>Parent/ Guardian Satisfaction Survey<br>Provider Survey
A192016	The Future of Healthcare Delivery : IPE / IPP Audiology and Nursing Student / Faculty Collaboration to Deliver Hearing Aids to Vulnerable Adults via Telehealth	Novak, Cantu, Zappler, Coco, & Champlin (2016)	Community clinic	Audiology students<br>Nursing students<br>Supervising faculty	Video otoscopy - nursing student in city 2<br>Survey - audiology student in city 1<br>Selection and fitting of hearing aids - nursing student in city 2<br>Remote programming of the hearing instruments - audiology student in city 1<br>Review the owner's manual, educate the patient on hearing aid

For peer review only

					parts, battery type/insertion/removal, and use of the push button volume control - nursing student in city 2<br>Real ear probe microphone measurements - audiology student in city 1
A202016	Infant Diagnostic Evaluation via Teleaudiology Following Newborn Screening in Eastern North Carolina	Stuart (2016)	Birthing centers	Remote Audiologist/ Technician<br>Clinical Telehealth Manager	Initial communication by call - option of Telemedicine or nearest diagnostic centre given<br><br>The diagnostic protocol:<br>Patient history<br>Cursory otoscopy<br>Middle-ear analysis<br>Distortion product otoacoustic emissions (DPOAEs)<br>Auditory brainstem response (ABR).
A212018	Cost and outcome of a community-based paediatric hearing screening programme in rural India with application of tele-audiology for follow-up diagnostic hearing assessment	Ramkumar et al. (2018)	Community clinic	Technician, Village health workers	DPOAE by VHW<br>Tele-ABR, in-person ABR
A222018	Comprehensive approach to the National Network of Teleaudiology in World Hearing Center in Kajetany, Poland	Skarżyński, Świerniak, Bruski, Ludwikowski, & Skarżyński (2018)	Community clinic	A monitoring clinician<br>A clinic engineer<br>Support speech specialist	Tele-consultation procedures<br><br>Remote fitting /Telefitting:<br>ART/NTR/NRI, eSRT<br>Programming<br><br>Telerehabilitation
A232019	Hearing Norton Sound: Mixed methods protocol of a community	Emmett, Robler, Gallo, et al. (2019)	School-based	Local Clinic staff<br>Teachers	Focus groups and community events held leading up to the

For peer review only

	randomised trial to address childhood hearing loss in rural Alaska			Audiologist<br>ENT Specialist	randomised trial<br>Qualitative, semi-structured interviews to elicit stakeholder perspectives on the intervention<br><br>Hearing screening day:<br>School screen<br>mHealth screen<br>Audiometric assessment<br>HEAR-QL<br>Outcomes measured on screening day:<br>Sensitivity and specificity of screening<br>Hearing loss prevalence<br>Hearing-related quality of life (HEAR-QL)
A242019	Hearing Norton Sound: a community randomised trial protocol to address childhood hearing loss in rural Alaska.	Emmett, Robler, Wang, et al. (2019)	School-based	Local Clinic staff<br>Teachers<br>Audiologist<br>ENT Specialist	Focus groups and community events held leading up to the randomised trial<br>Qualitative, semi-structured interviews to elicit stakeholder perspectives on the intervention (explanatory sequential stage<br><br>Hearing screening day:<br>School screen<br>mHealth screen<br>Audiometric assessment<br>HEAR-QL<br><br>Outcomes measured on screening day:<br>Sensitivity and specificity of screening

For peer review only

					Hearing loss prevalence<br>Hearing-related quality of life (HEAR-QL)
A252019	Comprehensive Community Screening of Otological Patients by Trained Technicians Using a Telemedicine Device: An Efficient and Cost-Effective Way to Triage Patients With Ear Diseases	Gupta et al. (2019)	Construction sites	Trained community health care workers	Screening, diagnosis, medical management, surgical intervention, and rehabilitation using hearing aid.<br><br>Video-otoscopy using ENTraview smartphone otoscopy device<br>Air conduction threshold screening at 4 speech frequencies: 500 Hz, 1000 Hz, 2000 Hz, and 4000 Hz.
A262019	Telehealth-enabled auditory brainstem response testing for infants living in rural communities: the British Columbia Early Hearing Program experience	Hatton, Rowlandson, Beers, & Small (2019)	Community clinics	An audiologist, audiometric technician (established hearing screeners), and a telehealth project manager	AC and BC ABR<br><br>Optional<br>Tympanometry testing with a 1000-Hz probe tone in case of elevated ABR thresholds<br>OAEs in case of SNHL<br><br>TH-ABR programme evaluation<br>Cost and time effectiveness<br>Measure of ABR test efficiency<br>Parent satisfaction
A272019	Implementation and evaluation of a rural community-based pediatric hearing screening program integrating in-person and tele-diagnostic auditory brainstem response (ABR)	Ramkumar, Nagarajan, Shankarnarayan, Kumaravelu, & Hall (2019)	Community clinic	Tele-technician<br>Village health workers	DPOAE by VHW<br>Tele-ABR, in-person ABR

For peer review only

A282019	Telefitting Between Kajetany, Poland and Odessa, Ukraine for Cochlear Implants	Skarżyński, Swierniak, Ludwikowski, & Bruski (2019)	Community clinic	A monitoring clinician<br>A clinic engineer<br>Support speech specialist	Remote fitting /Telefitting:<br>ART/NTR/NRI, eSRT<br>Programming
A292020	Cochlear Implant Mapping Through Telemedicine—A Feasibility Study	Luryi et al. (2020)	Community clinic	Local trained audiologist	Routine surveillance<br>Regular speech perception testing with AzBio sentence lists
A302020	Digital Proficiency Is Not a Significant Barrier for Taking Up Hearing Services With a Hybrid Online and Face-to-Face Model	Ratanjee-Vanmali, Swanepoel, & Laplante-Lévesque (2020)	Virtual clinic, home or office, or a satellite site for the clinic	Clinic Audiologist	Online Hearing Screening Test<br>Measures of Readiness and Stages of Change<br>Mobile Device and Computer Proficiency Questionnaires<br>Pure-Tone Average—Better Ear
A312020	Tele-Audiological Surveillance of Middle Ear Status among Individuals with Cleft Lip and/or Palate in Rural South India	Ravi et al. (2020)	Home-based<br>Camp based	Community workers<br>ENT consultant<br>Audiologist	Video-otoscopy<br>Pure tone audiometry<br>Tympanometry
A322020	Hearing Norton Sound: community involvement in the design of a mixed methods community randomized trial in 15 Alaska Native communities	Robler et al. (2020)	School-based	Local Clinic staff<br>Teachers<br>Audiologist<br>ENT Specialist	Focus groups and community events held leading up to the randomised trial<br>Qualitative, semi-structured interviews to elicit stakeholder perspectives on the intervention (explanatory sequential stage<br><br>Hearing screening day:<br>School screen<br>mHealth screen<br>Audiometric assessment<br>HEAR-QL<br>Outcomes measured on screening day:<br>Sensitivity and specificity of screening

For peer review only

					Hearing loss prevalence<br>Hearing-related quality of life (HEAR-QL)

For peer review only

Supplemental File – 9

Reference quotes from the semi-structured interviews

**Acceptability**	
1.	85% *(of the patients)* reported that they were very satisfied. Around 12% reported that they were satisfied. Some of them were not satisfied, but when we analyzed (*patient perspectives)* deeper, we found that this was reported only at the beginning. They wanted to have the first session on-site, at the centre, which we did. Then we found that the satisfaction level was over 95%.<br><br>- Project implementer of an ongoing telepractice service delivery program in Poland
2.	Parents were the best ambassadors of the idea when they saw that the results from the rehabilitation service were the best. So they were convinced of the method.<br><br>- Project implementer of a community-based telepractice service delivery program in India
3.	We have some testimony of parents saying, “This is awesome and it's motivational and it's informational. And I love that I didn't have to take my kid to *abc (referring to a distant location)* and get evaluated, then travel back and then travel down for surgery and travel back; I love that I could use this technology. I didn't have to travel or leave my community and my child is getting quick care and being seen.” And so we had a lot of positive reception around the technology in terms of the trial. Also, when we asked parents, “Do you want to be present for this follow-up?”. The idea was to try and make it very quick and not make it a scheduled appointment - not pull parents out of work to get it done. Like they've referred and then they got to the clinic and they got seen within a five-minute appointment. And so we had a lot of parents who said, “Yes, do it because it's hard for me. I got multiple kids at home or I'm working. And so that really helps me to make sure my kid gets seen.”<br><br>- Project implementer of an ongoing telepractice service delivery program in the US
4.	We felt it was essential to really capture narratives of the experience that we were investigating from community members and various stakeholders. One of the strengths of the trial was that we built a stakeholder group right away. There were multiple stakeholders on the ground, including individuals like myself who have served the region for a long time and who have become trusted members of that region. We interviewed teachers, special education teachers, school, administrators, hospital administrators, and community health workers, who are basically the on-the-ground eyes and ears in local community clinics - Parents and children, youth, elders in the community…… And so for us, that's been really helpful. We've also been working with the school district on the healthcare system to better refine the

For peer review only

	collaboration that's kind of required, to get this implemented without the research arm pushing it forward.<br><br>- Project implementer of an ongoing telepractice service delivery program in the US
5.	People were initially saying no. Then, initially, we did a lot of awareness activities. Wherever we planned to start the screening, for one week we put up posters, banners, and awareness activities. We went to the community and started with community dramas, for awareness, because nobody was ready to get screenings done. There were new dramas and other community activities done. There were 10- 20 days of awareness campaigns done so that they *(patients)* get convinced about getting the screening done because that behaviour has to be changed. The people over there need to understand what we are trying to do and what benefit they would get out of it. Then, we initiated these screenings door-to-door. Some people agreed based on that and more people followed. That's when screening happened.<br><br>- Project implementer of a community-based telepractice service delivery program in India
6.	So the people are seeing the mobile phone screen. So they want to know what their tympanic membrane looks like. So everybody began to say, "sir, please do it for me". Maybe in future, we can have visual feedback or a video about hearing and oral hygiene.<br><br>- Project implementer of an ongoing community-based telepractice service delivery program in South India
7.	We know that some of what we've done is not translatable to the general United States or even globally, but the *def (referring to the program)* is really a unique entity in that, healthcare is something that they are provided because their land has become part of the United States. So they receive *def* resources, and they had that as part of a single-payer system. So there was no cost to the parent or the end-user in terms of receiving this follow-up where potentially in another part of the United States this would potentially be something that would be billed to insurance or be a cost to a parent to receive.<br><br>- Project implementer of an ongoing telepractice service delivery program in the US
8.	One thing which we realized was that when you go to camps *(to conduct video-otoscopy)*, the patient expects something free of cost. So sometimes we ask the doctor if they can provide something in the camp. So ENTs always usually carry some kind of medication. Whatever is the medical treatment, the patients get it in the ground itself which is free of cost, and that also motivates them to come to the hospital.

	- Project implementer of a community-based telepractice service delivery program in India
9.	We have to do a better job of educating the patients and the consumers, the families that are accessing these services. We try to go through a lot of that orientation …... Making sure that we're setting appropriate expectations for the family that we're working with is very important.<br><br>- Project implementer of a long term telepractice service delivery program in the US
**Adoption**	
10.	Patients with some neurological issues, some rare diseases, are more demanding and in general, it is more difficult in terms of rehabilitation. So we try to help such patients on-site, in the clinic. For example, patients with mental disorders and after *(sustaining)* some trauma. If we have some risk group, we say, "No, sorry but you cannot use the telemedicine solution because the results would be not optimal."<br><br>- Project implementer of an ongoing telepractice service delivery program in Poland
11.	It's a lot of change management, which means you need the leadership teams and those clinics organizations and private practices to be wanting to change. It is a mind shift. It's the leadership team driving the vision to understand that you're not just adding technology to a patient journey. You're using technology to assist the patient and patient journey. You're not automating things for the sake of automating things. You're automating things so that the face-to-face appointments with the patient become a more enhanced conversation. For this model to be integrated into routine service delivery, each one can integrate the way that suits their context. But a mindset change and some amount of training to be able to make decisions on what might work best in terms of which components should be integrated are necessary.<br><br>- Project implementer of a hybrid telepractice service delivery program in South Africa
12.	Having the support of your institution or your hospital is critical - you need to know what you're doing so that they can get behind it. It can also be a way to reach the community, to get funding, to do other things - to demonstrate how you're serving the patient and the families that you're working with, in all of these different ways. So, the administrators certainly signed off on it; they saw the value in providing services to the families and the adults but also how it was going to enhance the training of the graduate students. I mean, that's our primary goal at the University - to teach and train the graduate students, so that when they leave they can become speech pathologists or audiologists. And so, it met all of these criteria - teaching, research and service

For peer review only

	delivery- these are the three areas that most universities are most concerned about. So we're able to demonstrate how having telepractice and having that lab satisfied all those areas.<br><br>- Project implementer of a long term telepractice service delivery program in the US
13.	The most interesting learning for me is the hesitancy of audiologists to embrace the model. I think that for me has been the most surprising, even through a COVID year. I think the audiologist's resistance and scepticism of such a model is interesting and maybe that's due to the fear of change. By no means, does the model remove the audiologist or remove the value of the audiologist; it is using technology to enhance the audiologist's value because you can take the conversation to a different level. You can use the time more efficiently.<br><br>- Project implementer of a hybrid telepractice service delivery program in South Africa
**Feasibility**	
14.	A lot of it has to do with management; there is planning, proper performance and looking for funds and adjusting the project according to the funds…….. I will advise future telepractice implementers to analyze the needs and the target group and to check in with the team which will be dedicated to the project, several times. Because there could be some gaps and misunderstandings, which could influence a non-successful project.<br><br>- Project implementer of an ongoing telepractice service delivery program in Poland
15.	This is a region that has a lot of middle ear diseases. We had to work with a school. We had to go to every school, we had to screen every child, and then the referral process. So, that was labour-intensive from a data collection standpoint for the study. Community health workers would say that "I don't want to do telemedicine because it's double the work. And, I'd rather just write it all down and then talk to a doctor on the phone." But what we see is that we don't always get the right diagnosis when that happens.<br><br>- Project implementer of an ongoing telepractice service delivery program in the US
16.	They (*technical and support staff)* try to work, they try to develop themselves and they earn money. I think salary is the first incentive and the second is the possibility of development……. So they work in something, which is a little bit unique. Of course, the money factor is important. Mostly, the incentive is looking at results. And

For peer review only

	the pay is also a good motivator.<br><br>- Project implementer of an ongoing telepractice service delivery program in Poland
17.	It's important to get that local connection. So that the patient, whenever he has some issues or some queries, can go directly to that person and get that sorted - that local connect is really important. It's also really important that they (*local liaison)* are able to put that commitment to work towards the community because there are very less people who want to work in communities and rural areas.<br><br>- Project implementer of a community-based telepractice service delivery program in India
18.	There were other individuals that we had brought in the stakeholder team; several native individuals from the community who served as our community outreach specialists. So she *(a community outreach specialist)* took a lot of what we were trying to say and created infographics to explain the trial in a way that through visuals and narrative. And so, I think that a lot of that helped us to gain trust….. I think we didn't experience as many cultural barriers because we went in the correct way. We identified our stakeholders right away; we used individuals who were from the region at the very beginning of the formation of the research question, and also while moving forward. And that really helps just kind of have it blend in seamlessly. Having enough focus groups in the beginning, generating awareness, bringing insight into the design of the trial. And a documentary narrative really helped us in getting the story that different stakeholders are telling…. I didn't see a lot of cultural barriers. But I think that's because we brought those people in.<br><br>- Project implementer of an ongoing telepractice service delivery program in the US
19.	You need to have a team - not only consisting of doctors and engineers, but also speech therapists. So we talk to audiophonologists and a whole management team starting from nurses and people who are dedicated to patient service; they could even be students. And this is quite important - to have that organizational service. It can not work with only one or two specialists. So having a team who worked earlier in medical centres, who know how to cope with patients, and can be dedicated to such a project. And there is no must for them to have a medical higher graduation.<br><br>- Project implementer of an ongoing telepractice service delivery program in Poland
20.	In some instances, in some communities, it worked fabulously because the school and the clinic already do a lot of hand-in-hand care. Those communication channels were well developed and seamless.

For peer review only

	- Project implementer of an ongoing telepractice service delivery program in the US
21.	The program is unique and is growing. It was designed and came out of sheer need and it basically has local community members in a trained program. They complete some initial tests - to check if they have enough reading and basic math skills. And then once they're in the program, then it routes them through a series of certifications. And so there are five levels of community provider. So one, two, three, four, and five as a practitioner, and then they learn different skills along each way. And so they are so integrated into healthcare that it's unbelievable. I'm involved in the training of our onboarding audiologists. I give them several cultural materials to read. I want them to come in with a very humble sense of the culture. They go through a three-day orientation of the hospital, which also is pretty culturally intensive in terms of nativity and heritage. And then we do a lot of on-the-job training from a telemedicine perspective. So that's one of the first things they learn because we do so much of it.<br><br>- Project implementer of an ongoing telepractice service delivery program in the US
22.	The instrument has to be charged completely for hours together, continuously. We were able to use it only for a maximum of four hours continuously because it has to support the LED light source and the mobile phone. And also, it'll consume more power to transfer data to the cloud by using the network. So there was a challenge in using the instrument continuously. It was not very user friendly initially, but after some time we could adapt it after the initial phase. Also, the equipment had texts that were only in English. We requested the company to modify it into the local language so that the community-based health workers are able to also read. So these were the challenges we faced initially, these were the difficulties.<br><br>- Project implementer of an ongoing community-based telepractice service delivery in South India
23.	I think that maybe the one important thing is having connectivity and that's the biggest issue right now. There's still sort of blackout areas where there's just very little connectivity or very very slow connectivity and that makes telepractice sometimes impossible to do. Most families do have internet services to some degree, but we still have some families that do not have internet. And that's the biggest problem that we run into, but not the most frequent. To figure out how they can get access to the connectivity can be a challenge from time to time. That probably happens 1% of the time; 99% of the time, they do have connectivity.<br><br>- Project implementer of a long term telepractice service delivery program in the US

For peer review only

24.	Undersea cables and just the continued expanse of getting closer and closer to broadband in our region has been pretty incredible, and obviously essential for this type of stuff.<br><br>- Project implementer of an ongoing telepractice service delivery program in the US
25.	Because in remote areas, the internet is still not available sometimes, it's usually store and forward technology that's used because it's much better than waiting for the community workers to come back and do it. It helps us save battery to do much more screenings and operationally that's viable also.<br><br>- Project implementer of a community-based telepractice service delivery program in India
26.	We have to be available to where the patients are and what devices they're using and not what we want them to use. Being able to connect to what they already have is going to be critical and going forward anyone starting a telepractice or telehealth program needs to realize that and that's sort of one of the best practices.<br><br>- Project implementer of a long term telepractice service delivery program in the US
27.	What's unique about *yyy (referring to a state in the US)* is that we've depended upon telemedicine early on, and so unique to conducting this trial in rural *yyy (referring to a state in the US)* is that we were building upon an established network and an established infrastructure on telemedicine. And so the goal of the study was really to use those resources that were available to us so that we knew when it came time to a sustainable solution that will continue beyond the research it's possible. We tapped into already existing infrastructure that people understand and know how to use, which was an asset for us both from completion and also a sustainability point of view. We did tweak it significantly to make it feasible.<br><br>- Project implementer of an ongoing telepractice service delivery program in the US
28.	If it wasn't the patient themselves who had a phone, it was the significant other or the caretaker. So I think digital proficiency is not a barrier. I think that's the assumption we have as audiologists, but there's always a support network around. Not always, but most of the time, there's a support network around the patient where technology is available.<br><br>- Project implementer of a hybrid telepractice service delivery program in South Africa

For peer review only

29.	I think we have about seven states that have passed this legislature of COMPACT now and I think when we get to *zzz (referring to a district in the US)*, then the COMPACT will go into effect. If you are in your home state and you're licensed in one of those states as a member of the COMPACT, then you can practice in any of the other states that are in the COMPACT. Though it is not an answer for everyone yet, these kinds of ideas have been out there and people have been trying to push those at the state level to get some of the legislation passed.<br><br>- Project implementer of a long term telepractice service delivery program in the US
30.	There are about eight different bills in the US Congress to make some changes permanent; to have more access to telehealth and telepractice and to have reimbursement available. So hopefully, one positive thing that may come out of this pandemic is the availability of telehealth and telepractice, but also the reimbursement that would be there for the providers to be able to provide these services.<br><br>- Project implementer of a long term telepractice service delivery program in the US
**Implementation Cost**	
31.	There was an initial investment cost and we bought high-end digital audiometers, we had a website etc. But it was the funding that allowed us to start a digital-first company. So, those costs come into play if you're starting from the ground up.<br><br>- Project implementer of a hybrid telepractice service delivery program in South Africa
32.	After an issue, an instrument got repaired and we didn't get funds to repair the instrument immediately. So suddenly, the process of intervention for hearing disorders stopped in the project. After some time, we got the funds, and we were able to procure new equipment.<br><br>- Project implementer of an ongoing community-based telepractice service delivery program in South India
33.	So Medicaid in *xxx (referring to a state in the US)* did not reimburse telepractice. The only time that they would reimburse for telepractice was if the SLP or whoever was delivering the services to a public school district and working with a child who was on Medicaid and receiving those funds. And so, no other funding of telepractice before Covid.<br><br>- Project implementer of a long term telepractice service delivery program in the US

For peer review only

34.	(In) the beginning, funds for the National Network of Teleaudiology were partially funded by the Norwegian funds …… for European Union. So in the beginning, it was from them. Some of the local projects are co-financed through different scientific grants. Always, we need to have our internal (financial) part, which we need to finance from our sources, which come from the services (we provide). ………. and some of them it's on a rental basis. So we don't buy flat, but we rent …. equipment…. Really, it's not easy.<br><br>- Project implementer of an ongoing telepractice service delivery program in Poland
35.	The biggest challenge was some blockage from the local insurance government, which didn't want to give us more financial support. So we have had limitations concerning that and we still have them sometimes. So, in such cases, we need to tell patients that they need to choose a private solution that they need to pay for. So it's not very comfortable for them either.<br><br>- Project implementer of an ongoing telepractice service delivery program in Poland
36.	I worked hard with all the stakeholders from the hospital to ensure that that encounter was billable, because if you're doing something that's not billable from a hospital perspective, then it's not sustainable.<br><br>- Project implementer of an ongoing telepractice service delivery program in the US
**<u>Sustainability</u>**	
37.	The first thing that we did to address sustainability was really try to utilize what was already existing in terms of technology and resources, and considered all the things that may make that encounter billable, so that the hospital could continue to provide that resource following the research project…. from a sustainability piece, we involved people who are already established resources. So, we use the health aids that were already in the clinic; there was no extra resources there. And then we used special education teachers already doing the screenings in the school so that, once we were done, it wouldn't be too abnormal for this process to continue.<br><br>Project implementer of an ongoing telepractice service delivery program in the US
38.	Having the support of the faculty and administrators, I think you know has been very positive over the years….the administrators, the colleagues, the cochlear implant surgeons and the pediatric audiologists I work with, I think they've been very supportive as well with the same kind of mindset of that we need to find different

	ways of serving these patients… so those are the kind of things that I think (*that influence sustainability)* - having the support of your institution or your hospital is really critical and that you know what you're doing and they can get behind it.<br><br>Project implementer of a long term telepractice service delivery program in the US

For peer review only
